# The MarR-Type Repressor MhqR Confers Quinone and Antimicrobial Resistance in *Staphylococcus aureus*

**DOI:** 10.1089/ars.2019.7750

**Published:** 2019-10-17

**Authors:** Verena Nadin Fritsch, Vu Van Loi, Tobias Busche, Anna Sommer, Karsten Tedin, Dennis J. Nürnberg, Jörn Kalinowski, Jörg Bernhardt, Marcus Fulde, Haike Antelmann

**Affiliations:** ^1^ Institute of Biology–Microbiology, Freie Universität Berlin, Berlin, Germany.; ^2^ Center for Biotechnology, Bielefeld University, Bielefeld, Germany.; ^3^ Institute of Microbiology and Epizootics, Freie Universität Berlin, Berlin, Germany.; ^4^ Institute of Experimental Physics, Freie Universität Berlin, Berlin, Germany.; ^5^ Institute for Microbiology, University of Greifswald, Greifswald, Germany.

**Keywords:** *Staphylococcus aureus*, MhqR, QsrR, quinones, antimicrobial resistance

## Abstract

***Aims:*** Quinone compounds are electron carriers and have antimicrobial and toxic properties due to their mode of actions as electrophiles and oxidants. However, the regulatory mechanism of quinone resistance is less well understood in the pathogen *Staphylococcus aureus*.

***Results:*** Methylhydroquinone (MHQ) caused a thiol-specific oxidative and electrophile stress response in the *S. aureus* transcriptome as revealed by the induction of the PerR, QsrR, CstR, CtsR, and HrcA regulons. The *SACOL2531-29* operon was most strongly upregulated by MHQ and was renamed as *mhqRED* operon based on its homology to the *Bacillus subtilis* locus. Here, we characterized the MarR-type regulator MhqR (SACOL2531) as quinone-sensing repressor of the *mhqRED* operon, which confers quinone and antimicrobial resistance in *S. aureus*. The *mhqRED* operon responds specifically to MHQ and less pronounced to pyocyanin and ciprofloxacin, but not to reactive oxygen species (ROS), hypochlorous acid, or aldehydes. The MhqR repressor binds specifically to a 9–9 bp inverted repeat (MhqR operator) upstream of the *mhqRED* operon and is inactivated by MHQ *in vitro*, which does not involve a thiol-based mechanism. In phenotypic assays, the *mhqR* deletion mutant was resistant to MHQ and quinone-like antimicrobial compounds, including pyocyanin, ciprofloxacin, norfloxacin, and rifampicin. In addition, the *mhqR* mutant was sensitive to sublethal ROS and 24 h post-macrophage infections but acquired an improved survival under lethal ROS stress and after long-term infections.

***Innovation:*** Our results provide a link between quinone and antimicrobial resistance *via* the MhqR regulon of *S. aureus*.

***Conclusion:*** The MhqR regulon was identified as a novel resistance mechanism towards quinone-like antimicrobials and contributes to virulence of *S. aureus* under long-term infections.

## Introduction

*S**taphylococcus aureus* is a major human pathogen, which can cause several diseases including life-threatening systemic and chronic infections, such as sepsis, necrotizing pneumonia, or endocarditis (3, 8, 47).The increasing prevalence of multiple antibiotic resistant strains, such as methicillin-resistant *S. aureus*, leads to treatment failure and high mortality rates (15, 54). Understanding the defense and resistance mechanisms of *S. aureus* to antibiotics and the host immune response, including reactive oxygen species (ROS) and reactive electrophilic species, will lead to the discovery of novel resistance mechanisms and potential new drug targets to combat multiple antimicrobial resistance.

Quinones are essential lipid electron carriers of the aerobic and anaerobic respiratory chain in bacteria (*e.g.*, ubiquinone and menaquinone) (31, 39, 71). However, many natural antimicrobial compounds contain quinone-like structures that are encountered as exogenous sources of quinone stress in pathogenic bacteria, such as the fungal 6-brom-2-vinyl-chroman-4-on (55) or the plant-derived 1,4-naphthoquinone lapachol (32). The toxic effect of quinones is caused by their electrophilic and oxidative modes of actions (35, 52, 57). Quinones have electron-deficient carbon centers and react as electrophiles with the nucleophilic thiol groups of cysteines *via* irreversible thiol-*S*-alkylations, leading to aggregation and depletion of thiol-containing proteins in the proteome (43). As oxidants, quinones can form highly reactive semiquinone radicals that subsequently promote ROS generation, such as superoxide anions, which in turn cause reversible thiol oxidations in proteins (7, 35, 52, 57).

In *Bacillus subtilis*, two MarR/DUF24 family regulators YodB and CatR as well as the MarR-type repressor MhqR respond to quinones and the azo compound diamide and control together paralogous quinone or azo compound reductases (AzoR1, AzoR2), nitroreductases (YodC, MhqN), and ring-cleavage dioxygenases (MhqA, MhqE, MhqO, CatE) for quinone detoxification (1, 2, 13, 29, 42, 69). The YodB- and MhqR-regulated quinone reductases have been shown to confer additive resistance to quinones and diamide in *B. subtilis* and function in quinone and diamide reduction to hydroquinones and dimethylurea, respectively. The thiol-dependent dioxygenases catalyze the ring cleavage of quinone-*S*-adducts formed by reaction with low-molecular-weight thiols, such as bacillithiol (BSH) (9). Apart from its role in detoxification of exogenous quinones, the catechol 2,3-dioxygenase CatE was recently shown to function in recycling of the endogenous catecholate siderophore bacillibactin under iron limitation in *B. subtilis* (65).

Furthermore, the *mhqR* mutant supported the growth of cell wall-deficient l-forms in *B. subtilis*, which are resistant to β-lactam antibiotics and promote persister formation (17, 34). The constitutive expression of quinone detoxification genes in the *mhqR* mutant was suggested to decrease respiratory chain activity and to limit ROS production as mechanism of l-form growth (34).

Innovation The adaptation strategies of *Staphylococcus aureus* toward reactive oxygen species and reactive electrophilic species are not fully understood, which are required for the successful infection and establishment of antibiotics resistance. In this work, we characterized the novel MhqR repressor as important quinone-sensing and regulatory mechanism in *S. aureus*, which controls quinone detoxification genes and conferred resistance to quinones and quinone-like antimicrobial compounds, including fluoroquinolones (ciprofloxacin, norfloxacin), rifampicin, and pyocyanin. The *mhqR* mutation further caused an increased survival of *S. aureus* during long-term macrophage infections, and thus, the enzymes of MhqR regulon could be possible drug targets.

YodB and CatR are redox-sensing repressors that sense and respond directly to quinones by a redox-switch mechanism involving thiol oxidation at the conserved Cys6 and Cys7 residues, respectively (12, 13). The YodB repressor forms intermolecular disulfides between Cys6 and the C-terminal Cys101 or Cys108 in the opposing subunits of the YodB dimer under quinone and diamide stress *in vitro* and *in vivo* (12, 41). However, the mechanism of MhqR regulation under quinone stress is unknown thus far and may not involve a thiol-switch mechanism (69).

In *S. aureus*, the YodB homologue QsrR has been ascribed to be implicated in quinone detoxification, which controls related quinone reductases and a nitroreductase, an flavin mononucleotide-linked monooxygenase, and thiol-dependent dioxygenases (33). The crystal structure of quinone-modified QsrR has been resolved, and the redox-regulatory mechanism was shown to involve thiol-*S*-alkylation of the conserved Cys5 by quinones *in vitro* (33). Importantly, the QsrR regulon was essential for the pathogenicity of *S. aureus* leading to reduced phagocytosis and increased resistance against killing by bone marrow-derived macrophages (33).

In this work, we aimed to further investigate the quinone-stress-specific response in *S. aureus* to elucidate novel mechanisms of redox signaling and antimicrobial resistance. Using RNA-seq transcriptomics, we identified the *mhqRED* operon as most strongly induced by methylhydroquinone (MHQ) in *S. aureus*, which is controlled by SACOL2531 (MhqR), a close homolog to MhqR of *B. subtilis* (69). Our results demonstrate that the *mhqRED* operon confers resistance to quinones and quinone-like antimicrobials, including pyocyanin, ciprofloxacin, norfloxacin, and rifampicin. Due to the increasing prevalence of multiple antibiotic resistant *S. aureus* isolates, these results are important to understand the underlying mechanisms of antimicrobial resistance.

## Results

### MHQ elicits a thiol-specific oxidative, electrophile, and metal stress response in the RNA-seq transcriptome of *S. aureus*

**FIG. 1.**
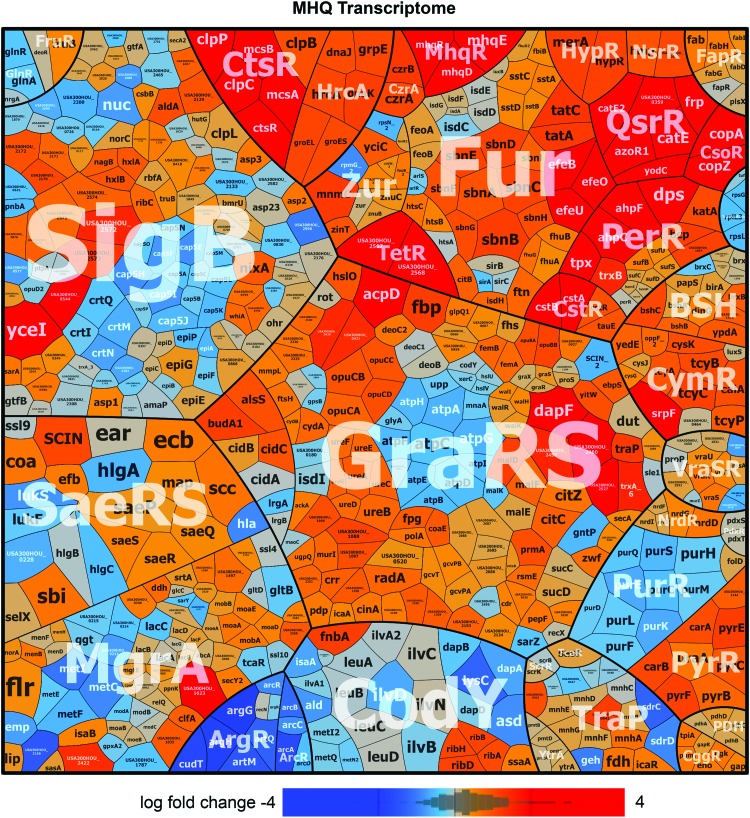
**The transcriptome treemap of *Staphylococcus aureus* COL under MHQ stress indicates a strong upregulation of the MhqR and QsrR regulons.** The transcriptome treemap shows the differential gene expression of *S. aureus* after exposure to 45 μ*M* MHQ as log2-fold-changes (*M*-values). The genes are classified into operons and regulons based on the RegPrecise database and previous publications (44, 49, 72). Differential gene expression is visualized using a *red–blue color code* where *red* indicates log2-fold induction and *blue* indicates repression of transcription under MHQ stress. The quinone-stress-specific regulons MhqR and QsrR are most strongly upregulated under MHQ stress in *S. aureus* COL. The induction of the PerR, CsoR, Fur, HrcA, CtsR, and GraRS regulons reveals an oxidative, electrophile, metal, and cell wall stress response and protein damage in *S. aureus*. The RNA-seq expression data of the selected highly transcribed genes after MHQ stress and their regulon classifications are listed in Supplementary Table S2. MHQ, methylhydroquinone. Color images are available online.

**FIG. 2.**
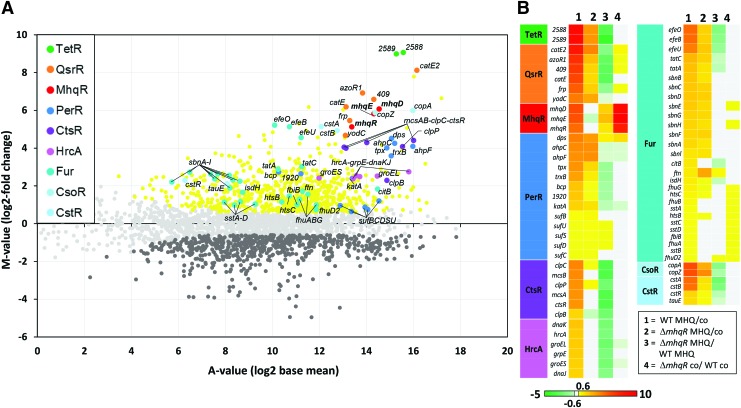
**RNA-seq transcriptomics of *S. aureus* COL wild type and the *mhqR* mutant under MHQ stress.** For RNA-seq transcriptomics, *S. aureus* COL and the *mhqR* mutant were grown in RPMI1640 medium and treated with 45 μ*M* MHQ stress for 30 min. **(A)** The gene expression profile of the wild type under MHQ stress is shown as ratio/intensity scatterplot (M/A-plot), which is based on the differential gene expression analysis using DeSeq2 (46). *Colored symbols* indicate significantly induced (*red*, *orange*, *yellow*, *blue*, *cyan*, *violet*, *green*) or repressed (*dark gray*) transcripts (*M*-value ≥0.6 or ≤−0.6; *p* ≤ 0.05). *Light gray symbols* denote transcripts with no fold-changes after MHQ stress (*p* > 0.05). The TetR, QsrR, MhqR, PerR, CtsR, HrcA, Fur, CsoR, and CstR regulons are most strongly upregulated under MHQ stress. **(B)** The *color-coded heat map* displays log2-fold-changes of gene expression between the wild type and the *mhqR* mutant under control and MHQ. *Red* and *green* indicate significantly induced and repressed transcripts (*M*-value ≥0.6 or ≤ −0.6; *p* ≤ 0.05) in three biological replicates, respectively. The RNA-seq expression data of all genes under MHQ stress and their regulon classifications are listed in Supplementary Tables S1 and S2. Color images are available online.

To investigate the quinone-stress-specific response of *S. aureus* COL, we analyzed the changes in the RNA-seq transcriptome after exposure to sublethal MHQ stress (45 μ*M*) (Supplementary Fig. S1) (30, 44). For significant fold-changes, the *M*-value cutoff (log2-fold-change MHQ *vs.* control) of ±0.6 was chosen (adjusted *p*-value ≤0.05). In total, 730 transcripts were significantly >1.5-fold upregulated and 675 were >1.5-fold downregulated in the transcriptome of *S. aureus* under MHQ stress (Supplementary Tables S1 and S2). A subset of the most strongly upregulated regulons is displayed in the Voronoi transcriptome treemap (Fig. 1). About 70 genes displayed the highest fold-changes under MHQ stress ranging from 10 to 536 (*M*-values of 3.3–9), which could be mainly allocated to the TetR, QsrR, PerR, Fur, CtsR, CstR, CsoR, SigB, and GraRS regulons (Figs. 1 and 2 and Supplementary Fig. S2; Supplementary Tables S1 and S2). This indicates that MHQ leads generally to a strong thiol-specific oxidative (PerR), electrophile (QsrR), metal (Fur, CsoR), and cell wall stress response (GraRS, SigB) in *S. aureus*.

Among the top hits was the *SACOL2588-89* operon of hypothetical functions (510- to 536-fold) and the QsrR regulon, including *SACOL2533* (*catE2*), *SACOL0408-09-10* (*catE-SACOL0409-azoR1*), *SACOL2534* (*frp*), and *SACOL2020* (*yodC*) (25- to 121-fold induced). Interestingly, our transcriptome data revealed also a strong (35- to 67-fold) upregulation of the *SACOL2531-30-29* operon that encodes for the phospholipase/carboxylesterase SACOL2529 (MhqD), the dioxygenase SACOL2530 (MhqE), and the unknown MarR-type regulator SACOL2531. SACOL2531 showed striking homology (39.4% sequence identity) to the quinone-specific MhqR repressor of *B. subtilis* (69) and was renamed MhqR in *S. aureus* (Supplementary Fig. S3A). Thus, the transcriptome results identified QsrR and MhqR as most responsive to MHQ in *S. aureus*, which resembles the quinone stress response in *B. subtilis* (1, 18, 29, 42, 55, 68, 69).

We have previously analyzed the transcriptome signature of *S. aureus* USA300 in response to the strong oxidant sodium hypochlorite (NaOCl) and the antimicrobial surface coating AgXX^®^, which causes ROS formation, such as hydroxyl radicals (44, 72). Our RNA-seq data after MHQ treatment showed a similar expression profile by the strong induction of the PerR, QsrR, Fur, CsoR, HrcA, CtsR, and GraRS regulons, as observed under NaOCl and AGXX stress (44, 72). This signature is indicative for a thiol-specific oxidative, electrophile, metal, and cell wall stress response as well as protein damage.

Specifically, MHQ leads to induction of the CtsR-controlled Clp proteases, including *clpP* (17-fold) and the *ctsR-mcsA-mcsB-clpC* operon (16- to 21-fold) involved in protein quality control and proteolytic degradation of quinone-aggregated proteins (1). The PerR, Fur, and CsoR regulons function in ROS detoxification, iron or copper homeostasis, and these metalloregulatory proteins have oxidation-sensitive metal binding sites (5, 23). The CstR regulon responds to reactive sulfur species and thiol persulfides (48). Transcription of the genes for cysteine and bacillithiol metabolism (*cysK*, *bshA* operon, *bshB*, *bshC*, *brxB*, and *ypdA*) was 1.6- to 5.6-fold elevated by MHQ in *S. aureus* supporting the thiol-reactive mode of action of quinones, which affects the cellular thiol-redox homeostasis (67). About 87 genes of the GraRS regulon and parts of the SigB regulon were upregulated by MHQ, which function in the cell wall and general stress response as well as in the oxidative stress defense (21).

However, the SigB-dependent *crtNMQIO* operon for staphyloxanthin biosynthesis and the capsule biosynthesis *cap5ABCDEFGHIJKLMNOP* operon were strongly repressed by MHQ (Fig. 1 and Supplementary Tables S1 and S2). Among the downregulated regulons were further the arginine biosynthesis ArgR regulon, including the *argBJCD*, *argHG*, and *artQM* operons, as well as the arginine catabolic ArcR regulon, controlling the arginine deiminase *arcCDBA* operon. In addition, the purine biosynthesis PurR regulon was downregulated by MHQ, which might be attributed to the reduced growth rate under sublethal MHQ (Supplementary Fig. S1). Altogether, the transcriptome signature of MHQ resembles the thiol-specific oxidative, electrophile, and metal stress response and identified the *mhqRED* operon as novel quinone-regulatory system that was selected for further study.

### The MhqR repressor senses quinones and controls the specific expression of the *mhqRED* operon in *S. aureus*

**FIG. 3.**
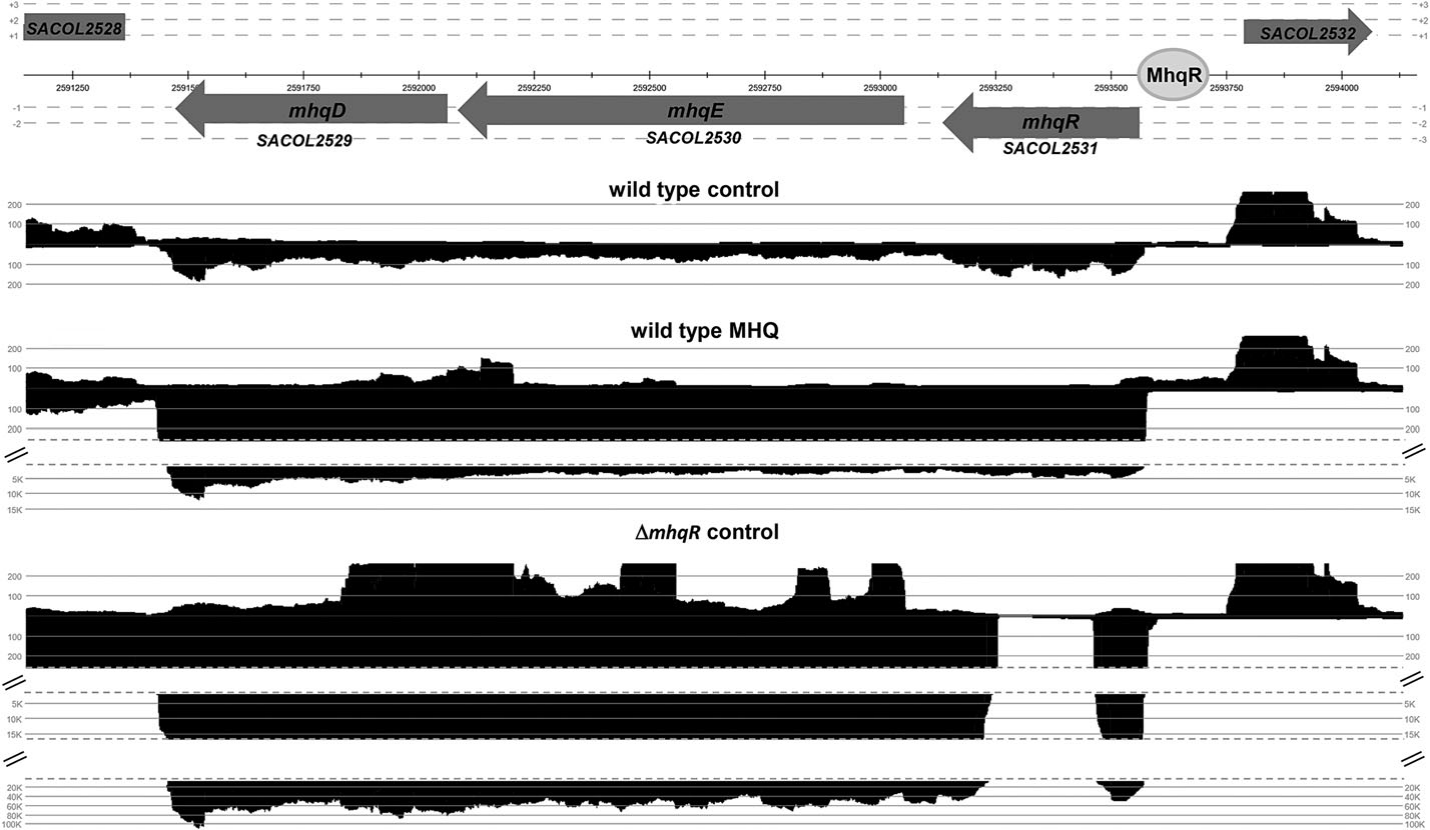
**The *mhqRED* operon (SACOL2531-2529) is strongly upregulated in the RNA-seq transcriptome of *S. aureus* COL under MHQ stress and fully derepressed in the *mhqR* mutant.** The mapped cDNA reads for the transcription profile of the *mhqRED* locus under control and MHQ stress are shown as displayed in Read Explorer (28). Transcription of the *mhqRED* operon is 35- to 67-fold induced under MHQ stress in *S. aureus* COL and most strongly derepressed in the *mhqR* mutant under control conditions (206- to 891-fold). Thus, *mhqR* encodes for a MarR-type transcriptional repressor of the *mhqE* and *mhqD* genes that encode for a dioxygenase and phospholipase/carboxylesterase, respectively.

We conducted RNA-Seq transcriptomics of a *mhqR* deletion mutant to identify the genes of the MhqR regulon. The *mhqE and mhqD* genes were most strongly upregulated (206.5- to 891.4-fold) under control conditions in the *mhqR* mutant transcriptome, indicating that MhqR represses transcription of the *mhqRED* operon in the wild type (Figs. 2 and 3 and Supplementary Fig. S2; Supplementary Table S2). MhqE and MhqD showed 35.4% and 38.8% sequence identity to the homologous dioxygenase MhqE and phospholipase/carboxylesterase MhqD of *B. subtilis*, respectively (Supplementary Fig. S3B). In contrast to *B. subtilis*, MhqR only controls the *mhqRED* operon in *S. aureus* (Fig. 2 and Supplementary Fig. S2; Supplementary Table S2) (69).

The transcriptome results of the *mhqR* mutant further revealed that most thiol-specific oxidative and electrophile stress regulons (*e.g.*, HypR, QsrR, and PerR) are expressed at a lower basal level under control conditions in the *mhqR* mutant compared with the wild type. For example, peroxide scavenging peroxiredoxins and catalases (*ahpCF* and *katA*) showed twofold lower basal level expression in the *mhqR* mutant compared with the wild type (Fig. 2 and Supplementary Fig. S2; Supplementary Table S2). This lower basal expression of antioxidant and quinone detoxification regulons might be due to the quinone-resistant phenotype of the *mhqR* mutant enabling faster quinone detoxification, which leads to lower basal levels of ROS. Consequently, the quinone and oxidative stress responsive HypR, QsrR, and PerR regulons and genes required for low molecular weight thiol biosynthesis (Cys, BSH) were only weakly upregulated in the *mhqR* mutant under MHQ treatment due to its higher tolerance for quinones. Similarly, the *mhqR* mutant displayed decreased fold-changes under MHQ for the majority of members of the cell wall, sulfide, and metal stress-sensing SigB, GraRS, CsoR, and CstR regulons (Fig. 2 and Supplementary Fig. S2; Supplementary Table S2). Moreover, the expression of the CtsR- and HrcA-controlled protein quality control machinery was >5-fold decreased under MHQ stress in the *mhqR* mutant. In conclusion, constitutive derepression of the MhqR regulon in the *mhqR* mutant leads to higher quinone detoxification capability, which limits ROS generation and the resulting protein oxidation and damage.

### The *mhqRED* operon responds specifically to quinones and the antimicrobials ciprofloxacin, pyocyanin, and lapachol in *S. aureus*

**FIG. 4.**
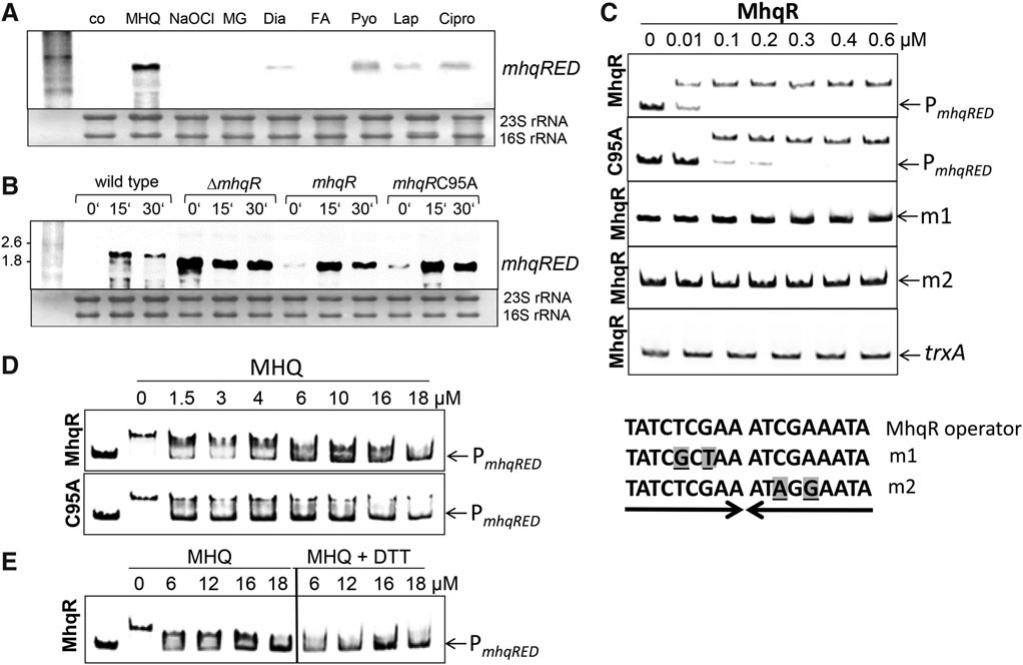
**Transcriptional induction of the MhqR regulon under quinones, aldehydes, and antimicrobials and the quinone response of MhqR in DNA binding assays *in vitro*. (A)** Transcription of the *mhqRED* operon was analyzed using the Northern blots in *S. aureus* COL wild type 30 min after exposure to 45 μ*M* MHQ, 1 m*M* NaOCl, 0.5 m*M* methylglyoxal (MG), 2 m*M* diamide (Dia), 0.75 m*M* formaldehyde (FA), 300 μ*M* lapachol (Lap), 90.5 μ*M* ciprofloxacin (Cipro), and 76 μ*M* pyocyanin (Pyo). The compounds were added at an OD_500_ of 0.5. The *mhqRED* operon responds most strongly to MHQ and less strongly to lapachol, pyocyanin, and ciprofloxacin. **(B)** The Northern blot analysis was performed with RNA of the wild type, the *mhqR* mutant, and the *mhqR* and *mhqRC95A* complemented strains before (0 min) and 15 and 30 min after MHQ stress. Cys95 is not required for DNA binding and quinone sensing of MhqR *in vivo*. The methylene blue stain is the RNA loading control indicating the 16S and 23S rRNAs. **(C)** MhqR binds specifically to the *mhqRED* promoter *in vitro*. EMSAs were used to analyze the DNA binding activity of increasing amounts (0.01–0.6 μ*M*) of MhqR and MhqRC95A proteins to the *mhqRED* promoter (P_*mhqRED*_) *in vitro*. To test the specificity of binding, two base substitutions were introduced in each half of the inverted repeat, denoted in *gray* and *underlined* (m1 and m2). As nonspecific control DNA probe we used the *trxA* gene. The *arrows* denote the free DNA probe and the *shifted band* indicates the DNA-MhqR promoter complex. **(D)** EMSAs of MhqR and MhqRC95A proteins (0.6 μ*M*) to the *mhqRED* promoter were performed to study the inactivation of MhqR by increasing amounts of MHQ (1.5–18 μ*M*) leading to the loss of DNA binding. The *arrows* denote the free *mhqRED* promoter probe and the *shifted band* indicates the DNA-MhqR promoter complex. **(E)** MhqR inactivation by quinones could not be reversed with 1 m*M* DTT, which was added to the MhqR-DNA binding reaction 30 min after MHQ addition. Cys95 is not important for MHQ sensing or DNA binding of MhqR *in vitro*. DTT, dithiothreitol; EMSA, electrophoretic mobility shift assay; NaOCl, sodium hypochlorite.

Next, we conducted Northern blot analysis to study *mhqRED* transcription in *S. aureus* COL under different stress conditions and antibiotic treatment, including 45 μ*M* MHQ, 1 m*M* NaOCl, 2 m*M* diamide, 0.75 m*M* formaldehyde, 0.5 m*M* methylglyoxal, 300 μ*M* lapachol, 76 μ*M* pyocyanin, and 90.5 μ*M* ciprofloxacin (Fig. 4A). The Northern blot results revealed that the *mhqRED* operon is most strongly induced by MHQ stress but does not respond to NaOCl and aldehydes. Interestingly, increased transcription of *mhqRED* operon was also found by the quinone-like antimicrobials, such as ciprofloxacin, pyocyanin, and the 1,4-naphthoquinone lapachol (Fig. 4A; Supplementary Fig. S4). Thus, the MhqR regulon responds specifically to quinones and diverse quinone-like antimicrobials in *S. aureus*, suggesting a function in antimicrobial resistance.

### The DNA binding activity of MhqR is inhibited by quinones *in vivo* and *in vitro*, which does not involve a thiol-based mechanism

*S. aureus* MhqR harbors a nonconserved Cys at position 95. To examine the role of Cys95 for DNA binding and quinone sensing, we complemented the *mhqR* mutant with plasmid-encoded *mhqR* and the *mhqRC95A* mutant allele. The Northern blot analyses confirmed the constitutive expression of the 1.7 kb truncated *mhqRED*-specific mRNA in the *mhqR* mutant. Complementation of the *mhqR* mutant with *mhqR* restored repression of transcription of the *mhqRED* operon under control conditions and the strong quinone response to wild-type level (Fig. 4B). However, the *mhqRC95A* mutant also showed the same low basal level transcription and strong responsiveness to MHQ of the *mhqRED* operon compared with the wild type and *mhqR* complemented strain. Thus, the Northern blot data revealed that Cys95 is neither required for DNA binding nor for quinone sensing *in vivo*.

Electrophoretic mobility shift assays (EMSAs) were used to investigate the DNA binding activity of purified MhqR protein to the *mhqRED* promoter *in vitro*. The *mhqRED*-specific promoter probe covered the region from +32 to −192 relative to the transcription start site (TSS). The gel shift results showed that purified MhqR binds to the *mhqRED* promoter probe, which is indicated by the band shift in the DNA binding reactions with MhqR (Fig. 4C).

**FIG. 5.**
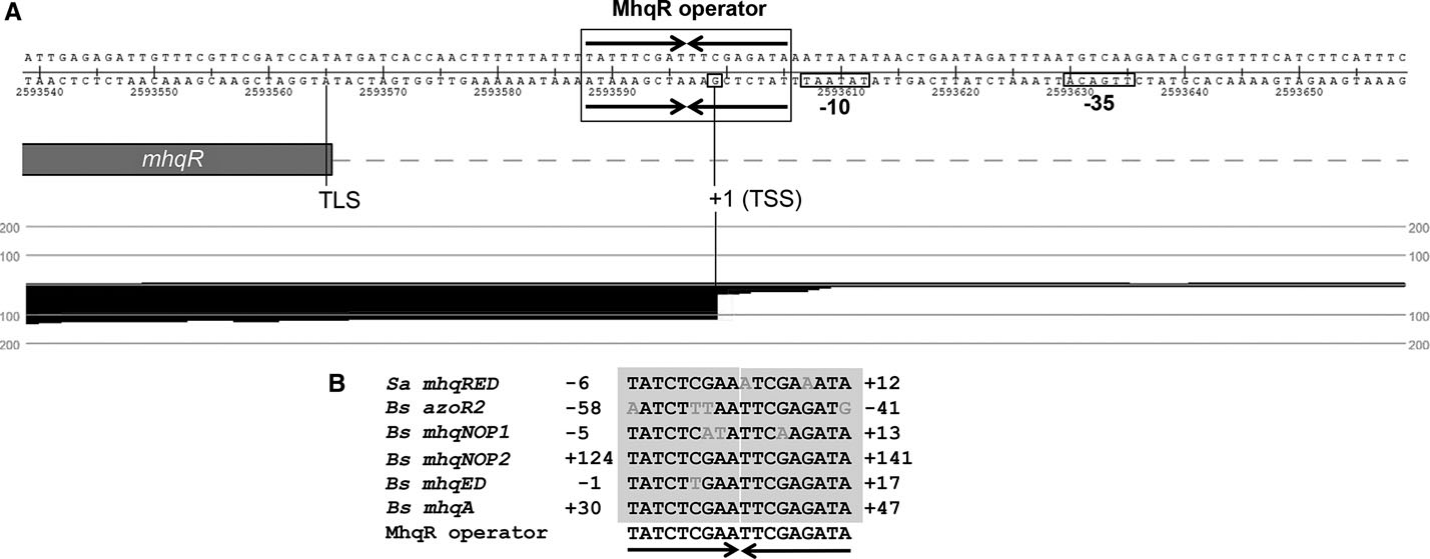
**TSS annotation of the *mhqRED* mRNA with the 9–9 bp inverted repeat as operator site for the MhqR repressor in *S. aureus*. (A)** The upstream promoter region of the *mhqRED* operon of *S. aureus* contains a 9–9 bp palindrome as MhqR operator (denoted with *boxes*) in position −6 to +12 relative to the TSS that is highly conserved upstream of *azoR2*, *mhqNOP*, *mhqED*, and *mhqA* of the MhqR regulon in *Bacillus subtilis* (69). The mapped reads enriched for primary 5′-transcripts of *S. aureus* USA300 transcriptome under control conditions are displayed for the 5′-end of *mhqRED* operon using Read Explorer as described in the Experimental Procedures section. The −10 and −35 promoter sequences, the TSS, and the TLS are indicated, and the MhqR operator is marked with *arrows*. **(B)** All 9–9 bp MhqR operator sites in front of genes of the MhqR regulons of *S. aureus* and *B. subtilis* (69) were aligned (denoted by *gray letters*), and the MhqR consensus sequence is indicated. TLS, translation start site; TSS, transcription start site.

Inspection of the *mhqRED* promoter region identified a 9–9 bp imperfect inverted repeat with the sequence TATCTCGAA-aTCGAaATA in position −6 to +12 relative to the TSS +1 (Fig. 5). The inverted repeat overlapping with the TSS was termed as MhqR operator based on its conservation with the MhqR operator upstream of *azoR2*, *mhqNOP*, *mhqED*, and *mhqA* in *B. subtilis* (69). To analyze the specific binding of MhqR to the MhqR operator, we exchanged two nucleotides in each half of the inverted repeat (m1: T to G and G to T; m2: C to A and A to G) and analyzed the DNA binding activity of MhqR to these mutated promoter probes (Fig. 4C). MhqR was unable to bind to the mutated inverted repeats m1 and m2 *in vitro*. In addition, no band shift was observed in the reaction of MhqR with the nonspecific *trxA* DNA probe, further supporting the specific binding of MhqR to the identified operator sequence (Fig. 4C).

Next, we investigated DNA binding and quinone-sensing of MhqR and MhqRC95A proteins. The MhqRC95A protein was able to bind with slightly decreased affinity to the *mhqRED* promoter probe compared with MhqR (Fig. 4C). Based on the EMSA results, the dissociation constants (*K*_d_) were calculated as 7.38 and 14.25 n*M* for MhqR and MhqRC95A mutant proteins, respectively. Treatment with increasing concentrations of MHQ resulted in complete dissociation of the MhqR and MhqRC95A proteins from the *mhqRED* promoter probe with 16–18 μ*M* MHQ, respectively (Fig. 4D). The addition of dithiothreitol (DTT) to the reaction of quinone-treated MhqR did not restore the DNA binding ability of MhqR, supporting that MhqR inactivation by quinones is not caused by a reversible thiol-switch (Fig. 4E). Thus, the nonconserved Cys95 of MhqR is not required for DNA binding and redox sensing of quinones *in vitro*, confirming our *in vivo* Northern blot results. This indicates that inactivation of the MhqR repressor by quinones does not involve a thiol-based mechanism. We speculate that MHQ binds to a specific ligand binding pocket in MhqR as revealed for other ligand binding MarR-type regulators (24), leading to its inactivation and derepression of the *mhqRED* operon.

Since no crystal structure of MhqR is available, the structure of *S. aureus* MhqR was modeled based on the template of the crystal structure of the MarR-family regulator ST1710 from *Sulfolobus tokodaii* (3GFI) using SWISS MODEL (6, 38) (Supplementary Fig. S5). MhqR of *S. aureus* shares 18.2% sequence identity with ST1710. The crystal structure of the ST1710 dimer was resolved in complex with its promoter DNA and with its ligand sodium salicylate, which is a common inhibitor of MarR proteins (38) (Supplementary Fig. S5A).

Similar to other MarR-type transcription factors, each subunit of the MhqR dimer is composed of six α-helices and two β-sheets, arranged as α1–α2–α3–α4–β1–β2–α5–α6 (Supplementary Fig. S5B). The α1, α5, and α6 helices form the dimer interface of the two MhqR subunits, and the DNA binding domain is composed of the α2, α3, α4 helices and the β1, β2 wing, known as winged helix-turn-helix (wHTH) DNA binding motif (16, 24). In the ST1710 structure complexed with salicylate, the ligand was coordinated by Y37 and Y111 of one subunit and A16, K17, and R20 of the opposing subunit of the dimer. This ligand binding pocket is located at the interface between the dimerization domains and the wHTH motif as described for other MarR-type regulators (24, 38). However, none of the salicylate coordinating tyrosine, lysine, or arginine residues of ST1710 is conserved in MhqR (Supplementary Fig. S5B). Thus, the mechanism of quinone binding in MhqR and the resulting conformational changes remain to be elucidated.

### The MhqR regulon confers resistance to MHQ and quinone-like antimicrobials in *S. aureus*

**FIG. 6.**
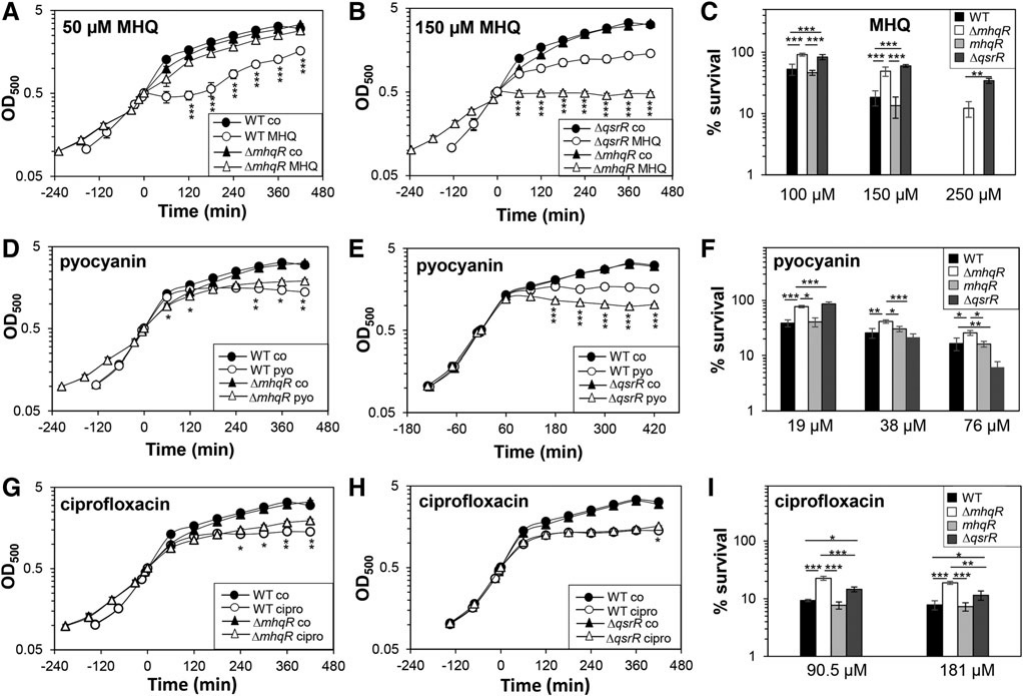
**The MhqR and QsrR regulons confer resistance to MHQ and the antimicrobials pyocyanin and ciprofloxacin. (A**, **B**, **D**, **E**, **G**, and **H)** For the growth curves, *S. aureus* COL wild type, *mhqR* and *qsrR* mutants, as well as the *mhqR* complemented strain (*mhqR*) were grown in RPMI until an OD_500_ of 0.5 and treated with 50 and 150 μ*M* MHQ, 76 μ*M* pyocyanin, and 90.5 μ*M* ciprofloxacin. **(C**, **F**, and **I)** Survival assays were performed by treatment with sublethal and lethal doses and plating 100 μL of serial dilutions onto LB agar plates after 4 h of stress exposure. The survival rates of CFUs for the treated samples were calculated relative to the control, which was set to 100%. The *mhqR* and *qsrR* mutants are significantly more resistant to MHQ, pyocyanin, and ciprofloxacin, which could be restored to wild-type levels in the *mhqR* complemented strain. The results are from four biological replicates. Error bars represent the standard deviation. *p < 0.05; **p < 0.01; ***p < 0.001. CFU, colony-forming unit; LB, Luria–Bertani.

**FIG. 7.**
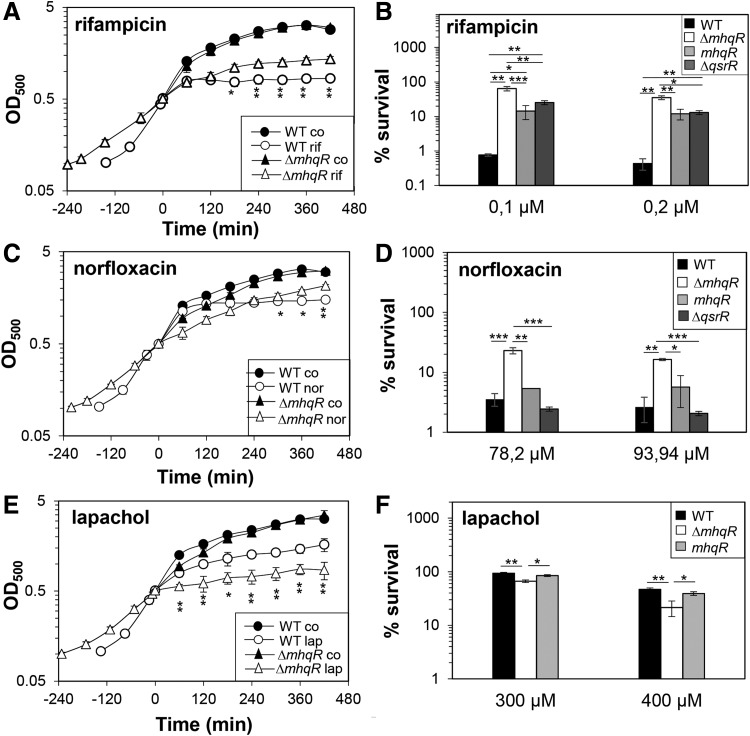
**The *mhqR* mutant is resistant to rifampicin and norfloxacin but impaired in survival after lapachol stress. (A**, **C**, **E)** For the growth curves, *S. aureus* COL wild type, the *mhqR* and *qsrR* mutants, and the *mhqR* complemented strain (*mhqR*) were grown in RPMI until an OD_500_ of 0.5 and treated with 0.05 μ*M* rifampicin, 62.6 μ*M* norfloxacin, and 300 μ*M* lapachol. **(B**, **D**, **F)** Survival assays were performed by treatment with sublethal and lethal doses and plating 100 μL of serial dilutions onto LB agar plates after 4 h of stress exposure. The survival rates of CFUs for the treated samples were calculated relative to the control, which was set to 100%. The *mhqR* mutant is significantly more resistant to rifampicin and norfloxacin, which could be restored in the *mhqR* complemented strain back to wild-type level. However, the *mhqR* mutant is significantly more susceptible to the naphthoquinone lapachol than the wild type. The results are from four biological replicates. Error bars represent the standard deviation. *p < 0.05; **p < 0.01; ***p < 0.001.

Next, we were interested whether the MhqR regulon is involved in quinone and antimicrobial resistance mechanisms. The growth and survival phenotypes of the *mhqR* mutant were analyzed under MHQ stress and after treatment with different antimicrobial compounds, including pyocyanin, ciprofloxacin, norfloxacin, rifampicin, and lapachol (Figs. 6 and 7). The *mhqR* mutant showed high resistance to 50 μ*M* MHQ and was not inhibited in growth compared with the wild type and *mhqR* complemented strain (Fig. 6A and Supplementary Fig. S6A). In addition, the *mhqR* mutant displayed two- to threefold increased survival in killing assays with lethal doses of 100–250 μ*M* MHQ (Fig. 6C).

Treatment of the *mhqR* mutant with the antimicrobials pyocyanin, ciprofloxacin, norfloxacin, and rifampicin resulted in slightly improved growth at sublethal doses and significantly enhanced survival in killing assays with lethal concentrations of the antimicrobial compounds (Figs. 6D–I and 7A–D). These antibiotic resistant phenotypes of the *mhqR* mutant could be restored back to wild-type level in the *mhqR* complemented strain (Supplementary Fig. S6B–E). However, the *mhqR* mutant was significantly impaired in growth and survival after treatment with the 1,4-naphthoquinone lapachol (Fig. 7E, F). These results indicate that the MhqR regulon protects *S. aureus* against benzoquinones, and many other antimicrobials that contain quinone-like structures, but not against naphthoquinones.

### The MhqR and QsrR regulons contribute independently to quinone and antimicrobial resistance

Apart from MhqR, the MarR/DUF24-type regulator QsrR was shown to mediate resistance to quinones and pyocyanin in *S. aureus* (33, 56). Thus, we compared the growth and survival phenotypes of the *mhqR* and *qsrR* mutants in response to MHQ, ciprofloxacin, norfloxacin, rifampicin, and pyocyanin (Figs. 6 and 7). The MhqR and QsrR regulons conferred significant resistance to MHQ, rifampicin, and the fluoroquinolone ciprofloxacin, but not to the same extent. The *qsrR* mutant was able to grow even with lethal doses of 150 μ*M* MHQ, which resulted in growth inhibition of the *mhqR* mutant (Fig. 6B). In survival assays, both mutants exhibited the same level of approximately two- to threefold increased resistance toward MHQ relative to the parent (Fig. 6C). Thus, the QsrR regulon conferred higher resistance to quinones than the *mhqR* mutant.

In contrast, the *mhqR* mutant showed higher ciprofloxacin resistance in growth assays and improved survival under ciprofloxacin, norfloxacin, and rifampicin treatment compared with the *qsrR* mutant (Figs. 6G–I and 7A–D).The MhqR and QsrR regulons contributed to a significant protection under low doses of 19 μ*M* pyocyanin (Fig. 6D–F). However, only the MhqR regulon protected against high pyocyanin concentrations (38–76 μ*M*) in killing assays. In contrast, the *qsrR* mutant was significantly more susceptible than the wild type at higher pyocyanin doses (Fig. 6E, F). These results point to independent roles of MhqR and QsrR as players in the quinone stress response. While the QsrR regulon mediates higher resistance to quinones, the MhqR regulon functions in resistance mechanisms against quinone-derived antimicrobials.

### The *mhqR* mutant shows differential susceptibilities to killing by murine macrophage *in vivo* and under oxidative stress *in vitro*

**FIG. 8.**
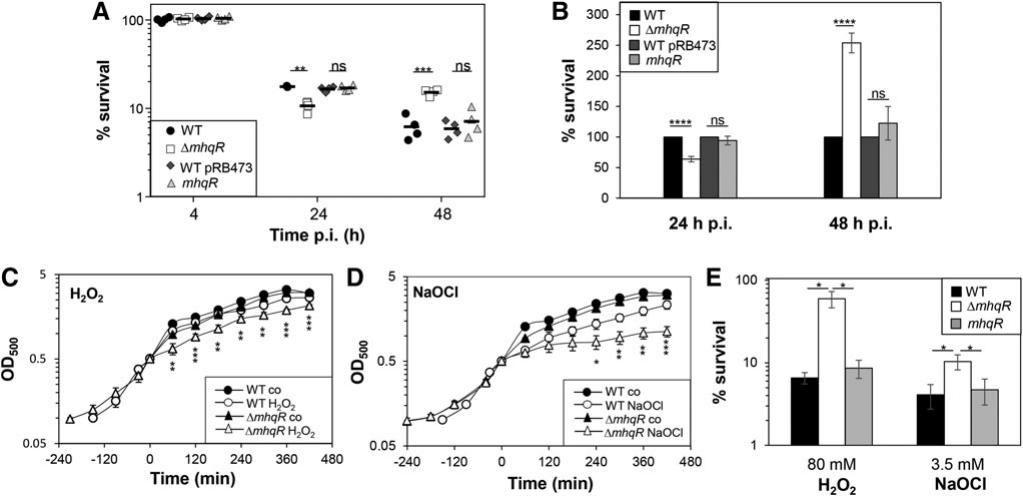
**The *mhqR* mutant is impaired in survival inside J-774.1 murine macrophages after 24 h and growth sensitive under sublethal ROS and NaOCl but resistant to long-term infections and toxic ROS and NaOCl. (A**, **B)** The survival of *S. aureus* strains was analyzed 2, 4, 24, and 48 h postinfection (p.i.) of the murine macrophage cell line J-774A.1 and the CFUs were determined. **(A)** The percentages in survival of the wild type (WT), WT pRB473, *mhqR* deletion mutant, and the *mhqR* complemented strain (*mhqR*) were calculated, and the survival at the 2 h time point was set to 100%. **(B)** The average percentage in survival was calculated for each mutant and complemented strains in relation to the WT or WT pRB473, which was set to 100%. Results of four biological replicates are presented as *scatter dots* in **(A)** and mean values **(B)**. **(C**, **D)** For growth curves, *S. aureus* COL wild type, the *mhqR* deletion mutant, and the *mhqR* complemented strain were grown in RPMI until an OD_500_ of 0.5 and treated with sublethal 10 m*M* H_2_O_2_ and 1.5 m*M* NaOCl. **(E)** Survival assays were performed by treatment with lethal 80 m*M* H_2_O_2_ and 3.5 m*M* NaOCl and plating serial dilutions onto LB agar plates after 4 h of stress exposure. The survival rates of CFUs for the treated samples were calculated relative to the control, which was set to 100%. The results are from three biological replicates. Error bars represent the standard deviation. ns, *p* > 0.05; *p < 0.05; **p < 0.01; ***p < 0.001; ****p ≤ 0.0001. H_2_O_2_, hydrogen peroxide; ROS, reactive oxygen species.

To analyze the role of the MhqR regulon under infection conditions, we determined the survival of the *mhqR* mutant in phagocytosis assays using the murine macrophage cell line J-774A.1, as previously described (44) (Fig. 8A, B). The colony-forming units (CFUs) of intracellular *S. aureus* were determined 2, 4, 24, and 48 h postinfection. At 24 h postinfection, the number of viable bacteria decreased to ∼20% for the wild type and 10% for the *mhqR* mutant (Fig. 8A). Thus, the *mhqR* mutant showed a 50% reduced survival rate compared with the wild type. This sensitive survival phenotype of the *mhqR* mutant could be restored to >90% in the *mhqR* complemented strain (Fig. 8B). Interestingly, 48 h postinfection, the number of surviving bacteria increased to ∼20% for the *mhqR* mutant and decreased to 6% for the wild type and *mhqR* complemented strain (Fig. 8A, B). Thus, the intramacrophage survival of the *mhqR* mutant was 2.5-fold higher compared with the wild type after 48 h of infections (Fig. 8B). This indicates that the *mhqR* mutation sensitizes *S. aureus* during early stages of macrophage infections, whereas improved survival of the *mhqR* mutant is acquired during long-term infection inside macrophages.

Transcriptome analysis revealed that the peroxide-specific PerR regulon was downregulated in the *mhqR* mutant under control and MHQ stress (Fig. 2 and Supplementary Fig. S2; Supplementary Table S2). Thus, we investigated the ROS susceptibility of the *mhqR* mutant *in vitro*. Growth phenotype analyses revealed an increased susceptibility of the *mhqR* deletion mutant under sublethal 1.5 m*M* NaOCl and 10 m*M* hydrogen peroxide (H_2_O_2_) stress (Fig. 8C, D). However, the *mhqR* mutant showed an improved survival upon lethal NaOCl and H_2_O_2_ stress compared with the wild type (Fig. 8E). The genetically encoded Brx-roGFP2 biosensor was applied to measure the changes in the BSH redox potential in the *mhqR* mutant during the growth and under H_2_O_2_ stress (Supplementary Fig. S7). The basal level oxidation of the Brx-roGFP2 was similar between the wild type and the *mhqR* mutant. However, the *mhqR* mutant showed a slightly higher oxidation increase and delayed recovery of the BSH redox potential compared with the wild type. Altogether, these results indicate that the *mhqR* mutant is sensitive in growth to sublethal ROS and to the host immune defense during the first 24 h of macrophage infections. However, under long-term infection conditions (48 h) and lethal ROS concentrations, the MhqR regulon is an important defense mechanism and required for *S. aureus* survival, providing an attractive drug target.

### The *mhqR* mutant shows enhanced respiratory chain activity and increased ATP levels

**FIG. 9.**
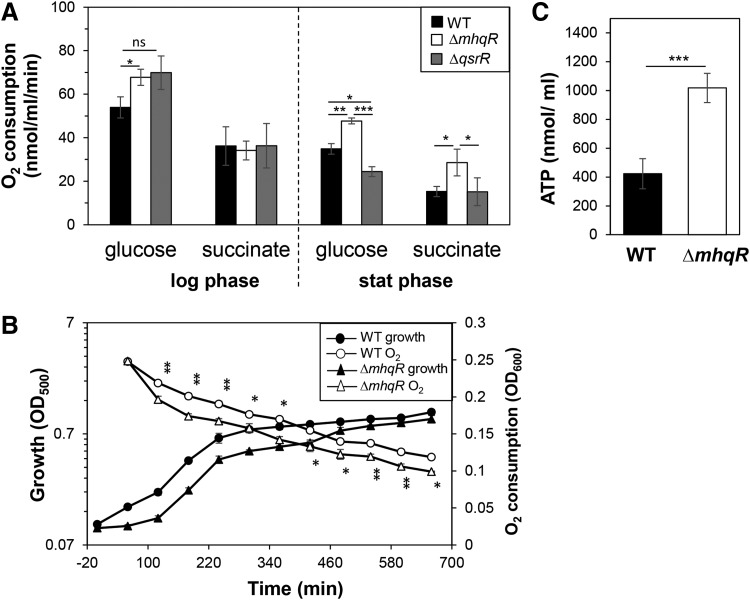
**The *mhqR* mutant shows a higher respiratory chain activity and increased ATP level. (A)** Oxygen consumption rates of the wild type and *mhqR* and *qsrR* mutants were determined during the exponential growth and stationary phases with glucose or succinate as electron donor using a Clark-type electrode. The results are presented as average values of three biological replicates with standard deviations. **(B)** To measure oxygen consumption under microaerophilic conditions, discoloration of methylene blue was measured as absorbance change at OD_600_ together with the OD_500_ as bacterial growth. **(C)** The ATP levels of the wild type and the *mhqR* mutant were determined during the exponential growth phrase with the ATP Bioluminescence Assay Kit CLS II (Sigma–Aldrich) according to the manufacturer's instructions. The results are from four biological replicates. Error bars represent the standard deviation. ns, *p* > 0.05; *p < 0.05; **p < 0.01; ***p < 0.001.

Quinones, such as menaquinone, are important electron carriers of the respiratory chain in *S. aureus*. Previous studies have shown that the quinone-sensing QsrR repressor responds also to menadione, the precursor of menaquinone in *S. aureus* (33). Thus, we investigated whether the upregulation of quinone degradation enzymes MhqD and MhqE in the *mhqR* mutant affects the electron transport to reduce molecular oxygen in the respiratory chain. Oxygen consumption rates were measured using a Clark-type electrode for the *mhqR* and *qsrR* mutants during the exponential growth and stationary phases with 1 m*M* glucose or 100 m*M* succinate as electron donors (Fig. 9A).

During the exponential growth phase, all strains showed high oxygen consumption rates of 55–70 nmol/mL/min with glucose as electron donor. The *mhqR* mutant had a significantly increased oxygen consumption rate with glucose compared with the wild type, but no differences were observed with succinate. During the stationary phase, the oxygen consumption rate of the wild type was ∼35 nmol/mL/min with glucose, significantly increased in the *mhqR* mutant (48 nmol/mL/min), but decreased in the *qsrR* mutant (Fig. 9A). Similarly, stationary phase *mhqR* mutant cells showed higher oxygen reduction rates with succinate (28 nmol/mL/min). These results of the higher respiratory chain activity in the *mhqR* mutant were also confirmed under microaerophilic conditions with methylene blue as indicator of oxygen consumption (Fig. 9B).

Due to the increased electron transport, elevated ATP levels could be determined in the *mhqR* mutant compared with the wild type (Fig. 9C). Thus, we speculate that quinones are more reduced in the *mhqR* mutant leading to an increased electron transport and higher ATP levels, which is supported by reduced expression of oxidative stress-specific genes in the transcriptome of the *mhqR* mutant.

## Discussion

In this study, we characterized the novel quinone-sensing MhqR repressor of *S. aureus* as an important component of the global response of *S. aureus* to quinones and antimicrobials. Transcriptome analysis in response to MHQ revealed the global signature of a thiol-specific oxidative and electrophile stress response, which is evident by the induction of the PerR, QsrR, MhqR, CtsR, and HrcA regulons. In addition, quinones caused a metal, sulfide, and cell wall stress response by upregulation of the Fur, CsoR, CstR, and GraRS regulons. This transcriptome profile overlaps strongly with the response to quinones in *B. subtilis* as shown by the inductions of the PerR, Spx, YodB, MhqR, HrcA, and CtsR regulons (1, 29, 42, 55, 68, 69).

**FIG. 10.**
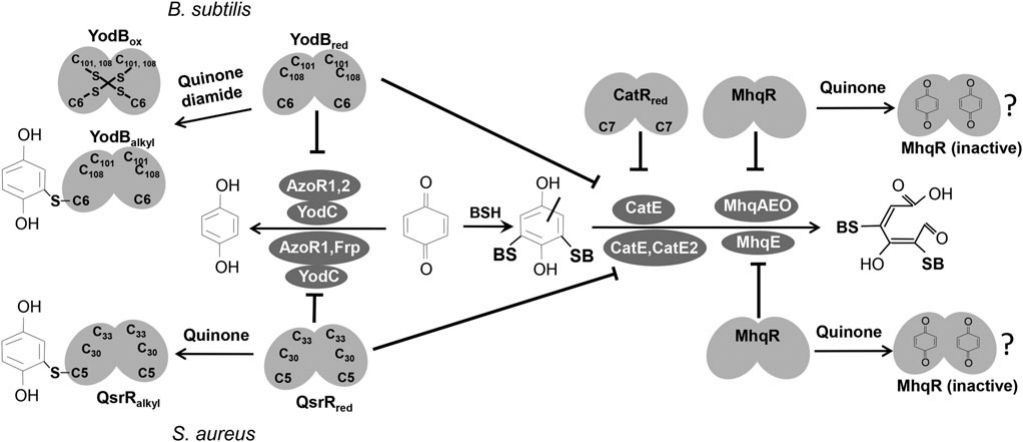
**The roles of the quinone-sensing MhqR, CatR, and YodB/QsrR regulons in *B. subtilis* and *S. aureus***. Exposure of *B. subtilis* and *S. aureus* to quinones induces the quinone detoxification regulons controlled by the homologous MarR-type repressors MhqR, YodB/QsrR, and CatR. The redox-sensing MarR/DUF24-family repressors YodB and CatR of *B. subtilis* are inactivated by intersubunit disulfide formation *in vivo* that involves the conserved Cys6 or Cys7 (1, 2, 12, 13, 69). The YodB and QsrR repressor mutant proteins with single Cys6 and Cys5 sense quinones also by thiol-*S*-alkylation *in vitro* (33, 41). The MhqR repressors might be inactivated by direct binding of quinones to a specific pocket. The MhqR and YodB/QsrR regulon include homologous quinone reductases, nitroreductases, and dioxygenases for quinone and diamide detoxification. The thiol-dependent dioxygenases MhqA, MhqE, CatE, and MhqO of *B. subtilis* and their respective homologs CatE, MhqE, and CatE2 of *S. aureus* (Supplementary Fig. S3B) are involved in ring cleavage of quinone-*S*-adducts. The quinone reductases AzoR1 and AzoR2 of *B. subtilis* and AzoR1 and Frp of *S. aureus* and the nitroreductases YodC and MhqN of *B. subtilis* and YodC of *S. aureus* catalyze the reduction of quinones to redox stable hydroquinones.

The MhqR and QsrR regulons represent the quinone stress signature in *S. aureus*. The QsrR regulon includes genes encoding ring-cleavage dioxygenases (*catE*, *catE2*), quinone reductases (*azoR1*, *frp*), and nitroreductases (*yodC*) (33). The MhqR regulon consists only of the *mhqRED* operon in *S. aureus* (Fig. 10). MhqD is annotated as phospholipase/carboxylesterase of the widespread alpha/beta fold hydrolase family (59). These enzymes cleave carboxylate esters to acids and alcohols and might be involved in the catabolism of quinone compounds. MhqE encodes a ring-cleavage dioxygenase in *S. aureus*. Thus, paralogous ring-cleavage dioxygenases and the nitro- and quinone reductases confer additive resistance to MHQ in *S. aureus*. Homologous dioxygenases (CatE, MhqA, MhqE, MhqO), quinone, and nitroreductases (AzoR1, AzoR2, YodC) have been shown to function in detoxification of exogenous quinones and catecholic compounds (Fig. 10) (12, 13, 42, 55, 68, 69), as well as the endogenous catecholate siderophore bacillibactin in *B. subtilis* (65). Thus, the QsrR and MhqR regulons have a similar composition of detoxification genes in both bacteria and confer resistance to quinones.

The catechol-2,3-dioxygenases CatE of *B. subtilis* was previously shown to cleave catechol to produce 2-hydroxymuconic semialdehyde (55, 68), whereas the dioxygenase MhqE of the MhqR regulon shares strong homology to hydroquinone-type 1,2-dioxygenase LinE of *Sphingomonas paucimobilis* that is involved in degradation of the xenobiotic insecticide hexachlorocyclohexane (51). Catechol-2,3-dioxygenases are iron-containing enzymes (51), and CatE was shown to respond also to iron limitation in *B. subtilis* through control by the Fur repressor (65). Thus, the *S. aureus* dioxygenases could be also involved in the decomposition of siderophores. *S. aureus* utilizes carboxylate siderophores staphyloferrin A and B but can also import xenosiderophores of other bacteria (25). However, the *S. aureus mhqR* and *qsrR* mutants showed no growth and survival phenotype upon treatment with the iron-scavenger 2,2′-dipyridyl compared with the wild type, indicating no function under iron limitation (Supplementary Fig. S8). More detailed studies are required to define the precise functions of the many detoxification enzymes of the MhqR and QsrR regulons in *S. aureus*.

MhqR belongs to the widespread MarR family of transcriptional regulators harboring wHTH DNA binding motifs that bind to 16–20 bp (pseudo) palindromic double-stranded DNA in adjacent major grooves (16). In previous studies, we identified a conserved 9–9 bp inverted repeat sequence as MhqR operator site for *B. subtilis* MhqR (69). This palindromic operator sequence was conserved in the *S. aureus mhqRED* upstream promoter region.

DNA binding assays revealed specific binding of MhqR to its operator with a high affinity (*K*_d_ = 7.38 n*M*). Comparative studies have shown that the dissociation constants vary across MarR type regulators (37, 75). However, the *K*_d_ value of MhqR is in the range of other MarR-type regulators, such as OhrR of *B. subtilis* (*K*_d_ = 5 n*M*) and MepR of *S. aureus* (*K*_d_ = 6.3 n*M*) (22, 36).

DNA binding assays further revealed that quinones lead to inhibition of the DNA binding activity of MhqR, which does not involve a thiol-based mechanism. Cys95 of MhqR is also not conserved in other MhqR homologs and dispensable for quinone regulation and DNA binding *in vivo* and *in vitro*. No involvement of the nonconserved Cys126 in quinone regulation was also shown for the *B. subtilis* MhqR protein (69). Thus, the regulatory mechanism of MhqR is different compared with redox-sensing MarR-type or Rrf2-family regulators, which sense directly redox-active compounds, such as ROS, hypochlorous acid (HOCl), or quinones by specific conserved redox-sensitive Cys residues (2, 29, 44). These redox-sensing regulators include YodB, CatR, HypR, and OhrR of *B. subtilis* and their homologs QsrR, SarZ, and MgrA of *S. aureus* (11, 12, 29, 33, 41, 42,44, 60, 61).

We speculate that the quinone-sensing mechanism of MhqR occurs *via* a direct binding of the quinone as ligand to a specific pocket. The DNA binding activity of many MarR-type regulators is altered by chemical ligands, such as phenolic or aromatic compounds (*e.g.*, salicylate, urate, protocatechuate, hydroxyphenylacetate, *p*-hydroxycinnamate-CoA) (24, 62, 75). Structural and biochemical studies of ligand-binding MarR-family proteins suggest a shared ligand-binding pocket between dimerization and DNA binding regions (16). This common ligand-binding pocket was also identified between the dimer interface and the wHTH motif in the structure of the MarR-type regulator ST1710 of *Sulfolobus tokodaii* in complex with its inhibitor salicylate (38).

The structure of the ST1710-salicylate complex was used as template to model the MhqR structure of *S. aureus* using SWISS-MODEL (Supplementary Fig. S5A). However, the salicylate contact residues Tyr37 and Tyr111 of one subunit and Ala16, Lys17, and Arg20 of the opposing subunit in the ST1710 dimer are not conserved in MhqR of *S. aureus*. Thus, the specific interactions of the putative ligand-binding pocket of MhqR with quinones and the resulting conformational changes in the wHTH motifs remain to be elucidated.

Apart from quinone resistance, the MhqR regulon also confers broad-spectrum antimicrobial resistance to quinone-like compounds in *S. aureus*, such as pyocyanin, ciprofloxacin, norfloxacin, and rifampicin. The fluoroquinolones ciprofloxacin and norfloxacin are priority class antibiotics to combat *S. aureus* infections, which act as DNA gyrase and topoisomerase inhibitors, causing superoxide anions and hydroxyl radicals through gyrase poisoning (19, 64). Pyocyanin is produced by *Pseudomonas aeruginosa*, a pathogen often co-isolated with *S. aureus* in cystic fibrosis patients. Pyocyanin blocks the electron transport chain by trapping electrons from NADH (26, 58). Mutations in *qsrR* have been previously selected as pyocyanin resistance mechanism (56). Rifampicin inhibits the RNA polymerase resulting in frequent *rpoB* mutations as resistance mechanism in *S. aureus* (74).

Our study revealed an involvement of the MhqR and QsrR regulons in antimicrobial resistance toward quinone-like antimicrobials in *S. aureus*. Similarly, the MarR-type regulators MarR of *Escherichia coli*, MgrA and MepR of *S. aureus*, as well as MexR in *P. aeruginosa* have been shown to confer resistance to multiple antibiotics by controlling efflux pumps (10, 14, 64, 70). We hypothesize that the MhqR- and QsrR-controlled dioxygenases and quinone reductases contribute to detoxification of the antimicrobial compounds with quinone structures as new resistance mechanism. There is also the controversial debate about the involvement of ROS generation in the killing mode of antibiotics. Thus, the antibiotic resistant phenotypes of the *mhqR* mutant could be connected to its ROS resistance in survival assays.

However, the MhqR regulon did not confer resistance to the naphthoquinone lapachol. Differences in the detoxification of benzoquinones and naphthoquinones have been described in *E. coli* (77). In *S. aureus*, flavohemoglobin has high substrate specificity for 2-hydroxy-1,4-naphthoquinones and might be more specific for naphthoquinone detoxification (53).

While the MhqR regulon plays an important role in antibiotic resistance, the *mhqR* mutant showed increased sensitivity at early time points of 24 h after macrophage infections and under sublethal ROS and HOCl exposure *in vitro*. We hypothesize that the lower basal transcription of PerR regulon genes in the *mhqR* mutant could contribute to the H_2_O_2_- and NaOCl-sensitive phenotypes as well as to decreased survival in infection assays. Surprisingly, the *mhqR* was delayed in growth after sublethal HOCl and H_2_O_2_ but acquired resistance to lethal doses of NaOCl and H_2_O_2_ in killing assays. In addition, at a later time point, 48 h postinfection of macrophages, the *mhqR* mutant showed a higher survival rate than the wild type.

It could be possible that the respiratory chain activity is decreased in the *S. aureus mhqR* mutant, as has been proposed in the *B. subtilis mhqR* mutant (34). Decreased respiratory chain activity was linked to lower ROS levels and facilitated growth of antibiotic resistant cell wall-deficient l-forms in *B. subtilis* (34). The *qsrR* mutant indeed showed decreased oxygen consumption with glucose, but only during the stationary phase. However, the *mhqR* mutant had a higher respiratory chain activity and increased ATP levels than the wild type. Thus, it might be possible that quinones are more reduced in the *mhqR* mutant, leading to enhanced electron transport. Our future analyses are directed to further investigate the functions and redox-sensing mechanisms of MhqR and QsrR in response to quinones and related antimicrobials.

## Experimental Procedures

### Bacterial strains, growth, and survival assays

Bacterial strains, plasmids, and primers are listed in Supplementary Tables S3 and S4. *E. coli* was cultivated in Luria–Bertani (LB) broth medium and *S. aureus* in RPMI medium. Survival assays were performed by plating 100 μL of serial dilutions of *S. aureus* onto LB agar plates and determination of CFUs. Statistical analysis was performed using Student's unpaired two-tailed *t*-test by the graph prism software. The compounds used for growth and survival assays (*e.g.*, MHQ, ciprofloxacin, norfloxacin, lapachol, pyocyanin, H_2_O_2_, NaOCl) were purchased from Sigma–Aldrich. NaOCl dissociates in aqueous solution to HOCl and hypochlorite (OCl^−^) (20). The concentration of HOCl was determined by absorbance measurements, as reported previously (76).

### Construction of the *S. aureus* COL *mhqR* and *qsrR* deletion mutants and the complemented *mhqR* and *mhqRC95A* mutant strains

The *S. aureus* COL *mhqR* (SACOL2531) and *qsrR* (SACOL2115) deletion mutants were constructed by allelic replacement *via* pMAD, as described previously (4, 44). The 500 bp upstream and downstream regions of *mhqR* and *qsrR* were each fused by overlap extension PCR and ligated into the *Bgl*II and *Sal*I sites of plasmid pMAD. The pMAD constructs were electroporated into *S. aureus* RN4220, transferred to *S. aureus* COL by phage transduction, and selected for plasmid excision leading to clean deletions of *mhqR* and *qsrR*, as described previously (44, 66).

The complemented *mhqR* and *mhqRC95A* mutant strains were constructed using the pRB473 plasmid, as described previously (44). The *mhqR* and *mhqRC95A* sequences were amplified from plasmids pET11b-*mhqR* and pET11b-*mhqRC95A*, digested with *Bam*HI and *Kpn*I, and inserted into pRB473 resulting in plasmids pRB473-*mhqR* and pRB473-*mhqRC95A* (Supplementary Table S3). The plasmids were introduced into the *mhqR* mutant *via* phage transduction, as described previously (44).

### RNA isolation, Northern blot analysis, RNA-seq transcriptomics, and bioinformatics

For RNA isolation, *S. aureus* COL was cultivated in RPMI medium and treated with 45 μ*M* MHQ, 300 μ*M* lapachol, 90.5 μ*M* ciprofloxacin, 76 μ*M* pyocyanin, 1 m*M* NaOCl, 0.5 m*M* methylglyoxal, 2 m*M* diamide, and 0.75 m*M* formaldehyde for 15 and 30 min, as described previously (73). Northern blot hybridizations were performed with the digoxigenin-labeled *mhqD*-specific antisense RNA probe synthesized *in vitro* using T7 RNA polymerase and the primer pairs SACOL2529-for/rev (Supplementary Table S4), as described previously (68, 73).

RNA-seq transcriptomics was performed using RNA of *S. aureus* COL and the *mhqR* deletion mutant isolated before and 30 min after 45 μ*M* MHQ, as described in previous studies (72). Differential gene expression analysis of three biological replicates was performed using DESeq2 (46) with ReadXplorer v2.2 (28) as described previously (72) using an adjusted *p*-value cutoff of ≤0.05 and a signal intensity ratio (*M*-value) cutoff of ≥0.6 or ≤−0.6 (fold-change of ±1.5).

The cDNAs enriched for primary 5′-transcripts were prepared according to the method described previously (63). cDNAs were sequenced paired end on an Illumina MiSeq System (San Diego, CA) using 75 bp read length. The R1 cDNA reads were mapped to the *S. aureus* USA300_TCH1516 genome (27) with bowtie2 v2.2.7 (40) using the default settings for single-end read mapping and visualized with Read Explorer v.2.2 (28). The whole transcriptome and 5′ enriched RNA-seq raw data files are available in the ArrayExpress database under E-MTAB-7074 and E-MTAB-7385.

### Cloning, expression, and purification of His-tagged MhqR and MhqRC95A mutant protein in *E. coli*

The *mhqR* gene (SACOL2531) was amplified from chromosomal DNA of *S. aureus* COL by PCR using primers SACOL2531-pET-for-NheI and SACOL2531-pET-rev-BamHI (Supplementary Table S4), digested with *Nhe*I and *Bam*HI, and inserted into plasmid pET11b (Novagen) to generate plasmid pET11b-*mhqR*. For the construction of *mhqRC95A* mutant, two first-round PCRs were performed using primer pairs SACOL2531-pET-for-NheI and SACOL2531-pET-C95A-Rev as well as primer pairs SACOL2531-pET-C95A-for and SACOL2531-pET-rev-BamHI (Supplementary Table S4). The two first-round PCR products were hybridized and amplified by a second round of PCR using primers SACOL2531-pET-for-NheI and SACOL2531-pET-rev-BamHI. The second-round PCR products were digested with *Nhe*I and *Bam*HI and inserted into plasmid pET11b to generate plasmid pET11b-*mhqRC95A*. For expression and purification of His-tagged MhqR and MhqRC95A proteins, *E. coli* BL21(DE3) p*lysS* was used with the plasmids pET11b-*mhqR* and pET11b-*mhqRC95A*, as described previously (44). Cultivation of the *E. coli* expression strains was performed in 1 L LB medium until the exponential growth phase at OD_600_ of 0.8, followed by the addition of 1 m*M* isopropyl-β-d-thiogalactopyranoside for 5 h at 30°C. Recombinant His-tagged MhqR and the MhqRC95A mutant proteins were purified, as described previously (44).

### EMSAs of MhqR and MhqRC95A proteins

For EMSAs, the DNA fragment containing the *mhqR* upstream region was amplified by PCR with the primer set emsa2531-for and emsa2531-rev (Supplementary Table S4). The DNA-binding reactions were performed with 15 ng/μL PCR product and purified His-MhqR and His-MhqRC95A proteins for 45 min, as described previously (44). MHQ was added to the DNA-MhqR-complex for 30 min to observe the dissociation of MhqR from the DNA. To analyze the reversibility of inhibition of MhqR by quinones, DTT was added 30 min after MHQ addition to the MhqR-DNA reaction. Thus, MHQ and DTT were added subsequently to the DNA-MhqR-complex for each 30 min. EMSAs were carried out as described previously (44).

### Brx-roGFP2 biosensor measurements

*S. aureus* COL and *mhqR* mutant strains with the Brx-roGFP2 biosensor plasmids were cultivated in LB and used for measurements of the biosensor oxidation degree along the growth curves and after injection of H_2_O_2_, as described previously (45). Fully reduced and oxidized controls were treated with 10 m*M* DTT and 5 m*M* diamide or 20 m*M* cumene hydroperoxide, respectively. Brx-roGFP2 biosensor fluorescence emission was measured at 510 nm after excitation at 405 and 488 nm using the CLARIOstar Microplate Reader (BMG Labtech), as described previously (45).

### Macrophage infection assays

The infection assays were performed using the murine macrophage cell line J-774A.1, as described previously (44). Intracellular survival of phagocytosed *S. aureus* was measured after 2, 4, 24, and 48 h postinfection by determination of CFUs, as described previously (44).

### Determination of oxygen consumption rates

The oxygen consumption rates of *S. aureus* strains were determined with a Clark-type electrode (Oxygraph; Hansatech) at 25°C according to a modified protocol, as described previously (50, 78). For determination of the respiratory chain activity during the exponential growth and stationary phases, cells were grown in tryptic soy broth medium to an OD_600_ of 0.6 and for 24 h. Cells were harvested by centrifugation, washed in 33 m*M* potassium phosphate buffer (pH 7.0), and adjusted to an OD_578_ of 5. Oxygen consumption was measured upon addition of 100 m*M* disodium succinate or 1 m*M* glucose as electron donors in three bioreplicates. Measurements were corrected for basal oxygen consumption without electron donors.

In addition, colorimetric determination of the oxygen consumption rates was performed by discoloration of methylene blue. Methylene blue was added at a final concentration of 0.004 mg/mL to 40 mL of *S. aureus* cells that were cultivated under microaerophilic conditions. The discoloration of methylene blue was determined as absorbance change at OD_600_ together with the optical density of the culture at OD_500_.

### ATP measurements

The ATP levels of *S. aureus* strains were determined with the ATP Bioluminescence Assay Kit CLS II (Sigma–Aldrich) according to the manufacturer's instructions. Briefly, 1 mL of exponentially growing cells was harvested, resuspended in 100 μL dilution buffer, and disrupted by boiling in 900 μL of 100 m*M* Tris, 4 m*M* ethylenediaminetetraacetic acid, pH 7.75, for 2 min. After centrifugation of the lysate, 50 μL of the supernatant was incubated with 50 μL luciferase and the luminescence was measured using the CLARIOstar Microplate Reader (BMG Labtech). The values were corrected for the autoluminescence of the cells, and the ATP level was determined based on the ATP standard curve.

## Supplementary Material

Supplemental data

Supplemental data

Supplemental data

Supplemental data

Supplemental data

Supplemental data

Supplemental data

Supplemental data

Supplemental data

Supplemental data

Supplemental data

Supplemental data

## Abbreviations Used

BSH: bacillithiol
CFU: colony-forming unit
DTT: dithiothreitol
EMSA: electrophoretic mobility shift assay
H_2_O_2_: hydrogen peroxide
HOCl: hypochlorous acid
LB: Luria–Bertani
MHQ: methylhydroquinone
NaOCl: sodium hypochlorite
ROS: reactive oxygen species
TSS: transcription start site
wHTH: winged helix-turn-helix
