# Supplementary material for: The MarR-Type Repressor MhqR Confers Quinone and Antimicrobial Resistance in Staphylococcus aureus
Source: Antioxid Redox Signal. 2019 Oct 17;31(16):1235–52. doi: 10.1089/ars.2019.7750 (PMC6798810; doi:10.1089/ars.2019.7750)
Supplement: Supplemental data [file Supp_Fig1.pdf]

Figure S1

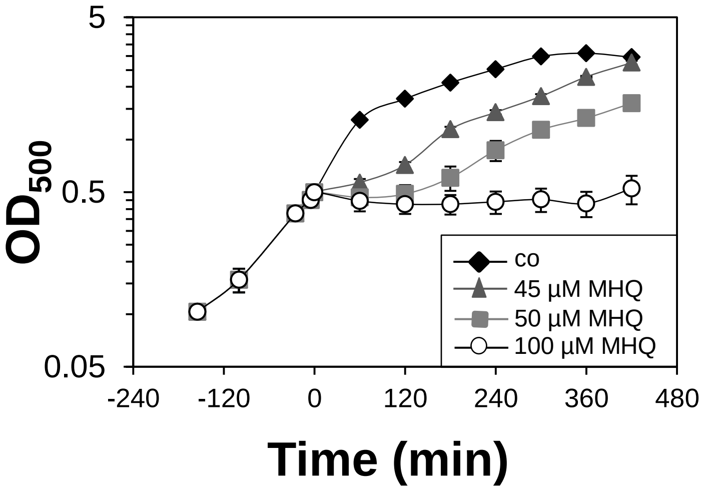

Fig. S1. Determination of the sub-lethal concentration of MHQ in *S. aureus* COL. For growth curves, *S. aureus* COL wild type was grown in RPMI medium until an OD_{500} of 0.5 and exposed to 45 µM, 50 µM and 100 µM MHQ. The results are from three biological replicates. Error bars represent the standard deviation.
