# Supplementary material for: The MarR-Type Repressor MhqR Confers Quinone and Antimicrobial Resistance in Staphylococcus aureus
Source: Antioxid Redox Signal. 2019 Oct 17;31(16):1235–52. doi: 10.1089/ars.2019.7750 (PMC6798810; doi:10.1089/ars.2019.7750)
Supplement: Supplemental data [file Supp_Table1.pdf]

TableS1: DESeq2 differential gene expression analysis of *S. aureus* COL wild type and the *mhqR* mutant before and after MHQ stress.

					WT MHQ vs control								WT MHQ/ WT co	ΔmhqR MHQ/ ΔmhqR co	ΔmhqR MHQ/ WT MHQ	ΔmhqR co/ WT co	WT MHQ/ WT co	ΔmhqR MHQ/ ΔmhqR co	ΔmhqR MHQ/ WT MHQ	ΔmhqR co/ WT co	WT MHQ/ WT co	ΔmhqR MHQ/ ΔmhqR co	ΔmhqR MHQ/ WT MHQ	ΔmhqR co/ WT co
Locus	Gene symbol	Operon	Regulon	Function	Base mean	A-value	M-value	Fold change	Standard error	Wald statistic	P-value	Adjusted P-value	M-value	M-value	M-value	M-value	Fold change	Fold change	Fold change	Fold change	Adj. P-value	Adj. P-value	Adj. P-value	Adj. P-value
SACOL2588	SACOL2588	SACOL2588 SACOL2589	TetR	hypothetical protein	48616,81	15,57	9,07	535,92	0,47	19,24	1,84E-82	6,41E-80	9,07	3,9	-3,58	0,18	535,92	14,93	0,08	1,13	6,41E-80	1,41E-15	2,36E-08	6,47E-01
SACOL2589	SACOL2589	SACOL2588 SACOL2589	TetR	hypothetical protein	39561,89	15,27	8,99	509,65	0,45	20,07	1,35E-89	7,52E-87	8,99	3,94	-4,38	-0,22	509,65	15,35	0,05	0,86	7,52E-87	6,87E-15	7,50E-22	6,21E-01
SACOL2533	catE2	catE2	QsrR	possible dioxygenase	72560,76	16,15	8,12	279,07	0,42	19,21	3,16E-82	9,79E-80	8,12	4,42	-2,41	1,38	279,07	21,41	0,19	2,6	9,79E-80	2,65E-244	1,97E-11	1,34E-16
SACOL0190	acpD	acpD	GraRS	azoreductase	66422,48	16,02	7,79	221,15	0,28	27,45	7,4E-166	2,05E-162	7,79	5,38	-1,31	1,05	221,15	41,64	0,4	2,07	2,05E-162	1,17E-303	6,50E-08	1,66E-07
SACOL0410	azoR1	catE SACOL0409 azoR1	QsrR	possible FMN reductase	14605,88	13,83	6,92	121,02	0,40	17,39	9,64E-68	1,92E-65	6,92	2,91	-3,16	0,78	121,02	7,52	0,11	1,72	1,92E-65	6,66E-56	1,39E-19	1,88E-03
SACOL2711	yceI	yceI	SigB	hypothetical protein	149736,44	17,19	6,64	100,06	0,37	17,98	2,85E-72	7,23E-70	6,64	4,5	-0,47	1,66	100,06	22,63	0,72	3,16	7,23E-70	2,43E-219	1,38E-01	1,33E-14
SACOL0409	SACOL0409	catE SACOL0409 azoR1	QsrR	possible alkanal monooxygenase (FMN-linked)	20380,88	14,31	6,58	95,92	0,41	16,18	6,91E-59	1,07E-56	6,58	2,33	-3,31	0,87	95,92	5,03	0,1	1,83	1,07E-56	1,68E-37	2,90E-21	3,42E-04
SACOL0408	catE	catE SACOL0409 azoR1	QsrR	possible lactoylglutathione lyase	8989,17	13,13	6,19	72,80	0,43	14,39	5,61E-47	4,73E-45	6,19	2,12	-3,4	0,59	72,80	4,35	0,09	1,51	4,73E-45	3,03E-29	1,97E-20	3,91E-02
SACOL2480	SACOL2480	SACOL2480	-	hypothetical protein	2987,95	11,54	6,18	72,37	0,48	12,91	3,92E-38	2,32E-36	6,18	2,35	-1,96	0,74	72,37	5,1	0,26	1,67	2,32E-36	2,86E-05	3,35E-03	4,56E-02
SACOL2529	mhqD	mhqR mhqD mhqE	MhqR	phospholipase/carboxylesterase MhqD	23839,00	14,54	6,07	67,27	0,53	11,52	1,07E-30	5,12E-29	6,07	-0,59	2,44	9,32	67,27	0,66	5,43	639,15	5,12E-29	9,23E-05	4,95E-08	0,00E+00
SACOL1678	SACOL1678	SACOL1678	MgrA	dehydrogenase	8264,77	13,01	6,04	65,84	0,25	23,85	9E-126	6,26E-123	6,04	3,61	-2,64	-0,28	65,84	12,21	0,16	0,82	6,26E-123	3,31E-64	4,31E-22	4,21E-01
SACOL2572	copA	copA copZ	CsoR	copper-translocating P-type ATPase	64298,10	15,97	6,00	63,78	0,30	19,85	1,03E-87	4,76E-85	6,00	3,34	-2,37	0,24	63,78	10,13	0,19	1,18	4,76E-85	1,97E-83	7,38E-16	2,62E-01
SACOL2573	copZ	copA copZ	CsoR	copper ion binding protein	21139,24	14,37	5,88	58,81	0,23	25,32	1,7E-141	1,61E-138	5,88	4,32	-1,66	-0,22	58,81	19,97	0,32	0,86	1,61E-138	2,88E-95	4,49E-08	3,48E-01
SACOL2530	mhqE	mhqR mhqD mhqE	MhqR	glyoxalase/dioxygenase MhqE	20409,98	14,32	5,82	56,55	0,54	10,84	2,14E-27	8,06E-26	5,82	-0,65	2,94	9,8	56,55	0,64	7,67	891,44	8,06E-26	3,55E-06	6,40E-11	0,00E+00
SACOL2534	frp	frp	QsrR	FMN reductase	10014,04	13,29	5,46	44,01	0,31	17,77	1,27E-70	2,72E-68	5,46	2,46	-2,19	0,74	44,01	5,5	0,22	1,67	2,72E-68	1,54E-44	4,24E-14	2,45E-04
SACOL0191	SACOL0191	SACOL0191	-	M23/M37 peptidase domain-containing protein	2237,14	11,13	5,29	39,26	0,37	14,43	3,47E-47	3,12E-45	5,29	3,06	-2,02	0,17	39,26	8,34	0,25	1,13	3,12E-45	8,78E-50	7,37E-10	6,65E-01
SACOL0414	efeO	efeOBU	Fur	iron (Fe2+)-binding lipoprotein	1093,34	10,09	5,22	37,21	0,33	15,59	8,16E-55	1,08E-52	5,22	2,44	-2,49	0,19	37,21	5,43	0,18	1,14	1,08E-52	1,19E-23	9,96E-19	7,11E-01
SACOL0063	cstA	cstAB sqr	CstR	rhodanese domain-containing protein	4327,31	12,08	5,14	35,26	0,57	9,00	2,18E-19	4,46E-18	5,14	1,99	-2,94	0,16	35,26	3,97	0,13	1,12	4,46E-18	1,13E-14	1,20E-09	7,31E-01
SACOL0415	efeB	efeOBU	Fur	iron (Fe2+)-dependent Dyp family peroxidase	1693,50	10,73	5,13	35,10	0,33	15,63	4,21E-55	5,86E-53	5,13	2,41	-2,29	0,36	35,10	5,31	0,2	1,28	5,86E-53	4,15E-35	1,40E-17	3,35E-01
SACOL2531	mhqR	mhqR mhqD mhqE	MhqR	MarR family transcriptional regulator MhqR	10654,34	13,38	5,13	34,98	0,53	9,67	4,11E-22	1,02E-20	5,13	-0,37	1,86	7,69	34,98	0,77	3,63	206,5	1,02E-20	1,06E-02	4,62E-05	0,00E+00
SACOL0597	hchA	hchA	SigB	ThiJ/PfpI family protein	54119,58	15,72	5,07	33,50	0,19	27,00	1,5E-160	2,09E-157	5,07	2,86	-2,23	-0,17	33,50	7,26	0,21	0,89	2,09E-157	3,74E-38	2,89E-13	4,64E-01
SACOL2414	SACOL2414	SACOL2414	-	hypothetical protein	2774,44	11,44	4,93	30,50	0,60	8,16	3,22E-16	5,10E-15	4,93	1,62	-2,22	0,4	30,50	3,07	0,21	1,32	5,10E-15	3,69E-04	1,89E-03	2,08E-01
SACOL0064	cstB	cstAB sqr	CstR	hydroxyacylglutathione hydrolase	6895,18	12,75	4,93	30,42	0,54	9,18	4,48E-20	9,73E-19	4,93	1,85	-2,57	0,53	30,42	3,61	0,17	1,44	9,73E-19	5,24E-28	1,59E-08	3,08E-02
SACOL0157	srpF	srpF	CymR	hypothetical protein	1036,44	10,02	4,84	28,67	0,43	11,32	9,97E-30	4,41E-28	4,84	1,28	-3,66	-0,12	28,67	2,43	0,08	0,92	4,41E-28	1,32E-06	6,42E-26	8,19E-01
SACOL0189	SACOL0189	SACOL0189	-	hypothetical protein	6312,62	12,62	4,79	27,70	0,34	14,14	2,06E-45	1,55E-43	4,79	3,84	-0,95	0,04	27,70	14,32	0,52	1,03	1,55E-43	8,64E-178	1,45E-03	8,99E-01
SACOL0412	SACOL0412	SACOL0412	-	hypothetical protein	796,76	9,64	4,69	25,82	0,35	13,30	2,28E-40	1,47E-38	4,69	2,51	-2,56	-0,35	25,82	5,7	0,17	0,78	1,47E-38	1,36E-22	3,32E-18	4,58E-01
SACOL2020	yodC	yodC	QsrR	possible nitroreductase	8825,16	13,11	4,68	25,55	0,24	19,78	4,02E-87	1,60E-84	4,68	3,47	-0,68	0,52	25,55	11,08	0,62	1,43	1,60E-84	1,90E-183	1,12E-03	3,10E-03
SACOL2478	fmrO	SACOL2477 fmrO dapF	GraRS	rRNA methyltransferase FmrO	380,18	8,57	4,64	24,97	0,63	7,32	2,51E-13	3,00E-12	4,64	1,88	-1,88	0,68	24,97	3,68	0,27	1,6	3,00E-12	1,89E-10	2,91E-04	1,44E-01
SACOL0047	SACOL0047	SACOL0046 SACOL0047	-	conserved hypothetical protein	3904,61	11,93	4,57	23,70	0,96	4,76	1,93E-06	8,72E-06	4,57	2,39	-2,98	-0,23	23,70	5,24	0,13	0,85	8,72E-06	4,95E-17	8,73E-05	6,67E-01
SACOL0416	efeU	efeOBU	Fur	OFeT family oxidase-dependent iron (Fe2+) transporter	2388,81	11,22	4,56	23,65	0,29	15,64	3,71E-55	5,43E-53	4,56	2,62	-2,03	-0,12	23,65	6,15	0,24	0,92	5,43E-53	1,11E-44	1,08E-13	7,32E-01
[illegible]	[illegible]	[illegible]	[illegible]	[illegible]	[illegible]	[illegible]	[illegible]	[illegible]	[illegible]	[illegible]	[illegible]	[illegible]	[illegible]	[illegible]	[illegible]	[illegible]	[illegible]	[illegible]	[illegible]	[illegible]	[illegible]	[illegible]	[illegible]	[illegible]

					WT MHQ vs control								WT MHQ/ WT co	ΔmhqR MHQ/ ΔmhqR co	ΔmhqR MHQ/ WT MHQ	ΔmhqR co/ WT co	WT MHQ/ WT co	ΔmhqR MHQ/ ΔmhqR co	ΔmhqR MHQ/ WT MHQ	ΔmhqR co/ WT co	WT MHQ/ WT co	ΔmhqR MHQ/ ΔmhqR co	ΔmhqR MHQ/ WT MHQ	ΔmhqR co/ WT co
Locus	Gene symbol	Operon	Regulon	Function	Base mean	A-value	M-value	Fold change	Standard error	Wald statistic	P-value	Adjusted P-value	M-value	M-value	M-value	M-value	Fold change	Fold change	Fold change	Fold change	Adj. P-value	Adj. P-value	Adj. P-value	Adj. P-value
SACOL1770	ugpQ2	ugpQ2	-	glycerophosphoryl diester phosphodiesterase	273,36	8,09	1,69	3,23	0,29	5,85	4,79E-09	3,34E-08	1,69	0,76	-1,13	-0,19	3,23	1,69	0,46	0,88	3,34E-08	1,23E-03	2,86E-06	6,31E-01
SACOL1034	SACOL1034	SACOL1034	-	lipoate-protein ligase A family protein	1341,91	10,39	1,69	3,22	0,27	6,16	7,1E-10	5,69E-09	1,69	1,15	-0,88	-0,33	3,22	2,22	0,54	0,8	5,69E-09	3,69E-10	7,71E-05	2,89E-01
SACOL1094	cydA	cydA cydB	GraRS	cytochrome d ubiquinol oxidase subunit I	1787,01	10,80	1,68	3,21	0,32	5,29	1,22E-07	6,68E-07	1,68	0,81	-0,87	-0,01	3,21	1,75	0,55	0,99	6,68E-07	4,35E-09	1,98E-03	9,85E-01
SACOL1138	sasJ	sasJ	-	LPXTG cell wall surface anchor protein	114,52	6,84	1,68	3,20	0,37	4,48	7,46E-06	2,99E-05	1,68	1,22	-0,71	-0,21	3,20	2,33	0,61	0,86	2,99E-05	1,81E-05	3,61E-03	6,95E-01
SACOL0981	leuA2	leuA2 SACOL0982 SACOL0983 SACOL0984	-	isopropylmalate synthase-related protein	43,95	5,46	1,68	3,20	0,56	2,98	0,002883	6,99E-03	1,68	-0,18	-1,08	0,53	3,20	0,88	0,47	1,44	6,99E-03	6,97E-01	1,94E-02	2,72E-01
SACOL0921	SACOL0921	SACOL0921 fabK	SigB	CBS domain protein	689,16	9,43	1,68	3,19	0,21	7,92	2,3E-15	3,35E-14	1,68	1,17	-0,34	0,13	3,19	2,25	0,79	1,09	3,35E-14	6,42E-12	8,13E-02	6,82E-01
SACOL0498	SACOL0498	SACOL0498 SACOL0499	-	hypothetical protein	2162,21	11,08	1,67	3,19	0,31	5,37	7,78E-08	4,45E-07	1,67	0,38	-0,96	0,32	3,19	1,3	0,51	1,25	4,45E-07	1,01E-02	5,38E-04	8,30E-02
SACOL0394	SACOL0394	SACOL0394 gcvH-L SACOL0396 sirTM lplA2	-	hypothetical protein	760,71	9,57	1,67	3,18	0,23	7,12	1,08E-12	1,20E-11	1,67	0,63	-0,73	0,28	3,18	1,55	0,6	1,21	1,20E-11	3,69E-04	1,07E-03	2,64E-01
SACOL0832	whiA	SACOL0830 SACOL0831 whiA	SigB	hypothetical protein	8563,89	13,06	1,67	3,18	0,27	6,27	3,62E-10	3,08E-09	1,67	0,33	-1,01	0,31	3,18	1,26	0,5	1,24	3,08E-09	1,37E-02	2,16E-05	4,90E-02
SACOL1781	isdH	isdH	Fur	cell wall surface anchored protein	425,55	8,73	1,67	3,17	0,29	5,68	1,31E-08	8,51E-08	1,67	0,76	-1,18	-0,28	3,17	1,69	0,44	0,82	8,51E-08	3,50E-04	1,22E-05	3,56E-01
SACOL0395	gcvH-L	SACOL0394 gcvH-L SACOL0396 sirTM lplA2	-	glycine cleavage system H protein	383,10	8,58	1,66	3,16	0,25	6,71	1,92E-11	1,88E-10	1,66	0,61	-0,93	0,1	3,16	1,53	0,52	1,07	1,88E-10	3,58E-03	7,75E-05	7,76E-01
SACOL0184	oppF_2	oppF_2	CymR	oligopeptide ABC transporter, ATP-binding protein	246,82	7,95	1,66	3,16	0,28	5,87	4,45E-09	3,12E-08	1,66	1,28	0,05	0,38	3,16	2,43	1,04	1,3	3,12E-08	4,43E-11	8,80E-01	2,10E-01
SACOL1369	rpmG2	rpmG1	-	50S ribosomal protein L33	17221,42	14,07	1,66	3,16	0,36	4,55	5,41E-06	2,22E-05	1,66	0,83	-0,61	0,21	3,16	1,78	0,66	1,16	2,22E-05	1,09E-05	9,82E-02	3,14E-01
SACOL1291	ribC	rbfA truB ribC	SigB	bifunctional riboflavin kinase/FAD synthase RibC	4700,14	12,20	1,66	3,15	0,31	5,40	6,55E-08	3,78E-07	1,66	0,41	-1,1	0,13	3,15	1,33	0,47	1,09	3,78E-07	2,19E-03	5,05E-05	5,03E-01
SACOL0173	ipdC	ipdC	CcpA	thiamine pyrophosphate TPP binding domain-containing protein	3079,11	11,59	1,64	3,13	0,35	4,69	2,69E-06	1,17E-05	1,64	0,89	-0,78	-0,03	3,13	1,85	0,58	0,98	1,17E-05	8,88E-10	1,35E-02	9,18E-01
SACOL2463	pepA2	pepA2	-	M42 family glutamyl aminopeptidase	5239,46	12,36	1,64	3,12	0,18	9,06	1,25E-19	2,61E-18	1,64	1,22	0,13	0,53	3,12	2,33	1,09	1,44	2,61E-18	9,71E-25	5,11E-01	1,06E-04
SACOL1550	graE	graE	-	AraC family transcriptional regulator	6063,72	12,57	1,63	3,09	0,30	5,37	7,93E-08	4,52E-07	1,63	-0,04	-1,61	0,04	3,09	0,97	0,33	1,03	4,52E-07	8,10E-01	2,49E-10	8,74E-01
SACOL2286	ureD	ureABCEFGD	GraRS	urease accessory protein UreD	718,77	9,49	1,63	3,09	0,29	5,66	1,48E-08	9,60E-08	1,63	-0,02	-1,84	-0,21	3,09	0,99	0,28	0,86	9,60E-08	9,14E-01	1,01E-12	4,38E-01
SACOL2535	ddh	ddh	MgrA	D-lactate dehydrogenase	17378,50	14,09	1,62	3,08	0,21	7,64	2,16E-14	2,80E-13	1,62	0,6	-0,8	0,19	3,08	1,52	0,57	1,14	2,80E-13	1,51E-04	2,00E-04	3,50E-01
[illegible]	[illegible]	[illegible]	[illegible]	[illegible]	[illegible]	[illegible]	[illegible]	[illegible]	[illegible]	[illegible]	[illegible]	[illegible]	[illegible]	[illegible]	[illegible]	[illegible]	[illegible]	[illegible]	[illegible]	[illegible]	[illegible]	[illegible]	[illegible]	[illegible]

					WT MHQ vs control								WT MHQ/ WT co	Δ*mhqR* MHQ/ Δ*mhqR* co	Δ*mhqR* MHQ/ WT MHQ	Δ*mhqR* co/ WT co	WT MHQ/ WT co	Δ*mhqR* MHQ/ Δ*mhqR* co	Δ*mhqR* MHQ/ WT MHQ	Δ*mhqR* co/ WT co	WT MHQ/ WT co	Δ*mhqR* MHQ/ Δ*mhqR* co	Δ*mhqR* MHQ/ WT MHQ	Δ*mhqR* co/ WT co
Locus	Gene symbol	Operon	Regulon	Function	Base mean	A-value	M-value	Fold change	Standard error	Wald statistic	P-value	Adjusted P-value	M-value	M-value	M-value	M-value	Fold change	Fold change	Fold change	Fold change	Adj. P-value	Adj. P-value	Adj. P-value	Adj. P-value
SACOL0089	SACOL0089	SACOL0089	SigB	myosin-cross-reactive antigen	220,44	7,78	0,83	1,78	0,36	2,31	0,020921	3,99E-02	0,83	0,8	0,89	0,86	1,78	1,74	1,85	1,82	3,99E-02	3,47E-04	6,94E-03	7,14E-04
SACOL0526	holB	holB SACOL0527 yabA	MgrA	DNA-directed DNA polymerase III delta' subunit	2106,71	11,04	0,83	1,78	0,28	2,98	0,002926	7,08E-03	0,83	0,68	-0,05	0,09	1,78	1,6	0,97	1,06	7,08E-03	7,16E-05	8,73E-01	7,91E-01
SACOL1129	ylbF	SACOL1128 ylbF	-	hypothetical protein	1174,63	10,20	0,83	1,77	0,25	3,36	0,000774	2,15E-03	0,83	-0,27	-0,75	0,32	1,77	0,83	0,59	1,25	2,15E-03	1,30E-01	6,36E-04	1,85E-01
SACOL2265	mobB	moaA mobA moaD moaE mobB moeA	MgrA	molybdopterin-guanine dinucleotide biosynthesis protein B	2114,96	11,05	0,82	1,77	0,22	3,80	0,000143	4,64E-04	0,82	0,01	-0,96	-0,15	1,77	1,01	0,51	0,9	4,64E-04	9,82E-01	2,19E-06	5,81E-01
SACOL2211	cbiO	truA SACOL2209 SACOL2210 SACOL2211	-	cobalt transporter ATP-binding subunit	820,84	9,68	0,82	1,76	0,27	3,08	0,002053	5,16E-03	0,82	0,78	-0,41	-0,37	1,76	1,72	0,75	0,77	5,16E-03	1,58E-06	1,23E-01	5,64E-02
SACOL2455	SACOL2455	-	-	hypothetical protein	199,79	7,64	0,82	1,76	0,31	2,59	0,009482	2,01E-02	0,82	-0,04	-0,93	-0,08	1,76	0,97	0,52	0,95	2,01E-02	8,88E-01	2,50E-04	8,74E-01
SACOL0548	SACOL0548	rplY pth mfd SACOL0548 mazG SACOL0550 divIC	AgrA	polysaccharide biosynthesis protein	3343,99	11,71	0,82	1,76	0,22	3,67	0,000239	7,35E-04	0,82	0,63	-0,43	-0,25	1,76	1,55	0,74	0,84	7,35E-04	2,41E-06	3,37E-02	1,75E-01
SACOL1253	ffh	smc ftsY SACOL1252 ffh	-	signal recognition particle protein	10598,31	13,37	0,82	1,76	0,22	3,65	0,000259	7,90E-04	0,82	0,36	-0,29	0,16	1,76	1,28	0,82	1,12	7,90E-04	2,68E-02	2,09E-01	5,06E-01
SACOL2555	cidR	cidR	-	LysR family transcriptional regulator	486,49	8,93	0,82	1,76	0,25	3,27	0,001087	2,90E-03	0,82	0,16	-0,24	0,38	1,76	1,12	0,85	1,3	2,90E-03	3,82E-01	3,03E-01	1,05E-01
SACOL0550	SACOL0550	rplY pth mfd SACOL0548 mazG SACOL0550 divIC	AgrA	S4 domain-containing protein	2060,93	11,01	0,81	1,76	0,21	3,89	9,9E-05	3,32E-04	0,81	0,52	-0,46	-0,16	1,76	1,43	0,73	0,9	3,32E-04	2,42E-03	1,89E-02	5,53E-01
SACOL0926	SACOL0926	SACOL0924 SACOL0925 SACOL0926	CodY	5'-nucleotidase	3088,88	11,59	0,81	1,75	0,19	4,22	2,45E-05	9,10E-05	0,81	0,36	-0,52	-0,08	1,75	1,28	0,7	0,95	9,10E-05	1,53E-02	6,70E-03	7,11E-01
SACOL2252	SACOL2252	SACOL2252 femX	GraRS	RND resistance-nodulation-cell division acriflavin:proton (H+) antiporter	7288,87	12,83	0,81	1,75	0,26	3,12	0,001813	4,61E-03	0,81	-0,11	-0,78	0,12	1,75	0,93	0,58	1,09	4,61E-03	4,80E-01	4,63E-04	6,02E-01
SACOL2539	srtA	srtA	MgrA	sortase	2774,73	11,44	0,81	1,75	0,34	2,36	0,018272	3,56E-02	0,81	-0,44	-0,72	0,5	1,75	0,74	0,61	1,41	3,56E-02	4,86E-03	2,05E-02	9,90E-03
SACOL0744	graA	graA cydC	Rex	ABC superfamily ATP binding cassette transporter, ABC protein	4261,05	12,06	0,80	1,75	0,19	4,14	3,5E-05	1,26E-04	0,80	0,26	-0,24	0,28	1,75	1,2	0,85	1,21	1,26E-04	1,50E-01	2,90E-01	2,19E-01
SACOL0717	graS	graXRS	GraRS	sensor histidine kinase	1262,95	10,30	0,80	1,74	0,23	3,46	0,000541	1,56E-03	0,80	-0,04	-0,62	0,21	1,74	0,97	0,65	1,16	1,56E-03	8,35E-01	2,45E-03	3,91E-01
SACOL1283	polC	polC	-	DNA polymerase III PolC	10755,77	13,39	0,80	1,74	0,23	3,50	0,000471	1,37E-03	0,80	0,48	-0,19	0,12	1,74	1,39	0,88	1,09	1,37E-03	7,16E-05	3,99E-01	5,18E-01
SACOL1811	SACOL1811	SACOL1810 SACOL1811	-	hypothetical protein	1032,63	10,01	0,80	1,74	0,20	4,08	4,58E-05	1,63E-04	0,80	0,38	-0,67	-0,25	1,74	1,3	0,63	0,84	1,63E-04	2,87E-02	1,93E-04	2,96E-01
SACOL1013	mgtE	SACOL1009 relA1 ppnK yjbO mgtE cpaA	MgrA	MgtE family magnesium (Mg2+)/cobalt (Co2+) transporter-E	2765,53	11,43	0,80	1,74	0,24	3,35	0,000821	2,26E-03	0,80	0,1	-0,61	0,07	1,74	1,07	0,66	1,05	2,26E-03	4,72E-01	3,94E-03	7,40E-01
SACOL0235	gatD	gatC gutB SACOL0234 gatD	-	oxidoreductase, zinc-binding dehydrogenase family	176,28	7,46	0,79	1,73	0,29	2,73	0,006303	1,39E-02	0,79	0,39	-0,46	-0,07	1,73	1,31	0,73	0,95	1,39E-02	1,24E-01	5,64E-02	8,80E-01
SACOL2708	mtsB	mtsA mtsB mtsC SACOL2710	GraRS	cobalt ABC transporter ATPase	182,98	7,52	0,79	1,73	0,27	2,88	0,004004	9,33E-03	0,79	0,66	-0,5	-0,35	1,73	1,58	0,71	0,78	9,33E-03	3,68E-03	3,76E-02	2,79E-01
SACOL1393	glcT	glcT SACOL1394 yrrI	-	transcription antiterminator	539,41	9,08	0,79	1,72	0,24	3,32	0,000887	2,41E-03	0,79	0,69	-0,12	-0,03	1,72	1,61	0,92	0,98	2,41E-03	3,71E-05	6,03E-01	9,40E-01
SACOL1091	ptsH	ptsH ptsI	-	phosphocarrier protein ptsH	11078,42	13,44	0,78	1,72	0,20	4,02	5,77E-05	2,03E-04	0,78	0,12	-0,51	0,14	1,72	1,09	0,7	1,1	2,03E-04	3,78E-01	6,10E-03	4,11E-01
SACOL0805	SACOL0805	SACOL0805	-	glycerate kinase	1448,10	10,50	0,78	1,71	0,32	2,41	0,015975	3,18E-02	0,78	-0,46	-1,51	-0,28	1,71	0,73	0,35	0,82	3,18E-02	6,37E-03	1,32E-07	2,18E-01
SACOL0762	SACOL0762	SACOL0762	-	hemolysin	8087,39	12,98	0,77	1,71	0,20	3,93	8,39E-05	2,85E-04	0,77	0,64	-0,03	0,08	1,71	1,56	0,98	1,06	2,85E-04	1,11E-07	8,85E-01	7,17E-01
SACOL2038	gcp	gcp rimI yeaZ yjeE	-	DNA-binding/iron metalloprotein/AP endonuclease	1731,24	10,76	0,77	1,70	0,26	3,00	0,002708	6,61E-03	0,77	0,43	-0,41	-0,08	1,70	1,35	0,75	0,95	6,61E-03	1,63E-03	8,14E-02	7,32E-01
SACOL1497	papS	dinG birA papS bshA	BSH	polynucleotide adenylyltransferase	2249,40	11,14	0,76	1,70	0,23	3,29	0,000986	2,67E-03	0,76	-0,33	-0,81	0,27	1,70	0,8	0,57	1,21	2,67E-03	1,36E-02	4,71E-05	1,29E-01
SACOL0013	SACOL0013	SACOL0013 SACOL0014 rplI dnaC	-	hypothetical protein	2997,42	11,55	0,76	1,69	0,25	3,03	0,002426	6,00E-03	0,76	0,49	-0,88	-0,6	1,69	1,4	0,54	0,66	6,00E-03	2,32E-04	5,02E-05	4,01E-04
SACOL1224	priA	coaBC priA	-	primosomal protein N`	5434,50	12,41	0,76	1,69	0,18	4,21	2,56E-05	9,44E-05	0,76	0,54	0	0,2	1,69	1,45	1	1,15	9,44E-05	5,29E-05	9,93E-01	2,97E-01
SACOL0915	sufD	sufCDSUB	PerR	FeS assembly protein SufD	17065,06	14,06	0,75	1,69	0,20	3,81	0,000142	4,60E-04	0,75	0,93	0,67	0,47	1,69	1,91	1,59	1,39	4,60E-04	1,36E-11	6,37E-05	9,93E-03
SACOL1053	menH	menF menD menH menB	MgrA	S33 family peptidase	3161,65	11,63	0,75	1,68	0,26	2,86	0,00422	9,77E-03	0,75	0,21	-0,1	0,43	1,68	1,16	0,93	1,35	9,77E-03	1,33E-01	7,36E-01	8,28E-03
SACOL0019	walR	walRKHI	GraRS	response regulator VicR	3439,48	11,75	0,75	1,68	0,24	3,16	0,001579	4,07E-03	0,75	0,24	-0,69	-0,18	1,68	1,18	0,62	0,88	4,07E-03	1,58E-01	1,06E-03	4,67E-01
[illegible]	[illegible]	[illegible]	[illegible]	[illegible]	[illegible]	[illegible]	[illegible]	[illegible]	[illegible]	[illegible]	[illegible]	[illegible]	[illegible]	[illegible]	[illegible]	[illegible]	[illegible]	[illegible]	[illegible]	[illegible]	[illegible]	[illegible]	[illegible]	[illegible]

Locus	Gene symbol	Operon	Regulon	Function	Base mean	A-value	M-value
SACOL1075	purC	purEKCSQLFMNHD	PurR	phosphoribosylaminoimidazolesuccinocarboxamide synthase	450,05	8,81	-0,95
SACOL2204	SACOL2204	clpA SACOL2204	-	hypothetical protein	1118,32	10,13	-0,95
SACOL0932	ghrB	SACOL0930 pdxp ghrB	-	glyoxylate reductase	2294,83	11,16	-0,95
SACOL1511	ubiE	hepT ubiE SACOL1512	-	ubiquinone/menaquinone biosynthesis methyltransferase	1614,01	10,66	-0,95
SACOL0239	tarF	tarF	-	TagF domain-containing protein	728,28	9,51	-0,95
SACOL1232	pknB	def2 fmt sun rlmN stp1 pknB	-	protein kinase	5730,77	12,48	-0,95
SACOL1968	hisR	hisR	-	hypothetical protein	1228,99	10,26	-0,96
SACOL2575	ywfG	ddh ywfG	CodY	N-succinyldiaminopimelate aminotransferase	598,50	9,23	-0,96
SACOL1255	rimM	rpsP rimM trmD rplS	-	16S rRNA processing protein RimM	708,88	9,47	-0,96
SACOL0666	fecD	btuF fecD SACOL0667 SACOL0668 SACOL0669 SACOL0670	-	iron (Fe3) ABC superfamily ATP binding cassette transporter, membrane protein	215,62	7,75	-0,96
SACOL2438	SACOL2438	SACOL2438	-	hypothetical protein	93,58	6,55	-0,96
SACOL1699	obgE	yajC tgt queA ruvB ruvA thrR obgE	-	GTPase ObgE	6142,21	12,58	-0,96
SACOL2267	moaC	moaC	-	molybdenum cofactor biosynthesis protein MoaC	1324,50	10,37	-0,96
SACOL1572	accB	ispA xseB xseA nusB SACOL1570 accC accB	-	acetyl-CoA carboxylase, biotin carboxyl carrier protein	1839,24	10,84	-0,96
SACOL0228	bglG	blgG SACOL0229 gatB	-	transcriptional antiterminator	56,68	5,82	-0,96
SACOL1256	trmD	rpsP rimM trmD rplS	-	tRNA (guanine-N1)-methyltransferase	1441,41	10,49	-0,96
SACOL2221	rpmD	adk secY rplO rpmD rpsE rplR rplF rpsH rpsN2 rplE rplX rplN rpsQ rpmC rplP rpsC rplV rpsS rplB rplW rplD rplC rpsJ	-	50S ribosomal protein L30	21666,36	14,40	-0,96
SACOL0861	cspC	cspC	-	CSD family cold shock protein	52488,33	15,68	-0,97
SACOL1947	SACOL1947	SACOL1947	-	hypothetical protein	195,84	7,61	-0,97
SACOL0146	cap5K	cap5ABCDEFGHIJKLMNOP	SigB	capsular polysaccharide synthesis protein Cap5K	198,60	7,63	-0,97
SACOL1734	gapB	gapB	-	glyceraldehyde 3-phosphate dehydrogenase 2	6945,78	12,76	-0,97
SACOL0810	tagO	tagO	-	glycosyl transferase, group 4 family protein	1602,32	10,65	-0,97
SACOL1509	ndk	ndk	-	nucleoside diphosphate kinase	863,04	9,75	-0,97
SACOL2728	SACOL2728	SACOL2728	-	hypothetical protein	69,35	6,12	-0,98
SACOL1038	SACOL1038	SACOL1038 SACOL1039 SACOL1040	-	membrane protein	42,96	5,42	-0,98
SACOL0201	hptR	hptRSA	-	AraC family DNA-binding response regulator	237,55	7,89	-0,98
SACOL2068	kdpA	SACOL2065 kdpC kdpB kdpA kdpF	MgrA	K+-transporting ATPase, A subunit	164,84	7,36	-0,98
SACOL1480	SACOL1480	ypcP SACOL1480	-	hypothetical protein	3322,92	11,70	-0,98
SACOL1510	hepT	hepT ubiE SACOL1512	-	polyprenyl synthetase	1656,27	10,69	-0,98
SACOL0202	hptS	hptRSA	-	sensor histidine kinase	283,84	8,15	-0,98
SACOL0425	ltrA	ltrA	-	low temperature requirement protein A	284,28	8,15	-0,98
SACOL0888	SACOL0888	SACOL0888 SACOL0889	-	pathogenicity island, lipoprotein, putative	119,89	6,91	-0,99
SACOL1196	murD	mraY murD divIB	MgrA	UDP-N-acetylmuramoylalanine--D-glutamate ligase	3541,88	11,79	-0,99
SACOL2100	atpE	atpCDGAHFEBI mnaA upp glyA ywlG	GraRS	proton-translocating F-type ATPase epsilon subunit C	4772,15	12,22	-0,99
SACOL1384	opuD1	opuD1	-	osmoprotectant transporter, BCCT family	4962,54	12,28	-0,99
SACOL1126	ctaM	ctaB ctaM	-	hypothetical protein	1953,77	10,93	-0,99
SACOL0404	mepR	mepRA	-	MarR family transcriptional regulator	1442,49	10,49	-0,99
SACOL2486	SACOL2486	SACOL2485 SACOL2486	-	hypothetical protein	146,39	7,19	-0,99
SACOL2483	SACOL2483	SACOL2483	-	transporter	412,98	8,69	-1,00
SACOL2227	rplE	adk secY rplO rpmD rpsE rplR rplF rpsH rpsN2 rplE rplX rplN rpsQ rpmC rplP rpsC rplV rpsS rplB rplW rplD rplC rpsJ	-	50S ribosomal protein L5	34038,36	15,05	-1,00
SACOL0330	SACOL0330	SACOL0330	-	conserved hypothetical protein	587,34	9,20	-1,00
SACOL2070	kdpD	kdpD kdpE	-	sensor histidine kinase KdpD	766,43	9,58	-1,00
SACOL0944	ndh2	ndh2	-	NADH dehydrogenase	13106,76	13,68	-1,00
SACOL0259	SACOL0259	SACOL0259	GraRS	hypothetical protein	240,81	7,91	-1,01
SACOL2224	rplF	adk secY rplO rpmD rpsE rplR rplF rpsH rpsN2 rplE rplX rplN rpsQ rpmC rplP rpsC rplV rpsS rplB rplW rplD rplC rpsJ	-	50S ribosomal protein L6	34928,23	15,09	-1,01
SACOL2162	SACOL2162	SACOL2160 SACOL2161 SACOL2162	-	hypothetical protein	3069,39	11,58	-1,01
SACOL0887	seq	sek sei	-	enterotoxin type I	108,66	6,76	-1,01
SACOL0647	SACOL0647	SACOL0647	-	hypothetical protein	106,33	6,73	-1,01
SACOL0130	ushA	SACOL0129 ushA	-	5-nucleotidase	462,36	8,85	-1,01
SACOL2223	rplR	adk secY rplO rpmD rpsE rplR rplF rpsH rpsN2 rplE rplX rplN rpsQ rpmC rplP rpsC rplV rpsS rplB rplW rplD rplC rpsJ	-	50S ribosomal protein L18	31245,91	14,93	-1,01
SACOL1971	sspA	sspB2 sspA	-	Staphostatin A	165,71	7,37	-1,02
SACOL1991	SACOL1991	SACOL1989 SACOL1990 SACOL1991	-	hypothetical protein	493,35	8,95	-1,02
SACOL0648	SACOL0648	SACOL0648	-	hypothetical protein	104,30	6,70	-1,02
SACOL2322	hipO2	hipO2	-	amino acid amidohydrolase	236,54	7,89	-1,02
SACOL1227	def2	def2 fmt sun rlmN stp1 pknB	-	peptide deformylase	530,68	9,05	-1,02
SACOL0871	SACOL0871	SACOL0871	MgrA	hypothetical protein	724,40	9,50	-1,02
SACOL1160	sdhB	sdhC sdhA sdhB	CcpA	succinate dehydrogenase iron-sulfur subunit	11288,86	13,46	-1,02
SACOL0249	bglR	bglR	-	GntR family transcriptional regulator	669,67	9,39	-1,02
SACOL1837	metK	metK	-	S-adenosylmethionine synthetase	3585,19	11,81	-1,02
SACOL0008	hutH	hutH	-	histidine ammonia-lyase	496,41	8,96	-1,02
SACOL1508	SACOL1508	SACOL1507 SACOL1508	-	hypothetical protein	36,90	5,21	-1,02
SACOL0605	tadA	tadA	-	putative tRNA-specific adenosine deaminase	85,98	6,43	-1,02
SACOL2226	rpsN_2	adk secY rplO rpmD rpsE rplR rplF rpsH rpsN2 rplE rplX rplN rpsQ rpmC rplP rpsC rplV rpsS rplB rplW rplD rplC rpsJ	-	ribosomal protein S14	20155,27	14,30	-1,03
SACOL2228	rplX	adk secY rplO rpmD rpsE rplR rplF rpsH rpsN2 rplE rplX rplN rpsQ rpmC rplP rpsC rplV rpsS rplB rplW rplD rplC rpsJ	-	50S ribosomal protein L24	23465,54	14,52	-1,03
SACOL2037	mutS	mutS	-	DNA mismatch repair ATPase	80,92	6,34	-1,03
SACOL2197	map-w	map-w	SigB	truncated MHC class II analog protein	194,68	7,60	-1,03
SACOL0236	tarI'	tarI' tarJ' tarL'	-	2-C-methyl-D-erythritol 4-phosphate cytidylyltransferase	755,04	9,56	-1,03
SACOL2332	galM	SACOL2331 galM ynfA	-	aldose 1-epimerase	1150,60	10,17	-1,03
SACOL1934	SACOL1934	SACOL1934	-	hypothetical protein	296,43	8,21	-1,03
SACOL2514	gntP	gntPKR SACOL2517	GraRS	GntP family gluconate:proton (H+) symporter	842,43	9,72	-1,03
SACOL1535	srrA	srrB srrA	-	DNA-binding response regulator SrrA	10002,75	13,29	-1,03
SACOL1850	SACOL1850	SACOL1850	-	hypothetical protein	186,24	7,54	-1,03
SACOL0646	SACOL0646	SACOL0646	-	hypothetical protein	105,16	6,72	-1,03
SACOL2099	atpF	atpCDGAHFEBI mnaA upp glyA ywlG	GraRS	proton-translocating F-type ATPase subunit B	6953,44	12,76	-1,03
SACOL2225	rpsH	adk secY rplO rpmD rpsE rplR rplF rpsH rpsN2 rplE rplX rplN rpsQ rpmC rplP rpsC rplV rpsS rplB rplW rplD rplC rpsJ	-	30S ribosomal protein S8	30885,25	14,91	-1,03
SACOL1859	SACOL1859	SACOL1859	-	hypothetical protein	202,72	7,66	-1,04
SACOL1076	purS	purEKCSQLFMNHD	PurR	phosphoribosylformylglycinamidine synthase	495,24	8,95	-1,04
SACOL0253	rbsK	rbsKDU	TraP	ribokinase	381,16	8,57	-1,04
SACOL1359	SACOL1359	SACOL1359	-	hypothetical protein	300,34	8,23	-1,04
SACOL1530	SACOL1530	SACOL1528 SACOL1529 SACOL1530	-	hypothetical protein	235,65	7,88	-1,04
SACOL1229	sun	def2 fmt sun rlmN stp1 pknB	-	sun protein	3360,86	11,71	-1,05
SACOL0208	SCIN_2	SCIN_2	GraRS	hypothetical protein	51,38	5,68	-1,05
SACOL2436	SACOL2436	glxK SACOL2436	VraSR	hypothetical protein	1613,96	10,66	-1,05
SACOL1779	sgtA	sgtA	-	transglycosylase domain-containing protein	466,29	8,87	-1,05
SACOL0203	hptA	hptRSA	-	iron compound ABC transporter iron compound-binding protein	176,20	7,46	-1,05
SACOL2288	SACOL2288	SACOL2288 sarY SACOL2290	MgrA	hypothetical protein	1449,56	10,50	-1,05
SACOL1650	nadD	holA comEC comEB comEA ubiE rsfS yqeK nadD yhbY aroE yqeH yqeG mtn	-	nicotinate (nicotinamide) nucleotide adenylyltransferase	2996,78	11,55	-1,06
SACOL0429	metF	metEFCI	MgrA	methylenetetrahydrofolate reductase/homocysteine S-methyltransferase	234,28	7,87	-1,06
SACOL2651	estA	estA	-	tributyrin esterase EstA	711,74	9,48	-1,06
SACOL2229	rplN	adk secY rplO rpmD rpsE rplR rplF rpsH rpsN2 rplE rplX rplN rpsQ rpmC rplP rpsC rplV rpsS rplB rplW rplD rplC rpsJ	-	50S ribosomal protein L14	28247,10	14,79	-1,06
SACOL1700	rpmA	rpmA SACOL1701 rplU	-	50S ribosomal protein L27	19023,86	14,22	-1,06
SACOL0737	SACOL0737	SACOL0736 SACOL0737 SACOL0738	SigB	possible lipoprotein	578,29	9,18	-1,06
SACOL1225	SACOL1225	SACOL1225	GraRS	hypothetical lipoprotein	237,89	7,89	-1,06
SACOL2230	rpsQ	adk secY rplO rpmD rpsE rplR rplF rpsH rpsN2 rplE rplX rplN rpsQ rpmC rplP rpsC rplV rpsS rplB rplW rplD rplC rpsJ	-	30S ribosomal protein S17	24600,53	14,59	-1,07
SACOL1228	fmt	def2 fmt sun rlmN stp1 pknB	-	methionyl-tRNA formyltransferase	1708,19	10,74	-1,07
SACOL0946	nhaC	nhaC SACOL0947	GraRS	NhaC family sodium:proton (Na :H) antiporter	407,95	8,67	-1,07
SACOL0482	lpl5	lpl5	-	staphylococcal tandem lipoprotein	29,47	4,88	-1,07
SACOL1913	cspR	cspR queG	-	RNA methyltransferase	455,98	8,83	-1,07
SACOL0336	SACOL0336	SACOL0335 - SACOL0338 ssb1 SACOL0340 - SACOL0349	-	hypothetical protein	61,87	5,95	-1,07
SACOL2558	SACOL2558	-	-	hypothetical protein	88,78	6,47	-1,07
SACOL1175	SACOL1175	SACOL1174 SACOL1175	MgrA	hypothetical protein	555,29	9,12	-1,08
SACOL2231	rpmC	adk secY rplO rpmD rpsE rplR rplF rpsH rpsN2 rplE rplX rplN rpsQ rpmC rplP rpsC rplV rpsS rplB rplW rplD rplC rpsJ	-	50S ribosomal protein L29	18350,07	14,16	-1,08
SACOL1905	airR	airR airS	-	LuxR family DNA-binding response regulator	198,32	7,63	-1,08
SACOL1976	nos	nos pheA	-	nitric-oxide synthase, oxygenase subunit	1120,73	10,13	-1,08
SACOL1066	fmtA	fmtA	-	fmt protein	341,48	8,42	-1,09
SACOL2390	nreB	nreA nreB nreC narI narJ narH narG	SigB	sensory box histidine kinase	1445,83	10,50	-1,09
SACOL1878	epiA	epiGEFPDCBA	SigB	lantibiotic epidermin EpiA	47,51	5,57	-1,09
SACOL1642	rpsT	rpsT	-	30S ribosomal protein S20	6132,14	12,58	-1,09
SACOL2098	atpH	atpCDGAHFEBI mnaA upp glyA ywlG	GraRS	proton-translocating F-type ATPase beta subunit	7799,35	12,93	-1,09
SACOL2383	SACOL2383	SACOL2383	-	hypothetical protein	282,36	8,14	-1,09
SACOL0289	SACOL0289	SACOL0289	-	conserved hypothetical protein	173,10	7,44	-1,09
SACOL0145	cap5J	cap5ABCDEFGHIJKLMNOP	SigB	capsular polysaccharide biosynthesis protein Cap5J	230,39	7,85	-1,09
SACOL0481	lpl4	lpl4	-	staphylococcal tandem lipoprotein	41,46	5,37	-1,10
SACOL0323	SACOL0323	SACOL0322 SACOL0323	-	hypothetical protein	444,43	8,80	-1,10
SACOL1606	gluP	SACOL1602 SACOL1603 glk SACOL1605 gluP fthC	-	peptidase, rhomboid family protein	2520,96	11,30	-1,10
SACOL1192	mraW	mraZ mraW ftsL pbp1	-	S-adenosyl-methyltransferase MraW	4855,61	12,25	-1,11
SACOL1171	SACOL1171	SACOL1171	-	hypothetical protein	178,51	7,48	-1,11
SACOL2303	suhB	suhB	SigB	inositol monophosphatase family protein	1359,75	10,41	-1,11
SACOL1367	SACOL1367	SACOL1367	-	amino acid permease	455,72	8,83	-1,11
SACOL1848	SACOL1848	SACOL1848	-	hypothetical protein	192,64	7,59	-1,11
SACOL1111	potD	SACOL1107 potA potB potC potD	-	spermidine/putrescine ABC transporter substrate-binding protein	2532,09	11,31	-1,12
SACOL2242	pbuG	pbuG	GraRS	NCS2 family nucleobase:cation symporter-2	11694,01	13,51	-1,12
SACOL1275	SACOL1275	SACOL1275	-	hypothetical protein	1718,64	10,75	-1,12
SACOL0665	btuF	btuF fecD SACOL0667 SACOL0668 SACOL0669 SACOL0670	-	iron (Fe3) ABC superfamily ATP binding cassette transporter, binding protein	194,99	7,61	-1,12
SACOL0938	dltD	dltX dltA dltB dltC dltD	-	DltD protein	10027,65	13,29	-1,12
SACOL1114	SACOL1114	SACOL1114	-	Mn2+/Fe2+ transporter, NRAMP family	9282,35	13,18	-1,13
SACOL0437	rpsF	rpsF ssb rpsR	-	30S ribosomal protein S6	24499,70	14,58	-1,13
SACOL1584	SACOL1584	SACOL1584	-	hypothetical protein	96,65	6,59	-1,13
SACOL2097	atpA	atpCDGAHFEBI mnaA upp glyA ywlG	GraRS	proton-translocating F-type ATPase alpha subunit	19674,46	14,26	-1,13
SACOL1652	aroE	holA comEC comEB comEA ubiE rsfS yqeK nadD yhbY aroE yqeH yqeG mtn	-	shikimate 5-dehydrogenase	2294,30	11,16	-1,14
SACOL0612	SACOL0612	SACOL0612	-	glycosyl transferase, group 1 family protein	4180,04	12,03	-1,14
SACOL2096	atpG	atpCDGAHFEBI mnaA upp glyA ywlG	GraRS	proton-translocating F-type ATPase gamma subunit	12776,95	13,64	-1,14
SACOL1106	SACOL1106	SACOL1106	-	hypothetical protein	459,40	8,84	-1,14
SACOL1979	sdcS	sdcS	-	sodium-dependent transporter	1049,40	10,04	-1,14
SACOL1398	msrR	msrR	-	transcriptional regulator	454,29	8,83	-1,14
SACOL2727	SACOL2727	SACOL2727	-	integrase/recombinase core subunit	372,18	8,54	-1,14
SACOL1174	SACOL1174	SACOL1174 SACOL1175	MgrA	hypothetical protein	198,32	7,63	-1,14
SACOL0405	mepA	mepRA	-	MATE efflux family protein	1327,26	10,37	-1,14
SACOL2620	gabT	gabT	MgrA	4-aminobutyrate transaminase	389,17	8,60	-1,15
SACOL2661	SACOL2661	SACOL2661	-	hypothetical protein	67,81	6,08	-1,15
SACOL2435	glxK	glxK SACOL2436	VraSR	glycerate kinase	1205,30	10,24	-1,15
SACOL2352	tcaA	tcaA	-	tcaA protein	459,54	8,84	-1,15
SACOL0054	SACOL0054	SACOL0052 SACOL0054	-	hypothetical protein	2308,25	11,17	-1,16
SACOL0448	SACOL0448	SACOL0448	-	hypothetical protein	542,25	9,08	-1,16
SACOL2577	crtM	crtNMQIO	SigB	squalene desaturase	234,44	7,87	-1,16
SACOL0669	SACOL0669	btuF fecD SACOL0667 SACOL0668 SACOL0669 SACOL0670	-	hypothetical protein	16263,85	13,99	-1,16
SACOL2329	rpiA	rpiA	-	ribose-5-phosphate isomerase A	768,00	9,58	-1,17
SACOL0199	SACOL0199	SACOL0199	SaeRS	hypothetical protein	49,91	5,64	-1,17
SACOL1362	hom	hom thrC thrB	CodY	homoserine dehydrogenase	347,05	8,44	-1,17
SACOL0989	oppB	oppB	-	hypothetical protein	28,18	4,82	-1,17
SACOL1651	yhbY	holA comEC comEB comEA ubiE rsfS yqeK nadD yhbY aroE yqeH yqeG mtn	-	RNA-binding protein YbhY	1063,97	10,06	-1,17
SACOL1849	SACOL1849	SACOL1849	-	hypothetical protein	132,78	7,05	-1,17
SACOL2431	msbA3	SACOL2430 msbA3	GraRS	ABC transporter, ATP-binding protein	39,60	5,31	-1,18
SACOL1388	plsY	plsY	-	hypothetical protein	3174,43	11,63	-1,18
SACOL0814	comFC	comFA comFC	-	hypothetical protein	146,82	7,20	-1,18
SACOL0281	SACOL0281	esaA essA esaB essB yukA esxC esxB esaE esxD SACOL0281	GraRS	hypothetical protein	2280,15	11,15	-1,18
SACOL2580	crtO	crtNMQIO	SigB	hypothetical membrane protein	397,84	8,64	-1,18
SACOL0789	queF	queF SACOL0790	LexA	7-cyano-7-deazaguanine reductase	666,12	9,38	-1,18
SACOL2363	lctP_2	lctP_2	GraRS	LctP family L-lactate permease	19300,27	14,24	-1,19
SACOL2072	cshA	cshA	-	DEAD/DEAH box helicase	86430,14	16,40	-1,19
SACOL1654	yqeG	holA comEC comEB comEA ubiE rsfS yqeK nadD yhbY aroE yqeH yqeG mtn	-	haloacid dehalogenase-like hydrolase	1407,40	10,46	-1,19
SACOL0439	rpsR	rpsF ssb rpsR	-	30S ribosomal protein S18	14413,02	13,82	-1,19
SACOL0438	ssb	rpsF ssb rpsR	-	single-stranded DNA-binding protein	32790,52	15,00	-1,19
SACOL0812	SACOL0812	SACOL0812	-	degV family protein	3384,33	11,72	-1,20
SACOL2717	SACOL2717	SACOL2717	SigB	hypothetical protein	322,67	8,33	-1,20
SACOL1195	mraY	mraY murD divIB	MgrA	phospho-N-acetylmuramoyl-pentapeptide- transferase	1315,92	10,36	-1,20
SACOL2662	SACOL2662	SACOL2662 manP pmi	-	BglG family transcriptional antiterminator	209,49	7,71	-1,21
SACOL1653	yqeH	holA comEC comEB comEA ubiE rsfS yqeK nadD yhbY aroE yqeH yqeG mtn	-	GTPase family protein	3079,63	11,59	-1,21
SACOL1020	SACOL1020	SACOL1020	-	hypothetical protein	4675,15	12,19	-1,22
SACOL1529	SACOL1529	SACOL1528 SACOL1529 SACOL1530	-	hypothetical protein	87,95	6,46	-1,22
SACOL0018	purA	purA	PurR	adenylosuccinate synthase	4941,12	12,27	-1,22
SACOL2206	rpsI	rpsI rplM	-	30S ribosomal protein S9	18345,12	14,16	-1,22
SACOL2467	SACOL2467	SACOL2467	-	lipoprotein	798,71	9,64	-1,22
SACOL0311	nanT	nanT nanA	-	sodium:solute symporter family protein	133,28	7,06	-1,22
SACOL0334	SACOL0334	SACOL0334	-	hypothetical protein	50,23	5,65	-1,22
SACOL0587	rsmC	secE nusG rplK rplA rplJ rplL rsmC	-	hypothetical protein	3512,43	11,78	-1,22
SACOL1655	mtnN	holA comEC comEB comEA ubiE rsfS yqeK nadD yhbY aroE yqeH yqeG mtn	-	5'-methylthioadenosine/S-adenosylhomocysteine nucleosidase	1228,47	10,26	-1,23
SACOL0937	dltC	SACOL0934 dltA dltB dltC dltD	-	D-alanine--poly(phosphoribitol) ligase subunit 2 DltC	2985,50	11,54	-1,23
SACOL2582	SACOL2582	SACOL2582	GraRS	possible acetyltransferase	1404,99	10,46	-1,23
SACOL1184	eta	eta	-	exfoliative toxin	194,16	7,60	-1,23
SACOL0936	dltB	SACOL0934 dltA dltB dltC dltD	-	DltB protein	7063,43	12,79	-1,23
SACOL1978	SACOL1978	SACOL1978	-	hypothetical protein	95,88	6,58	-1,23
SACOL1914	queG	cspR queG	-	iron-sulfur cluster-binding protein	815,40	9,67	-1,23
SACOL1586	SACOL1586	SACOL1586	-	hypothetical protein	33,24	5,05	-1,23
SACOL1073	purE	purEKCSQLFMNHD	PurR	5-(carboxyamino)imidazole ribonucleotide mutase	130,23	7,02	-1,24
SACOL1074	purK	purEKCSQLFMNHD	PurR	5-(carboxyamino)imidazole ribonucleotide synthase	463,42	8,86	-1,24
SACOL1110	potC	SACOL1107 potA potB potC potD	-	spermidine/putrescine ABC transporter, permease protein	1469,68	10,52	-1,24
SACOL2024	agrD	agrBDCA	TraP	accessory gene regulator protein D	493,92	8,95	-1,24
SACOL0322	SACOL0322	SACOL0322 SACOL0323	-	prophage L54a, Cro-related protein	281,49	8,14	-1,24
SACOL0254	rbsD	rbsKDU	TraP	ribose ABC transporter, membrane protein	236,63	7,89	-1,24
SACOL0073	SACOL0073	SACOL0073	-	hypothetical protein	30,68	4,94	-1,24
SACOL0083	SACOL0083	SACOL0083	-	tandem lipoprotein	115,36	6,85	-1,24
SACOL1274	rpsB	rpsB	-	30S ribosomal protein S2	18840,22	14,20	-1,25
SACOL2042	ilvD	ilvDBNC-leuABCD-ilvA2	CodY	dihydroxy-acid dehydratase	277,34	8,12	-1,25
SACOL1191	mraZ	mraZ mraW ftsL pbp1	-	cell division protein MraZ	4794,34	12,23	-1,25
SACOL1426	cvfB	cvfB	-	hypothetical protein	9704,04	13,24	-1,25
SACOL1963	putP	putP	CodY	high affinity proline permease	1342,86	10,39	-1,26
SACOL0689	mntB	mntABC	-	ABC transporter permease	221,27	7,79	-1,26
SACOL1723	SACOL1723	SACOL1723 SACOL1724	-	conserved hypothetical protein	1462,58	10,51	-1,27
SACOL0813	comFA	comFA comFC	-	comf operon protein 1	223,18	7,80	-1,27
SACOL1860	SACOL1860	SACOL1860	-	hypothetical protein	38,69	5,27	-1,27
SACOL0583	rplK	secE nusG rplK rplA rplJ rplL rsmC	-	50S ribosomal protein L11	23187,69	14,50	-1,27
SACOL0313	nanK	nanK	-	ROK family protein	61,42	5,94	-1,27
SACOL2389	nreC	nreA nreB nreC narI narJ narH narG	SigB	transcriptional regulatory protein	745,64	9,54	-1,27
SACOL1172	SACOL1172	SACOL1172	-	hypothetical protein	604,60	9,24	-1,27
SACOL1043	tarM	SACOL1042 tarM	-	glycosyl transferase, group 1 family protein	1136,31	10,15	-1,28
SACOL0991	oppB	SACOL0990 oppB oppC oppD oppF oppA	MgrA	oligopeptide ABC transporter, membrane protein	413,00	8,69	-1,28
SACOL1828	comK2	comK2	-	hypothetical protein	66,72	6,06	-1,29
SACOL0033	mecA	-	-	penicillin-binding protein 2'	79453,99	16,28	-1,29
SACOL0156	SACOL0156	SACOL0156	SigB	hypothetical protein	141,49	7,14	-1,29
SACOL0287	SACOL0287	SACOL0287	-	hypothetical protein	120,51	6,91	-1,29
SACOL1482	SACOL1482	SACOL1482	-	hypothetical protein	574,01	9,16	-1,30
SACOL0992	oppC	SACOL0990 oppB oppC oppD oppF oppA	MgrA	oligopeptide ABC transporter, membrane protein	562,30	9,14	-1,30
SACOL2559	SACOL2559	SACOL2559	-	hypothetical protein	196,92	7,62	-1,30
SACOL1908	fumC	fumC	-	fumarate hydratase	2711,77	11,41	-1,31
SACOL0790	SACOL0790	queF SACOL0790	LexA	integral membrane domain-containing protein	697,33	9,45	-1,31
SACOL2112	rpmE2	rpmE2	-	50S ribosomal protein L31	16572,81	14,02	-1,31
SACOL1430	dapA	lysC asd dapA dapB dapD	CodY	dihydrodipicolinate synthase	459,14	8,84	-1,31
SACOL1137	rpmF	SACOL1136 rpmF	-	ribosomal protein L32	5755,26	12,49	-1,32
SACOL0286	SACOL0286	SACOL0286	-	hypothetical protein	107,45	6,75	-1,32
SACOL2584	isaA	isaA	CodY	immunodominant antigen A	11557,45	13,50	-1,32
SACOL1348	SACOL1348	SACOL1346 SACOL1347 SACOL1348	MgrA	hypothetical protein	57,23	5,84	-1,32
SACOL1109	potB	SACOL1107 potA potB potC potD	-	spermidine/putrescine ABC transporter, permease protein	978,51	9,93	-1,33
SACOL2576	crtN	crtNMQIO	SigB	squalene synthase	436,06	8,77	-1,33
SACOL2238	rplD	adk secY rplO rpmD rpsE rplR rplF rpsH rpsN2 rplE rplX rplN rpsQ rpmC rplP rpsC rplV rpsS rplB rplW rplD rplC rpsJ	-	ribosomal protein L4	30286,16	14,89	-1,34
SACOL0584	rplA	secE nusG rplK rplA rplJ rplL rsmC	-	50S ribosomal protein L1	36385,36	15,15	-1,34
SACOL1556	SACOL1556	SACOL1556	-	hypothetical protein	69,46	6,12	-1,34
SACOL0516	treP	trePCR	TreR	PTS family trehalose (tre) porter component IIBC	1786,45	10,80	-1,34
SACOL1343	SACOL1343	-	-	hypothetical protein	35,91	5,17	-1,34
SACOL0545	rplY	rplY pth mfd SACOL0548 mazG SACOL0550 divIC	AgrA	50S ribosomal protein L25/general stress protein rplY	23987,68	14,55	-1,34
SACOL0885	int	int	-	pathogenicity island protein, integrase	98,55	6,62	-1,35
SACOL2567	SACOL2567	SACOL2567	-	hypothetical protein	1411,59	10,46	-1,35
SACOL0177	murQ	SACOL0176 murQ murP SACOL0179	CcpA	N-acetylmuramic acid-6-phosphate etherase	218,74	7,77	-1,35
SACOL0183	SACOL0183	SACOL0181 sepS16B SACOL0183	-	hypothetical protein	922,26	9,85	-1,35
SACOL1932	sgtB	sgtB	VraSR	monofunctional glycosyltransferase	1829,81	10,84	-1,36
SACOL0088	nptA	nptA	GraRS	possible PnaS family phosphate:sodium (Na+) symporter	359,35	8,49	-1,36
SACOL2134	SACOL2134	SACOL2134 manA1	-	hypothetical protein	328,48	8,36	-1,36
SACOL0329	SACOL0329	SACOL0329	-	conserved hypothetical protein	173,36	7,44	-1,37
SACOL2240	rpsJ	adk secY rplO rpmD rpsE rplR rplF rpsH rpsN2 rplE rplX rplN rpsQ rpmC rplP rpsC rplV rpsS rplB rplW rplD rplC rpsJ	-	ribosomal protein S10	18594,31	14,18	-1,37
SACOL0076	SACOL0076	SACOL0076	-	hypothetical protein	774,95	9,60	-1,37
SACOL0630	SACOL0630	SACOL0630	SigB	APC family amino acid-polyamine-organocation transporter	1582,94	10,63	-1,38
SACOL2333	ynfA	SACOL2331 galM ynfA	-	hypothetical protein	423,05	8,72	-1,38
SACOL0858	emp	emp	MgrA	secretory extracellular matrix and plasma binding protein	62,94	5,98	-1,38
SACOL0114	cap5M2	galE cap5M2	MgrA	capsular polysaccharide biosynthesis protein	29,80	4,90	-1,39
SACOL0333	SACOL0333	SACOL0333	-	hypothetical protein	135,40	7,08	-1,39
SACOL0244	scdA	scdA	-	cell wall biosynthesis protein ScdA	628,23	9,30	-1,39
SACOL0331	SACOL0331	SACOL0331	-	hypothetical protein	91,69	6,52	-1,39
SACOL2508	gtaB	gtaB	-	UTP-glucose-1-phosphate uridylyltransferase	729,20	9,51	-1,39
SACOL1333	SACOL1333	SACOL1333	GraRS	hypothetical protein	38,72	5,28	-1,39
SACOL0182	sepS16B	SACOL0181 sepS16B SACOL0183	-	hypothetical protein	945,62	9,89	-1,39
SACOL0178	murP	SACOL0176 murQ murP SACOL0179	CcpA	N-acetylmuramic acid phosphotransfer permease	270,33	8,08	-1,39
SACOL1483	SACOL1483	SACOL1483	-	hypothetical protein	807,65	9,66	-1,39
SACOL2347	emrB	emrA emrB	-	EmrB/QacA family drug resistance transporter	445,02	8,80	-1,40
SACOL2207	rplM	rpsI rplM	-	50S ribosomal protein L13	15021,40	13,87	-1,41
SACOL0299	SACOL0299	SACOL0299	-	hypothetical protein	110,16	6,78	-1,41
SACOL2306	spdB	spdB	-	hypothetical protein	305,39	8,25	-1,41
SACOL1347	SACOL1347	SACOL1346 SACOL1347 SACOL1348	MgrA	hypothetical protein	413,22	8,69	-1,42
SACOL2734	SACOL2734	SACOL2733 SACOL2734	-	hypothetical protein	71,86	6,17	-1,43
SACOL1701	SACOL1701	rpmA SACOL1701 rplU	-	hypothetical protein	15169,04	13,89	-1,43
SACOL1136	SACOL1136	SACOL1136 rpmF	-	conserved hypothetical protein	12950,89	13,66	-1,43
SACOL1724	SACOL1724	SACOL1723 SACOL1724	-	MutT/nudix family protein	921,96	9,85	-1,44
SACOL1775	ptaA	SACOL1774 ptaA	CodY	PTS system, IIBC components	1378,30	10,43	-1,44
SACOL0435	ychF	kefA SACOL0434 ychF	-	GTP-dependent nucleic acid-binding protein	3237,14	11,66	-1,44
SACOL2120	rpoE	rpoE	-	DNA-directed RNA polymerase subunit delta	5701,35	12,48	-1,44
SACOL0860	nuc	nuc	SigB	thermonuclease precursor	21,39	4,42	-1,44
SACOL2583	SACOL2583	SACOL2583	-	acetyltransferase	126,69	6,99	-1,44
SACOL2076	SACOL2076	SACOL2076 SACOL2077 csoR	SigB	hypothetical protein	5376,49	12,39	-1,46
SACOL1702	rplU	rpmA SACOL1701 rplU	-	50S ribosomal protein L21	19210,79	14,23	-1,46
SACOL1725	rplT	rplT rpmI infC	-	ribosomal protein L20	9291,84	13,18	-1,47
SACOL1342	SACOL1342	SACOL1342	-	hypothetical protein	17,22	4,11	-1,47
SACOL2122	coaA	coaA	-	pantothenate kinase	724,34	9,50	-1,47
SACOL1478	ald	steT ilvA1 ald	CodY	alanine dehydrogenase	55,02	5,78	-1,47
SACOL1769	rpsD	rpsD	-	30S ribosomal protein S4	22754,84	14,47	-1,48
SACOL0169	argC	argBJCD	ArgR	N-acetyl-gamma-glutamyl-phosphate reductase	51,89	5,70	-1,48
SACOL0327	SACOL0327	SACOL0327	-	hypothetical protein	251,15	7,97	-1,48
SACOL1108	potA	SACOL1107 potA potB potC potD	-	spermidine/putrescine ABC transporter ATP-binding protein	1361,79	10,41	-1,48
SACOL0660	adh1	adh1	SigB	alcohol dehydrogenase	2984,99	11,54	-1,48
SACOL0411	yeiH	yeiH	-	hypothetical protein	234,28	7,87	-1,49
SACOL0325	SACOL0325	SACOL0325 SACOL0326	-	prophage L54a, antirepressor, putative	761,60	9,57	-1,49
SACOL0434	SACOL0434	kefA SACOL0434 ychF	-	hypothetical protein	544,82	9,09	-1,49
SACOL2537	SACOL2537	SACOL2537 SACOL2538	-	ABC transporter ATP-binding protein	180,32	7,49	-1,50
SACOL2107	ptpB	ptpB SACOL2108 hemK prfA tdk	-	phosphotyrosine protein phosphatase	578,35	9,18	-1,50
SACOL0255	rbsU	rbsKDU	TraP	DMT superfamily drug/metabolite transporter	519,20	9,02	-1,50
SACOL1838	pckA	pckA	-	phosphoenolpyruvate carboxykinase	4694,14	12,20	-1,51
SACOL0170	argD	argBJCD	ArgR	ornithine--oxo-acid transaminase	47,13	5,56	-1,51
SACOL0144	cap5I	cap5ABCDEFGHIJKLMNOP	SigB	capsular polysaccharide biosynthesis protein Cap5I	256,29	8,00	-1,51
SACOL0181	SACOL0181	SACOL0181 sepS16B SACOL0183	-	hypothetical protein	1187,36	10,21	-1,51
SACOL1042	SACOL1042	SACOL1042 tarM	-	hypothetical protein	184,10	7,52	-1,52
SACOL0141	cap5F	cap5ABCDEFGHIJKLMNOP	SigB	capsular polysaccharide biosynthesis protein Cap5F	225,88	7,82	-1,52
SACOL0566	nupC	nupC	-	nucleoside permease NupC	1468,11	10,52	-1,53
SACOL0935	dltA	SACOL0934 dltA dltB dltC dltD	-	D-alanine--poly(phosphoribitol) ligase subunit 1 DltA	5928,07	12,53	-1,53
SACOL2382	gltT	gltT	-	proton/sodium-glutamate symport protein	9135,57	13,16	-1,53
SACOL2023	agrB	agrBDCA	TraP	accessory gene regulator protein B	1298,12	10,34	-1,53
SACOL0110	SACOL0110	SACOL0110	-	hypothetical protein	83,06	6,38	-1,54
SACOL2376	scrA	scrA	ScrR	PTS family sucrose porter component IIBC	2875,13	11,49	-1,54
SACOL0024	adsA	adsA	-	5'-nucleotidase	270,22	8,08	-1,55
SACOL1816	putA	putA	-	proline dehydrogenase	3035,66	11,57	-1,55
SACOL1548	rnz	rnz	-	AtsA/ElaC family protein	601,59	9,23	-1,55
SACOL0066	SACOL0066	SACOL0066	-	hypothetical protein	96,98	6,60	-1,55
SACOL2315	SACOL2315	SACOL2315	-	hypothetical protein	365,89	8,52	-1,55
SACOL0852	SACOL0852	-	-	hypothetical protein	23,13	4,53	-1,56
SACOL2006	lukS	lukSF	SaeRS	possible leukocidin subunit	93,52	6,55	-1,56
SACOL2391	nreA	nreA nreB nreC narI narJ narH narG	SigB	hypothetical protein	866,48	9,76	-1,56
SACOL0303	SACOL0303	SACOL0303	-	5'-nucleotidase	25,30	4,66	-1,57
SACOL0326	SACOL0326	SACOL0325 SACOL0326	-	hypothetical protein	291,95	8,19	-1,57
SACOL2108	SACOL2108	ptpB SACOL2108 hemK prfA tdk	-	Sua5/YciO/YrdC/YwlC family protein	1222,53	10,26	-1,57
SACOL1758	ald2	ald2	CcpA	alanine dehydrogenase	1845,33	10,85	-1,58
SACOL0115	cap8H	cap8H cps2H SACOL0117	MgrA	glycosyltransferase	25,97	4,70	-1,58
SACOL0082	SACOL0082	SACOL0082	-	hypothetical protein	87,01	6,44	-1,58
SACOL0096	sarS	sarS	SigB	staphylococcal accessory regulator S	1642,83	10,68	-1,58
SACOL1969	purB	purB	PurR	adenylosuccinate lyase	2663,58	11,38	-1,58
SACOL1429	asd	lysC asd dapA dapB dapD	CodY	aspartate-semialdehyde dehydrogenase	443,17	8,79	-1,59
SACOL0081	SACOL0081	SACOL0081	-	hypothetical protein	24,24	4,60	-1,59
SACOL1257	rplS	rpsP rimM trmD rplS	-	ribosomal protein L19	21396,12	14,39	-1,61
SACOL0067	dus	dus	-	tRNA dihydrouridine synthase	2072,20	11,02	-1,61
SACOL0140	cap5E	cap5ABCDEFGHIJKLMNOP	SigB	capsular polysaccharide biosynthesis protein Cap5E	159,40	7,32	-1,62

					WT MHQ vs control								WT MHQ/ WT co	ΔmhqR MHQ/ ΔmhqR co	ΔmhqR MHQ/ WT MHQ	ΔmhqR co/ WT co	WT MHQ/ WT co	ΔmhqR MHQ/ ΔmhqR co	ΔmhqR MHQ/ WT MHQ	ΔmhqR co/ WT co	WT MHQ/ WT co	ΔmhqR MHQ/ ΔmhqR co	ΔmhqR MHQ/ WT MHQ	ΔmhqR co/ WT co
Locus	Gene symbol	Operon	Regulon	Function	Base mean	A-value	M-value	Fold change	Standard error	Wald statistic	P-value	Adjusted P-value	M-value	M-value	M-value	M-value	Fold change	Fold change	Fold change	Fold change	Adj. P-value	Adj. P-value	Adj. P-value	Adj. P-value
SACOL2393	narJ	nreA nreB nreC narI narJ narH narG	SigB	respiratory nitrate reductase subunit delta	94,60	6,56	-1,62	0,33	0,43	-3,79	0,000149	4,81E-04	-1,62	-2,07	-0,7	-0,17	0,33	0,24	0,62	0,89	4,81E-04	6,73E-10	8,69E-02	7,32E-01
SACOL1346	SACOL1346	SACOL1346 SACOL1347 SACOL1348	MgrA	hypothetical protein	549,65	9,10	-1,62	0,33	0,27	-5,99	2,09E-09	1,53E-08	-1,62	-0,39	0,74	-0,47	0,33	0,76	1,67	0,72	1,53E-08	3,39E-02	1,25E-03	5,68E-02
SACOL0809	SACOL0809	SACOL0809	MgrA	diguanylate cyclase GGDEF domain protein	1562,80	10,61	-1,63	0,32	0,26	-6,18	6,47E-10	5,20E-09	-1,63	-1,35	0,29	0,01	0,32	0,39	1,22	1,01	5,20E-09	2,07E-19	2,64E-01	9,83E-01
SACOL0931	pdxp	SACOL0930 pdxp ghrB	-	HAD-superfamily hydrolase	1721,62	10,75	-1,64	0,32	0,33	-4,95	7,59E-07	3,67E-06	-1,64	-0,83	0,89	0,06	0,32	0,56	1,85	1,04	3,67E-06	4,21E-12	1,63E-03	7,82E-01
SACOL2654	arcC	arcCDBA	ArcR	carbamate kinase	425,60	8,73	-1,64	0,32	0,24	-6,84	7,95E-12	8,10E-11	-1,64	-0,87	0,9	0,13	0,32	0,55	1,87	1,09	8,10E-11	1,09E-07	2,00E-04	5,59E-01
SACOL2718	SACOL2718	SACOL2718	-	anion transporter family protein	1041,08	10,02	-1,64	0,32	0,27	-6,13	8,7E-10	6,90E-09	-1,64	-0,97	0,59	-0,09	0,32	0,51	1,51	0,94	6,90E-09	8,08E-11	8,06E-03	7,56E-01
SACOL2335	SACOL2335	SACOL2334 SACOL2335	-	ABC transporter ATP-binding protein	191,23	7,58	-1,65	0,32	0,29	-5,59	2,21E-08	1,38E-07	-1,65	-0,39	0,4	-0,81	0,32	0,76	1,32	0,57	1,38E-07	8,97E-02	1,75E-01	1,93E-04
SACOL0788	dtpT	dtpT	-	proton-dependent oligopeptide transporter family protein	4645,70	12,18	-1,65	0,32	0,27	-6,15	7,92E-10	6,34E-09	-1,65	-0,64	0,74	-0,26	0,32	0,64	1,67	0,84	6,34E-09	1,00E-04	1,73E-03	2,71E-01
SACOL2113	rho	rho	-	transcription termination factor Rho	9654,87	13,24	-1,66	0,32	0,19	-8,69	3,65E-18	7,01E-17	-1,66	-1,17	0,43	-0,05	0,32	0,44	1,35	0,97	7,01E-17	5,58E-17	1,69E-02	8,36E-01
SACOL0171	brnQ1	brnQ1	GraRS	possible LIVCS family branched chain amino acid:cation symporter	711,02	9,47	-1,66	0,32	0,34	-4,82	1,44E-06	6,67E-06	-1,66	-0,71	0,65	-0,28	0,32	0,61	1,57	0,82	6,67E-06	3,00E-04	4,09E-02	3,09E-01
SACOL1845	SACOL1845	SACOL1845	-	hypothetical protein	90,05	6,49	-1,66	0,32	0,34	-4,96	7,18E-07	3,49E-06	-1,66	0,1	0,71	-0,93	0,32	1,07	1,64	0,52	3,49E-06	7,70E-01	3,24E-02	2,18E-03
SACOL2630	cudC	cudC	-	transcriptional regulator-like protein	586,31	9,20	-1,67	0,32	0,20	-8,32	8,73E-17	1,47E-15	-1,67	-0,83	0,7	-0,13	0,32	0,56	1,62	0,91	1,47E-15	6,47E-07	1,94E-03	5,17E-01
SACOL1608	rpmG_2	rpmG_2	-	ribosomal protein L33	2758,13	11,43	-1,67	0,31	0,29	-5,73	9,79E-09	6,52E-08	-1,67	-0,35	1,06	-0,26	0,31	0,78	2,08	0,84	6,52E-08	3,76E-02	5,07E-06	3,58E-01
SACOL0407	glpT	glpT	-	glycerol-3-phosphate transporter	336,34	8,39	-1,67	0,31	0,34	-4,87	1,14E-06	5,37E-06	-1,67	-1	0,17	-0,2	0,31	0,5	1,13	0,87	5,37E-06	5,86E-02	6,92E-01	7,40E-01
SACOL1323	miaA	glpK glpD SACOL1322 miaA hfq	SarA	tRNA delta(2)-isopentenylpyrophosphate transferase	1505,27	10,56	-1,68	0,31	0,24	-6,99	2,75E-12	2,92E-11	-1,68	-1,38	-0,18	-0,47	0,31	0,38	0,88	0,72	2,92E-11	5,77E-18	4,77E-01	2,40E-02
SACOL2356	hrtA	hrtA hrtB	-	ABC transporter ATP-binding protein	122,77	6,94	-1,68	0,31	0,30	-5,70	1,19E-08	7,77E-08	-1,68	-1,88	0,29	0,47	0,31	0,27	1,22	1,39	7,77E-08	6,15E-21	3,66E-01	4,54E-02
SACOL0609	sdrD	sdrD	TraP	Ser-Asp rich fibrinogen/bone sialoprotein-binding protein SdrD	1018,93	9,99	-1,68	0,31	0,30	-5,53	3,22E-08	1,95E-07	-1,68	-0,67	-0,79	-1,71	0,31	0,63	0,58	0,31	1,95E-07	8,63E-04	3,80E-03	9,27E-17
SACOL0142	cap5G	cap5A cap5B cap5C cap5D cap5E cap5F cap5G cap5H cap5I cap5J cap5K cap5L cap5M cap5N cap5O cap5P	-	UDP-N-acetylglucosamine 2-epimerase Cap5G	268,58	8,07	-1,68	0,31	0,29	-5,82	6E-09	4,10E-08	-1,68	0,21	1,02	-0,82	0,31	1,16	2,03	0,57	4,10E-08	3,62E-01	1,51E-04	1,09E-04
SACOL0933	SACOL0933	SACOL0933	-	hypothetical protein	130,37	7,03	-1,68	0,31	0,32	-5,28	1,26E-07	6,85E-07	-1,68	-0,32	0,65	-0,64	0,31	0,8	1,57	0,64	6,85E-07	2,69E-01	3,85E-02	3,49E-02
SACOL0934	dltX	SACOL0934 dltA dltB dltC dltD	-	DltX protein	423,00	8,72	-1,68	0,31	0,29	-5,82	5,78E-09	3,97E-08	-1,68	-0,46	0,79	-0,41	0,31	0,73	1,73	0,75	3,97E-08	1,66E-02	2,12E-03	9,49E-02
SACOL0599	capD	capD	CcpA	NAD dependent epimerase/dehydratase family protein	5973,83	12,54	-1,69	0,31	0,37	-4,58	4,65E-06	1,93E-05	-1,69	-1,09	0,54	-0,07	0,31	0,47	1,45	0,95	1,93E-05	5,51E-08	1,37E-01	8,47E-01
SACOL2574	ddh	ddh ywfG	CodY	D-isomer specific 2-hydroxyacid dehydrogenase family protein	322,40	8,33	-1,70	0,31	0,29	-5,83	5,4E-09	3,73E-08	-1,70	-0,45	0,88	-0,37	0,31	0,73	1,84	0,77	3,73E-08	6,77E-03	4,69E-04	6,71E-02
SACOL0430	metC	metEFCI	MgrA	bifunctional cystathionine gamma-lyase/gamma-synthase	57,88	5,86	-1,70	0,31	0,37	-4,57	4,79E-06	1,98E-05	-1,70	-0,23	0,4	-0,91	0,31	0,85	1,32	0,53	1,98E-05	5,43E-01	3,14E-01	4,84E-03
SACOL1726	rpmI	rplT rpmI infC	-	ribosomal protein L35	9829,06	13,26	-1,71	0,31	0,23	-7,39	1,47E-13	1,78E-12	-1,71	-0,52	1,39	0,2	0,31	0,7	2,62	1,15	1,78E-12	1,68E-04	8,40E-12	2,64E-01
SACOL1107	SACOL1107	SACOL1107 potA potB potC potD	-	Cro/CI family transcriptional regulator	728,18	9,51	-1,71	0,31	0,25	-6,92	4,4E-12	4,59E-11	-1,71	-1,21	0,91	0,4	0,31	0,43	1,88	1,32	4,59E-11	5,39E-10	6,26E-04	8,35E-02
SACOL2652	clfB	clfB	TraP	clumping factor B	62466,17	15,93	-1,71	0,31	0,26	-6,63	3,38E-11	3,23E-10	-1,71	-0,18	1,5	-0,02	0,31	0,88	2,83	0,99	3,23E-10	3,81E-01	2,02E-09	9,39E-01
SACOL2392	narI	nreA nreB nreC narI narJ narH narG	SigB	respiratory nitrate reductase subunit gamma	606,53	9,24	-1,71	0,31	0,27	-6,37	1,85E-10	1,60E-09	-1,71	-1,35	0,33	-0,03	0,31	0,39	1,26	0,98	1,60E-09	2,54E-15	2,11E-01	9,40E-01
SACOL2653	arcR	arcR	ArgR	Crp/FNR family transcriptional regulator	560,54	9,13	-1,72	0,30	0,25	-6,79	1,09E-11	1,10E-10	-1,72	-0,81	0,96	0,05	0,30	0,57	1,95	1,04	1,10E-10	5,57E-07	6,60E-05	8,73E-01
SACOL2338	SACOL2338	SACOL2338	-	hypothetical protein	495,92	8,95	-1,72	0,30	0,23	-7,41	1,3E-13	1,60E-12	-1,72	-1,03	-0,08	-0,75	0,30	0,49	0,95	0,59	1,60E-12	5,97E-11	7,70E-01	5,31E-06
SACOL1345	SACOL1345	SACOL1345	MgrA	hypothetical protein	505,53	8,98	-1,72	0,30	0,32	-5,32	1,06E-07	5,86E-07	-1,72	-0,51	0,37	-0,8	0,30	0,7	1,29	0,57	5,86E-07	8,60E-03	2,09E-01	1,41E-03
SACOL0285	SACOL0285	SACOL0285	-	hypothetical protein	234,87	7,88	-1,73	0,30	0,30	-5,73	1E-08	6,64E-08	-1,73	-1,01	0,65	-0,06	0,30	0,5	1,57	0,96	6,64E-08	8,76E-07	4,21E-03	8,88E-01
[illegible]	[illegible]	[illegible]	[illegible]	[illegible]	[illegible]	[illegible]	[illegible]	[illegible]	[illegible]	[illegible]	[illegible]	[illegible]	[illegible]	[illegible]	[illegible]	[illegible]	[illegible]	[illegible]	[illegible]	[illegible]	[illegible]	[illegible]	[illegible]	[illegible]

					WT MHQ vs control								WT MHQ/ WT co	ΔmhqR MHQ/ ΔmhqR co	ΔmhqR MHQ/ WT MHQ	ΔmhqR co/ WT co	WT MHQ/ WT co	ΔmhqR MHQ/ ΔmhqR co	ΔmhqR MHQ/ WT MHQ	ΔmhqR co/ WT co	WT MHQ/ WT co	ΔmhqR MHQ/ ΔmhqR co	ΔmhqR MHQ/ WT MHQ	ΔmhqR co/ WT co
Locus	Gene symbol	Operon	Regulon	Function	Base mean	A-value	M-value	Fold change	Standard error	Wald statistic	P-value	Adjusted P-value	M-value	M-value	M-value	M-value	Fold change	Fold change	Fold change	Fold change	Adj. P-value	Adj. P-value	Adj. P-value	Adj. P-value
SACOL0552	SACOL0552	SACOL0552	-	hypothetical protein	9674,92	13,24	0,39	1,31	0,19	1,98	0,047665	8,21E-02	0,39	0,36	-0,15	-0,13	1,31	1,28	0,9	0,91	8,21E-02	9,57E-03	4,79E-01	5,22E-01
SACOL1007	hbo	yjbM hbo	GraRS	globin family protein	1011,53	9,98	0,76	1,70	0,38	1,98	0,047662	8,21E-02	0,76	-0,35	-1,65	-0,55	1,70	0,78	0,32	0,68	8,21E-02	6,44E-02	4,73E-07	2,02E-02
SACOL2523	yvmA	yvmA SACOL2524	-	MFS family major facilitator transporter	106,18	6,73	0,66	1,58	0,33	1,98	0,0478	8,22E-02	0,66	0,51	0,42	0,5	1,58	1,42	1,34	1,41	8,22E-02	3,52E-02	1,38E-01	1,56E-01
SACOL1579	SACOL1579	SACOL1579	-	hypothetical protein	292,81	8,19	-0,51	0,70	0,26	-1,98	0,048048	8,26E-02	-0,51	-0,02	0,62	0,12	0,70	0,99	1,54	1,09	8,26E-02	9,04E-01	6,46E-03	6,51E-01
SACOL2663	manP	SACOL2662 manP pmi	-	PTS system, fructose-specific IIABC components	1521,63	10,57	-0,77	0,59	0,39	-1,97	0,048322	8,30E-02	-0,77	0,16	1,1	0,16	0,59	1,12	2,14	1,12	8,30E-02	3,92E-01	2,04E-04	6,58E-01
SACOL0803	SACOL0803	SACOL0803	-	hypothetical protein	391,26	8,61	-0,46	0,73	0,24	-1,97	0,049197	8,45E-02	-0,46	-0,17	0,07	-0,21	0,73	0,89	1,05	0,86	8,45E-02	3,85E-01	7,68E-01	4,28E-01
SACOL0610	sdrE	sdrE	GraRS	sdrE protein	2843,47	11,47	-0,57	0,68	0,29	-1,96	0,049696	8,53E-02	-0,57	-0,68	-0,04	0,06	0,68	0,62	0,97	1,04	8,53E-02	1,42E-05	8,98E-01	8,46E-01
SACOL1883	SACOL1883	SACOL1882 SACOL1883	-	hypothetical protein	1254,31	10,29	0,49	1,41	0,25	1,96	0,049757	8,53E-02	0,49	-0,1	-0,39	0,19	1,41	0,93	0,76	1,14	8,53E-02	6,05E-01	1,28E-01	3,99E-01
SACOL1815	SACOL1815	pldB SACOL1815	-	hypothetical protein	129,82	7,02	0,59	1,50	0,30	1,96	0,049839	8,54E-02	0,59	0,35	-0,7	-0,42	1,50	1,27	0,62	0,75	8,54E-02	2,37E-01	7,77E-03	2,64E-01
SACOL1825	SACOL1825	SACOL1825	-	N-acetylmuramoyl-L-alanine amidase	4506,80	12,14	0,41	1,33	0,21	1,96	0,049839	8,54E-02	0,41	0	-0,87	-0,46	1,33	1	0,55	0,73	8,54E-02	9,99E-01	2,00E-07	7,11E-03
SACOL0808	SACOL0808	pepT SACOL0807 SACOL0808	-	hypothetical protein	136,90	7,10	-0,63	0,64	0,32	-1,96	0,050159	8,59E-02	-0,63	-0,71	-0,37	-0,27	0,64	0,61	0,77	0,83	8,59E-02	6,87E-03	2,56E-01	4,64E-01
SACOL2040	yeaZ	gcp rimI yeaZ yjeE	-	glycoprotease	406,53	8,67	0,58	1,49	0,30	1,96	0,050193	8,59E-02	0,58	0,2	-0,54	-0,16	1,49	1,15	0,69	0,9	8,59E-02	3,80E-01	3,77E-02	6,51E-01
SACOL0530	SACOL0530	SACOL0529 SACOL0530 rsmI	MgrA	hypothetical protein	492,60	8,94	-0,60	0,66	0,30	-1,96	0,050344	8,61E-02	-0,60	0,09	1,05	0,35	0,66	1,06	2,07	1,27	8,61E-02	6,86E-01	7,75E-05	1,59E-01
SACOL2121	blt	blt	-	acetyltransferase	913,95	9,84	0,45	1,36	0,23	1,96	0,050366	8,61E-02	0,45	0,06	-0,5	-0,12	1,36	1,04	0,71	0,92	8,61E-02	7,34E-01	1,57E-02	6,34E-01
SACOL1763	SACOL1763	SACOL1763 thiI nifZ	TraP	hypothetical protein	828,25	9,69	-0,39	0,76	0,20	-1,96	0,050448	8,62E-02	-0,39	-0,44	0,25	0,28	0,76	0,74	1,19	1,21	8,62E-02	1,75E-03	1,67E-01	1,40E-01
SACOL0136	cap5A	cap5ABCDEFGHIJKLMNOP	SigB	capsular polysaccharide biosynthesis protein Cap5A	256,75	8,00	-0,80	0,57	0,41	-1,96	0,050578	8,63E-02	-0,80	0,43	0,73	-0,49	0,57	1,35	1,66	0,71	8,63E-02	2,20E-02	4,57E-02	3,22E-02
SACOL2415	gpmA	gpmA	-	phosphoglyceromutase	3216,98	11,65	-0,76	0,59	0,39	-1,96	0,050563	8,63E-02	-0,76	0,09	0,64	-0,22	0,59	1,06	1,56	0,86	8,63E-02	5,13E-01	6,65E-02	2,07E-01
SACOL2135	manA1	SACOL2134 manA1	-	mannose-6-phosphate isomerase, class I	1139,43	10,15	-0,93	0,53	0,48	-1,95	0,050608	8,63E-02	-0,93	0,26	0,74	-0,44	0,53	1,2	1,67	0,74	8,63E-02	1,10E-01	8,48E-02	3,16E-02
SACOL0994	oppF	SACOL0990 oppB oppC oppD oppF oppA	MgrA	oligopeptide ABC transporter, ATP-binding protein	1249,98	10,29	-0,52	0,70	0,27	-1,95	0,050734	8,64E-02	-0,52	-0,17	-0,1	-0,44	0,70	0,89	0,93	0,74	8,64E-02	4,34E-01	7,65E-01	3,14E-02
SACOL0750	SACOL0750	SACOL0750	-	anion transporter family protein	1190,43	10,22	-0,40	0,76	0,20	-1,95	0,051151	8,71E-02	-0,40	-0,7	0,18	0,46	0,76	0,62	1,13	1,38	8,71E-02	2,37E-07	3,76E-01	9,33E-03
SACOL0795	SACOL0795	-	-	hypothetical protein	16,76	4,07	1,17	2,24	0,60	1,95	0,051467	8,76E-02	1,17	0,91	0,35	0,25	2,24	1,88	1,27	1,19	8,76E-02	7,39E-02	4,56E-01	6,67E-01
SACOL2564	feoB	feoAB	Fur	FeoB family ferrous iron (Fe2+) uptake protein	579,26	9,18	0,66	1,58	0,34	1,94	0,052088	8,86E-02	0,66	0,29	0,56	0,9	1,58	1,22	1,47	1,87	8,86E-02	6,39E-02	6,12E-02	6,94E-07
[illegible]																								

					WT MHQ vs control								WT MHQ/ WT co	ΔmhqR MHQ/ ΔmhqR co	ΔmhqR MHQ/ WT MHQ	ΔmhqR co/ WT co	WT MHQ/ WT co	ΔmhqR MHQ/ ΔmhqR co	ΔmhqR MHQ/ WT MHQ	ΔmhqR co/ WT co	WT MHQ/ WT co	ΔmhqR MHQ/ ΔmhqR co	ΔmhqR MHQ/ WT MHQ	ΔmhqR co/ WT co
Locus	Gene symbol	Operon	Regulon	Function	Base mean	A-value	M-value	Fold change	Standard error	Wald statistic	P-value	Adjusted P-value	M-value	M-value	M-value	M-value	Fold change	Fold change	Fold change	Fold change	Adj. P-value	Adj. P-value	Adj. P-value	Adj. P-value
SACOL1239	vfrA	vfrA fakA	-	alkaline shock protein	2351,39	11,20	-0,29	0,82	0,21	-1,38	0,167636	2,39E-01	-0,29	-0,03	0,38	0,11	0,82	0,98	1,3	1,08	2,39E-01	8,68E-01	4,73E-02	7,12E-01
SACOL2171	iucB	iucB	Fur	possible Iuc family aerobactin synthesis protein	873,50	9,77	0,30	1,23	0,22	1,38	0,168097	2,40E-01	0,30	0,06	-0,37	-0,14	1,23	1,04	0,77	0,91	2,40E-01	7,27E-01	5,90E-02	5,29E-01
SACOL0126	phnE2	phnE1 phnE2 phnC phnD	GraRS	phosphonate ABC transporter, permease	34,06	5,09	-0,63	0,65	0,46	-1,38	0,168287	2,40E-01	-0,63	-0,06	-0,15	-0,54	0,65	0,96	0,9	0,69	2,40E-01	9,08E-01	7,82E-01	2,44E-01
SACOL1745	pykA	pyk pfkA	-	pyruvate kinase	29251,72	14,84	0,37	1,29	0,27	1,38	0,168281	2,40E-01	0,37	0,09	-0,32	-0,05	1,29	1,06	0,8	0,97	2,40E-01	5,34E-01	2,19E-01	8,00E-01
SACOL0010	azlC	azlC azlD	-	branched-chain amino acid permease	208,80	7,71	0,33	1,26	0,24	1,38	0,168231	2,40E-01	0,33	0,84	0,01	-0,47	1,26	1,79	1,01	0,72	2,40E-01	1,16E-04	9,71E-01	1,40E-01
SACOL0725	SACOL0725	SACOL0725 sarX	-	transcriptional regulator, AraC family	417,00	8,70	0,42	1,34	0,30	1,38	0,168332	2,40E-01	0,42	0,36	-0,02	0,02	1,34	1,28	0,99	1,01	2,40E-01	1,05E-01	9,50E-01	9,67E-01
SACOL2726	SACOL2726	vraD vraE SACOL2726	-	hypothetical protein	28,40	4,83	0,73	1,66	0,53	1,38	0,168145	2,40E-01	0,73	0,65	-0,19	-0,09	1,66	1,57	0,88	0,94	2,40E-01	1,77E-01	6,74E-01	8,99E-01
SACOL0090	SACOL0090	SACOL0090	-	integral membrane domain-containing protein	4,01	2,00	1,31	2,48	0,95	1,38	0,168555	2,40E-01	1,31	0,78	0,16	0	2,48	1,72	1,12	1	2,40E-01	2,90E-01	8,56E-01	0,00E+00
SACOL0041	ccrB	-	CodY	cassette chromosome recombinase B	383,09	8,58	0,41	1,33	0,30	1,37	0,170244	2,42E-01	0,41	0,15	-0,13	0,12	1,33	1,11	0,91	1,09	2,42E-01	4,58E-01	6,76E-01	7,10E-01
SACOL2680	SACOL2680	SACOL2680	-	hypothetical protein	16,90	4,08	-1,04	0,48	0,76	-1,37	0,17049	2,42E-01	-1,04	0,32	0,13	-0,63	0,48	1,25	1,09	0,65	2,42E-01	6,71E-01	8,87E-01	1,96E-01
SACOL2297	SACOL2297	SACOL2297	-	hypothetical protein	1456,90	10,51	-0,32	0,80	0,23	-1,37	0,171382	2,43E-01	-0,32	-0,27	0,44	0,38	0,80	0,83	1,36	1,3	2,43E-01	6,19E-02	3,98E-02	1,81E-02
SACOL0634	eutD	eutD lipL	-	phosphotransacetylase	9196,04	13,17	0,28	1,21	0,20	1,36	0,173065	2,46E-01	0,28	-0,19	-0,28	0,18	1,21	0,88	0,82	1,13	2,46E-01	2,08E-01	1,58E-01	4,02E-01
SACOL1691	recJ	apt recJ	-	single-stranded-DNA-specific exonuclease RecJ	2771,86	11,44	-0,46	0,73	0,34	-1,36	0,173484	2,46E-01	-0,46	0,1	0,78	0,22	0,73	1,07	1,72	1,16	2,46E-01	5,34E-01	7,85E-03	2,82E-01
SACOL1315	mutS	mutSL glpP	GraRS	DNA mismatch repair protein MutS	2796,73	11,45	0,28	1,21	0,20	1,36	0,173668	2,46E-01	0,28	0,33	-0,1	-0,16	1,21	1,26	0,93	0,9	2,46E-01	1,66E-02	6,41E-01	4,17E-01
SACOL1624	era	recO era cdd dgkA ybeY phoH	-	GTP-binding protein Era	8068,24	12,98	0,30	1,24	0,22	1,36	0,173723	2,46E-01	0,30	-0,24	-0,15	0,38	1,24	0,85	0,9	1,3	2,46E-01	1,18E-01	5,15E-01	4,96E-02
SACOL2325	hutR	hutR	-	LysR family transcriptional regulator	151,71	7,25	-0,80	0,57	0,59	-1,36	0,173697	2,46E-01	-0,80	-0,49	-0,7	-0,97	0,57	0,71	0,62	0,51	2,46E-01	1,25E-01	1,97E-01	2,63E-03
SACOL0133	SACOL0133	-	-	hypothetical protein	2,31	1,21	1,41	2,67	1,04	1,36	0,174329	2,47E-01	1,41	0,42	-0,69	0	2,67	1,34	0,62	1	2,47E-01	5,31E-01	4,38E-01	0,00E+00
SACOL0402	SACOL0402	ulaA SACOL0401 SACOL0402 SACOL0403	ArlR	PTS system, IIA component	66,15	6,05	0,62	1,54	0,46	1,36	0,17433	2,47E-01	0,62	-0,14	0,63	1,25	1,54	0,91	1,55	2,38	2,47E-01	6,24E-01	1,04E-01	7,21E-06
SACOL0420	SACOL0420	SACOL0420 SACOL0421 SACOL0422 SACOL0424	-	Cro/CI family transcriptional regulator	74,39	6,22	-0,46	0,73	0,34	-1,36	0,174243	2,47E-01	-0,46	-0,28	-0,15	-0,29	0,73	0,82	0,9	0,82	2,47E-01	3,87E-01	6,63E-01	4,84E-01
SACOL0153	SACOL0153	SACOL0152 SACOL0153	GraRS	hypothetical membrane protein	258,88	8,02	-0,42	0,75	0,31	-1,36	0,174478	2,47E-01	-0,42	-0,72	0,6	0,85	0,75	0,61	1,52	1,8	2,47E-01	2,88E-05	2,11E-02	6,37E-05
SACOL1127	SACOL1127	SACOL1127	-	hypothetical protein	425,72	8,73	-0,38	0,77	0,28	-1,35	0,175836	2,48E-01	-0,38	-0,79	-0,37	0,02	0,77	0,58	0,77	1,01	2,48E-01	2,02E-04	1,15E-01	9,59E-01
SACOL0680	mnhB	SACOL0678 mnhABCDEFG	TraP	CPA3 family monovalent cation (K+ or Na+):proton (H+) antiporter-3, MnhB subunit	166,70	7,38	0,57	1,49	0,42	1,35	0,177872	2,51E-01	0,57	1,4	0,79	-0,02	1,49	2,64	1,73	0,99	2,51E-01	8,48E-15	9,05E-03	9,64E-01
SACOL0497	SACOL0497	SACOL0497	-	hypothetical protein	122,95	6,94	-0,47	0,72	0,35	-1,35	0,177989	2,51E-01	-0,47	-0,57	-0,56	-0,42	0,72	0,67	0,68	0,75	2,51E-01	3,42E-02	8,51E-02	1,97E-01
SACOL0622	vraB	vraABC SACOL0624	VraSR	acetyl-CoA C-acetyltransferase	118,33	6,89	-0,47	0,72	0,35	-1,34	0,178665	2,52E-01	-0,47	0,04	0,83	0,29	0,72	1,03	1,78	1,22	2,52E-01	8,67E-01	2,77E-03	4,03E-01
SACOL1300	SACOL1300	SACOL1300 SACOL1301 pgsA	-	ACT domain-containing protein	5224,64	12,35	0,27	1,20	0,20	1,34	0,179093	2,52E-01	0,27	0,36	0,31	0,2	1,20	1,28	1,24	1,15	2,52E-01	3,12E-02	1,12E-01	4,17E-01
SACOL1466	SACOL1466	brx cvfC SACOL1466 SACOL1467	BSH	hypothetical protein	758,36	9,57	0,44	1,36	0,33	1,34	0,180734	2,55E-01	0,44	-0,37	-0,58	0,21	1,36	0,77	0,67	1,16	2,55E-01	1,32E-02	4,52E-02	3,75E-01
[illegible]	[illegible]	[illegible]	[illegible]	[illegible]	[illegible]	[illegible]	[illegible]	[illegible]	[illegible]	[illegible]	[illegible]	[illegible]	[illegible]	[illegible]	[illegible]	[illegible]	[illegible]	[illegible]	[illegible]	[illegible]	[illegible]	[illegible]	[illegible]	[illegible]

					WT MHQ vs control								WT MHQ/ WT co	ΔmhqR MHQ/ ΔmhqR co	ΔmhqR MHQ/ WT MHQ	ΔmhqR co/ WT co	WT MHQ/ WT co	ΔmhqR MHQ/ ΔmhqR co	ΔmhqR MHQ/ WT MHQ	ΔmhqR co/ WT co	WT MHQ/ WT co	ΔmhqR MHQ/ ΔmhqR co	ΔmhqR MHQ/ WT MHQ	ΔmhqR co/ WT co
Locus	Gene symbol	Operon	Regulon	Function	Base mean	A-value	M-value	Fold change	Standard error	Wald statistic	P-value	Adjusted P-value	M-value	M-value	M-value	M-value	Fold change	Fold change	Fold change	Fold change	Adj. P-value	Adj. P-value	Adj. P-value	Adj. P-value
SACOL1747	accA	accA accD	SarA	acetyl-CoA carboxylase carboxyltransferase subunit alpha	4221,22	12,04	0,18	1,13	0,23	0,77	0,441879	5,40E-01	0,18	0,05	0,13	0,25	1,13	1,04	1,09	1,19	5,40E-01	7,47E-01	5,72E-01	1,40E-01
SACOL1851	SACOL1851	SACOL1851	-	hypothetical protein	88,81	6,47	-0,34	0,79	0,44	-0,77	0,44184	5,40E-01	-0,34	0,12	-0,07	-0,48	0,79	1,09	0,95	0,72	5,40E-01	7,17E-01	8,82E-01	1,87E-01
SACOL1716	hemD	hemLBDCXA	MgrA	uroporphyrinogen-III synthase	2461,23	11,27	-0,19	0,88	0,24	-0,76	0,446351	5,45E-01	-0,19	0,52	0,88	0,16	0,88	1,43	1,84	1,12	5,45E-01	5,03E-04	2,38E-05	5,12E-01
SACOL1876	epiC	epiGEFPDCBA	SigB	lantibiotic epidermin biosynthesis protein EpiC	28,67	4,84	0,34	1,27	0,45	0,76	0,448076	5,47E-01	0,34	0,41	0,6	0,39	1,27	1,33	1,52	1,31	5,47E-01	3,17E-01	1,59E-01	4,21E-01
SACOL0224	glcC	SACOL0223 glcC	MgrA	PTS system, IIBC components	89,39	6,48	0,24	1,18	0,32	0,76	0,450067	5,49E-01	0,24	-0,15	-0,52	-0,13	1,18	0,9	0,7	0,91	5,49E-01	6,46E-01	7,95E-02	7,73E-01
SACOL1656	SACOL1656	SACOL1656	-	hypothetical protein	27,64	4,79	0,40	1,32	0,53	0,75	0,450385	5,49E-01	0,40	-0,87	-1,37	-0,04	1,32	0,55	0,39	0,97	5,49E-01	1,08E-01	4,72E-03	9,53E-01
SACOL1857	SACOL1857	SACOL1857	-	hypothetical protein	163,81	7,36	-0,25	0,84	0,34	-0,76	0,450241	5,49E-01	-0,25	0,1	0,64	0,24	0,84	1,07	1,56	1,18	5,49E-01	7,23E-01	2,81E-02	5,42E-01
SACOL2454	SACOL2454	SACOL2454	-	hypothetical protein	1,27	0,35	-0,80	0,58	1,05	-0,75	0,450411	5,49E-01	-0,80	1,05	1,37	0	0,58	2,07	2,58	1	5,49E-01	1,51E-01	9,69E-02	0,00E+00
SACOL1988	SACOL1988	SACOL1988	-	hypothetical protein	180,14	7,49	-0,31	0,81	0,41	-0,75	0,451998	5,50E-01	-0,31	-0,27	-0,26	-0,3	0,81	0,83	0,84	0,81	5,50E-01	2,36E-01	5,18E-01	3,16E-01
SACOL0877	gcvH	gcvH	-	glycine cleavage system protein H	4098,54	12,00	-0,16	0,89	0,22	-0,75	0,453495	5,52E-01	-0,16	-0,1	-0,2	-0,26	0,89	0,93	0,87	0,84	5,52E-01	5,65E-01	4,26E-01	1,34E-01
SACOL2182	lacF	lacGEFDCBA SACOL2187	MgrA	PTS lactose-N,N-diacetylchitobiose-beta-glucoside (lac) porter component IIA	3,18	1,67	0,71	1,64	0,95	0,75	0,455242	5,53E-01	0,71	0,22	0,53	0,33	1,64	1,16	1,44	1,26	5,53E-01	7,74E-01	4,90E-01	4,97E-01
SACOL1040	SACOL1040	SACOL1038 SACOL1039 SACOL1040	-	ABC transporter, ATP-binding protein	56,06	5,81	0,38	1,30	0,51	0,75	0,455101	5,53E-01	0,38	0,29	-0,07	0,01	1,30	1,22	0,95	1,01	5,53E-01	4,39E-01	8,91E-01	9,92E-01
SACOL1417	opp-2B	opp-2FDCB	GraRS	oligopeptide ABC transporter, membrane protein	55,71	5,80	0,27	1,21	0,36	0,75	0,455792	5,54E-01	0,27	-0,1	-0,2	0,14	1,21	0,93	0,87	1,1	5,54E-01	7,90E-01	5,89E-01	7,83E-01
SACOL1808	leuS	SACOL1807 leuS	-	leucyl-tRNA synthetase	3754,31	11,87	-0,14	0,91	0,19	-0,75	0,455978	5,54E-01	-0,14	-0,51	-0,12	0,23	0,91	0,7	0,92	1,17	5,54E-01	3,62E-04	5,60E-01	2,40E-01
SACOL1983	SACOL1983	SACOL1983	-	hypothetical protein	3,78	1,92	0,76	1,69	1,01	0,75	0,456008	5,54E-01	0,76	0,4	-0,03	0	1,69	1,32	0,98	1	5,54E-01	6,18E-01	9,77E-01	0,00E+00
SACOL1360	thrD	thrD	-	aspartate kinase	21,84	4,45	-0,39	0,76	0,52	-0,74	0,456314	5,54E-01	-0,39	-1,2	-0,39	0,35	0,76	0,44	0,76	1,27	5,54E-01	1,07E-02	5,36E-01	4,77E-01
SACOL2715	SACOL2715	SACOL2715 padR	-	hypothetical protein	352,28	8,46	0,19	1,14	0,25	0,74	0,456785	5,54E-01	0,19	0,19	0,18	0,15	1,14	1,14	1,13	1,11	5,54E-01	3,30E-01	4,21E-01	6,29E-01
SACOL1843	menC	menCE	-	o-succinylbenzoic acid (OSB) synthetase	647,97	9,34	0,17	1,12	0,22	0,74	0,457498	5,55E-01	0,17	-0,32	-0,23	0,24	1,12	0,8	0,85	1,18	5,55E-01	4,31E-02	2,65E-01	2,79E-01
SACOL0749	SACOL0749	SACOL0748 SACOL0749	MgrA	hypothetical membrane protein	590,42	9,21	0,19	1,14	0,26	0,74	0,458688	5,56E-01	0,19	0,03	0,08	0,22	1,14	1,02	1,06	1,16	5,56E-01	8,89E-01	7,37E-01	4,94E-01
SACOL1332	SACOL1332	SACOL1332	-	hypothetical protein	15,26	3,93	0,44	1,35	0,59	0,74	0,458602	5,56E-01	0,44	0,32	-0,5	-0,24	1,35	1,25	0,71	0,85	5,56E-01	6,40E-01	4,02E-01	6,85E-01
SACOL0730	SACOL0730	SACOL0730	-	hypothetical protein	1310,10	10,36	0,22	1,16	0,29	0,74	0,459511	5,56E-01	0,22	-0,19	-0,65	-0,24	1,16	0,88	0,64	0,85	5,56E-01	2,51E-01	1,13E-02	2,75E-01
SACOL1751	SACOL1751	dnaE SACOL1751	-	hypothetical protein	2635,50	11,36	0,21	1,15	0,28	0,74	0,461089	5,58E-01	0,21	-0,51	-0,26	0,44	1,15	0,7	0,84	1,36	5,58E-01	1,06E-04	3,13E-01	2,82E-02
[illegible]																								

					WT MHQ vs control								WT MHQ/ WT co	Δ*mhqR* MHQ/ Δ*mhqR* co	Δ*mhqR* MHQ/ WT MHQ	Δ*mhqR* co/ WT co	WT MHQ/ WT co	Δ*mhqR* MHQ/ Δ*mhqR* co	Δ*mhqR* MHQ/ WT MHQ	Δ*mhqR* co/ WT co	WT MHQ/ WT co	Δ*mhqR* MHQ/ Δ*mhqR* co	Δ*mhqR* MHQ/ WT MHQ	Δ*mhqR* co/ WT co
**Locus**	**Gene symbol**	**Operon**	**Regulon**	**Function**	**Base mean**	**A-value**	**M-value**	**Fold change**	**Standard error**	**Wald statistic**	**P-value**	**Adjusted P-value**	**M-value**	**M-value**	**M-value**	**M-value**	**Fold change**	**Fold change**	**Fold change**	**Fold change**	**Adj. P-value**	**Adj. P-value**	**Adj. P-value**	**Adj. P-value**
SACOL1744	SACOL1744	SACOL1744	-	hypothetical protein	16,53	4,05	0,16	1,12	0,61	0,26	0,795836	8,48E-01	0,16	0,66	0,18	-0,21	1,12	1,58	1,13	0,86	8,48E-01	2,25E-01	7,68E-01	7,20E-01
SACOL2691	icaB	icaADBC	GraRS	intercellular adhesion protein B	19,58	4,29	0,17	1,13	0,68	0,26	0,797279	8,49E-01	0,17	0,38	-0,29	-0,26	1,13	1,3	0,82	0,84	8,49E-01	5,18E-01	5,92E-01	6,49E-01
SACOL1203	ylmG	SACOL1200 ylmE sepF/ylmF ylmG ylmH SACOL1205	-	hypothetical protein	4789,23	12,23	-0,05	0,97	0,19	-0,25	0,799268	8,51E-01	-0,05	0,15	0,75	0,53	0,97	1,11	1,68	1,44	8,51E-01	2,74E-01	1,93E-06	7,14E-04
SACOL0097	sirC	sirABC	Fur	Siderophore staphylobactin ABC transporter, permease protein SirC	504,53	8,98	-0,09	0,94	0,34	-0,25	0,800154	8,51E-01	-0,09	0,59	0,81	0,13	0,94	1,51	1,75	1,09	8,51E-01	2,08E-04	6,47E-03	5,90E-01
SACOL1626	dgkA	recO era cdd dgkA ybeY phoH	-	diacylglycerol kinase	2232,75	11,12	0,05	1,04	0,20	0,25	0,80086	8,51E-01	0,05	-0,25	0,19	0,47	1,04	0,84	1,14	1,39	8,51E-01	6,14E-02	3,02E-01	1,86E-03
SACOL1844	menE	menCE	-	O-succinylbenzoic acid--CoA ligase	548,85	9,10	-0,06	0,96	0,23	-0,25	0,800584	8,51E-01	-0,06	-0,52	-0,46	-0,01	0,96	0,7	0,73	0,99	8,51E-01	7,53E-04	2,05E-02	9,64E-01
SACOL0231	SACOL0231	SACOL0231	-	hypothetical protein	0,68	-0,56	-0,23	0,85	0,94	-0,25	0,803665	8,54E-01	-0,23	-0,34	-0,32	0	0,85	0,79	0,8	1	8,54E-01	4,80E-01	5,70E-01	0,00E+00
SACOL0999	opp-4B	opp-4A opp-4D opp-4F opp-4B opp-4C	GraRS	oligopeptide ABC transporter, permease	30,78	4,94	0,12	1,09	0,49	0,25	0,804064	8,54E-01	0,12	0,03	0,16	0,18	1,09	1,02	1,12	1,13	8,54E-01	9,39E-01	7,45E-01	7,40E-01
SACOL0541	spoVG	spoVG	-	spoVG protein	12815,59	13,65	-0,07	0,95	0,28	-0,25	0,806137	8,56E-01	-0,07	0,5	1,06	0,49	0,95	1,41	2,08	1,4	8,56E-01	1,10E-04	8,14E-06	8,36E-04
SACOL0362	SACOL0362	SACOL0358 - SACOL0363	AgrA	hypothetical protein	84,73	6,40	-0,08	0,94	0,34	-0,24	0,806973	8,56E-01	-0,08	-0,37	0,27	0,48	0,94	0,77	1,21	1,39	8,56E-01	2,12E-01	4,53E-01	1,85E-01
SACOL0449	SACOL0449	SACOL0449	-	hypothetical protein	1380,38	10,43	0,05	1,04	0,22	0,24	0,809092	8,58E-01	0,05	0,28	0,42	0,19	1,04	1,21	1,34	1,14	8,58E-01	1,11E-01	5,55E-02	3,92E-01
SACOL0507	sle1	sle1	GraRS	possible LysM family autolysin	7651,09	12,90	-0,07	0,96	0,27	-0,24	0,809159	8,58E-01	-0,07	0,19	0,19	-0,07	0,96	1,14	1,14	0,95	8,58E-01	2,81E-01	5,15E-01	7,80E-01
SACOL0723	ssaA	ssaA	-	secretory antigen SsaA-like protein	5706,96	12,48	-0,08	0,95	0,32	-0,24	0,811668	8,60E-01	-0,08	0,37	0,45	0	0,95	1,29	1,37	1	8,60E-01	4,52E-02	1,42E-01	9,97E-01
SACOL0050	pls	pls	-	methicillin-resistant surface protein	122970,17	16,91	0,06	1,04	0,24	0,24	0,812757	8,61E-01	0,06	-0,16	-1,18	-0,92	1,04	0,9	0,44	0,53	8,61E-01	3,76E-01	1,35E-08	2,23E-06
SACOL0523	ldcC	ldcC tmk pstA	-	arginine/lysine/ornithine decarboxylase	428,86	8,74	0,06	1,04	0,27	0,23	0,814757	8,62E-01	0,06	0,41	0,01	-0,32	1,04	1,33	1,01	0,8	8,62E-01	3,98E-02	9,61E-01	2,63E-01
SACOL2378	SACOL2378	SACOL2378	-	transcriptional regulator, AraC family	1034,46	10,01	-0,05	0,97	0,19	-0,23	0,81469	8,62E-01	-0,05	-0,18	0,35	0,45	0,97	0,88	1,27	1,37	8,62E-01	3,91E-01	1,23E-01	5,26E-02
SACOL2358	hssR	hssRS lytT SACOL2361	GraRS	hemin DNA-binding response regulator	128,34	7,00	-0,07	0,95	0,31	-0,23	0,815514	8,63E-01	-0,07	-0,2	-0,35	-0,21	0,95	0,87	0,78	0,86	8,63E-01	4,70E-01	2,34E-01	5,54E-01
SACOL2690	icaD	icaADBC	GraRS	intercellular adhesion protein D	7,24	2,86	0,19	1,14	0,83	0,23	0,816892	8,64E-01	0,19	0,53	-0,46	-0,3	1,14	1,44	0,73	0,81	8,64E-01	4,95E-01	5,35E-01	5,32E-01
SACOL1926	mutY	mutY	-	A/G-specific adenine glycosylase	1382,71	10,43	0,07	1,05	0,29	0,23	0,816708	8,64E-01	0,07	0,54	0,48	0	1,05	1,45	1,39	1	8,64E-01	1,01E-03	6,51E-02	9,94E-01
SACOL1783	acsA	acs	CcpA	acetyl-CoA synthetase	493,04	8,95	-0,08	0,95	0,33	-0,23	0,817742	8,64E-01	-0,08	-0,69	-1	-0,39	0,95	0,62	0,5	0,76	8,64E-01	6,90E-05	4,70E-04	1,02E-01
SACOL1354	SACOL1354	SACOL1352 SACOL1353 SACOL1354 SACOL1355	-	sensor histidine kinase	510,57	9,00	0,05	1,03	0,21	0,23	0,820538	8,67E-01	0,05	-0,17	-0,19	0,02	1,03	0,89	0,88	1,01	8,67E-01	3,47E-01	3,68E-01	9,59E-01
SACOL2625	SACOL2625	SACOL2625	SigB	hypothetical protein	974,54	9,93	-0,09	0,94	0,38	-0,22	0,822028	8,68E-01	-0,09	-0,06	0,77	0,68	0,94	0,96	1,71	1,6	8,68E-01	7,81E-01	1,06E-02	1,76E-02
SACOL1807	SACOL1807	SACOL1807 leuS	-	rhodanese-like domain-containing protein	546,77	9,09	0,05	1,03	0,22	0,22	0,82283	8,68E-01	0,05	-0,46	-0,09	0,39	1,03	0,73	0,94	1,31	8,68E-01	6,09E-03	7,12E-01	7,34E-02
SACOL2616	panE	panE	-	2-dehydropantoate 2-reductase	575,03	9,17	-0,04	0,97	0,20	-0,22	0,82313	8,68E-01	-0,04	0,37	-0,11	-0,51	0,97	1,29	0,93	0,7	8,68E-01	2,07E-02	6,14E-01	5,61E-03
SACOL2681	SACOL2681	SACOL2681	SigB	hypothetical protein	959,81	9,91	0,09	1,07	0,41	0,22	0,823522	8,68E-01	0,09	0,77	0,36	-0,29	1,07	1,71	1,28	0,82	8,68E-01	5,53E-09	3,52E-01	1,68E-01
SACOL2417	SACOL2417	-	-	hypothetical protein	18,29	4,19	-0,13	0,92	0,58	-0,22	0,826588	8,71E-01	-0,13	0,33	0,08	-0,23	0,92	1,26	1,06	0,85	8,71E-01	5,65E-01	8,91E-01	7,04E-01
SACOL1027	SACOL1027	SACOL1027	-	hypothetical protein	2,91	1,54	0,22	1,16	1,02	0,22	0,828955	8,74E-01	0,22	0,3	0,56	0	1,16	1,23	1,47	1	8,74E-01	6,94E-01	5,10E-01	0,00E+00
SACOL1609	pbp3	pbp3	-	penicillin-binding protein 3	2665,18	11,38	0,05	1,04	0,24	0,21	0,831963	8,76E-01	0,05	-0,68	-0,85	-0,13	1,04	0,62	0,55	0,91	8,76E-01	3,72E-07	1,37E-05	5,88E-01
SACOL0520	dnaX	dnaX	-	DNA polymerase III subunits gamma and tau	6473,17	12,66	0,05	1,03	0,22	0,21	0,832787	8,77E-01	0,05	-0,11	0,11	0,25	1,03	0,93	1,08	1,19	8,77E-01	4,47E-01	6,27E-01	1,46E-01
SACOL0711	SACOL0711	SACOL0711	-	hypothetical protein	2773,36	11,44	-0,05	0,97	0,23	-0,21	0,83435	8,78E-01	-0,05	0,13	-0,31	-0,47	0,97	1,09	0,81	0,72	8,78E-01	4,41E-01	1,04E-01	3,22E-02
SACOL1577	SACOL1577	SACOL1575 SACOL1576 SACOL1577 SACOL1578	-	hypothetical protein	84,53	6,40	-0,07	0,95	0,32	-0,21	0,834114	8,78E-01	-0,07	-0,01	0,14	0,06	0,95	0,99	1,1	1,04	8,78E-01	9,87E-01	6,60E-01	8,88E-01
SACOL0087	SACOL0087	SACOL0087	-	hypothetical protein	7,03	2,81	0,18	1,13	0,84	0,21	0,83507	8,78E-01	0,18	0,84	0,12	-0,25	1,13	1,79	1,09	0,84	8,78E-01	2,36E-01	8,88E-01	6,20E-01
SACOL2701	hisC	hisI hisF hisA hisH hisB hisC hisD hisG hisZ	HisR	histidinol-phosphate aminotransferase	70,51	6,14	0,09	1,06	0,41	0,21	0,836109	8,79E-01	0,09	0,59	0,66	0,13	1,06	1,51	1,58	1,09	8,79E-01	4,42E-02	4,13E-02	7,93E-01
SACOL0538	ispE	ispE purR SACOL0540	-	4-diphosphocytidyl-2C-methyl-D-erythritol kinase	12210,12	13,58	-0,06	0,96	0,31	-0,20	0,838448	8,81E-01	-0,06	0,98	0,93	-0,1	0,96	1,97	1,91	0,93	8,81E-01	3,81E-07	6,36E-04	7,80E-01
SACOL2045	ilvC	ilvDBNC-leuABCD-ilvA2	CodY	ketol-acid reductoisomerase	524,75	9,04	0,10	1,07	0,51	0,20	0,839554	8,82E-01	0,10	0,49	-0,22	-0,65	1,07	1,4	0,86	0,64	8,82E-01	3,47E-01	7,24E-01	1,81E-03
SACOL0924	SACOL0924	SACOL0924 SACOL0925 SACOL0926	CodY	hypothetical protein	1129,28	10,14	-0,05	0,97	0,25	-0,20	0,841485	8,84E-01	-0,05	-0,04	-0,32	-0,31	0,97	0,97	0,8	0,81	8,84E-01	8,43E-01	2,18E-01	2,41E-01
SACOL1266	dprA	dprA topA	MgrA	DNA processing protein DprA	42,96	5,42	-0,09	0,94	0,43	-0,20	0,842836	8,85E-01	-0,09	0,43	0,37	-0,12	0,94	1,35	1,29	0,92	8,85E-01	2,97E-01	3,52E-01	8,47E-01
SACOL1415	opp-2D	opp-2FDCB	GraRS	oligopeptide ABC transporter, ATP-binding protein	89,47	6,48	0,07	1,05	0,34	0,20	0,845299	8,87E-01	0,07	0,09	0,12	0,08	1,05	1,06	1,09	1,06	8,87E-01	7,72E-01	7,45E-01	8,60E-01
SACOL2385	SACOL2385	SACOL2385	-	heat shock protein, Hsp20 family	1260,60	10,30	0,04	1,03	0,22	0,20	0,845264	8,87E-01	0,04	-0,65	-0,22	0,46	1,03	0,64	0,86	1,38	8,87E-01	3,16E-05	3,66E-01	1,12E-02
SACOL0532	SACOL0532	SACOL0532	-	hypothetical protein	0,86	-0,22	-0,19	0,87	1,02	-0,19	0,848065	8,89E-01	-0,19	0,18	-0,24	0	0,87	1,13	0,85	1	8,89E-01	6,41E-01	7,53E-01	0,00E+00
SACOL0606	SACOL0606	SACOL0606 azo1	GraRS	hydrolase	288,40	8,17	-0,08	0,95	0,40	-0,19	0,848214	8,89E-01	-0,08	1,49	1,38	-0,15	0,95	2,81	2,6	0,9	8,89E-01	1,28E-17	1,97E-05	6,59E-01
SACOL2485	SACOL2485	SACOL2485 SACOL2486	-	hypothetical protein	292,32	8,19	-0,04	0,97	0,23	-0,19	0,848732	8,89E-01	-0,04	0,06	-0,18	-0,27	0,97	1,04	0,88	0,83	8,89E-01	7,98E-01	4,11E-01	3,32E-01
SACOL2175	amaP	asp23 SACOL2174 amaP opuD2	SigB	hypothetical protein	12959,83	13,66	-0,07	0,95	0,37	-0,19	0,849763	8,90E-01	-0,07	-0,3	0,13	0,34	0,95	0,81	1,09	1,27	8,90E-01	6,30E-02	7,26E-01	1,67E-01
SACOL2046	leuA	ilvDBNC-leuABCD-ilvA2	CodY	2-isopropylmalate synthase	710,36	9,47	-0,09	0,94	0,47	-0,19	0,851361	8,91E-01	-0,09	0,5	-0,22	-0,87	0,94	1,41	0,86	0,55	8,91E-01	3,58E-01	7,12E-01	3,66E-05
SACOL1614	nfo	nfo cshB	-	endonuclease IV	4708,33	12,20	0,05	1,03	0,25	0,19	0,852303	8,92E-01	0,05	0,2	0,45	0,3	1,03	1,15	1,37	1,23	8,92E-01	1,59E-01	4,81E-02	7,81E-02
SACOL2437	bcr	bcr	-	MFS family major facilitator transporter, bicyclomycin:cation symporter	1933,24	10,92	0,04	1,03	0,21	0,18	0,854152	8,93E-01	0,04	0,43	0,55	0,14	1,03	1,35	1,46	1,1	8,93E-01	9,42E-03	1,04E-03	6,10E-01
SACOL2429	SACOL2429	SACOL2429	-	hypothetical protein	2,87	1,52	0,18	1,13	0,97	0,18	0,854112	8,93E-01	0,18	0,35	-0,08	0	1,13	1,27	0,95	1	8,93E-01	6,66E-01	9,36E-01	0,00E+00
SACOL1524	ypbB	recQ2 ypbB	-	hypothetical protein	1056,17	10,04	-0,05	0,97	0,26	-0,18	0,854677	8,94E-01	-0,05	-0,4	-0,57	-0,22	0,97	0,76	0,67	0,86	8,94E-01	1,52E-02	1,61E-02	3,77E-01
SACOL0620	proP	proP	VraSR	MFS family major facilitator transporter, proline/betaine:cation symporter	3771,51	11,88	0,04	1,03	0,24	0,18	0,859059	8,98E-01	0,04	0,46	0,65	0,21	1,03	1,38	1,57	1,16	8,98E-01	1,68E-04	1,07E-04	3,93E-01
SACOL0470	ssl3	ssl3	AgrA	superantigen-like protein 3	38,60	5,27	0,09	1,06	0,51	0,18	0,859363	8,98E-01	0,09	-0,26	-0,09	0,19	1,06	0,84	0,94	1,14	8,98E-01	5,42E-01	8,73E-01	7,32E-01
SACOL1373	SACOL1373	SACOL1373	-	conserved hypothetical protein	1842,39	10,85	-0,06	0,96	0,36	-0,18	0,860259	8,98E-01	-0,06	-0,99	-0,64	0,25	0,96	0,5	0,64	1,19	8,98E-01	4,34E-10	6,62E-02	1,73E-01
SACOL0056	SACOL0056	-	-	hypothetical protein	3,22	1,69	-0,16	0,89	0,94	-0,17	0,861548	8,99E-01	-0,16	0,7	0,05	0	0,89	1,62	1,04	1	8,99E-01	3,56E-01	9,60E-01	0,00E+00
SACOL1142	isdD	isdABCDEFG	Fur	hemoglobin iron ABC transporter, membrane component IsdD	79,73	6,32	0,08	1,06	0,46	0,17	0,861852	8,99E-01	0,08	-0,01	-0,25	-0,16	1,06	0,99	0,84	0,9	8,99E-01	9,71E-01	5,94E-01	7,25E-01
SACOL0058	SACOL0058	SACOL0058	-	conserved hypothetical protein	1195,06	10,22	-0,05	0,96	0,31	-0,17	0,86133	8,99E-01	-0,05	-0,05	-0,33	-0,33	0,96	0,97	0,8	0,8	8,99E-01	8,10E-01	3,04E-01	1,15E-01
SACOL2648	phoB	phoB	-	alkaline phosphatase	10,72	3,42	-0,12	0,92	0,68	-0,17	0,861814	8,99E-01	-0,12	0,07	-0,08	-0,15	0,92	1,05	0,95	0,9	8,99E-01	9,15E-01	9,11E-01	8,04E-01
SACOL1455	ctpA	ctpA	-	carboxyl-terminal protease	1659,62	10,70	-0,05	0,97	0,26	-0,17	0,862312	8,99E-01	-0,05	0,13	0,2	0,02	0,97	1,09	1,15	1,01	8,99E-01	4,18E-01	4,31E-01	9,30E-01
SACOL0361	rinB	-	-	hypothetical protein	64,87	6,02	0,07	1,05	0,40	0,17	0,865262	9,01E-01	0,07	-0,26	0,27	0,53	1,05	0,84	1,21	1,44	9,01E-01	3,62E-01	4,74E-01	1,34E-01
SACOL1718	hemX	hemLBDCXA	MgrA	possible cytochrome c assembly protein	4060,77	11,99	0,04	1,03	0,26	0,17	0,864939	9,01E-01	0,04	0,71	0,65	-0,02	1,03	1,64	1,57	0,99	9,01E-01	9,18E-09	2,83E-03	9,41E-01
SACOL1559	SACOL1559	SACOL1559	-	hypothetical protein	217,18	7,76	-0,04	0,97	0,24	-0,17	0,865462	9,01E-01	-0,04	0,25	0,4	0,1	0,97	1,19	1,32	1,07	9,01E-01	2,32E-01	5,32E-02	7,68E-01
SACOL0726	sarX	SACOL0725 sarX	GraRS	possible staphylococcal accessory protein X	84,89	6,41	0,06	1,04	0,33	0,17	0,866606	9,02E-01	0,06	0,26	0,74	0,46	1,04	1,2	1,67	1,38	9,02E-01	3,34E-01	7,26E-03	1,85E-01
SACOL0698	tagD	tagD	-	glycerol-3-phosphate cytidylyltransferase	633,46	9,31	0,04	1,03	0,23	0,17	0,868449	9,03E-01	0,04	-0,76	-0,72	0,06	1,03	0,59	0,61	1,04	9,03E-01	9,23E-07	9,31E-04	7,99E-01
SACOL0997	opp-4D	opp-4A opp-4D opp-4F opp-4B opp-4C	GraRS	oligopeptide ABC transporter, ATP-binding protein	17,70	4,15	0,09	1,06	0,55	0,17	0,868936	9,04E-01	0,09	0,29	-0,23	-0,26	1,06	1,22	0,85	0,84	9,04E-01	6,44E-01	6,98E-01	6,51E-01
SACOL2698	hisA	hisI hisF hisA hisH hisB hisC hisD hisG hisZ	HisR	1-(5-phosphoribosyl)-5-[(5- phosphoribosylamino)methylideneamino] imidazole-4-carboxamide isomerase	88,91	6,47	-0,06	0,96	0,35	-0,16	0,871869	9,06E-01	-0,06	-0,17	0,38	0,4	0,96	0,89	1,3	1,32	9,06E-01	6,30E-01	2,32E-01	3,53E-01
SACOL0230	gatB	blgG SACOL0229 gatB	-	phosphotransferase system galactitol-specific enzyme II, B component	10,60	3,41	0,12	1,08	0,72	0,16	0,871644	9,06E-01	0,12	-0,85	-0,44	0,33	1,08	0,55	0,74	1,26	9,06E-01	1,36E-01	5,21E-01	5,58E-01
SACOL2409	fmhA	fmhA	-	fmhA protein	85,27	6,41	0,06	1,04	0,38	0,16	0,873393	9,07E-01	0,06	-0,19	-0,65	-0,36	1,04	0,88	0,64	0,78	9,07E-01	5,91E-01	6,19E-02	3,81E-01
SACOL1858	SACOL1858	SACOL1858	-	hypothetical protein	74,61	6,22	-0,05	0,96	0,34	-0,16	0,874775	9,08E-01	-0,05	0,09	0,55	0,34	0,96	1,06	1,46	1,27	9,08E-01	7,94E-01	5,74E-02	4,17E-01
SACOL1707	radC	radC comC	-	DNA repair protein RadC	118,44	6,89	0,05	1,04	0,34	0,15	0,877063	9,10E-01	0,05	0,38	0,57	0,21	1,04	1,3	1,48	1,16	9,10E-01	7,96E-02	4,71E-02	5,37E-01
SACOL0881	trxA_3	SACOL0880 trxA_3	SigB	possible thioredoxin	377,40	8,56	0,04	1,02	0,23	0,15	0,877305	9,10E-01	0,04	-0,25	0	0,26	1,02	0,84	1	1,2	9,10E-01	1,63E-01	9,89E-01	2,83E-01
SACOL2462	SACOL2462	ybbM SACOL2462	SigB	ABC transporter, ATP-binding protein	850,48	9,73	0,06	1,04	0,38	0,16	0,876762	9,10E-01	0,06	0,36	0,41	0,11	1,04	1,28	1,33	1,08	9,10E-01	1,91E-02	2,51E-01	6,76E-01
SACOL2031	nrgA	nrgA	GlnR	AMT family ammonium or ammonia transporter	10,56	3,40	-0,10	0,93	0,66	-0,15	0,880378	9,12E-01	-0,10	0	0,32	0,12	0,93	1	1,25	1,09	9,12E-01	9,96E-01	6,09E-01	8,60E-01
SACOL0443	SACOL0443	SACOL0443	-	hypothetical protein	8,18	3,03	-0,11	0,93	0,73	-0,15	0,87988	9,12E-01	-0,11	-0,02	0,85	0,44	0,93	0,99	1,8	1,36	9,12E-01	9,77E-01	1,55E-01	4,15E-01
SACOL2683	msrA3	msrA3 SACOL2684	-	methionine sulfoxide reductase A	41,79	5,39	-0,06	0,96	0,41	-0,15	0,880258	9,12E-01	-0,06	0,08	-0,11	-0,2	0,96	1,06	0,93	0,87	9,12E-01	8,52E-01	8,04E-01	6,97E-01
SACOL2360	lytT	hssRS lytT SACOL2361	GraRS	LytTr DNA-binding region-containing protein	188,83	7,56	0,06	1,04	0,38	0,15	0,881959	9,13E-01	0,06	-0,71	0,99	1,56	1,04	0,61	1,99	2,95	9,13E-01	3,26E-03	2,40E-03	1,53E-08
SACOL2732	SACOL2732	SACOL2732	-	transcriptional regulator, putative	84,48	6,40	0,06	1,04	0,41	0,15	0,882239	9,13E-01	0,06	0,53	0,69	0,19	1,04	1,44	1,61	1,14	9,13E-01	8,71E-02	5,99E-02	6,94E-01
SACOL0848	SACOL0848	SACOL0848	-	hypothetical protein	47,51	5,57	-0,06	0,96	0,41	-0,15	0,883282	9,14E-01	-0,06	0,38	-0,3	-0,57	0,96	1,3	0,81	0,67	9,14E-01	3,65E-01	4,28E-01	2,06E-01
SACOL2460	SACOL2460	SACOL2460	-	drug transporter	971,94	9,92	-0,04	0,97	0,29	-0,14	0,885456	9,16E-01	-0,04	1,18	0,83	-0,37	0,97	2,27	1,78	0,77	9,16E-01	1,52E-19	7,37E-04	4,88E-02
SACOL2032	SACOL2032	-	-	hypothetical protein	1,65	0,72	0,15	1,11	1,04	0,14	0,885928	9,16E-01	0,15	0,58	0,04	0	1,11	1,49	1,03	1	9,16E-01	3,90E-01	9,66E-01	0,00E+00
SACOL0463	SACOL0463	SACOL0463	GraRS	hypothetical protein	45,69	5,51	0,06	1,04	0,43	0,14	0,886223	9,16E-01	0,06	-0,03	0,12	0,17	1,04	0,98	1,09	1,13	9,16E-01	9,43E-01	7,73E-01	7,32E-01
SACOL1513	hup	hup	ArlR	DNA-binding protein HU	83762,27	16,35	-0,04	0,97	0,27	-0,14	0,887441	9,17E-01	-0,04	-0,13	-0,06	0,03	0,97	0,91	0,96	1,02	9,17E-01	4,44E-01	8,71E-01	8,99E-01
SACOL2364	SACOL2364	SACOL2364	Rex	hypothetical protein	1560,76	10,61	0,03	1,02	0,23	0,14	0,887848	9,17E-01	0,03	-0,31	-0,47	-0,13	1,02	0,81	0,72	0,91	9,17E-01	2,01E-02	2,24E-02	4,89E-01
SACOL2513	SACOL2513	SACOL2513	-	hypothetical protein	1,64	0,71	-0,14	0,91	1,02	-0,14	0,888984	9,17E-01	-0,14	-0,54	-0,13	0	0,91	0,69	0,91	1	9,17E-01	4,82E-01	8,99E-01	0,00E+00
SACOL2049	leuD	ilvDBNC-leuABCD-ilvA2	CodY	3-isopropylmalate dehydratase small subunit	300,89	8,23	0,07	1,05	0,49	0,14	0,890525	9,19E-01	0,07	0,65	-0,23	-0,78	1,05	1,57	0,85	0,58	9,19E-01	5,49E-02	7,02E-01	7,63E-03
SACOL0681	mnhC	SACOL0678 mnhABCDEFG	TraP	CPA3 family monovalent cation (K+ or Na+):proton (H+) antiporter-3, MnhC subunit	117,08	6,87	0,05	1,04	0,40	0,14	0,891887	9,19E-01	0,05	1,11	0,85	-0,17	1,04	2,16	1,8	0,89	9,19E-01	2,66E-06	9,82E-03	6,94E-01
SACOL2276	SACOL2276	SACOL2276	-	inosine-uridine preferring nucleoside hydrolase	207,30	7,70	-0,04	0,97	0,32	-0,14	0,891436	9,19E-01	-0,04	0,38	0,66	0,22	0,97	1,3	1,58	1,16	9,19E-01	6,26E-02	1,13E-02	5,08E-01
SACOL2029	scrB	scrBK	ScrR	beta-fructofuranosidase	2127,10	11,05	-0,04	0,98	0,27	-0,14	0,892006	9,19E-01	-0,04	-0,89	-0,5	0,32	0,98	0,54	0,71	1,25	9,19E-01	2,32E-06	3,74E-02	2,71E-01
SACOL1322	SACOL1322	glpK glpD SACOL1322 miaA hfq	SarA	alpha/beta fold family hydrolase	464,61	8,86	0,04	1,03	0,30	0,13	0,897634	9,24E-01	0,04	-0,03	-0,87	-0,77	1,03	0,98	0,55	0,59	9,24E-01	8,82E-01	1,30E-03	3,04E-04
SACOL1389	parE	parE parC	-	DNA topoisomerase IV subunit B	5281,67	12,37	-0,02	0,98	0,18	-0,13	0,898312	9,24E-01	-0,02	-0,24	0,09	0,3	0,98	0,85	1,06	1,23	9,24E-01	8,80E-02	6,67E-01	3,57E-02
SACOL1544	SACOL1544	SACOL1544 SACOL1545	-	hypothetical protein	47,88	5,58	0,05	1,04	0,43	0,13	0,898317	9,24E-01	0,05	0,57	0,1	-0,3	1,04	1,48	1,07	0,81	9,24E-01	1,69E-01	8,06E-01	5,83E-01
SACOL2423	bioX	bioX	MgrA	hypothetical protein	45,13	5,50	-0,05	0,97	0,38	-0,13	0,897312	9,24E-01	-0,05	0,18	-0,05	-0,23	0,97	1,13	0,97	0,85	9,24E-01	6,75E-01	9,17E-01	6,62E-01
SACOL2536	supH	supH	-	haloacid dehalogenase-like hydrolase	194,37	7,60	-0,03	0,98	0,27	-0,13	0,898345	9,24E-01	-0,03	0,04	0,97	0,81	0,98	1,03	1,96	1,75	9,24E-01	8,62E-01	8,63E-06	1,93E-03
SACOL0164	ausA	ausA acpS	-	gramicidin S synthetase 2 related protein	9906,65	13,27	-0,03	0,98	0,23	-0,13	0,899087	9,24E-01	-0,03	0,25	-0,01	-0,29	0,98	1,19	0,99	0,82	9,24E-01	8,54E-02	9,57E-01	1,42E-01
SACOL0011	azlD	azlC azlD	-	Branched-chain amino acid transport protein, AzlD	147,86	7,21	0,03	1,02	0,26	0,12	0,902904	9,28E-01	0,03	0,45	0,02	-0,36	1,02	1,37	1,01	0,78	9,28E-01	5,40E-02	9,31E-01	2,59E-01
SACOL2497	SACOL2497	SACOL2496 SACOL2497	-	staphylococcus tandem lipoprotein	374,07	8,55	-0,03	0,98	0,26	-0,12	0,903758	9,28E-01	-0,03	0,14	0,11	-0,06	0,98	1,1	1,08	0,96	9,28E-01	5,27E-01	6,89E-01	8,60E-01
SACOL1363	thrC	hom thrC thrB	CodY	threonine synthase	454,38	8,83	-0,03	0,98	0,25	-0,12	0,904231	9,28E-01	-0,03	-0,26	0,19	0,39	0,98	0,84	1,14	1,31	9,28E-01	1,37E-01	4,32E-01	7,94E-02
SACOL2047	leuB	ilvDBNC-leuABCD-ilvA2	CodY	3-isopropylmalate dehydrogenase	522,62	9,03	-0,06	0,96	0,50	-0,12	0,904531	9,28E-01	-0,06	0,44	-0,35	-0,89	0,96	1,36	0,78	0,54	9,28E-01	3,96E-01	5,54E-01	5,43E-05
SACOL2495	SACOL2495	SACOL2494 SACOL2495	-	hypothetical protein	177,62	7,47	-0,03	0,98	0,29	-0,12	0,906238	9,30E-01	-0,03	0,28	0,2	-0,11	0,98	1,21	1,15	0,93	9,30E-01	2,38E-01	4,81E-01	7,70E-01
SACOL2427	bioA	bioWFBAD	SigB	adenosylmethionine--8-amino-7-oxononanoate transaminase	17,88	4,16	-0,06	0,96	0,55	-0,12	0,907563	9,31E-01	-0,06	0,12	0,28	0,05	0,96	1,09	1,21	1,04	9,31E-01	8,30E-01	6,20E-01	9,39E-01
SACOL2048	leuC	ilvDBNC-leuABCD-ilvA2	CodY	3-isopropylmalate dehydratase large subunit	700,99	9,45	0,06	1,04	0,50	0,12	0,908338	9,31E-01	0,06	0,5	-0,26	-0,77	1,04	1,41	0,84	0,59	9,31E-01	3,55E-01	6,67E-01	5,01E-04
SACOL1416	opp-2C	opp-2FDCB	GraRS	oligopeptide ABC transporter, membrane protein	69,35	6,12	0,04	1,03	0,36	0,11	0,913376	9,36E-01	0,04	0,02	-0,05	-0,03	1,03	1,01	0,97	0,98	9,36E-01	9,70E-01	8,99E-01	9,59E-01
SACOL1183	arcD1	arcD1	SigB	arginine/ornithine APC family amino acid-polyamine-organocation antiporter	318,11	8,31	-0,03	0,98	0,24	-0,11	0,914462	9,36E-01	-0,03	0,37	0,5	0,08	0,98	1,29	1,41	1,06	9,36E-01	3,85E-02	1,73E-02	7,94E-01
SACOL1454	SACOL1454	SACOL1454	-	acetyltransferase (GNAT) family protein	285,79	8,16	0,03	1,02	0,30	0,11	0,914209	9,36E-01	0,03	-0,05	0,12	0,18	1,02	0,97	1,09	1,13	9,36E-01	8,21E-01	7,11E-01	5,52E-01
SACOL0123	deoC1	deoC1 deoB	GraRS	deoxyribose-phosphate aldolase	1645,62	10,68	0,03	1,02	0,32	0,10	0,920196	9,42E-01	0,03	-0,1	0,16	0,29	1,02	0,93	1,12	1,22	9,42E-01	5,34E-01	6,20E-01	1,88E-01
SACOL1892	ecsB	ecsBA	MgrA	ABC transporter, permease	1847,08	10,85	-0,02	0,99	0,21	-0,10	0,921774	9,42E-01	-0,02	0,17	0,05	-0,15	0,99	1,13	1,04	0,9	9,42E-01	2,92E-01	8,43E-01	5,37E-01
SACOL0602	SACOL0602	SACOL0602	-	HAD superfamily hydrolase	460,51	8,85	0,03	1,02	0,34	0,10	0,921528	9,42E-01	0,03	0,43	0,13	-0,27	1,02	1,35	1,09	0,83	9,42E-01	1,19E-02	7,29E-01	2,25E-01
SACOL1658	SACOL1658	SACOL1658	-	hypothetical protein	207,10	7,69	-0,03	0,98	0,30	-0,10	0,921919	9,42E-01	-0,03	-0,47	-0,13	0,28	0,98	0,72	0,91	1,21	9,42E-01	1,61E-02	6,75E-01	3,14E-01
SACOL1620	yqfL	dnaG yqfL SACOL1621	-	Putative pyruvate, phosphate dikinase regulatory protein	1794,51	10,81	-0,02	0,98	0,25	-0,10	0,923213	9,43E-01	-0,02	-0,59	-0,46	0,09	0,98	0,66	0,73	1,06	9,43E-01	1,40E-05	2,55E-02	7,10E-01
SACOL0232	gatC	gatC gutB SACOL0234 gatD	-	PTS system galactitol-specific IIC component	51,50	5,69	0,04	1,03	0,40	0,10	0,923226	9,43E-01	0,04	-0,2	0,11	0,27	1,03	0,87	1,08	1,21	9,43E-01	5,74E-01	7,93E-01	5,40E-01
SACOL1910	SACOL1910	SACOL1910	-	hypothetical protein	1,69	0,76	0,10	1,07	1,05	0,10	0,92335	9,43E-01	0,10	-0,29	0,39	0	1,07	0,82	1,31	1	9,43E-01	7,22E-01	6,80E-01	0,00E+00
SACOL0421	SACOL0421	SACOL0420 SACOL0421 SACOL0422 SACOL0424	-	hypothetical protein	247,52	7,95	-0,02	0,98	0,24	-0,10	0,92412	9,43E-01	-0,02	-0,12	-0,24	-0,14	0,98	0,92	0,85	0,91	9,43E-01	5,99E-01	3,11E-01	6,75E-01
SACOL1143	isdE	isdABCDEFG	Fur	hemoglobin iron ABC transporter, lipoprotein IsdE	73,57	6,20	0,04	1,03	0,46	0,09	0,931119	9,50E-01	0,04	0,18	0,35	0,19	1,03	1,13	1,27	1,14	9,50E-01	5,58E-01	4,10E-01	6,46E-01
SACOL0359	SACOL0359	SACOL0358 - SACOL0363	-	hypothetical protein	78,79	6,30	0,03	1,02	0,34	0,09	0,931116	9,50E-01	0,03	-0,2	0,12	0,29	1,02	0,87	1,09	1,22	9,50E-01	5,35E-01	7,41E-01	4,87E-01
SACOL0352	SACOL0352	SACOL0352 - SACOL0356 dut	-	hypothetical protein	64,17	6,00	-0,03	0,98	0,35	-0,09	0,931589	9,50E-01	-0,03	-0,45	0,4	0,73	0,98	0,73	1,32	1,66	9,50E-01	9,44E-02	2,23E-01	1,48E-02
SACOL2422	hlgB	hlgCB	SaeRS	gamma hemolysin component B	253,86	7,99	-0,04	0,97	0,46	-0,08	0,93243	9,50E-01	-0,04	-1,39	-1,81	-0,45	0,97	0,38	0,29	0,73	9,50E-01	1,60E-08	1,19E-06	2,32E-01
SACOL0543	glmU	SACOL0542 glmU	-	UDP-N-acetylglucosamine pyrophosphorylase	7500,91	12,87	-0,02	0,99	0,19	-0,08	0,936221	9,54E-01	-0,02	0,34	0,78	0,4	0,99	1,27	1,72	1,32	9,54E-01	3,24E-02	7,27E-07	6,46E-02
SACOL0864	SACOL0864	SACOL0864	-	hypothetical protein	150,15	7,23	0,03	1,02	0,36	0,08	0,93814	9,55E-01	0,03	0,39	0,71	0,29	1,02	1,31	1,64	1,22	9,55E-01	5,57E-02	5,48E-03	4,36E-01
SACOL1336	SACOL1336	SACOL1336	-	hypothetical protein	4,12	2,04	0,07	1,05	0,98	0,07	0,941756	9,59E-01	0,07	0,27	0,01	-0,08	1,05	1,21	1,01	0,95	9,59E-01	7,37E-01	9,90E-01	8,84E-01
SACOL0559	folB	folP folB folK	-	dihydroneopterin aldolase	773,27	9,59	0,01	1,01	0,20	0,07	0,942223	9,59E-01	0,01	0,15	0,13	-0,01	1,01	1,11	1,09	0,99	9,59E-01	4,17E-01	5,34E-01	9,72E-01
SACOL0356	SACOL0356	SACOL0352 - SACOL0356 dut	-	conserved hypothetical protein	80,92	6,34	0,02	1,02	0,32	0,07	0,943532	9,60E-01	0,02	-0,14	0,45	0,49	1,02	0,91	1,37	1,4	9,60E-01	6,74E-01	1,09E-01	2,31E-01
SACOL0017	SACOL0017	SACOL0017	-	hypothetical protein	70,76	6,14	0,02	1,02	0,33	0,07	0,945817	9,62E-01	0,02	0,81	0,37	-0,37	1,02	1,75	1,29	0,77	9,62E-01	7,19E-03	2,81E-01	3,55E-01
SACOL1715	hemB	hemLBDCXA	MgrA	porphobilinogen synthase	4668,93	12,19	-0,02	0,99	0,24	-0,07	0,947003	9,62E-01	-0,02	0,41	0,63	0,2	0,99	1,33	1,55	1,15	9,62E-01	2,08E-03	2,30E-03	2,77E-01
SACOL1297	SACOL1297	ftsK SACOL1296 SACOL1297 SACOL1298 phbB	AgrA	hypothetical protein	842,70	9,72	-0,02	0,99	0,24	-0,07	0,947035	9,62E-01	-0,02	0,41	0,65	0,2	0,99	1,33	1,57	1,15	9,62E-01	4,25E-02	3,91E-03	5,14E-01
SACOL1371	guaC	guaC	-	GMP reductase	1075,80	10,07	-0,03	0,98	0,39	-0,07	0,947595	9,62E-01	-0,03	-1,13	-0,59	0,47	0,98	0,46	0,66	1,39	9,62E-01	6,67E-12	1,19E-01	2,03E-03
SACOL0827	maa	hprK lgt maa SACOL0828	GraRS	putative acetyltransferase	854,89	9,74	0,01	1,01	0,21	0,06	0,950568	9,65E-01	0,01	-0,18	-0,11	0,07	1,01	0,88	0,93	1,05	9,65E-01	2,59E-01	5,97E-01	7,80E-01
SACOL1186	psmß1	psmß1_2	TraP	antibacterial protein	10,15	3,34	-0,04	0,97	0,69	-0,06	0,951142	9,65E-01	-0,04	0,21	-0,07	-0,18	0,97	1,16	0,95	0,88	9,65E-01	7,67E-01	9,26E-01	7,71E-01
SACOL1235	cfxE	rsgA cfxE thiN	-	ribulose-phosphate 3-epimerase (cfxE/rpe)	1473,07	10,52	0,01	1,01	0,23	0,06	0,950807	9,65E-01	0,01	-0,46	-0,19	0,27	1,01	0,73	0,88	1,21	9,65E-01	1,52E-03	3,91E-01	1,92E-01
SACOL1471	rnhA	rnhA	ArlR	cell wall enzyme EbsB-like protein	6,58	2,72	-0,05	0,97	0,82	-0,06	0,951034	9,65E-01	-0,05	0,82	0,58	-0,16	0,97	1,77	1,49	0,9	9,65E-01	2,27E-01	4,50E-01	7,81E-01
SACOL1199	ftsZ	ftsA ftsZ	-	cell division protein FtsZ	23581,24	14,53	0,01	1,01	0,21	0,06	0,952651	9,65E-01	0,01	-0,04	-0,21	-0,17	1,01	0,97	0,86	0,89	9,65E-01	8,01E-01	3,10E-01	3,12E-01
SACOL0040	SACOL0040	SACOL0037 SACOL0038 SACOL0039 SACOL0040	SigB	hypothetical protein	11,43	3,51	-0,04	0,97	0,67	-0,06	0,95264	9,65E-01	-0,04	0,32	0,38	0	0,97	1,25	1,3	1	9,65E-01	5,91E-01	5,19E-01	1,00E+00
SACOL2428	bioD	bioWFBAD	SigB	dethiobiotin synthase	11,96	3,58	0,04	1,03	0,75	0,06	0,953665	9,66E-01	0,04	0,05	-0,28	-0,16	1,03	1,04	0,82	0,9	9,66E-01	9,50E-01	7,26E-01	7,94E-01
SACOL0009	serS	serS	-	seryl-tRNA synthetase	1964,49	10,94	-0,01	0,99	0,25	-0,06	0,954382	9,67E-01	-0,01	0,26	0,69	0,37	0,99	1,2	1,61	1,29	9,67E-01	2,29E-01	2,46E-03	1,87E-01
SACOL0174	SACOL0174	SACOL0174	-	hypothetical protein	4,56	2,19	-0,05	0,97	0,87	-0,05	0,956221	9,68E-01	-0,05	0,87	0,51	0	0,97	1,83	1,42	1	9,68E-01	2,33E-01	5,00E-01	0,00E+00
SACOL2050	ilvA2	ilvDBNC-leuABCD-ilvA2	CodY	threonine ammonia-lyase	582,95	9,19	-0,03	0,98	0,48	-0,05	0,957493	9,69E-01	-0,03	0,59	-0,08	-0,75	0,98	1,51	0,95	0,59	9,69E-01	2,80E-01	8,99E-01	2,63E-03
SACOL1753	uspA2	uspA2	-	universal stress protein	5389,77	12,40	-0,01	0,99	0,25	-0,05	0,957512	9,69E-01	-0,01	-0,36	-0,25	0,09	0,99	0,78	0,84	1,06	9,69E-01	9,33E-03	3,39E-01	6,62E-01
SACOL1149	pheT	pheS pheT	-	phenylalanyl-tRNA synthetase subunit beta	9618,10	13,23	-0,01	0,99	0,22	-0,05	0,958623	9,69E-01	-0,01	-0,04	-0,03	0	0,99	0,97	0,98	1	9,69E-01	7,87E-01	9,13E-01	9,99E-01
SACOL2548	SACOL2548	SACOL2548	-	hypothetical protein	226,25	7,82	0,02	1,01	0,33	0,05	0,95978	9,70E-01	0,02	0,43	-0,15	-0,54	1,01	1,35	0,9	0,69	9,70E-01	8,58E-02	6,92E-01	5,47E-02
SACOL1694	tgt	yajC tgt queA ruvB ruvA thrR obgE	-	queuine tRNA-ribosyltransferase	10090,60	13,30	-0,01	0,99	0,21	-0,05	0,961495	9,71E-01	-0,01	0,25	0,23	-0,04	0,99	1,19	1,17	0,97	9,71E-01	7,55E-02	2,69E-01	8,84E-01
SACOL0360	SACOL0360	SACOL0358 - SACOL0363	-	hypothetical protein	90,94	6,51	-0,01	0,99	0,31	-0,05	0,963722	9,73E-01	-0,01	-0,23	0,19	0,36	0,99	0,85	1,14	1,28	9,73E-01	4,20E-01	6,00E-01	2,77E-01
SACOL1330	SACOL1330	-	-	hypothetical protein	37,97	5,25	-0,02	0,99	0,45	-0,04	0,964548	9,74E-01	-0,02	0	0,33	0,24	0,99	1	1,26	1,18	9,74E-01	9,95E-01	3,98E-01	6,25E-01
SACOL2501	pgcA	pgcA	-	phosphoglucomutase/phosphomannomutase	405,82	8,66	-0,01	0,99	0,30	-0,04	0,967589	9,76E-01	-0,01	-0,41	-0,93	-0,5	0,99	0,75	0,52	0,71	9,76E-01	1,11E-01	1,47E-03	9,26E-02
SACOL0216	prsS	prsS	-	hypothetical protein	230,85	7,85	0,01	1,01	0,26	0,04	0,9685	9,77E-01	0,01	0,31	-0,63	-0,88	1,01	1,24	0,65	0,54	9,77E-01	2,38E-01	1,80E-02	3,58E-04
SACOL1703	mreD	mreD mreC	-	rod shape-determining protein MreD	675,65	9,40	-0,01	0,99	0,26	-0,04	0,970275	9,78E-01	-0,01	-0,42	-0,24	0,15	0,99	0,75	0,85	1,11	9,78E-01	8,18E-03	3,46E-01	5,53E-01
SACOL1340	SACOL1340	SACOL1340	-	hypothetical protein	2,10	1,07	0,04	1,03	1,03	0,04	0,971506	9,79E-01	0,04	-0,7	-0,24	0	1,03	0,62	0,85	1	9,79E-01	3,62E-01	8,04E-01	0,00E+00
SACOL0473	ssl9	ssl9	SaeRS	staphylococcal exotoxin	56,57	5,82	-0,01	0,99	0,42	-0,03	0,972146	9,80E-01	-0,01	-0,28	-0,04	0,18	0,99	0,82	0,97	1,13	9,80E-01	4,19E-01	9,31E-01	7,12E-01
SACOL0248	lrgB	lrgA lrgB	CidR	murein hydrolase regulator LrgB	3404,69	11,73	0,02	1,01	0,48	0,03	0,974118	9,81E-01	0,02	-1,59	-1,18	0,22	1,01	0,33	0,44	1,16	9,81E-01	8,55E-17	9,04E-06	7,12E-01
SACOL0028	tnp	tnp	-	IS431mec, transposase	66,89	6,06	0,01	1,01	0,36	0,03	0,973783	9,81E-01	0,01	0,38	0,38	0	1,01	1,3	1,3	1	9,81E-01	2,24E-01	2,80E-01	1,00E+00
SACOL0364	SACOL0364	SACOL0364	-	prophage L54a, transcriptional regulator, RinA family	249,38	7,96	-0,01	0,99	0,23	-0,03	0,974171	9,81E-01	-0,01	-0,05	0,21	0,23	0,99	0,97	1,16	1,17	9,81E-01	8,27E-01	4,18E-01	4,15E-01
SACOL0055	SACOL0055	-	-	hypothetical protein	20,33	4,35	0,02	1,01	0,61	0,03	0,974774	9,81E-01	0,02	0,24	-0,34	-0,39	1,01	1,18	0,79	0,76	9,81E-01	6,98E-01	6,05E-01	4,80E-01
SACOL1356	SACOL1356	SACOL1356	-	hypothetical protein	10,73	3,42	0,02	1,01	0,72	0,03	0,976428	9,82E-01	0,02	-0,88	-0,29	0,39	1,01	0,54	0,82	1,31	9,82E-01	1,14E-01	6,86E-01	4,85E-01
SACOL1717	hemC	hemLBDCXA	MgrA	hydroxymethylbilane synthase	4735,50	12,21	-0,01	1,00	0,28	-0,03	0,979727	9,85E-01	-0,01	0,75	0,74	-0,02	1,00	1,68	1,67	0,99	9,85E-01	1,26E-08	1,82E-03	9,39E-01
SACOL1709	folC	folC valS	-	folylpolyglutamate synthase/dihydrofolate synthase	6409,06	12,65	0,00	1,00	0,19	-0,02	0,980626	9,86E-01	0,00	0,35	0,6	0,23	1,00	1,27	1,52	1,17	9,86E-01	9,20E-03	1,73E-04	2,07E-01
SACOL2060	alr	alr acpS ydbS ydbS SACOL2064	-	alanine racemase	3798,28	11,89	0,00	1,00	0,22	-0,02	0,982559	9,87E-01	0,00	-0,04	0,04	0,07	1,00	0,97	1,03	1,05	9,87E-01	8,04E-01	8,69E-01	7,56E-01
SACOL0693	tagA	tagA	-	teichoic acid biosynthesis protein	390,43	8,61	-0,01	0,99	0,40	-0,02	0,984019	9,88E-01	-0,01	0,83	0,33	-0,46	0,99	1,78	1,26	0,73	9,88E-01	5,00E-05	3,87E-01	1,36E-01
SACOL0349	SACOL0349	SACOL0335 - SACOL0338 ssb1 SACOL0340 - SACOL0349	-	conserved hypothetical protein	67,88	6,08	0,01	1,00	0,34	0,02	0,987843	9,91E-01	0,01	-0,07	0,89	0,83	1,00	0,95	1,85	1,78	9,91E-01	8,06E-01	7,96E-04	1,01E-02
SACOL0212	fadB	fadAB	CcpA	3-hydroxyacyl-CoA dehydrogenase	47,28	5,56	-0,01	1,00	0,39	-0,01	0,988284	9,91E-01	-0,01	0,02	0,08	0,04	1,00	1,01	1,06	1,03	9,91E-01	9,62E-01	8,57E-01	9,46E-01
SACOL1748	accD	accA accD	SarA	acetyl-CoA carboxylase subunit beta	2978,19	11,54	0,00	1,00	0,22	0,01	0,989342	9,92E-01	0,00	0,01	0,23	0,21	1,00	1,01	1,17	1,16	9,92E-01	9,46E-01	2,64E-01	2,18E-01
SACOL0472	ssl4	ssl4	MgrA	superantigen-like protein	12,18	3,61	0,01	1,01	0,64	0,01	0,989968	9,92E-01	0,01	-0,53	-0,46	0,06	1,01	0,69	0,73	1,04	9,92E-01	3,84E-01	4,65E-01	9,37E-01
SACOL1598	comGD	comGD comGC comGB comGA	MgrA	competence protein ComGD	35,33	5,14	-0,01	1,00	0,48	-0,01	0,990537	9,93E-01	-0,01	0,24	0,75	0,4	1,00	1,18	1,68	1,32	9,93E-01	5,27E-01	4,91E-02	3,97E-01
SACOL0729	SACOL0729	-	-	hypothetical protein	54,18	5,76	0,00	1,00	0,37	0,01	0,994851	9,97E-01	0,00	-0,35	-0,58	-0,18	1,00	0,78	0,67	0,88	9,97E-01	3,55E-01	7,81E-02	7,23E-01
SACOL2167	htsA	htsABC	Fur	heme ABC transporter, heme-binding protein HtsA	1897,26	10,89	0,00	1,00	0,37	0,00	0,996253	9,98E-01	0,00	0,1	0,85	0,74	1,00	1,07	1,8	1,67	9,98E-01	4,99E-01	6,38E-03	2,50E-05
SACOL0525	pstA	ldcC tmk pstA	-	hypothetical protein	5949,72	12,54	0,00	1,00	0,22	0,00	0,997	9,98E-01	0,00	0,22	0,59	0,35	1,00	1,16	1,51	1,27	9,98E-01	1,92E-01	5,78E-03	7,05E-02
SACOL2314	yocS	yocS	-	sodium/bile acid symporter family protein	70,88	6,15	0,00	1,00	0,32	0,00	0,997073	9,98E-01	0,00	0,36	-0,56	-0,76	1,00	1,28	0,68	0,59	9,98E-01	3,45E-01	7,19E-02	4,67E-02
SACOL0702	SACOL0702	SACOL0702	-	hypothetical protein	17,50	4,13	0,00	1,00	0,66	0,00	0,998866	9,99E-01	0,00	0,18	0,82	0,45	1,00	1,13	1,77	1,37	9,99E-01	6,91E-01	1,14E-01	3,75E-01
SACOL0246	lytR	lytS lytR	MgrA	response regulator LytR	125.94	6.98	0.00	1.00	0.38	0.00	0.999706	1.00E+00	0.00	0.11	0	-0.1	1.00	1.08	1	0.93	1.00E+00	7.31E-01	9.99E-01	8.11E-01

Table S1: DESeq2 differential gene expression analysis of *S. aureus* COL wild type and the *mhqR* mutant before and after MHQ stress. *S. aureus* wild type and *mhqR* mutant cells were grown in 3 biological replicates and harvested before and after exposure to 45 µM MHQ stress. The RNA-isolation, library preparation, sequencing and mapping was performed as described in the methods section. Transcripts were analyzed for differential gene expression using the software DEseq2 included in the ReadXplorer v2.2 software. The signal intensity value (A-value) was calculated by log2 base mean of normalized read counts and the signal intensity ratio (M-value) by log2 fold change. The evaluation of the differential RNAseq data was performed using an adjusted p-value cut-off of P ≤ 0.05 and a signal intensity ratio (M-value) cut-off of ≥ 0.6 or ≤ - 0.6 (printed in bold). Genes were classified into regulons according to the RegPrecise database and Aureowiki database.
