# Supplementary material for: The MarR-Type Repressor MhqR Confers Quinone and Antimicrobial Resistance in Staphylococcus aureus
Source: Antioxid Redox Signal. 2019 Oct 17;31(16):1235–52. doi: 10.1089/ars.2019.7750 (PMC6798810; doi:10.1089/ars.2019.7750)
Supplement: Supplemental data [file Supp_Table2.pdf]

Table S2: RNA-Seq transcriptome analysis of gene expression changes in *S. aureus* COL wild type and the *mhqR* deletion mutant before and after MHQ stress.

FunCat	Regulon	Locus	Operon	Gene symbol	log2-fold change (M-value)				Fold changes				Function
					WT MHQ/ WT co	Δ*mhqR* MHQ/ Δ*mhqR* co	Δ*mhqR* MHQ/ WT MHQ	Δ*mhqR* co/ WT co	WT MHQ/ WT co	Δ*mhqR* MHQ/ Δ*mhqR* co	Δ*mhqR* MHQ/ WT MHQ	Δ*mhqR* co/ WT co	
Amino acid biosynthesis	CodY	SACOL0302	brnQ2	brnQ2	1,31	1,02	-0,94	-0,65	2,48	2,03	0,52	0,64	branched-chain amino acid transport system II carrier protein
Amino acid biosynthesis	CodY	SACOL2574	ddh ywfG	ddh	-1,70	-0,45	0,88	-0,37	0,31	0,73	1,84	0,77	D-isomer specific 2-hydroxyacid dehydrogenase family protein
Amino acid biosynthesis	CodY	SACOL2575	ddh ywfG	ywfG	-0,96	-0,11	0,62	-0,22	0,52	0,93	1,54	0,86	N-succinyldiaminopimelate aminotransferase
Amino acid biosynthesis	CodY	SACOL2511	fnbA	fnbA	2,76	1,40	-1,57	-0,20	6,78	2,64	0,34	0,87	fibronectin-binding protein A
Amino acid biosynthesis	CodY	SACOL1362	hom thrC thrB	hom	-1,17	-0,58	0,63	0,03	0,44	0,67	1,55	1,02	homoserine dehydrogenase
Amino acid biosynthesis	CodY	SACOL1363	hom thrC thrB	thrC	-0,03	-0,26	0,19	0,39	0,98	0,84	1,14	1,31	threonine synthase
Amino acid biosynthesis	CodY	SACOL1364	hom thrC thrB	thrB	0,19	-0,01	0,23	0,40	1,14	0,99	1,17	1,32	homoserine kinase
Amino acid biosynthesis	CodY	SACOL2584	isaA	isaA	-1,32	-0,43	0,77	-0,12	0,40	0,74	1,71	0,92	immunodominant antigen A
Amino acid biosynthesis	CodY	SACOL1428	lysC asd dapA dapB dapD	lysC	-2,49	-0,47	0,65	-1,22	0,18	0,72	1,57	0,43	aspartate kinase
Amino acid biosynthesis	CodY	SACOL1429	lysC asd dapA dapB dapD	asd	-1,59	-0,33	0,18	-0,99	0,33	0,80	1,13	0,50	aspartate-semialdehyde dehydrogenase
Amino acid biosynthesis	CodY	SACOL1430	lysC asd dapA dapB dapD	dapA	-1,31	-0,08	0,12	-1,03	0,40	0,95	1,09	0,49	dihydrodipicolinate synthase
Amino acid biosynthesis	CodY	SACOL1431	lysC asd dapA dapB dapD	dapB	-0,71	0,05	0,02	-0,72	0,61	1,04	1,01	0,61	dihydrodipicolinate reductase
Amino acid biosynthesis	CodY	SACOL1432	lysC asd dapA dapB dapD	dapD	-0,40	0,13	-0,11	-0,61	0,76	1,09	0,93	0,66	2,3,4,5-tetrahydropyridine-2,6-dicarboxylate N-succinyltransferase
Amino acid biosynthesis	CodY	SACOL1963	putP	putP	-1,26	-0,39	0,42	-0,44	0,42	0,76	1,34	0,74	high affinity proline permease
Amino acid biosynthesis	CodY	SACOL1817	ribHBAED	ribH	2,54	1,24	-1,40	-0,11	5,81	2,36	0,38	0,93	riboflavin synthase beta subunit
Amino acid biosynthesis	CodY	SACOL1818	ribHBAED	ribA	2,42	1,39	-1,31	-0,28	5,33	2,62	0,40	0,82	bifunctional 3,4-dihydroxy-2-butanone-4-phosphate synthase/GTP cyclohydrolase II
Amino acid biosynthesis	CodY	SACOL1819	ribHBAED	ribB	2,59	1,37	-1,32	-0,11	6,01	2,58	0,40	0,93	riboflavin synthase alpha subunit
Amino acid biosynthesis	CodY	SACOL1820	ribHBAED	ribD	2,58	1,28	-1,46	-0,18	5,96	2,43	0,36	0,88	diaminohydroxyphosphoribosylaminopyrimidine deaminase
Amino acid biosynthesis	CodY	SACOL0924	SACOL0924 SACOL0925 SACOL0926	SACOL0924	-0,05	-0,04	-0,32	-0,31	0,97	0,97	0,80	0,81	hypothetical protein
Amino acid biosynthesis	CodY	SACOL0925	SACOL0924 SACOL0925 SACOL0926	SACOL0925	0,49	0,27	-0,38	-0,16	1,41	1,21	0,77	0,90	hypothetical protein
Amino acid biosynthesis	CodY	SACOL0926	SACOL0924 SACOL0925 SACOL0926	SACOL0926	0,81	0,36	-0,52	-0,08	1,75	1,28	0,70	0,95	5'-nucleotidase
Amino acid biosynthesis	CodY	SACOL1365	SACOL1365	SACOL1365	0,71	0,55	-0,02	0,13	1,64	1,46	0,99	1,09	HAD superfamily hydrolase
Amino acid biosynthesis	CodY	SACOL1774	SACOL1774 ptaA	SACOL1774	-0,06	-0,23	-0,35	-0,18	0,96	0,85	0,78	0,88	HAD superfamily hydrolase
Amino acid biosynthesis	CodY	SACOL1775	SACOL1774 ptaA	ptaA	-1,44	-0,64	0,81	0,01	0,37	0,64	1,75	1,01	PTS system, IIBC components
Amino acid biosynthesis	CodY	SACOL2318	SACOL2318	SACOL2318	1,17	-0,14	-1,29	0,00	2,25	0,91	0,41	1,00	hypothetical protein
Amino acid biosynthesis	CodY	SACOL2619	SACOL2619	SACOL2619	-1,82	-0,45	0,75	-0,58	0,28	0,73	1,68	0,67	amino acid permease
Amino acid biosynthesis	CodY	SACOL2295	ssaA	ssaA	1,19	1,55	0,55	0,22	2,27	2,93	1,46	1,16	secretory antigen SsaA
Amino acid biosynthesis	CodY	SACOL1477	steT ilvA1 ald	ilvA1	-0,36	0,48	0,62	-0,20	0,78	1,39	1,54	0,87	threonine ammonia-lyase
Amino acid biosynthesis	CodY	SACOL1478	steT ilvA1 ald	ald	-1,47	-0,10	0,72	-0,54	0,36	0,93	1,65	0,69	alanine dehydrogenase
Amino acid biosynthesis	CodY	SACOL1403	trpE trpG trpD trpC trpF trpB trpA	trpE	1,62	1,09	-0,02	0,38	3,08	2,13	0,99	1,30	anthranilate synthase component I
Amino acid biosynthesis	CodY	SACOL1404	trpE trpG trpD trpC trpF trpB trpA	trpG	0,91	1,15	0,22	-0,05	1,88	2,22	1,16	0,97	anthranilate synthase component II
Amino acid biosynthesis	CodY	SACOL1405	trpE trpG trpD trpC trpF trpB trpA	trpD	1,13	0,66	-0,17	0,24	2,18	1,58	0,89	1,18	anthranilate phosphoribosyltransferase
Amino acid biosynthesis	CodY	SACOL1406	trpE trpG trpD trpC trpF trpB trpA	trpC	1,03	0,76	-0,11	0,12	2,04	1,69	0,93	1,09	indole-3-glycerol-phosphate synthase
Amino acid biosynthesis	CodY	SACOL1407	trpE trpG trpD trpC trpF trpB trpA	trpF	1,89	0,69	-0,41	0,57	3,71	1,61	0,75	1,48	N-(5'-phosphoribosyl)anthranilate isomerase
Amino acid biosynthesis	CodY	SACOL1408	trpE trpG trpD trpC trpF trpB trpA	trpB	1,50	1,19	-0,31	-0,02	2,83	2,28	0,81	0,99	tryptophan synthase subunit beta
Amino acid biosynthesis	CodY	SACOL1409	trpE trpG trpD trpC trpF trpB trpA	trpA	1,29	0,49	-0,54	0,22	2,45	1,40	0,69	1,16	tryptophan synthase subunit alpha
Amino acid biosynthesis	CodY	SACOL1401	tyrA	tyrA	1,26	1,19	0,13	0,17	2,40	2,28	1,09	1,13	prephenate dehydrogenase
Amino acid biosynthesis	CodY	SACOL0023	yycJ	yycJ	0,68	0,31	-0,14	0,23	1,60	1,24	0,91	1,17	metallo-beta-lactamase YycJ
Amino acid utilization	HisR	SACOL0784	hisC	hisC	2,37	1,93	-0,84	-0,40	5,19	3,81	0,56	0,76	histidinol-phosphate aminotransferase
Ammonium biosynthesis	ArcR	SACOL2654	arcCDBA	arcC	-1,64	-0,87	0,90	0,13	0,32	0,55	1,87	1,09	carbamate kinase
Ammonium biosynthesis	ArcR	SACOL2655	arcCDBA	arcD	-0,94	-0,43	0,36	-0,12	0,52	0,74	1,28	0,92	APC family amino acid-polyamine-organocation transporter
Ammonium biosynthesis	ArcR	SACOL2656	arcCDBA	arcB	-2,29	-0,85	0,75	-0,60	0,20	0,55	1,68	0,66	ornithine carbamoyltransferase
Ammonium biosynthesis	ArcR	SACOL2657	arcCDBA	arcA	-3,38	-0,94	1,30	-0,95	0,10	0,52	2,46	0,52	arginine deiminase
Ammonium biosynthesis	ArgR	SACOL2653	arcR	arcR	-1,72	-0,81	0,96	0,05	0,30	0,57	1,95	1,04	Crp/FNR family transcriptional regulator
Ammonium biosynthesis	ArgR	SACOL0167	argBJCD	argB	-0,58	0,07	0,47	-0,16	0,67	1,05	1,39	0,90	acetylglutamate kinase
Ammonium biosynthesis	ArgR	SACOL0168	argBJCD	argJ	-0,88	-0,04	0,36	-0,45	0,54	0,97	1,28	0,73	glutamate N-acetyltransferase
Ammonium biosynthesis	ArgR	SACOL0169	argBJCD	argC	-1,48	-0,10	0,79	-0,48	0,36	0,93	1,73	0,72	N-acetyl-gamma-glutamyl-phosphate reductase
Ammonium biosynthesis	ArgR	SACOL0170	argBJCD	argD	-1,51	-0,35	0,52	-0,52	0,35	0,78	1,43	0,70	ornithine--oxo-acid transaminase
Ammonium biosynthesis	ArgR	SACOL0963	argH argG	argH	-3,84	-2,24	0,04	-0,54	0,07	0,21	1,03	0,69	argininosuccinate lyase
Ammonium biosynthesis	ArgR	SACOL0964	argH argG	argG	-4,95	-2,68	-0,19	-1,08	0,03	0,16	0,88	0,47	argininosuccinate synthase
Ammonium biosynthesis	ArgR	SACOL1915	artQM	artQ	-2,53	-0,88	0,97	-0,26	0,17	0,54	1,96	0,84	glutamate ABC transporter, ATP-binding protein
Ammonium biosynthesis	ArgR	SACOL1916	artQM	artM	-2,93	-0,91	0,97	-0,43	0,13	0,53	1,96	0,74	amino acid ABC transporter, membrane protein
Ammonium biosynthesis	ArgR	SACOL2632	cudT	cudT	-3,52	-1,84	1,58	-0,08	0,09	0,28	2,99	0,95	BCCT family betaine/carnitine/choline transporter
Ammonium metabolism	FruR	SACOL0757	deoR	deoR	0,22	0,07	0,24	0,23	1,16	1,05	1,18	1,17	DeoR family transcriptional regulator
Ammonium metabolism	FruR	SACOL0758	fruAB	fruB	1,05	0,43	0,04	0,57	2,08	1,35	1,03	1,48	1-phosphofructokinase
Ammonium metabolism	GlnR	SACOL1328	glnRA	glnR	-0,77	-0,61	-0,21	-0,37	0,59	0,66	0,86	0,77	glutamate--ammonia ligase repressor
Ammonium metabolism	GlnR	SACOL1329	glnRA	glnA	-0,66	-0,35	0,31	0,00	0,63	0,78	1,24	1,00	glutamate--ammonia ligase
Antibiotics resistance	VraSR	SACOL2435	glxK SACOL2436	glxK	-1,15	-0,51	0,54	-0,11	0,45	0,70	1,45	0,93	glycerate kinase
Antibiotics resistance	VraSR	SACOL2436	glxK SACOL2436	SACOL2436	-1,05	-0,54	0,47	-0,04	0,48	0,69	1,39	0,97	hypothetical protein
Antibiotics resistance	VraSR	SACOL1897	prsA	prsA	0,70	0,23	-0,93	-0,46	1,62	1,17	0,52	0,73	peptidylprolyl isomerase
Antibiotics resistance	VraSR	SACOL1932	sgtB	sgtB	-1,36	-0,59	0,70	-0,06	0,39	0,66	1,62	0,96	monofunctional glycosyltransferase
Antibiotics resistance	VraSR	SACOL1942	vraRSTU	vraR	1,46	0,73	-0,64	0,07	2,75	1,66	0,64	1,05	response regulator VraR
Antibiotics resistance	VraSR	SACOL1943	vraRSTU	vraS	1,33	0,65	-0,60	0,07	2,51	1,57	0,66	1,05	sensor histidine kinase VraS
Antibiotics resistance	VraSR	SACOL1944	vraRSTU	vraT	1,05	0,62	-0,39	0,03	2,08	1,54	0,76	1,02	hypothetical membrane protein
Antibiotics resistance	VraSR	SACOL1945	vraRSTU	vraU	0,93	0,49	-0,37	0,06	1,91	1,40	0,77	1,04	hypothetical protein
Cell wall biosynthesis	CidR	SACOL2198	budA1 alsS	budA1	2,77	1,21	-1,12	0,68	6,83	2,31	0,46	1,60	acetolactate decarboxylase
Cell wall biosynthesis	CidR	SACOL2199	budA1 alsS	alsS	2,25	1,35	-0,72	0,10	4,77	2,55	0,61	1,07	acetolactate synthase
Cell wall biosynthesis	CidR	SACOL2553	cidCBA	cidC	2,20	0,61	-1,06	0,55	4,58	1,53	0,48	1,46	pyruvate oxidase
Cell wall biosynthesis	CidR	SACOL2554	cidCBA	cidB	0,77	-0,28	-0,50	0,54	1,71	0,82	0,71	1,45	hypothetical membrane protein
**Cell wall biosynthesis**	**GraRS**	**SACOL0031**	**-**	**ugpQ**	**1,47**	**0,77**	**-0,73**	**-0,03**	**2,78**	**1,71**	**0,60**	**0,98**	**glycerophosphodiester phosphodiesterase**
**Cell wall biosynthesis**	**GraRS**	**SACOL2129**	**-**	**deoC2**	**1,40**	**2,32**	**1,19**	**0,26**	**2,65**	**4,99**	**2,28**	**1,20**	**deoxyribose-phosphate aldolase**
Cell wall biosynthesis	GraRS	SACOL1743	aapA	aapA	-0,84	-0,05	0,55	-0,24	0,56	0,97	1,46	0,85	APC family amino acid-polyamine-organocation transporter
**Cell wall biosynthesis**	**GraRS**	**SACOL1760**	**ackA SACOL1761**	**ackA**	**1,24**	**0,80**	**-0,58**	**-0,15**	**2,35**	**1,74**	**0,67**	**0,90**	**acetate kinase**
**Cell wall biosynthesis**	**GraRS**	**SACOL1761**	**ackA SACOL1761**	**SACOL1761**	**2,28**	**1,75**	**-1,14**	**-0,58**	**4,85**	**3,36**	**0,45**	**0,67**	**hypothetical protein**
**Cell wall biosynthesis**	**GraRS**	**SACOL0190**	**acpD**	**acpD**	**7,79**	**5,38**	**-1,31**	**1,05**	**221,15**	**41,64**	**0,40**	**2,07**	**azoreductase**
Cell wall biosynthesis	GraRS	SACOL0135	adhE	adhE	-0,95	-0,21	0,37	-0,35	0,52	0,86	1,29	0,78	alcohol dehydrogenase
**Cell wall biosynthesis**	**GraRS**	**SACOL2192**	**akr5F**	**akr5F**	**1,78**	**0,66**	**-1,11**	**-0,01**	**3,43**	**1,58**	**0,46**	**0,99**	**oxidoreductase, aldo/keto reductase**
Cell wall biosynthesis	GraRS	SACOL2094	atpCDGAHFEBI mnaA upp glyA ywlG	atpC	-0,86	-0,62	0,25	0,01	0,55	0,65	1,19	1,01	proton- translocating F-type ATPase epsilon subunit
Cell wall biosynthesis	GraRS	SACOL2095	atpCDGAHFEBI mnaA upp glyA ywlG	atpD	-0,94	-0,70	0,35	0,10	0,52	0,62	1,27	1,07	proton-translocating F-type ATPase delta subunit
Cell wall biosynthesis	GraRS	SACOL2096	atpCDGAHFEBI mnaA upp glyA ywlG	atpG	-1,14	-0,80	0,54	0,19	0,45	0,57	1,45	1,14	proton-translocating F-type ATPase gamma subunit
Cell wall biosynthesis	GraRS	SACOL2097	atpCDGAHFEBI mnaA upp glyA ywlG	atpA	-1,13	-0,59	0,61	0,06	0,46	0,66	1,53	1,04	proton-translocating F-type ATPase alpha subunit
Cell wall biosynthesis	GraRS	SACOL2098	atpCDGAHFEBI mnaA upp glyA ywlG	atpH	-1,09	-0,70	0,50	0,10	0,47	0,62	1,41	1,07	proton-translocating F-type ATPase beta subunit
Cell wall biosynthesis	GraRS	SACOL2099	atpCDGAHFEBI mnaA upp glyA ywlG	atpF	-1,03	-0,72	0,47	0,16	0,49	0,61	1,39	1,12	proton-translocating F-type ATPase subunit B
Cell wall biosynthesis	GraRS	SACOL2100	atpCDGAHFEBI mnaA upp glyA ywlG	atpE	-0,99	-0,86	0,40	0,26	0,50	0,55	1,32	1,20	proton-translocating F-type ATPase epsilon subunit C
Cell wall biosynthesis	GraRS	SACOL2101	atpCDGAHFEBI mnaA upp glyA ywlG	atpB	-0,92	-0,81	0,33	0,21	0,53	0,57	1,26	1,16	proton-translocating F-type ATPase beta subunit A
Cell wall biosynthesis	GraRS	SACOL2102	atpCDGAHFEBI mnaA upp glyA ywlG	atpI	-0,88	-0,81	0,29	0,22	0,55	0,57	1,22	1,16	hypothetical protein
Cell wall biosynthesis	GraRS	SACOL2103	atpCDGAHFEBI mnaA upp glyA ywlG	mnaA	-0,57	-0,36	0,34	0,13	0,68	0,78	1,27	1,09	UDP-N-acetylglucosamine 2-epimerase
Cell wall biosynthesis	GraRS	SACOL2104	atpCDGAHFEBI mnaA upp glyA ywlG	upp	-0,54	-0,34	0,29	0,09	0,69	0,79	1,22	1,06	uracil phosphoribosyltransferase
Cell wall biosynthesis	GraRS	SACOL2105	atpCDGAHFEBI mnaA upp glyA ywlG	glyA	-0,48	-0,33	0,26	0,09	0,71	0,80	1,20	1,06	glycine hydroxymethyltransferase
Cell wall biosynthesis	GraRS	SACOL2106	atpCDGAHFEBI mnaA upp glyA ywlG	ywlG	-0,48	-0,37	0,22	0,11	0,72	0,77	1,16	1,08	hypothetical protein
Cell wall biosynthesis	GraRS	SACOL0171	brnQ1	brnQ1	-1,66	-0,71	0,65	-0,28	0,32	0,61	1,57	0,82	possible LIVCS family branched chain amino acid:cation symporter
**Cell wall biosynthesis**	**GraRS**	**SACOL0975**	**cdr yitU**	**cdr**	**1,11**	**0,14**	**-1,11**	**-0,15**	**2,16**	**1,10**	**0,46**	**0,90**	**coenzyme A disulfide reductase**
**Cell wall biosynthesis**	**GraRS**	**SACOL0976**	**cdr yitU**	**yitU**	**1,20**	**0,02**	**-1,39**	**-0,23**	**2,29**	**1,01**	**0,38**	**0,85**	**haloacid dehalogenase-like hydrolase**
**Cell wall biosynthesis**	**GraRS**	**SACOL1303**	**cinA**	**cinA**	**1,32**	**0,59**	**-0,66**	**0,05**	**2,49**	**1,51**	**0,63**	**1,04**	**competence-damage inducible protein CinA**
**Cell wall biosynthesis**	**GraRS**	**SACOL1741**	**citCZ**	**citC**	**1,85**	**-0,79**	**-2,08**	**0,49**	**3,62**	**0,58**	**0,24**	**1,40**	**isocitrate dehydrogenase (NADP(+))**
**Cell wall biosynthesis**	**GraRS**	**SACOL1742**	**citCZ**	**citZ**	**1,42**	**-0,97**	**-2,17**	**0,17**	**2,68**	**0,51**	**0,22**	**1,13**	**citrate (Si)-synthase**
**Cell wall biosynthesis**	**GraRS**	**SACOL1735**	**coaE fpg polA**	**coaE**	**2,02**	**0,41**	**-1,38**	**0,20**	**4,07**	**1,33**	**0,38**	**1,15**	**dephospho-CoA kinase**
**Cell wall biosynthesis**	**GraRS**	**SACOL1736**	**coaE fpg polA**	**fpg**	**1,95**	**0,11**	**-1,42**	**0,40**	**3,86**	**1,08**	**0,37**	**1,32**	**DNA-formamidopyrimidine glycosylase**
**Cell wall biosynthesis**	**GraRS**	**SACOL1737**	**coaE fpg polA**	**polA**	**1,43**	**-0,10**	**-1,21**	**0,29**	**2,69**	**0,93**	**0,43**	**1,22**	**DNA-directed DNA polymerase I**
**Cell wall biosynthesis**	**GraRS**	**SACOL1094**	**cydA cydB**	**cydA**	**1,68**	**0,81**	**-0,87**	**-0,01**	**3,21**	**1,75**	**0,55**	**0,99**	**cytochrome d ubiquinol oxidase subunit I**
**Cell wall biosynthesis**	**GraRS**	**SACOL1095**	**cydA cydB**	**cydB**	**1,24**	**0,65**	**-0,55**	**0,03**	**2,37**	**1,57**	**0,68**	**1,02**	**cytochrome d ubiquinol oxidase subunit II**
Cell wall biosynthesis	GraRS	SACOL0603	dck dgk	dck	-0,94	-0,05	1,15	0,24	0,52	0,97	2,22	1,18	deoxynucleoside kinase
Cell wall biosynthesis	GraRS	SACOL0604	dck dgk	dgk	-0,40	0,56	0,94	-0,02	0,76	1,47	1,92	0,99	deoxynucleoside kinase
**Cell wall biosynthesis**	**GraRS**	**SACOL1522**	**ebpS**	**ebpS**	**1,97**	**-0,03**	**-2,05**	**-0,07**	**3,93**	**0,98**	**0,24**	**0,95**	**elastin-binding protein**
Cell wall biosynthesis	GraRS	SACOL0275	esaA essA esaB essB yukA esxC esxB esaE esxD SACOL0281	essB	-2,57	-0,70	-0,74	-2,34	0,17	0,62	0,60	0,20	virulence protein EssB
Cell wall biosynthesis	GraRS	SACOL0281	esaA essA esaB essB yukA esxC esxB esaE esxD SACOL0281	SACOL0281	-1,18	-0,66	-0,33	-0,82	0,44	0,63	0,80	0,57	hypothetical protein
**Cell wall biosynthesis**	**GraRS**	**SACOL2527**	**fbp**	**fbp**	**2,77**	**-0,01**	**-3,54**	**-0,71**	**6,84**	**0,99**	**0,09**	**0,61**	**fructose-bisphosphatase**
**Cell wall biosynthesis**	**GraRS**	**SACOL1410**	**femAB**	**femA**	**1,62**	**0,39**	**-1,29**	**-0,08**	**3,07**	**1,31**	**0,41**	**0,95**	**methicillin resistance factor FemA**
**Cell wall biosynthesis**	**GraRS**	**SACOL1411**	**femAB**	**femB**	**1,70**	**0,41**	**-1,18**	**0,09**	**3,25**	**1,33**	**0,44**	**1,06**	**methicillin resistance factor FemB**
**Cell wall biosynthesis**	**GraRS**	**SACOL1782**	**fhs**	**fhs**	**0,75**	**-0,57**	**-1,70**	**-0,40**	**1,68**	**0,67**	**0,31**	**0,76**	**formate--tetrahydrofolate ligase**
**Cell wall biosynthesis**	**GraRS**	**SACOL0555**	**ftsH**	**ftsH**	**1,46**	**0,67**	**-1,01**	**-0,22**	**2,76**	**1,59**	**0,50**	**0,86**	**cell division protein FtsH**
**Cell wall biosynthesis**	**GraRS**	**SACOL1593**	**gcvPB gcvPA gcvT**	**gcvPB**	**1,03**	**-0,20**	**-1,39**	**-0,19**	**2,04**	**0,87**	**0,38**	**0,88**	**glycine dehydrogenase (decarboxylating) subunit 2**
**Cell wall biosynthesis**	**GraRS**	**SACOL1594**	**gcvPB gcvPA gcvT**	**gcvPA**	**0,87**	**-0,24**	**-1,25**	**-0,16**	**1,83**	**0,85**	**0,42**	**0,90**	**glycine dehydrogenase (decarboxylating) subunit 1**
**Cell wall biosynthesis**	**GraRS**	**SACOL1595**	**gcvPB gcvPA gcvT**	**gcvT**	**0,93**	**-0,18**	**-1,29**	**-0,20**	**1,91**	**0,88**	**0,41**	**0,87**	**aminomethyltransferase**
Cell wall biosynthesis	GraRS	SACOL1319	glpF	glpF	-2,13	-0,98	1,40	0,37	0,23	0,51	2,64	1,29	MIP family major intrinsic protein channel protein
**Cell wall biosynthesis**	**GraRS**	**SACOL0207**	**glpQ1**	**glpQ1**	**1,22**	**1,52**	**0,26**	**-0,05**	**2,33**	**2,87**	**1,20**	**0,97**	**possible glycerophosphodiester phosphodiesterase**
Cell wall biosynthesis	GraRS	SACOL2514	gntPKR SACOL2517	gntP	-1,03	-1,16	0,57	0,52	0,49	0,45	1,48	1,43	GntP family gluconate:proton (H+) symporter
Cell wall biosynthesis	GraRS	SACOL2515	gntPKR SACOL2517	gntK	-0,56	-0,54	-0,18	-0,16	0,68	0,69	0,88	0,90	gluconokinase
Cell wall biosynthesis	GraRS	SACOL2516	gntPKR SACOL2517	gntR	-0,92	-0,38	-0,17	-0,68	0,53	0,77	0,89	0,62	gluconate operon transcriptional repressor
Cell wall biosynthesis	GraRS	SACOL2517	gntPKR SACOL2517	SACOL2517	-0,28	0,22	-0,12	-0,61	0,82	1,16	0,92	0,66	MerR family transcriptional regulator
Cell wall biosynthesis	GraRS	SACOL0715	graXRS	graX	0,42	0,10	-0,53	-0,20	1,34	1,07	0,69	0,87	hypothetical protein
Cell wall biosynthesis	GraRS	SACOL0716	graXRS	graR	0,53	0,12	-0,46	-0,05	1,45	1,09	0,73	0,97	response regulator
**Cell wall biosynthesis**	**GraRS**	**SACOL0717**	**graXRS**	**graS**	**0,80**	**-0,04**	**-0,62**	**0,21**	**1,74**	**0,97**	**0,65**	**1,16**	**sensor histidine kinase**
Cell wall biosynthesis	GraRS	SACOL0825	hprK lgt maa SACOL0828	hprK	-0,16	-0,17	0,03	0,03	0,90	0,89	1,02	1,02	HPr kinase
Cell wall biosynthesis	GraRS	SACOL0826	hprK lgt maa SACOL0828	lgt	-0,13	-0,16	-0,04	-0,02	0,91	0,90	0,97	0,99	prolipoprotein diacylglyceryl transferase
Cell wall biosynthesis	GraRS	SACOL0827	hprK lgt maa SACOL0828	maa	0,01	-0,18	-0,11	0,07	1,01	0,88	0,93	1,05	putative acetyltransferase
Cell wall biosynthesis	GraRS	SACOL0828	hprK lgt maa SACOL0828	SACOL0828	0,34	0,01	-0,18	0,13	1,26	1,01	0,88	1,09	hypothetical TPR domain protein
**Cell wall biosynthesis**	**GraRS**	**SACOL0556**	**hslO**	**hslO**	**2,21**	**1,41**	**-0,67**	**0,12**	**4,64**	**2,66**	**0,63**	**1,09**	**heat shock protein Hsp33**
Cell wall biosynthesis	GraRS	SACOL2363	lctP 2	lctP 2	-1,19	-0,68	0,53	0,01	0,44	0,62	1,44	1,01	LctP family L-lactate permease
Cell wall biosynthesis	GraRS	SACOL0192	malKEFD	malK	-0,73	0,17	-0,79	-1,65	0,60	1,13	0,58	0,32	maltose ABC transporter ATP-binding protein
**Cell wall biosynthesis**	**GraRS**	**SACOL0193**	**malKEFD**	**malE**	**0,62**	**-0,14**	**-0,42**	**0,30**	**1,54**	**0,91**	**0,75**	**1,23**	**maltose ABC transporter, binding protein**
**Cell wall biosynthesis**	**GraRS**	**SACOL0194**	**malKEFD**	**malF**	**1,84**	**0,14**	**-0,94**	**0,65**	**3,57**	**1,10**	**0,52**	**1,57**	**maltose ABC transporter, permease**
**Cell wall biosynthesis**	**GraRS**	**SACOL0195**	**malKEFD**	**malD**	**2,72**	**0,54**	**-1,15**	**0,92**	**6,61**	**1,45**	**0,45**	**1,89**	**maltose ABC transporter, permease**
**Cell wall biosynthesis**	**GraRS**	**SACOL0973**	**mhpD**	**mhpD**	**1,38**	**0,44**	**-1,11**	**-0,19**	**2,59**	**1,36**	**0,46**	**0,88**	**2-keto-4-pentenoate hydratase**
**Cell wall biosynthesis**	**GraRS**	**SACOL2566**	**mmpL**	**mmpL**	**1,28**	**0,70**	**-0,41**	**0,14**	**2,43**	**1,62**	**0,75**	**1,10**	**MmpL efflux pump**
**Cell wall biosynthesis**	**GraRS**	**SACOL1633**	**mtaB rsmE prmA dnaJ dnaK grpE hrcA**	**mtaB**	**0,81**	**-0,12**	**-1,06**	**-0,16**	**1,75**	**0,92**	**0,48**	**0,90**	**possible 2-methylthioadenine synthase**
**Cell wall biosynthesis**	**GraRS**	**SACOL1634**	**mtaB rsmE prmA dnaJ dnaK grpE hrcA**	**rsmE**	**1,59**	**0,00**	**-1,72**	**-0,16**	**3,02**	**1,00**	**0,30**	**0,90**	**hypothetical protein**
**Cell wall biosynthesis**	**GraRS**	**SACOL1635**	**mtaB rsmE prmA dnaJ dnaK grpE hrcA**	**prmA**	**2,28**	**0,33**	**-2,24**	**-0,32**	**4,87**	**1,26**	**0,21**	**0,80**	**ribosomal protein L11 methyltransferase**
**Cell wall biosynthesis**	**GraRS**	**SACOL2146**	**mtlF mtlR mtlA mtlD**	**mtlF**	**1,28**	**-0,03**	**-0,85**	**0,41**	**2,43**	**0,98**	**0,55**	**1,33**	**PTS system, mannitol-specific IIBC components**
Cell wall biosynthesis	GraRS	SACOL2147	mtlF mtlR mtlA mtlD	mtlR	0,26	0,13	0,07	0,18	1,20	1,09	1,05	1,13	BglG family transcriptional antiterminator
Cell wall biosynthesis	GraRS	SACOL2148	mtlF mtlR mtlA mtlD	mtlA	-0,13	0,06	-0,22	-0,39	0,92	1,04	0,86	0,76	PTS system, mannitol-specific IIA component
Cell wall biosynthesis	GraRS	SACOL2149	mtlF mtlR mtlA mtlD	mtlD	-0,23	0,13	-0,03	-0,38	0,85	1,09	0,98	0,77	mannitol-1-phosphate 5-dehydrogenase
Cell wall biosynthesis	GraRS	SACOL2707	mtsA mtsB mtsC SACOL2710	mtsC	0,59	0,61	-0,50	-0,47	1,50	1,53	0,71	0,72	cobalt (Co2) ABC superfamily ATP binding cassette transporter, membrane prot.
**Cell wall biosynthesis**	**GraRS**	**SACOL2708**	**mtsA mtsB mtsC SACOL2710**	**mtsB**	**0,79**	**0,66**	**-0,50**	**-0,35**	**1,73**	**1,58**	**0,71**	**0,78**	**cobalt ABC transporter ATPase**
**Cell wall biosynthesis**	**GraRS**	**SACOL2709**	**mtsA mtsB mtsC SACOL2710**	**mtsA**	**0,68**	**0,68**	**-0,56**	**-0,42**	**1,60**	**1,60**	**0,68**	**0,75**	**hypothetical protein**
**Cell wall biosynthesis**	**GraRS**	**SACOL2710**	**mtsA mtsB mtsC SACOL2710**	**SACOL2710**	**0,72**	**0,40**	**-0,85**	**-0,42**	**1,64**	**1,32**	**0,55**	**0,75**	**hypothetical protein**
**Cell wall biosynthesis**	**GraRS**	**SACOL1163**	**murI rdgB SACOL1163**	**SACOL1163**	**2,55**	**0,91**	**-1,07**	**0,54**	**5,85**	**1,88**	**0,48**	**1,45**	**hypothetical protein**
**Cell wall biosynthesis**	**GraRS**	**SACOL1162**	**murI rdgB SACOL1164**	**rdgB**	**2,26**	**0,47**	**-1,05**	**0,71**	**4,79**	**1,39**	**0,48**	**1,64**	**deoxyribonucleotide triphosphate pyrophosphatase**
**Cell wall biosynthesis**	**GraRS**	**SACOL1161**	**murI rdgB SACOL1165**	**murI**	**1,71**	**0,07**	**-0,80**	**0,82**	**3,28**	**1,05**	**0,57**	**1,77**	**glutamate racemase**
Cell wall biosynthesis	GraRS	SACOL0946	nhaC SACOL0947	nhaC	-1,07	-0,24	0,88	0,05	0,48	0,85	1,84	1,04	NhaC family sodium:proton (Na :H) antiporter
Cell wall biosynthesis	GraRS	SACOL0947	nhaC SACOL0947	SACOL0947	-0,68	-0,48	0,33	0,13	0,62	0,72	1,26	1,09	hypothetical protein
Cell wall biosynthesis	GraRS	SACOL0088	nptA	nptA	-1,36	-0,21	0,67	-0,45	0,39	0,86	1,59	0,73	possible PnaS family phosphate:sodium (Na+) symporter
**Cell wall biosynthesis**	**GraRS**	**SACOL0781**	**opuBA opuBB**	**opuBA**	**2,26**	**1,05**	**-1,03**	**0,16**	**4,79**	**2,07**	**0,49**	**1,12**	**glycine/betaine/carnitine/choline ABC transporter, ATP-binding**
**Cell wall biosynthesis**	**GraRS**	**SACOL0783**	**opuBA opuBB**	**opuBB**	**2,60**	**1,23**	**-1,26**	**0,09**	**6,04**	**2,35**	**0,42**	**1,06**	**glycine betaine/carnitine/choline ABC transporter, membrane prot.**
**Cell wall biosynthesis**	**GraRS**	**SACOL2450**	**opuCD opuCC opuCB opuCA**	**opuCD**	**2,30**	**1,54**	**-1,40**	**-0,63**	**4,91**	**2,91**	**0,38**	**0,65**	**glycine betaine/L-proline ABC transporter, membrane protein**

FunCat	Regulon	Locus	Operon	Gene symbol	log2-fold change (M-value)				Fold changes				Function
					WT MHQ/ WT co	Δ*mhqR* MHQ/ Δ*mhqR* co	Δ*mhqR* MHQ/ WT MHQ	Δ*mhqR* co/ WT co	WT MHQ/ WT co	Δ*mhqR* MHQ/ Δ*mhqR* co	Δ*mhqR* MHQ/ WT MHQ	Δ*mhqR* co/ WT co	
Cell wall biosynthesis	GraRS	SACOL2451	opuCD opuCC opuCB opuCA	opuCC	2,16	1,59	-1,37	-0,77	4,46	3,01	0,39	0,59	glycine betaine/L-proline ABC transporter, binding protein
Cell wall biosynthesis	GraRS	SACOL2452	opuCD opuCC opuCB opuCA	opuCB	2,17	1,32	-1,45	-0,58	4,50	2,50	0,37	0,67	glycine betaine/choline ABC transporter, membrane protein
Cell wall biosynthesis	GraRS	SACOL2453	opuCD opuCC opuCB opuCA	opuCA	1,78	1,38	-1,14	-0,71	3,44	2,60	0,45	0,61	glycine betaine/choline ABC transporter, ATP-binding protein
Cell wall biosynthesis	GraRS	SACOL2242	pbuG	pbuG	-1,12	-0,53	0,35	-0,24	0,46	0,69	1,27	0,85	NCS2 family nucleobase:cation symporter-2
Cell wall biosynthesis	GraRS	SACOL2128	pdp	pdp	1,30	1,81	0,80	0,28	2,46	3,51	1,74	1,21	pyrimidine nucleoside phosphorylase
Cell wall biosynthesis	GraRS	SACOL0125	phnE1 phnE2 phnC phnD	phnE1	-0,42	-0,12	-0,25	-0,41	0,75	0,92	0,84	0,75	phosphonate ABC transporter, permease
Cell wall biosynthesis	GraRS	SACOL0126	phnE1 phnE2 phnC phnD	phnE2	-0,63	-0,06	-0,15	-0,54	0,65	0,96	0,90	0,69	phosphonate ABC transporter, permease
Cell wall biosynthesis	GraRS	SACOL0127	phnE1 phnE2 phnC phnD	phnC	-0,79	-0,16	0,55	-0,04	0,58	0,90	1,46	0,97	phosphonate ABC transporter, ATP-binding protein
Cell wall biosynthesis	GraRS	SACOL0128	phnE1 phnE2 phnC phnD	phnD	-2,00	-0,46	1,04	-0,37	0,25	0,73	2,06	0,77	phosphonate ABC transporter, phosphonate-binding protein
Cell wall biosynthesis	GraRS	SACOL0572	radA pilT	radA	1,72	0,12	-2,12	-0,55	3,30	1,09	0,23	0,68	DNA repair protein RadA
Cell wall biosynthesis	GraRS	SACOL0573	radA pilT	pilT	0,86	-0,17	-1,95	-0,93	1,82	0,89	0,26	0,52	Integral membrane protein (PIN domain superfamily)
Cell wall biosynthesis	GraRS	SACOL2518	relP	relP	-0,85	0,34	-0,17	-1,33	0,55	1,27	0,89	0,40	hypothetical protein
Cell wall biosynthesis	GraRS	SACOL0077	SACOL0077	SACOL0077	-0,72	-0,30	0,45	0,02	0,61	0,81	1,37	1,01	hypothetical membrane protein
Cell wall biosynthesis	GraRS	SACOL0259	SACOL0259	SACOL0259	-1,01	-1,27	-0,09	0,16	0,50	0,41	0,94	1,12	hypothetical protein
Cell wall biosynthesis	GraRS	SACOL0283	SACOL0283	SACOL0283	-2,06	-0,84	0,55	-0,63	0,24	0,56	1,46	0,65	hypothetical membrane protein
Cell wall biosynthesis	GraRS	SACOL0300	SACOL0300	SACOL0300	-1,77	-1,51	0,20	-0,06	0,29	0,35	1,15	0,96	hypothetical membrane protein
Cell wall biosynthesis	GraRS	SACOL0466	SACOL0466 SACOL0467	SACOL0466	3,52	0,91	-2,30	0,20	11,44	1,88	0,20	1,15	hypothetical membrane protein
Cell wall biosynthesis	GraRS	SACOL0467	SACOL0466 SACOL0467	SACOL0467	3,35	0,74	-2,40	0,12	10,22	1,67	0,19	1,09	hypothetical protein
Cell wall biosynthesis	GraRS	SACOL0508	SACOL0508	SACOL0508	1,01	0,34	-0,59	0,08	2,02	1,27	0,66	1,06	hypothetical protein
Cell wall biosynthesis	GraRS	SACOL0511	SACOL0511 SACOL0512	SACOL0511	0,53	0,82	0,47	0,18	1,44	1,77	1,39	1,13	hypothetical protein
Cell wall biosynthesis	GraRS	SACOL0512	SACOL0511 SACOL0512	SACOL0512	0,98	0,74	-0,47	-0,21	1,97	1,67	0,72	0,86	hypothetical membrane protein
Cell wall biosynthesis	GraRS	SACOL0606	SACOL0606 azo1	SACOL0606	-0,08	1,49	1,38	-0,15	0,95	2,81	2,60	0,90	hydrolase
Cell wall biosynthesis	GraRS	SACOL0607	SACOL0606 azo1	azo1	0,60	1,49	1,07	0,21	1,52	2,81	2,10	1,16	hypothetical protein
Cell wall biosynthesis	GraRS	SACOL0849	SACOL0849 SACOL0850	SACOL0849	-1,91	-0,53	-0,19	-1,49	0,27	0,69	0,88	0,36	hypothetical protein
Cell wall biosynthesis	GraRS	SACOL0850	SACOL0849 SACOL0850	SACOL0850	-1,90	-0,41	-0,32	-1,67	0,27	0,75	0,80	0,31	hypothetical protein
Cell wall biosynthesis	GraRS	SACOL0851	SACOL0851	SACOL0851	-2,51	-0,61	-0,56	-2,42	0,18	0,66	0,68	0,19	hypothetical lipoprotein
Cell wall biosynthesis	GraRS	SACOL1005	SACOL1004 pepF	pepF	1,37	1,76	-0,01	-0,37	2,58	3,39	0,99	0,77	M03 family oligopeptidase F
Cell wall biosynthesis	GraRS	SACOL1225	SACOL1225	SACOL1225	-1,06	0,50	0,19	-1,26	0,48	1,41	1,14	0,42	hypothetical lipoprotein
Cell wall biosynthesis	GraRS	SACOL1333	SACOL1333	SACOL1333	-1,39	-0,50	0,42	-0,37	0,38	0,71	1,34	0,77	hypothetical protein
Cell wall biosynthesis	GraRS	SACOL1456	SACOL1456 crr	SACOL1456	1,97	0,48	-1,03	0,44	3,92	1,39	0,49	1,36	hypothetical protein
Cell wall biosynthesis	GraRS	SACOL1457	SACOL1456 crr	crr	1,88	0,47	-0,98	0,42	3,69	1,39	0,51	1,34	PTS family glucose/glucoside (glc) porter component IIA
Cell wall biosynthesis	GraRS	SACOL1902	SACOL1902 SACOL1903	SACOL1902	1,13	0,29	-0,85	-0,02	2,20	1,22	0,55	0,99	hypothetical protein
Cell wall biosynthesis	GraRS	SACOL1903	SACOL1902 SACOL1903	SACOL1903	-0,63	-0,13	0,61	0,09	0,64	0,91	1,53	1,06	hypothetical protein
Cell wall biosynthesis	GraRS	SACOL2127	SACOL2127	SACOL2127	0,99	1,27	0,62	0,33	1,98	2,41	1,54	1,26	hypothetical membrane protein
Cell wall biosynthesis	GraRS	SACOL2196	SACOL2196	SACOL2196	1,26	-0,25	-1,31	0,16	2,40	0,84	0,40	1,12	possible HAD superfamily hydrolase
Cell wall biosynthesis	GraRS	SACOL2252	SACOL2252 femX	SACOL2252	0,81	-0,11	-0,78	0,12	1,75	0,93	0,58	1,09	RND resistance-nodulation-cell division acriflavin:proton antiporter
Cell wall biosynthesis	GraRS	SACOL2253	SACOL2252 femX	femX	0,26	-0,25	-0,53	-0,03	1,20	0,84	0,69	0,98	peptidoglycan pentaglycine interpeptide biosynthesis protein FmhB
Cell wall biosynthesis	GraRS	SACOL2343	SACOL2343 SACOL2344	SACOL2343	1,51	0,48	-1,23	-0,20	2,84	1,39	0,43	0,87	hypothetical protein
Cell wall biosynthesis	GraRS	SACOL2344	SACOL2343 SACOL2344	SACOL2344	1,32	0,33	-1,09	-0,10	2,49	1,26	0,47	0,93	hypothetical protein
Cell wall biosynthesis	GraRS	SACOL2430	SACOL2430 msbA3	SACOL2430	0,59	0,91	-0,10	-0,35	1,51	1,88	0,93	0,78	ABC transporter, ATP-binding protein
Cell wall biosynthesis	GraRS	SACOL2431	SACOL2430 msbA3	msbA3	-1,18	0,23	0,54	-0,68	0,44	1,17	1,45	0,62	ABC transporter, ATP-binding protein
Cell wall biosynthesis	GraRS	SACOL2477	SACOL2477 fmrO dapF	SACOL2477	3,62	2,11	-1,11	0,37	12,33	4,32	0,46	1,29	hypothetical protein
Cell wall biosynthesis	GraRS	SACOL2478	SACOL2477 fmrO dapF	fmrO	4,64	1,88	-1,88	0,68	24,97	3,68	0,27	1,60	rRNA methyltransferase FmrO
Cell wall biosynthesis	GraRS	SACOL2479	SACOL2477 fmrO dapF	dapF	4,08	2,16	-1,68	0,05	16,90	4,47	0,31	1,04	hypothetical protein
Cell wall biosynthesis	GraRS	SACOL2522	SACOL2522	SACOL2522	1,71	0,54	-2,80	-1,46	3,27	1,45	0,14	0,36	alkaline phosphatase
Cell wall biosynthesis	GraRS	SACOL2582	SACOL2582	SACOL2582	-1,23	0,12	1,62	0,28	0,43	1,09	3,07	1,21	possible acetyltransferase
Cell wall biosynthesis	GraRS	SACOL2384	sarZ	sarZ	-0,75	-0,91	0,17	0,31	0,59	0,53	1,13	1,24	MarR/SarA family transcriptional regulator
Cell wall biosynthesis	GraRS	SACOL0208	SCIN_2	SCIN_2	-1,05	-1,04	-0,14	-0,09	0,48	0,49	0,91	0,94	hypothetical protein
Cell wall biosynthesis	GraRS	SACOL0816	secA	secA	0,98	0,37	-0,80	-0,19	1,98	1,29	0,57	0,88	Sec Type I general secretory pathway preprotein translocase SecA
Cell wall biosynthesis	GraRS	SACOL0270	ssaA	ssaA	-2,35	-0,65	-0,95	-2,32	0,20	0,64	0,52	0,20	secretory antigen precursor SsaA
Cell wall biosynthesis	GraRS	SACOL1891	traP	traP	2,85	1,63	-1,50	-0,31	7,19	3,10	0,35	0,81	RNAIII-activating protein TRAP
Cell wall biosynthesis	GraRS	SACOL2550	trxA_6 SACOL2551	trxA_6	3,74	0,48	-2,40	0,72	13,40	1,39	0,19	1,65	possible thioredoxin
Cell wall biosynthesis	GraRS	SACOL2551	trxA_6 SACOL2551	SACOL2551	4,42	-0,07	-2,83	1,32	21,47	0,95	0,14	2,50	possible thioesterase
Cell wall biosynthesis	GraRS	SACOL2280	ureABCEFGD	ureA	1,97	0,29	-2,16	-0,36	3,93	1,22	0,22	0,78	urease gamma subunit
Cell wall biosynthesis	GraRS	SACOL2281	ureABCEFGD	ureB	1,73	0,22	-2,12	-0,52	3,33	1,16	0,23	0,70	urease beta subunit
Cell wall biosynthesis	GraRS	SACOL2282	ureABCEFGD	ureC	1,85	0,48	-1,87	-0,49	3,59	1,39	0,27	0,71	urease alpha subunit
Cell wall biosynthesis	GraRS	SACOL2283	ureABCEFGD	ureE	1,70	0,36	-1,81	-0,44	3,25	1,28	0,29	0,74	urease accessory protein UreE
Cell wall biosynthesis	GraRS	SACOL2284	ureABCEFGD	ureF	1,72	0,39	-1,75	-0,42	3,30	1,31	0,30	0,75	urease accessory protein UreF
Cell wall biosynthesis	GraRS	SACOL2285	ureABCEFGD	ureG	1,81	0,38	-1,59	-0,16	3,52	1,30	0,33	0,90	urease accessory protein UreG
Cell wall biosynthesis	GraRS	SACOL2286	ureABCEFGD	ureD	1,63	-0,02	-1,84	-0,21	3,09	0,99	0,28	0,86	urease accessory protein UreD
Cell wall biosynthesis	GraRS	SACOL0019	walRKHI	walR	0,75	0,24	-0,69	-0,18	1,68	1,18	0,62	0,88	response regulator VicR
Cell wall biosynthesis	GraRS	SACOL0020	walRKHI	walK	0,84	0,20	-0,73	-0,10	1,79	1,15	0,60	0,93	sensor histidine kinase VicK
Cell wall biosynthesis	GraRS	SACOL0021	walRKHI	walH	1,01	0,33	-0,74	-0,07	2,01	1,26	0,60	0,95	hypothetical protein YycH
Cell wall biosynthesis	GraRS	SACOL0022	walRKHI	walI	0,99	0,34	-0,73	-0,09	1,98	1,27	0,60	0,94	hypothetical protein YycI
Cell wall biosynthesis	GraRS	SACOL1440	xpaC telA	xpaC	2,02	0,62	-1,59	-0,20	4,06	1,54	0,33	0,87	5-bromo-4-chloroindolyl phosphate hydrolase
Cell wall biosynthesis	GraRS	SACOL1441	xpaC telA	telA	1,94	0,58	-1,45	-0,11	3,83	1,49	0,37	0,93	toxic anion resistance protein TelA
Cell wall biosynthesis	GraRS	SACOL2367	yhfP	yhfP	1,74	1,04	-1,26	-0,57	3,34	2,06	0,42	0,67	zinc-binding alcohol dehydrogenase
Cell wall biosynthesis	GraRS	SACOL0977	yitW	yitW	2,31	1,35	-1,10	-0,15	4,95	2,55	0,47	0,90	hypothetical protein
Cell wall biosynthesis	GraRS	SACOL1006	yjbM hbo	yjbM	1,09	-0,16	-1,58	-0,33	2,13	0,90	0,33	0,80	hypothetical protein
Cell wall biosynthesis	GraRS	SACOL1007	yjbM hbo	hbo	0,76	-0,35	-1,65	-0,55	1,70	0,78	0,32	0,68	globin family protein
Cell wall biosynthesis	GraRS	SACOL0656	ywqN	ywqN	0,99	-0,20	-1,07	0,10	1,99	0,87	0,48	1,07	putative flavodoxin
Cell wall biosynthesis	GraRS	SACOL1549	zwf	zwf	1,54	-0,34	-1,80	0,06	2,90	0,79	0,29	1,04	glucose-6-phosphate 1-dehydrogenase
DNA damage	LexA	SACOL1400	dinB2	dinB2	2,50	1,51	-1,22	-0,19	5,64	2,85	0,43	0,88	ImpB/MucB/SamB family DNA-damage repair protein
DNA damage	LexA	SACOL0789	queF SACOL0790	queF	-1,18	-0,36	0,93	0,10	0,44	0,78	1,91	1,07	7-cyano-7-deazaguanine reductase
DNA damage	LexA	SACOL0790	queF SACOL0790	SACOL0790	-1,31	-0,46	1,02	0,17	0,40	0,73	2,03	1,13	integral membrane domain-containing protein
DNA damage	LexA	SACOL1304	recA	recA	1,40	0,91	-0,66	-0,16	2,65	1,88	0,63	0,90	recombinase A
DNA damage	LexA	SACOL0823	uvrB uvrA	uvrB	0,97	0,06	-0,94	-0,04	1,96	1,04	0,52	0,97	excinuclease ABC subunit B
DNA damage	LexA	SACOL0824	uvrB uvrA	uvrA	1,73	0,51	-1,06	0,15	3,32	1,42	0,48	1,11	excinuclease ABC subunit A
Energy metabolism	CggR	SACOL0837	gapR gap pgk tpiA pgm eno	gapR	0,74	0,10	0,47	1,11	1,67	1,07	1,39	2,16	DeoR family transcriptional regulator
Energy metabolism	CggR	SACOL0838	gapR gap pgk tpiA pgm eno	gap	1,10	-0,11	0,44	1,66	2,15	0,93	1,36	3,16	glyceraldehyde-3-phosphate dehydrogenase (phosphorylating)
Energy metabolism	CggR	SACOL0839	gapR gap pgk tpiA pgm eno	pgk	1,44	0,04	0,17	1,57	2,71	1,03	1,13	2,97	phosphoglycerate kinase
Energy metabolism	CggR	SACOL0840	gapR gap pgk tpiA pgm eno	tpiA	1,41	-0,03	0,06	1,44	2,65	0,98	1,04	2,71	triose-phosphate isomerase
Energy metabolism	CggR	SACOL0841	gapR gap pgk tpiA pgm eno	pgm	1,41	-0,04	0,05	1,47	2,66	0,97	1,04	2,77	bisphosphoglycerate mutase
Energy metabolism	CggR	SACOL0842	gapR gap pgk tpiA pgm eno	eno	1,24	0,05	-0,11	1,07	2,37	1,04	0,93	2,10	phosphopyruvate hydratase
Energy metabolism	PDH	SACOL1102	pdhA pdhB pdhC pdhD	pdhA	0,44	-0,08	-0,43	0,08	1,36	0,95	0,74	1,06	pyruvate dehydrogenase (acetyl-transferring) alpha subunit
Energy metabolism	PDH	SACOL1103	pdhA pdhB pdhC pdhD	pdhB	0,53	-0,04	-0,53	0,03	1,44	0,97	0,69	1,02	pyruvate dehydrogenase (acetyl-transferring) beta subunit
Energy metabolism	PDH	SACOL1104	pdhA pdhB pdhC pdhD	pdhC	0,60	0,00	-0,50	0,09	1,52	1,00	0,71	1,06	dihydrolipoyllysine-residue acetyltransferase
Energy metabolism	PDH	SACOL1105	pdhA pdhB pdhC pdhD	pdhD	0,74	0,19	-0,38	0,17	1,67	1,14	0,77	1,13	dihydrolipoyl dehydrogenase
Fatty acid biosynthesis	FapR	SACOL0987	fabHF	fabH	1,31	0,73	-1,33	-0,77	2,48	1,66	0,40	0,59	3-oxoacyl-[acyl-carrier-protein] synthase
Fatty acid biosynthesis	FapR	SACOL0988	fabHF	fab	1,40	0,65	-1,41	-0,67	2,65	1,57	0,38	0,63	3-oxoacyl-[acyl-carrier-protein] synthase
Fatty acid biosynthesis	FapR	SACOL1016	fabI	fabI	0,92	0,08	-0,80	0,04	1,90	1,06	0,57	1,03	enoyl-[acyl-carrier-protein] reductase (NADH)
Fatty acid biosynthesis	FapR	SACOL1242	fapR plsX fabDG	fapR	0,08	-0,25	-0,90	-0,56	1,06	0,84	0,54	0,68	hypothetical protein
Fatty acid biosynthesis	FapR	SACOL1243	fapR plsX fabDG	plsX	0,37	-0,06	-0,84	-0,41	1,30	0,96	0,56	0,75	fatty acid/phospholipid synthesis protein
Fatty acid biosynthesis	FapR	SACOL1244	fapR plsX fabDG	fabD	0,60	0,06	-0,86	-0,32	1,51	1,04	0,55	0,80	[acyl-carrier-protein] S-malonyltransferase
Fatty acid biosynthesis	FapR	SACOL1245	fapR plsX fabDG	fabG	0,72	0,20	-0,77	-0,25	1,65	1,15	0,59	0,84	3-oxoacyl-[acyl-carrier-protein] reductase
LMW thiols	BSH	SACOL1190	bshC	bshC	2,48	0,97	-1,53	-0,03	5,58	1,96	0,35	0,98	bacillithiol biosynthesis cysteine-adding enzyme BshC
LMW thiols	BSH	SACOL1495	dinG birA papS bshA	dinG	1,17	0,53	-0,77	-0,14	2,25	1,44	0,59	0,91	DNA-directed DNA polymerase III epsilon subunit
LMW thiols	BSH	SACOL1496	dinG birA papS bshA	birA	0,68	-0,10	-0,80	-0,03	1,61	0,93	0,57	0,98	biotin--[acetyl-CoA-carboxylase] ligase/biotin operon transcriptional regulator
LMW thiols	BSH	SACOL1497	dinG birA papS bshA	papS	0,76	-0,33	-0,81	0,27	1,70	0,80	0,57	1,21	polynucleotide adenylyltransferase
LMW thiols	BSH	SACOL1498	dinG birA papS bshA	bshA	0,69	-0,35	-0,82	0,20	1,61	0,78	0,57	1,15	glycosyltransferase
LMW thiols	BSH	SACOL0614	SACOL0613 bshB folE2	bshB	1,78	0,37	-1,27	0,13	3,42	1,29	0,41	1,09	bacillithiol biosynthesis deacetylase BshB2
LMW thiols	BSH	SACOL1557	SACOL1557 brxB	SACOL1557	1,96	0,73	-1,09	0,13	3,90	1,66	0,47	1,09	hypothetical protein
LMW thiols	BSH	SACOL1558	SACOL1557 brxB	brxB	1,93	0,63	-1,24	0,05	3,82	1,55	0,42	1,04	bacillredoxin BrxB
LMW thiols	BSH	SACOL1520	ypdA	ypdA	2,00	0,91	-1,08	0,00	4,00	1,88	0,47	1,00	putative bacillithiol system oxidoreductase, YpdA family
LMW thiols	CymR	SACOL2278	caiA	caiA	2,26	0,42	-1,79	0,02	4,78	1,34	0,29	1,01	acyl-CoA dehydrogenase
LMW thiols	CymR	SACOL2638	cysJ-cysG	cysG	1,07	1,42	0,49	0,14	2,09	2,68	1,40	1,10	possible sirohydrochlorin ferrochelatase
LMW thiols	CymR	SACOL2639	cysJ-cysG	cysJ	0,68	1,22	0,55	0,00	1,60	2,33	1,46	1,00	sulfite reductase (NADPH) alpha subunit
LMW thiols	CymR	SACOL0557	cysK	cysK	2,32	1,28	-0,92	0,10	4,98	2,43	0,53	1,07	cysteine synthase
LMW thiols	CymR	SACOL2126	luxS	luxS	0,32	-0,10	-0,63	-0,22	1,24	0,93	0,65	0,86	S-ribosylhomocysteine lyase
LMW thiols	CymR	SACOL0184	oppF_2	oppF_2	1,66	1,28	0,05	0,38	3,16	2,43	1,04	1,30	oligopeptide ABC transporter, ATP-binding protein
LMW thiols	CymR	SACOL2034	SACOL2033 yedE	yedE	1,80	1,24	-0,86	-0,29	3,48	2,36	0,55	0,82	hypothetical membrane protein
LMW thiols	CymR	SACOL0157	srpF	srpF	4,84	1,28	-3,66	-0,12	28,67	2,43	0,08	0,92	hypothetical protein
LMW thiols	CymR	SACOL2410	tcyABC	tcyC	2,65	0,42	-2,12	0,08	6,27	1,34	0,23	1,06	ABC transporter, ATP-binding protein
LMW thiols	CymR	SACOL2411	tcyABC	tcyB	2,40	0,25	-2,00	0,13	5,29	1,19	0,25	1,09	ABC transporter, membrane protein
LMW thiols	CymR	SACOL2412	tcyABC	tcyA	2,22	0,40	-1,77	0,03	4,66	1,32	0,29	1,02	amino acid ABC transporter, binding protein
LMW thiols	CymR	SACOL0454	tcyP	tcyP	2,01	0,57	-0,78	0,63	4,03	1,48	0,58	1,55	DAACS family dicarboxylate/amino acid:sodium or proton symporter
Nucleotide biosynthesis	NrdR	SACOL2634	nrdDG	nrdG	1,06	1,88	0,62	-0,18	2,08	3,68	1,54	0,88	anaerobic ribonucleotide reductase small subunit
Nucleotide biosynthesis	NrdR	SACOL2635	nrdDG	nrdD	1,36	0,85	0,31	0,80	2,57	1,80	1,24	1,74	anaerobic ribonucleotide reductase large subunit
Nucleotide biosynthesis	PurR	SACOL0018	purA	purA	-1,22	-1,29	0,15	0,21	0,43	0,41	1,11	1,16	adenylosuccinate synthase
Nucleotide biosynthesis	PurR	SACOL1969	purB	purB	-1,58	-1,15	0,56	0,12	0,33	0,45	1,47	1,09	adenylosuccinate lyase
Nucleotide biosynthesis	PurR	SACOL1073	purEKCSQLFMNHD	purE	-1,24	-1,02	0,04	-0,14	0,42	0,49	1,03	0,91	5-(carboxyamino)imidazole ribonucleotide mutase
Nucleotide biosynthesis	PurR	SACOL1074	purEKCSQLFMNHD	purK	-1,24	-0,86	-0,02	-0,40	0,42	0,55	0,99	0,76	5-(carboxyamino)imidazole ribonucleotide synthase
Nucleotide biosynthesis	PurR	SACOL1075	purEKCSQLFMNHD	purC	-0,95	-1,00	-0,22	-0,17	0,52	0,50	0,86	0,89	phosphoribosylaminoimidazolesuccinocarboxamide synthase
Nucleotide biosynthesis	PurR	SACOL1076	purEKCSQLFMNHD	purS	-1,04	-1,03	-0,25	-0,25	0,49	0,49	0,84	0,84	phosphoribosylformylglycinamidine synthase
Nucleotide biosynthesis	PurR	SACOL1077	purEKCSQLFMNHD	purQ	-0,75	-1,08	-0,51	-0,11	0,59	0,47	0,70	0,93	phosphoribosylformylglycinamidine synthase subunit I
Nucleotide biosynthesis	PurR	SACOL1078	purEKCSQLFMNHD	purL	-0,62	-1,03	-0,57	-0,16	0,65	0,49	0,67	0,90	phosphoribosylformylglycinamidine synthase subunit II
Nucleotide biosynthesis	PurR	SACOL1079	purEKCSQLFMNHD	purF	-0,59	-0,90	-0,42	-0,10	0,66	0,54	0,75	0,93	amidophosphoribosyltransferase
Nucleotide biosynthesis	PurR	SACOL1080	purEKCSQLFMNHD	purM	-0,58	-0,81	-0,30	-0,07	0,67	0,57	0,81	0,95	phosphoribosylformylglycinamidine cyclo-ligase
Nucleotide biosynthesis	PurR	SACOL1082	purEKCSQLFMNHD	purH	-0,58	-0,82	-0,14	0,11	0,67	0,57	0,91	1,08	phosphoribosylaminoimidazolecarboxamide formyltransferase/IMP cyclohydrolase
Nucleotide biosynthesis	PurR	SACOL1083	purEKCSQLFMNHD	purD	-0,57	-0,72	0,08	0,22	0,67	0,61	1,06	1,16	phosphoribosylamine--glycine ligase
Nucleotide biosynthesis	PyrR	SACOL1211	uraA pyrB pyrC carA carB pyrF pyrE SACOL1218	uraA	1,41	-0,33	-0,51	1,13	2,66	0,80	0,70	2,19	NCS family uracil:cation symporter
Nucleotide biosynthesis	PyrR	SACOL1212	uraA pyrB pyrC carA carB pyrF pyrE SACOL1218	pyrB	1,72	-0,03	-0,69	0,99	3,30	0,98	0,62	1,99	aspartate carbamoyltransferase
Nucleotide biosynthesis	PyrR	SACOL1213	uraA pyrB pyrC carA carB pyrF pyrE SACOL1218	pyrC	2,06	0,21	-0,89	0,92	4,18	1,16	0,54	1,89	dihydroorotase
Nucleotide biosynthesis	PyrR	SACOL1214	uraA pyrB pyrC carA carB pyrF pyrE SACOL1218	carA	2,34	0,57	-0,90	0,84	5,06	1,48	0,54	1,79	carbamoyl-phosphate synthase (glutamine-hydrolyzing), small subunit
Nucleotide biosynthesis	PyrR	SACOL1215	uraA pyrB pyrC carA carB pyrF pyrE SACOL1218	carB	2,51	1,33	-0,79	0,38	5,70	2,51	0,58	1,30	carbamoyl-phosphate synthase (glutamine-hydrolyzing), large subunit
Nucleotide biosynthesis	PyrR	SACOL1216	uraA pyrB pyrC carA carB pyrF pyrE SACOL1218	pyrF	2,68	1,75	-0,77	0,12	6,39	3,36	0,59	1,09	orotidine-5'-phosphate decarboxylase
Nucleotide biosynthesis	PyrR	SACOL1217	uraA pyrB pyrC carA carB pyrF pyrE SACOL1218	pyrE	2,75	1,89	-0,88	-0,04	6,73	3,71	0,54	0,97	orotate phosphoribosyltransferase
Nucleotide biosynthesis	PyrR	SACOL1218	uraA pyrB pyrC carA carB pyrF pyrE SACOL1218	SACOL1218	1,91	1,08	-0,86	-0,06	3,76	2,11	0,55	0,96	hypothetical protein
Protein folding	CtsR	SACOL0979	clpB	clpB	2,29	-0,73	-2,84	0,04	4,90	0,60	0,14	1,03	S14 family endopeptidase ClpB
Protein folding	CtsR	SACOL0833	clpP	clpP	4,08	1,30	-2,81	-0,06	16,96	2,46	0,14	0,96	S14 family endopeptidase ClpP
Protein folding	CtsR	SACOL0567	ctsR mcsA mcsB clpC	ctsR	4,00	0,54	-3,64	-0,28	16,00	1,45	0,08	0,82	CtsR family transcriptional regulator
Protein folding	CtsR	SACOL0568	ctsR mcsA mcsB clpC	mcsA	4,05	0,47	-3,63	-0,12	16,60	1,39	0,08	0,92	hypothetical protein
Protein folding	CtsR	SACOL0569	ctsR mcsA mcsB clpC	mcsB	4,30	0,54	-3,72	-0,04	19,66	1,45	0,08	0,97	ATP:guanido phosphotransferase
Protein folding	CtsR	SACOL0570	ctsR mcsA mcsB clpC	clpC	4,41	0,89	-3,66	-0,21	21,25	1,85	0,08	0,86	AAA family ATP-binding protein
Protein folding	HrcA	SACOL2016	groEL-groES	groEL	2,52	0,25	-2,92	-0,71	5,73	1,19	0,13	0,61	chaperone GroEL
Protein folding	HrcA	SACOL2017	groEL-groES	groES	2,42	0,26	-2,75	-0,64	5,34	1,20	0,15	0,64	chaperone GroES
Protein folding	HrcA	SACOL1636	hrcA-grpE-dnaKJ	dnaJ	2,38	0,19	-2,43	-0,29	5,21	1,14	0,19	0,82	chaperone DnaJ
Protein folding	HrcA	SACOL1637	hrcA-grpE-dnaKJ	dnaK	2,75	0,20	-2,74	-0,26	6,73	1,15	0,15	0,84	chaperone DnaK
Protein folding	HrcA	SACOL1638	hrcA-grpE-dnaKJ	grpE	2,49	0,25	-2,59	-0,41	5,63	1,19	0,17	0,75	chaperone GrpE
Protein folding	HrcA	SACOL1639	hrcA-grpE-dnaKJ	hrcA	2,53	0,17	-2,56	-0,27	5,79	1,13	0,17	0,83	heat shock transcriptional repressor HrcA
Redox regulons	CstR	SACOL0063	cstAB sqr	cstA	5,14	1,99	-2,94	0,16	35,26	3,97	0,13	1,12	rhodanese domain-containing protein
Redox regulons	CstR	SACOL0064	cstAB sqr	cstB	4,93	1,85	-2,57	0,53	30,42	3,61	0,17	1,44	hydroxyacylglutathione hydrolase
Redox regulons	CstR	SACOL0061	cstR tauE	tauE	2,13	1,28	-0,89	-0,03	4,37	2,43	0,54	0,98	hypothetical membrane protein
Redox regulons	CstR	SACOL0062	cstR tauE	cstR	2,29	1,26	-1,33	-0,22	4,90	2,39	0,40	0,86	repressor of hydrogen sulfide detoxification cstAB-sqr operon
Redox regulons	CzrA	SACOL2137	czrAB	czrA	2,53	2,53	0,68	0,64	5,79	5,78	1,60	1,56	transcriptional regulator CzrA
Redox regulons	CzrA	SACOL2138	czrAB	czrB	2,97	3,09	0,17	0,10	7,83	8,51	1,13	1,07	CDF family cation diffusion facilitator CzrB

FunCat	Regulon	Locus	Operon	Gene symbol	log2-fold change (M-value)				Fold changes				Function
					WT MHQ/ WT co	Δ*mhqR* MHQ/ Δ*mhqR* co	Δ*mhqR* MHQ/ WT MHQ	Δ*mhqR* co/ WT co	WT MHQ/ WT co	Δ*mhqR* MHQ/ Δ*mhqR* co	Δ*mhqR* MHQ/ WT MHQ	Δ*mhqR* co/ WT co	
Redox regulons	Fur	SACOL1385	citB	citB	1,83	0,27	-1,47	0,08	3,56	1,21	0,36	1,06	aconitate hydratase
Redox regulons	Fur	SACOL0414	efeOBU	efeO	5,22	2,44	-2,49	0,19	37,21	5,43	0,18	1,14	iron (Fe2+)-binding lipoprotein
Redox regulons	Fur	SACOL0415	efeOBU	efeB	5,13	2,41	-2,29	0,36	35,10	5,31	0,20	1,28	iron (Fe2+)-dependent Dyp family peroxidase
Redox regulons	Fur	SACOL0416	efeOBU	efeU	4,56	2,62	-2,03	-0,12	23,65	6,15	0,24	0,92	OFeT family oxidase-dependent iron (Fe2+) transporter
Redox regulons	Fur	SACOL0874	fbiB	fbiB	1,01	0,43	0,15	0,70	2,02	1,35	1,11	1,62	possible nitroreductase
Redox regulons	Fur	SACOL0704	fhuABG	fhuA	0,99	0,08	-0,25	0,65	1,99	1,06	0,84	1,57	iron (Fe3+) ABC transporter, ATP-binding protein
Redox regulons	Fur	SACOL0705	fhuABG	fhuB	1,29	0,43	0,07	0,90	2,44	1,35	1,05	1,87	iron (Fe3+) ABC transporter, membrane protein
Redox regulons	Fur	SACOL0706	fhuABG	fhuG	1,56	0,64	0,18	1,07	2,95	1,56	1,13	2,10	iron (Fe3+) ABC transporter, membrane protein
Redox regulons	Fur	SACOL2277	fhuD2	fhuD2	0,74	0,56	0,65	0,81	1,67	1,47	1,57	1,75	iron (Fe+3) ABC transporter, iron-binding protein
Redox regulons	Fur	SACOL1952	ftnA	ftn	1,72	3,52	1,37	-0,41	3,29	11,47	2,58	0,75	ferritin
Redox regulons	Fur	SACOL2165	htsABC	htsC	1,46	1,02	0,50	0,95	2,76	2,03	1,41	1,93	heme ABC transporter, permease HtsC
Redox regulons	Fur	SACOL2166	htsABC	htsB	1,15	0,89	0,59	0,86	2,22	1,85	1,51	1,82	heme ABC transporter, permease HtsB
Redox regulons	Fur	SACOL2167	htsABC	htsA	0,00	0,10	0,85	0,74	1,00	1,07	1,80	1,67	heme ABC transporter, heme-binding protein HtsA
Redox regulons	Fur	SACOL1781	isdH	isdH	1,67	0,76	-1,18	-0,28	3,17	1,69	0,44	0,82	cell wall surface anchored protein
Redox regulons	Fur	SACOL0100	sbnABCDEFGHI	sbnA	2,21	1,89	-0,21	0,06	4,62	3,71	0,86	1,04	possible pyridoxal-phosphate dependent enzyme
Redox regulons	Fur	SACOL0101	sbnABCDEFGHI	sbnB	2,73	1,90	-0,20	0,38	6,63	3,73	0,87	1,30	possible ornithine cyclodeaminase
Redox regulons	Fur	SACOL0102	sbnABCDEFGHI	sbnC	2,64	2,39	0,11	0,28	6,24	5,24	1,08	1,21	IucA/IucC family siderophore biosynthesis protein
Redox regulons	Fur	SACOL0103	sbnABCDEFGHI	sbnD	2,63	2,46	0,21	0,29	6,19	5,50	1,16	1,22	MFS family major facilitator transporter
Redox regulons	Fur	SACOL0104	sbnABCDEFGHI	sbnE	2,56	2,12	0,24	0,61	5,91	4,35	1,18	1,53	IucA/IucC family siderophore biosynthesis protein
Redox regulons	Fur	SACOL0105	sbnABCDEFGHI	sbnF	2,25	2,38	0,27	0,10	4,75	5,21	1,21	1,07	IucA/IucC family siderophore biosynthesis protein
Redox regulons	Fur	SACOL0106	sbnABCDEFGHI	sbnG	2,38	2,18	0,47	0,57	5,20	4,53	1,39	1,48	HpcH/HpaI family aldolase
Redox regulons	Fur	SACOL0107	sbnABCDEFGHI	sbnH	2,32	2,02	0,46	0,66	4,98	4,06	1,38	1,58	diaminopimelate decarboxylase
Redox regulons	Fur	SACOL0108	sbnABCDEFGHI	sbnI	1,89	1,96	0,47	0,36	3,70	3,89	1,39	1,28	hypothetical protein
Redox regulons	Fur	SACOL0796	sstABCD	sstA	1,17	-0,11	-0,45	0,78	2,25	0,93	0,73	1,72	siderophore-Fe(III) ABC transporter permease
Redox regulons	Fur	SACOL0797	sstABCD	sstB	0,99	-0,21	-0,36	0,75	1,98	0,86	0,78	1,68	siderophore-Fe(III) ABC transporter permease
Redox regulons	Fur	SACOL0798	sstABCD	sstC	1,14	-0,11	-0,07	1,05	2,20	0,93	0,95	2,07	siderophore-Fe(III) ABC transporter, ATP-binding protein
Redox regulons	Fur	SACOL0799	sstABCD	sstD	1,04	-0,37	-0,44	0,93	2,06	0,77	0,74	1,91	siderophore-Fe(III) ABC transporter,lipoprotein
Redox regulons	Fur	SACOL0417	tatAC	tatC	3,03	2,43	-1,12	-0,54	8,16	5,39	0,46	0,69	Tat family twin arginine targeting transporter TatC
Redox regulons	Fur	SACOL0418	tatAC	tatA	2,88	1,96	-1,30	-0,40	7,37	3,89	0,41	0,76	Tat family twin arginine targeting transporter TatA
Redox regulons	HypR	SACOL0640	hypR merA	merA	2,77	0,29	-2,21	0,21	6,81	1,22	0,22	1,16	pyridine nucleotide-disulfide reductase
Redox regulons	HypR	SACOL0641	hypR merA	hypR	2,67	0,50	-2,20	-0,11	6,35	1,41	0,22	0,93	Rrf2-family repressor of the hypR-merA operon
Redox regulons	MhqR	SACOL2529	mhqR mhqD mhqE	mhqD	6,07	-0,59	2,44	9,32	67,27	0,66	5,43	639,15	phospholipase/carboxylesterase
Redox regulons	MhqR	SACOL2530	mhqR mhqD mhqE	mhqE	5,82	-0,65	2,94	9,80	56,55	0,64	7,67	891,44	dioxygnease
Redox regulons	MhqR	SACOL2531	mhqR mhqD mhqE	mhqR	5,13	-0,37	1,86	7,69	34,98	0,77	3,63	206,50	MarR family transcriptional regulator
Redox regulons	NsrR	SACOL0219	SACOL0219 hmp	SACOL0219	2,43	0,71	-1,87	-0,15	5,37	1,64	0,27	0,90	hypothetical protein
Redox regulons	NsrR	SACOL0220	SACOL0219 hmp	hmp	2,22	0,53	-2,07	-0,37	4,67	1,44	0,24	0,77	flavohemoprotein
Redox regulons	PerR	SACOL0451	ahpCF	ahpF	4,10	4,16	-0,77	-0,78	17,09	17,88	0,59	0,58	peroxiredoxin subunit F
Redox regulons	PerR	SACOL0452	ahpCF	ahpC	4,26	4,17	-0,89	-0,75	19,10	18,00	0,54	0,59	peroxiredoxin subunit C
Redox regulons	PerR	SACOL1920	bcp SACOL1920	SACOL1920	2,64	1,01	-1,78	-0,16	6,25	2,01	0,29	0,90	D-3-phosphoglycerate dehydrogenase
Redox regulons	PerR	SACOL1921	bcp SACOL1920	bcp	2,74	1,03	-1,82	-0,13	6,66	2,04	0,28	0,91	possible peroxiredoxin
Redox regulons	PerR	SACOL2131	dps	dps	4,52	3,84	1,57	2,07	22,89	14,32	2,97	4,20	Dps family stress protein
Redox regulons	PerR	SACOL1368	katA	katA	2,39	1,98	-1,34	-0,90	5,25	3,94	0,40	0,54	catalase
Redox regulons	PerR	SACOL0914	sufCDSUB	sufC	0,64	0,85	0,48	0,26	1,56	1,80	1,39	1,20	FeS assembly ATPase SufC
Redox regulons	PerR	SACOL0915	sufCDSUB	sufD	0,75	0,93	0,67	0,47	1,69	1,91	1,59	1,39	FeS assembly protein SufD
Redox regulons	PerR	SACOL0916	sufCDSUB	sufS	0,88	1,02	0,70	0,53	1,84	2,03	1,62	1,44	SufS subfamily cysteine desulfurase
Redox regulons	PerR	SACOL0917	sufCDSUB	sufU	0,96	1,13	0,71	0,52	1,95	2,19	1,64	1,43	NifU family SUF system FeS assembly protein
Redox regulons	PerR	SACOL0918	sufCDSUB	sufB	1,20	1,29	0,53	0,44	2,30	2,45	1,44	1,36	FeS assembly protein SufB
Redox regulons	PerR	SACOL1762	tpx	tpx	4,01	2,15	-1,95	-0,14	16,16	4,44	0,26	0,91	possible peroxiredoxin
Redox regulons	PerR	SACOL0829	trxB	trxB	3,59	1,70	-1,97	-0,12	12,04	3,25	0,26	0,92	thioredoxin-disulfide reductase
Redox regulons	QsrR	SACOL0408	catE SACOL0409 azoR1	catE	6,19	2,12	-3,40	0,59	72,80	4,35	0,09	1,51	possible lactoylglutathione lyase
Redox regulons	QsrR	SACOL0409	catE SACOL0409 azoR1	SACOL0409	6,58	2,33	-3,31	0,87	95,92	5,03	0,10	1,83	possible alkanal monooxygenase (FMN-linked)
Redox regulons	QsrR	SACOL0410	catE SACOL0409 azoR1	azoR1	6,92	2,91	-3,16	0,78	121,02	7,52	0,11	1,72	possible FMN reductase
Redox regulons	QsrR	SACOL2533	catE2	catE2	8,12	4,42	-2,41	1,38	279,07	21,41	0,19	2,60	possible dioxygenase
Redox regulons	QsrR	SACOL2534	frp	frp	5,46	2,46	-2,19	0,74	44,01	5,50	0,22	1,67	FMN reductase
Redox regulons	QsrR	SACOL2020	yodC	yodC	4,68	3,47	-0,68	0,52	25,55	11,08	0,62	1,43	possible nitroreductase
Redox regulons	Rex	SACOL0744	graA cydC	graA	0,80	0,26	-0,24	0,28	1,75	1,20	0,85	1,21	ABC superfamily ATP binding cassette transporter, ABC protein
Redox regulons	Rex	SACOL0745	graA cydC	cydC	1,47	0,41	-0,55	0,47	2,77	1,33	0,68	1,39	ABC superfamily ATP binding cassette transporter, ABC protein
Redox regulons	Rex	SACOL0204	pflB pflA	pflB	0,56	0,47	0,59	0,64	1,47	1,39	1,51	1,56	formate acetyltransferase
Redox regulons	Rex	SACOL0205	pflB pflA	pflA	2,91	1,28	0,58	2,07	7,51	2,43	1,49	4,20	pyruvate formate-lyase-activating enzyme
Redox regulons	Rex	SACOL2491	SACOL2491	SACOL2491	-0,93	0,01	1,09	0,14	0,53	1,01	2,13	1,10	hypothetical protein
Redox regulons	TetR	SACOL2588	SACOL2588 SACOL2589	SACOL2588	9,07	3,90	-3,58	0,18	535,92	14,93	0,08	1,13	hypothetical protein
Redox regulons	TetR	SACOL2589	SACOL2588 SACOL2589	SACOL2589	8,99	3,94	-4,38	-0,22	509,65	15,35	0,05	0,86	hypothetical protein
Redox regulons	Zur	SACOL0948	mnmC	mnmC	2,92	1,18	-1,74	0,11	7,59	2,27	0,30	1,08	hypothetical protein
Redox regulons	Zur	SACOL2598	SACOL2600 feoB_3 cobW_3	cobW_3	0,90	0,56	-0,79	-0,42	1,87	1,47	0,58	0,75	cobalamin (vitamin B12) biosynthesis protein
Redox regulons	Zur	SACOL2599	SACOL2600 feoB_3 cobW_3	feoB_3	1,30	0,78	-0,99	-0,43	2,46	1,72	0,50	0,74	FeoB family ferrous iron (Fe2+) uptake protein
Redox regulons	Zur	SACOL2600	SACOL2600 feoB_3 cobW_3	SACOL2600	1,16	0,53	-1,31	-0,60	2,24	1,44	0,40	0,66	hypothetical protein
Redox regulons	Zur	SACOL0491	yciC	yciC	2,02	0,53	-1,83	-0,35	4,04	1,44	0,28	0,78	cobalamin (vitamin B12) biosynthesis protein
Redox regulons	Zur	SACOL2403	zinT	zinT	2,19	1,26	-1,24	-0,31	4,57	2,39	0,42	0,81	ABC transporter, binding protein
Redox regulons	Zur	SACOL1611	znuCB zur	zur	0,32	0,33	-0,41	-0,40	1,25	1,26	0,75	0,76	Zn-uptake transcriptional regulator
Redox regulons	Zur	SACOL1612	znuCB zur	znuB	0,73	0,49	-0,13	0,10	1,65	1,40	0,91	1,07	zinc ABC transporter, permease
Redox regulons	Zur	SACOL1613	znuCB zur	znuC	0,88	0,43	-0,26	0,18	1,84	1,35	0,84	1,13	zinc ABC transporter, ATP-binding protein
Stress and Starvation	SigB	SACOL0835	-	SACOL0835	0,84	-0,45	-1,41	-0,15	1,80	0,73	0,38	0,90	hypothetical protein
Stress and Starvation	SigB	SACOL2593	acrR SACOL2594 - SACOL2597	acrR	3,60	2,45	-0,99	0,06	12,12	5,46	0,50	1,04	TetR family transcriptional regulator
Stress and Starvation	SigB	SACOL2594	acrR SACOL2594 - SACOL2597	SACOL2594	3,07	2,10	-1,09	-0,12	8,42	4,29	0,47	0,92	dehydrogenase
Stress and Starvation	SigB	SACOL2596	acrR SACOL2594 - SACOL2597	SACOL2596	1,83	1,08	-1,03	-0,27	3,55	2,11	0,49	0,83	hypothetical protein
Stress and Starvation	SigB	SACOL2597	acrR SACOL2594 - SACOL2597	SACOL2597	1,75	1,11	-0,81	-0,18	3,36	2,16	0,57	0,88	hydrolase
Stress and Starvation	SigB	SACOL0660	adh1	adh1	-1,48	-1,01	0,80	0,23	0,36	0,50	1,74	1,17	alcohol dehydrogenase
Stress and Starvation	SigB	SACOL0136	cap5ABCDEFGHIJKLMNOP	cap5A	-0,80	0,43	0,73	-0,49	0,57	1,35	1,66	0,71	capsular polysaccharide biosynthesis protein Cap5A
Stress and Starvation	SigB	SACOL0137	cap5ABCDEFGHIJKLMNOP	cap5B	-0,52	0,38	0,43	-0,45	0,70	1,30	1,35	0,73	capsular polysaccharide biosynthesis protein Cap5B
Stress and Starvation	SigB	SACOL0138	cap5ABCDEFGHIJKLMNOP	cap5C	-0,56	0,45	0,33	-0,65	0,68	1,37	1,26	0,64	capsular polysaccharide biosynthesis protein Cap5C
Stress and Starvation	SigB	SACOL0140	cap5ABCDEFGHIJKLMNOP	cap5E	-1,62	0,26	1,27	-0,56	0,33	1,20	2,41	0,68	capsular polysaccharide biosynthesis protein Cap5E
Stress and Starvation	SigB	SACOL0141	cap5ABCDEFGHIJKLMNOP	cap5F	-1,52	0,28	0,98	-0,72	0,35	1,21	1,97	0,61	capsular polysaccharide biosynthesis protein Cap5F
Stress and Starvation	SigB	SACOL0143	cap5ABCDEFGHIJKLMNOP	cap5H	-1,65	0,42	1,14	-0,86	0,32	1,34	2,20	0,55	capsular polysaccharide biosynthesis protein Cap5H
Stress and Starvation	SigB	SACOL0144	cap5ABCDEFGHIJKLMNOP	cap5I	-1,51	0,02	0,88	-0,62	0,35	1,01	1,84	0,65	capsular polysaccharide biosynthesis protein Cap5I
Stress and Starvation	SigB	SACOL0145	cap5ABCDEFGHIJKLMNOP	cap5J	-1,09	-0,04	0,55	-0,49	0,47	0,97	1,46	0,71	capsular polysaccharide biosynthesis protein Cap5J
Stress and Starvation	SigB	SACOL0146	cap5ABCDEFGHIJKLMNOP	cap5K	-0,97	0,03	0,75	-0,24	0,51	1,02	1,68	0,85	capsular polysaccharide synthesis protein Cap5K
Stress and Starvation	SigB	SACOL0147	cap5ABCDEFGHIJKLMNOP	cap5L	-0,89	-0,03	0,56	-0,29	0,54	0,98	1,47	0,82	capsular polysaccharide biosynthesis protein Cap5L
Stress and Starvation	SigB	SACOL0148	cap5ABCDEFGHIJKLMNOP	cap5M	-0,65	0,03	0,53	-0,15	0,64	1,02	1,44	0,90	capsular polysaccharide biosynthesis protein Cap5M
Stress and Starvation	SigB	SACOL0149	cap5ABCDEFGHIJKLMNOP	cap5N	-0,77	-0,11	0,48	-0,18	0,58	0,93	1,39	0,88	capsular polysaccharide biosynthesis protein Cap5N
Stress and Starvation	SigB	SACOL0150	cap5ABCDEFGHIJKLMNOP	cap5O	-0,68	0,07	0,45	-0,30	0,62	1,05	1,37	0,81	capsular polysaccharide biosynthesis protein Cap5O
Stress and Starvation	SigB	SACOL0151	cap5ABCDEFGHIJKLMNOP	cap5P	-0,42	0,05	0,03	-0,41	0,75	1,04	1,02	0,75	capsular polysaccharide biosynthesis protein Cap5P
Stress and Starvation	SigB	SACOL2563	clpL	clpL	0,65	0,22	-0,43	0,00	1,57	1,16	0,74	1,00	S14 family endopeptidase Clp
Stress and Starvation	SigB	SACOL2576	crtNMQIO	crtN	-1,33	0,07	1,59	0,19	0,40	1,05	3,01	1,14	squalene synthase
Stress and Starvation	SigB	SACOL2577	crtNMQIO	crtM	-1,16	0,20	1,48	0,13	0,45	1,15	2,79	1,09	squalene desaturase
Stress and Starvation	SigB	SACOL2578	crtNMQIO	crtQ	-0,74	0,27	1,05	0,06	0,60	1,21	2,07	1,04	glycosyltransferase
Stress and Starvation	SigB	SACOL2579	crtNMQIO	crtI	-0,52	0,07	0,72	0,14	0,70	1,05	1,65	1,10	phytoene dehydrogenase
Stress and Starvation	SigB	SACOL2580	crtNMQIO	crtO	-1,18	-0,49	0,55	-0,15	0,44	0,71	1,46	0,90	hypothetical membrane protein
Stress and Starvation	SigB	SACOL0764	csbB	csbB	0,94	0,10	-0,02	0,78	1,92	1,07	0,99	1,72	possible glycosyltransferase
Stress and Starvation	SigB	SACOL0985	eap	eap	1,16	0,94	-0,33	-0,11	2,23	1,92	0,80	0,93	extracellular adherence protein Eap
Stress and Starvation	SigB	SACOL1871	epiGEFPDCBA	epiG	0,84	0,16	0,40	1,01	1,79	1,12	1,32	2,01	lantibiotic epidermin immunity protein F
Stress and Starvation	SigB	SACOL1872	epiGEFPDCBA	epiE	0,60	-0,10	0,27	0,87	1,51	0,93	1,21	1,83	epidermin ABC transporter, membrane protein
Stress and Starvation	SigB	SACOL1873	epiGEFPDCBA	epiF	-0,50	-0,62	0,55	0,62	0,71	0,65	1,46	1,54	epidermin ABC transporter, ATP-binding protein
Stress and Starvation	SigB	SACOL1874	epiGEFPDCBA	epiP	-0,88	-0,96	0,53	0,54	0,54	0,51	1,44	1,45	S8A family lantibiotic epidermin serine protease
Stress and Starvation	SigB	SACOL1875	epiGEFPDCBA	epiD	0,32	0,19	0,18	0,19	1,25	1,14	1,13	1,14	lantibiotic epidermin biosynthesis protein EpiD
Stress and Starvation	SigB	SACOL1876	epiGEFPDCBA	epiC	0,34	0,41	0,60	0,39	1,27	1,33	1,52	1,31	lantibiotic epidermin biosynthesis protein EpiC
Stress and Starvation	SigB	SACOL1877	epiGEFPDCBA	epiB	-0,26	0,66	0,59	-0,27	0,83	1,58	1,51	0,83	lantibiotic epidermin biosynthesis protein EpiB
Stress and Starvation	SigB	SACOL1878	epiGEFPDCBA	epiA	-1,09	-0,60	-0,12	-0,48	0,47	0,66	0,92	0,72	lantibiotic epidermin EpiA
Stress and Starvation	SigB	SACOL0962	glpQ	glpQ	-1,73	-1,35	0,16	-0,17	0,30	0,39	1,12	0,89	glycerophosphoryl diester phosphodiesterase
Stress and Starvation	SigB	SACOL2669	gtfAB secA2 asp3 asp2 asp1	gtfB	0,07	0,42	0,38	0,03	1,05	1,34	1,30	1,02	hypothetical protein
Stress and Starvation	SigB	SACOL2670	gtfAB secA2 asp3 asp2 asp1	gtfA	0,37	0,61	0,40	0,15	1,29	1,53	1,32	1,11	glycosyl transferase, group 1 family protein
Stress and Starvation	SigB	SACOL2671	gtfAB secA2 asp3 asp2 asp1	secA2	0,47	0,62	0,43	0,27	1,39	1,54	1,35	1,21	Sec family Type I general secretory pathway protein SecA2
Stress and Starvation	SigB	SACOL2672	gtfAB secA2 asp3 asp2 asp1	asp3	1,30	1,02	-0,42	-0,15	2,46	2,03	0,75	0,90	accessory secretory protein Asp3
Stress and Starvation	SigB	SACOL2673	gtfAB secA2 asp3 asp2 asp1	asp2	1,33	1,16	-0,41	-0,24	2,52	2,23	0,75	0,85	accessory secretory protein Asp2
Stress and Starvation	SigB	SACOL2674	gtfAB secA2 asp3 asp2 asp1	asp1	1,10	1,16	-0,36	-0,41	2,14	2,23	0,78	0,75	accessory secretory protein Asp1
Stress and Starvation	SigB	SACOL0597	hchA	hchA	5,07	2,86	-2,23	-0,17	33,50	7,26	0,21	0,89	ThiJ/PfpI family protein
Stress and Starvation	SigB	SACOL2011	ktrB	ktrB	1,03	0,68	-1,28	-0,88	2,05	1,60	0,41	0,54	sodium transport family protein
Stress and Starvation	SigB	SACOL2197	map-w	map-w	-1,03	-0,78	1,36	1,01	0,49	0,58	2,57	2,01	truncated MHC class II analog protein
Stress and Starvation	SigB	SACOL0636	mvaK1 mvaD mvaK2	mvaK1	-0,73	-0,58	0,32	0,16	0,60	0,67	1,25	1,12	mevalonate kinase
Stress and Starvation	SigB	SACOL0637	mvaK1 mvaD mvaK2	mvaD	-0,68	-0,60	0,40	0,32	0,63	0,66	1,32	1,25	diphosphomevalonate decarboxylase
Stress and Starvation	SigB	SACOL0638	mvaK1 mvaD mvaK2	mvaK2	-0,43	-0,52	0,30	0,38	0,74	0,70	1,23	1,30	phosphomevalonate kinase
Stress and Starvation	SigB	SACOL0616	nagB hxlAB	nagB	1,96	1,12	-1,14	-0,30	3,90	2,17	0,45	0,81	glucosamine-6-phosphate deaminase
Stress and Starvation	SigB	SACOL0617	nagB hxlAB	hxlA	2,09	1,20	-1,08	-0,21	4,27	2,30	0,47	0,86	possible 3-hexulose-6-phosphate synthase
Stress and Starvation	SigB	SACOL0618	nagB hxlAB	hxlB	2,13	1,14	-1,16	-0,19	4,39	2,20	0,45	0,88	glucose-6-phosphate isomerase
Stress and Starvation	SigB	SACOL2721	nixA	nixA	2,02	1,95	-0,13	-0,06	4,05	3,86	0,91	0,96	NiCoT family nickel (Ni2+)-cobalt (Co2+) transporter
Stress and Starvation	SigB	SACOL0086	norC hipO	norC	0,66	0,58	0,05	0,12	1,58	1,49	1,04	1,09	possible MFS family major facilitator transporter
Stress and Starvation	SigB	SACOL2389	nreA nreB nreC narI narJ narH narG	nreC	-1,27	-0,79	0,36	-0,12	0,41	0,58	1,28	0,92	transcriptional regulatory protein
Stress and Starvation	SigB	SACOL2390	nreA nreB nreC narI narJ narH narG	nreB	-1,09	-0,79	0,41	0,10	0,47	0,58	1,33	1,07	sensory box histidine kinase
Stress and Starvation	SigB	SACOL2391	nreA nreB nreC narI narJ narH narG	nreA	-1,56	-1,03	0,63	0,08	0,34	0,49	1,55	1,06	hypothetical protein
Stress and Starvation	SigB	SACOL2392	nreA nreB nreC narI narJ narH narG	narI	-1,71	-1,35	0,33	-0,03	0,31	0,39	1,26	0,98	respiratory nitrate reductase subunit gamma
Stress and Starvation	SigB	SACOL2393	nreA nreB nreC narI narJ narH narG	narJ	-1,62	-2,07	-0,70	-0,17	0,33	0,24	0,62	0,89	respiratory nitrate reductase subunit delta
Stress and Starvation	SigB	SACOL2394	nreA nreB nreC narI narJ narH narG	narH	-2,43	-2,35	-0,34	-0,06	0,19	0,20	0,79	0,96	respiratory nitrate reductase subunit beta
Stress and Starvation	SigB	SACOL2395	nreA nreB nreC narI narJ narH narG	narG	-4,38	-2,88	-0,34	-0,54	0,05	0,14	0,79	0,69	respiratory nitrate reductase subunit alpha
Stress and Starvation	SigB	SACOL0860	nuc	nuc	-1,44	0,08	1,02	-0,33	0,37	1,06	2,03	0,80	thermonuclease precursor
Stress and Starvation	SigB	SACOL2459	pnbA	pnbA	-0,90	-0,98	0,75	0,78	0,53	0,51	1,68	1,72	carboxylesterase
Stress and Starvation	SigB	SACOL0770	queEDC	queE	-0,81	0,98	1,60	-0,17	0,57	1,97	3,03	0,89	organic radical-activating protein
Stress and Starvation	SigB	SACOL0771	queEDC	queD	-0,72	1,01	1,79	0,08	0,61	2,01	3,46	1,06	possible 6-pyruvoyltetrahydropterin synthase
Stress and Starvation	SigB	SACOL0772	queEDC	queC	-0,87	1,15	1,94	-0,05	0,55	2,22	3,84	0,97	ExsB family post-transcriptional regulator
Stress and Starvation	SigB	SACOL1289	rbfA truB ribC	rbfA	0,64	0,50	0,09	0,21	1,55	1,41	1,06	1,16	ribosome-binding factor A
Stress and Starvation	SigB	SACOL1290	rbfA truB ribC	truB	0,94	0,54	-0,42	-0,03	1,92	1,45	0,75	0,98	pseudouridylate synthase
Stress and Starvation	SigB	SACOL1291	rbfA truB ribC	ribC	1,66	0,41	-1,10	0,13	3,15	1,33	0,47	1,09	bifunctional riboflavin kinase/FAD synthase RibC
Stress and Starvation	SigB	SACOL0085	SACOL0085 SACOL0086	hipO	-0,58	-0,05	0,80	0,24	0,67	0,97	1,74	1,18	peptidase, M20/M25/M40 family
Stress and Starvation	SigB	SACOL0089	SACOL0089	SACOL0089	0,83	0,80	0,89	0,86	1,78	1,74	1,85	1,82	myosin-cross-reactive antigen
Stress and Starvation	SigB	SACOL0156	SACOL0156	SACOL0156	-1,29	-0,81	0,93	0,42	0,41	0,57	1,91	1,34	hypothetical protein
Stress and Starvation	SigB	SACOL0399	SACOL0399	SACOL0399	0,97	-0,28	-0,73	0,49	1,95	0,82	0,60	1,40	possible dehydrogenase
Stress and Starvation	SigB	SACOL0455	SACOL0455 SACOL0456 SACOL0457	SACOL0455	0,09	0,42	0,19	-0,14	1,06	1,34	1,14	0,91	hypothetical protein
Stress and Starvation	SigB	SACOL0456	SACOL0455 SACOL0456 yflT	SACOL0456	1,28	0,59	-0,80	-0,11	2,43	1,51	0,57	0,93	hypothetical protein
Stress and Starvation	SigB	SACOL0457	SACOL0455 SACOL0456 yflT	yflT	0,99	0,32	-0,38	0,27	1,98	1,25	0,77	1,21	putative heat induced stress protein
Stress and Starvation	SigB	SACOL0630	SACOL0630	SACOL0630	-1,38	-0,32	1,04	-0,02	0,38	0,80	2,06	0,99	APC family amino acid-polyamine-organocation transporter
Stress and Starvation	SigB	SACOL0671	SACOL0671	SACOL0671	-0,76	-1,07	0,00	0,28	0,59	0,48	1,00	1,21	hydrolase
Stress and Starvation	SigB	SACOL0736	SACOL0736 SACOL0737 SACOL0738	SACOL0736	-0,69	-0,08	0,46	-0,11	0,62	0,95	1,38	0,93	possible GNAT family acetyltransferase
Stress and Starvation	SigB	SACOL0737	SACOL0736 SACOL0737 SACOL0738	SACOL0737	-1,06	-0,78	0,06	-0,22	0,48	0,58	1,04	0,86	possible lipoprotein
Stress and Starvation	SigB	SACOL0738	SACOL0736 SACOL0737 SACOL0738	SACOL0738	-0,41	-0,15	-0,39	-0,67	0,75	0,90	0,76	0,63	hypothetical protein
Stress and Starvation	SigB	SACOL0830	SACOL0830 SACOL0831 whiA	SACOL0830	1,70	0,19	-1,27	0,22	3,25	1,14	0,41	1,16	hypothetical protein
Stress and Starvation	SigB	SACOL0831	SACOL0830 SACOL0831 whiA	SACOL0831	1,49	0,18	-1,04	0,26	2,81	1,13	0,49	1,20	hypothetical protein
Stress and Starvation	SigB	SACOL0832	SACOL0830 SACOL0831 whiA	whiA	1,67	0,33	-1,01	0,31	3,18	1,26	0,50	1,24	hypothetical protein
Stress and Starvation	SigB	SACOL0834	SACOL0834	SACOL0834	-0,83	-0,42	0,65	0,23	0,56	0,75	1,57	1,17	hypothetical protein

FunCat	Regulon	Locus	Operon	Gene symbol	log2-fold change (M-value) WT MHQ/ WT co	ΔmhqR MHQ/ ΔmhqR co	ΔmhqR MHQ/ WT MHQ	ΔmhqR co/ WT co	Fold changes WT MHQ/ WT co	ΔmhqR MHQ/ ΔmhqR co	ΔmhqR MHQ/ WT MHQ	ΔmhqR co/ WT co	Function
**Stress and Starvation**	**SigB**	**SACOL0921**	**SACOL0921 fabK**	**SACOL0921**	**1,68**	**1,17**	**-0,34**	**0,13**	**3,19**	**2,25**	**0,79**	**1,09**	**CBS domain protein**
**Stress and Starvation**	**SigB**	**SACOL0922**	**SACOL0921 fabK**	**fabK**	**1,91**	**1,51**	**-0,57**	**-0,18**	**3,75**	**2,85**	**0,67**	**0,88**	**2-nitropropane dioxygenase**
**Stress and Starvation**	**SigB**	**SACOL1046**	**SACOL1046**	**SACOL1046**	**1,34**	**0,23**	**-0,96**	**0,13**	**2,54**	**1,17**	**0,51**	**1,09**	**hypothetical protein**
**Stress and Starvation**	**SigB**	**SACOL1788**	**SACOL1788 SACOL1789**	**SACOL1788**	**1,27**	**0,87**	**-0,22**	**0,18**	**2,42**	**1,83**	**0,86**	**1,13**	**hypothetical protein**
**Stress and Starvation**	**SigB**	**SACOL1789**	**SACOL1788 SACOL1789**	**SACOL1789**	**0,99**	**0,78**	**-0,28**	**-0,07**	**1,99**	**1,72**	**0,82**	**0,95**	**possible general stress protein**
Stress and Starvation	SigB	SACOL1802	SACOL1802 rsuA SACOL1804	SACOL1802	-0,38	-0,31	0,22	0,14	0,77	0,81	1,16	1,10	hypothetical protein
Stress and Starvation	SigB	SACOL1803	SACOL1802 rsuA SACOL1804	rsuA	-0,56	-0,40	0,30	0,14	0,68	0,76	1,23	1,10	ribosomal small subunit pseudouridine synthase A
Stress and Starvation	SigB	SACOL1804	SACOL1802 rsuA SACOL1804	SACOL1804	-0,73	-0,47	0,40	0,13	0,60	0,72	1,32	1,09	polysaccharide biosynthesis protein
**Stress and Starvation**	**SigB**	**SACOL1911**	**SACOL1911 SACOL1912**	**SACOL1911**	**0,97**	**0,37**	**-0,48**	**0,11**	**1,96**	**1,29**	**0,72**	**1,08**	**hypothetical protein**
Stress and Starvation	SigB	SACOL1912	SACOL1911 SACOL1912	SACOL1912	0,55	0,31	-0,47	-0,23	1,47	1,24	0,72	0,85	glucosamine-6-phosphate isomerase
**Stress and Starvation**	**SigB**	**SACOL2012**	**SACOL2012**	**SACOL2012**	**1,11**	**0,25**	**-0,72**	**0,12**	**2,15**	**1,19**	**0,61**	**1,09**	**GNAT family acetyltransferase**
Stress and Starvation	SigB	SACOL2076	SACOL2076 SACOL2077 csoR	SACOL2076	-1,46	-1,08	0,83	0,43	0,36	0,47	1,78	1,35	hypothetical protein
Stress and Starvation	SigB	SACOL2077	SACOL2076 SACOL2077 csoR	SACOL2077	-0,71	-0,68	0,73	0,66	0,61	0,62	1,66	1,58	hypothetical protein
Stress and Starvation	SigB	SACOL2078	SACOL2076 SACOL2077 csoR	csoR	-0,86	-0,54	0,92	0,56	0,55	0,69	1,89	1,47	Copper-sensing transcriptional repressor RicR
**Stress and Starvation**	**SigB**	**SACOL2114**	**SACOL2114**	**aldA**	**2,36**	**1,80**	**-0,41**	**0,14**	**5,15**	**3,48**	**0,75**	**1,10**	**aldehyde dehydrogenase**
**Stress and Starvation**	**SigB**	**SACOL2132**	**SACOL2132**	**SACOL2132**	**2,20**	**0,98**	**-1,08**	**0,12**	**4,61**	**1,97**	**0,47**	**1,09**	**hypothetical protein**
**Stress and Starvation**	**SigB**	**SACOL2168**	**SACOL2168 SACOL2169 SACOL2170**	**SACOL2168**	**2,50**	**1,56**	**-0,27**	**0,66**	**5,65**	**2,95**	**0,83**	**1,58**	**hypothetical protein**
**Stress and Starvation**	**SigB**	**SACOL2169**	**SACOL2168 SACOL2169 SACOL2170**	**SACOL2169**	**1,75**	**1,01**	**-0,20**	**0,53**	**3,37**	**2,01**	**0,87**	**1,44**	**possible luc family aerobactin synthesis protein**
**Stress and Starvation**	**SigB**	**SACOL2170**	**SACOL2168 SACOL2169 SACOL2170**	**SACOL2170**	**2,24**	**1,14**	**-0,60**	**0,47**	**4,72**	**2,20**	**0,66**	**1,39**	**MFS family major facilitator transporter**
**Stress and Starvation**	**SigB**	**SACOL2519**	**SACOL2519**	**SACOL2519**	**1,88**	**1,50**	**-1,06**	**-0,67**	**3,68**	**2,83**	**0,48**	**0,63**	**hypothetical protein**
Stress and Starvation	SigB	SACOL2621	SACOL2621	SACOL2621	-1,92	-0,61	2,02	0,68	0,26	0,66	4,06	1,60	hypothetical membrane protein
Stress and Starvation	SigB	SACOL2631	SACOL2631	SACOL2631	-2,22	-1,07	0,97	-0,18	0,21	0,48	1,96	0,88	possible metallo-beta-lactamase
Stress and Starvation	SigB	SACOL2717	SACOL2717	SACOL2717	-1,20	-0,75	0,71	0,25	0,43	0,59	1,64	1,19	hypothetical protein
**Stress and Starvation**	**SigB**	**SACOL0672**	**sarA**	**sarA**	**1,33**	**0,23**	**-1,14**	**-0,05**	**2,51**	**1,17**	**0,45**	**0,97**	**staphylococcal accessory regulator A**
Stress and Starvation	SigB	SACOL0096	sarS	sarS	-1,58	-1,07	-0,07	-0,53	0,33	0,48	0,95	0,69	staphylococcal accessory regulator S
Stress and Starvation	SigB	SACOL2054	sigB rsbW rsbV rsbU	sigB	-0,59	0,12	0,75	0,05	0,67	1,09	1,68	1,04	DNA-directed RNA polymerase sigma subunit FliA
Stress and Starvation	SigB	SACOL2055	sigB rsbW rsbV rsbU	rsbW	-0,69	0,09	0,62	-0,17	0,62	1,06	1,54	0,89	anti-sigma B factor
Stress and Starvation	SigB	SACOL2056	sigB rsbW rsbV rsbU	rsbV	-0,71	-0,05	0,85	0,18	0,61	0,97	1,80	1,13	anti-sigma B factor antagonist
Stress and Starvation	SigB	SACOL2057	sigB rsbW rsbV rsbU	rsbU	-0,58	-0,08	0,53	0,02	0,67	0,95	1,44	1,01	sigma factor B regulator
**Stress and Starvation**	**SigB**	**SACOL0118**	**sodA1**	**sodM**	**1,43**	**1,14**	**-0,59**	**-0,28**	**2,69**	**2,20**	**0,66**	**0,82**	**superoxide dismutase**
Stress and Starvation	SigB	SACOL2303	suhB	suhB	-1,11	-1,50	0,29	0,64	0,46	0,35	1,22	1,56	inositol monophosphatase family protein
**Stress and Starvation**	**SigB**	**SACOL2711**	**yceI**	**yceI**	**6,64**	**4,50**	**-0,47**	**1,66**	**100,06**	**22,63**	**0,72**	**3,16**	**hypothetical protein**
Stress and Starvation	SigB	SACOL2136	ylbE	ylbE	-0,79	-0,20	0,92	0,31	0,58	0,87	1,89	1,24	putative NAD dependent epimerase
Stress and Starvation	SigB	SACOL2300	yrhD fdhA	yrhD	-0,63	0,30	0,73	-0,19	0,65	1,23	1,66	0,88	hypothetical protein
Stress and Starvation	SigB	SACOL2301	yrhD fdhA	fdhA	0,25	0,57	0,04	-0,28	1,19	1,48	1,03	0,82	formate dehydrogenase alpha subunit
Sugar catabolism	ArlR	SACOL1048	SACOL1048	SACOL1048	2,45	0,21	-2,50	-0,22	5,48	1,16	0,18	0,86	acetyltransferase
Sugar catabolism	CcpA	SACOL1758	ald2	ald2	-1,58	-1,47	0,50	0,37	0,33	0,36	1,41	1,29	alanine dehydrogenase
Sugar catabolism	CcpA	SACOL0154	aldA	aldA	3,56	0,32	-2,99	0,18	11,79	1,25	0,13	1,13	aldehyde dehydrogenase
Sugar catabolism	CcpA	SACOL0599	capD	capD	-1,69	-1,09	0,54	-0,07	0,31	0,47	1,45	0,95	NAD dependent epimerase/dehydratase family protein
Sugar catabolism	CcpA	SACOL1786	ccpA	ccpA	2,82	0,60	-1,94	0,24	7,05	1,52	0,26	1,18	catabolite control protein A
Sugar catabolism	CcpA	SACOL0707	dhaKLM	dhaK	1,10	0,10	-0,50	0,47	2,14	1,07	0,71	1,39	dihydroxyacetone kinase subunit DhaK
Sugar catabolism	CcpA	SACOL0708	dhaKLM	dhaL	1,32	0,25	-0,42	0,61	2,50	1,19	0,75	1,53	DAK2 domain-containing protein DhaL
Sugar catabolism	CcpA	SACOL0709	dhaKLM	dhaM	1,28	0,20	-0,39	0,62	2,43	1,15	0,76	1,54	PTS-dependent dihydroxyacetone kinase phosphotransferase subunit DhaM
Sugar catabolism	CcpA	SACOL0213	fadDEX	fadD	-0,65	-1,41	-0,22	0,34	0,64	0,38	0,86	1,27	acyl-CoA dehydrogenase
Sugar catabolism	CcpA	SACOL0214	fadDEX	fadE	-0,92	-1,16	-0,44	-0,12	0,53	0,45	0,74	0,92	long-chain-fatty-acid--CoA ligase
Sugar catabolism	CcpA	SACOL0215	fadDEX	fadX	-2,20	-1,74	-0,52	-0,91	0,22	0,30	0,70	0,53	propionate CoA-transferase
Sugar catabolism	CcpA	SACOL2316	glvC	glvC	0,67	0,44	-0,51	-0,27	1,59	1,36	0,70	0,83	PTS system, IIBC components
Sugar catabolism	CcpA	SACOL0173	ipdC	ipdC	1,64	0,89	-0,78	-0,03	3,13	1,85	0,58	0,98	thiamine pyrophosphate TPP binding domain-containing protein
Sugar catabolism	CcpA	SACOL0960	rocD gluD	rocD	1,15	-0,43	-1,26	0,30	2,22	0,74	0,42	1,23	ornithine--oxo-acid transaminase
Sugar catabolism	CcpA	SACOL0961	rocD gluD	gudB	0,21	-0,95	-0,85	0,29	1,16	0,52	0,55	1,22	glutamate dehydrogenase
Sugar catabolism	CcpA	SACOL0176	SACOL0176 murQ murP SACOL0179	SACOL0176	-1,90	-1,37	0,15	-0,39	0,27	0,39	1,11	0,76	hypothetical protein
Sugar catabolism	CcpA	SACOL0177	SACOL0176 murQ murP SACOL0179	murQ	-1,35	-1,53	0,11	0,25	0,39	0,35	1,08	1,19	N-acetylmuramic acid-6-phosphate etherase
Sugar catabolism	CcpA	SACOL0178	SACOL0176 murQ murP SACOL0179	murP	-1,39	-1,49	0,77	0,79	0,38	0,36	1,71	1,73	N-acetylmuramic acid phosphotransfer permease
Sugar catabolism	CcpA	SACOL0179	SACOL0176 murQ murP SACOL0179	SACOL0179	-0,90	-1,03	0,81	0,85	0,54	0,49	1,75	1,80	RpiR family phosphosugar-binding transcriptional regulator
Sugar catabolism	CcpA	SACOL0308	SACOL0308 psuG nupC	SACOL0308	0,59	0,06	-0,45	0,04	1,51	1,04	0,73	1,03	carbohydrate kinase
Sugar catabolism	CcpA	SACOL0309	SACOL0308 psuG nupC	psuG	0,32	-0,26	-0,85	-0,22	1,25	0,84	0,55	0,86	hypothetical protein
Sugar catabolism	CcpA	SACOL0310	SACOL0308 psuG nupC	nupC	1,37	0,10	-0,87	0,30	2,59	1,07	0,55	1,23	nucleoside permease
Sugar catabolism	CcpA	SACOL1158	sdhC sdhA sdhB	sdhC	-0,65	-0,38	0,64	0,35	0,64	0,77	1,56	1,27	succinate dehydrogenase, cytochrome b558 subunit
Sugar catabolism	CcpA	SACOL1159	sdhC sdhA sdhB	sdhA	-0,90	-0,58	0,86	0,51	0,54	0,67	1,82	1,42	succinate dehydrogenase flavoprotein subunit
Sugar catabolism	CcpA	SACOL1160	sdhC sdhA sdhB	sdhB	-1,02	-0,72	1,05	0,71	0,49	0,61	2,07	1,64	succinate dehydrogenase iron-sulfur subunit
Translation	Translation	SACOL0590	rplL3 rpsL rpsG fusA tuf	rplL3	-0,87	-0,13	0,21	-0,53	0,55	0,91	1,16	0,69	possible RNA-binding ribosomal protein
Translation	Translation	SACOL0591	rplL3 rpsL rpsG fusA tuf	rpsL	-0,89	-0,19	0,60	-0,09	0,54	0,88	1,52	0,94	ribosomal protein S12
Translation	Translation	SACOL0592	rplL3 rpsL rpsG fusA tuf	rpsG	-0,86	-0,18	0,72	0,03	0,55	0,88	1,65	1,02	ribosomal protein S7
Translation	Translation	SACOL0594	rplL3 rpsL rpsG fusA tuf	tuf	-0,27	-0,03	0,35	0,11	0,83	0,98	1,27	1,08	elongation factor EF1A
Transport	ScrR	SACOL2376	scrA	scrA	-1,54	-1,09	1,36	0,89	0,34	0,47	2,57	1,85	PTS family sucrose porter component IIBC
Transport	TcaR	SACOL2668	sasF	sasF	0,64	0,56	0,49	0,57	1,56	1,47	1,40	1,48	LPXTG family cell wall surface anchor protein
Transport	TraP	SACOL0608	-	sdrC	-2,50	-1,20	0,15	-1,08	0,18	0,44	1,11	0,47	Ser-Asp rich fibrinogen/bone sialoprotein-binding protein SdrC
Transport	TraP	SACOL2023	agrBDCA	agrB	-1,53	-0,58	-0,27	-1,15	0,35	0,67	0,83	0,45	accessory gene regulator protein B
Transport	TraP	SACOL2024	agrBDCA	agrD	-1,24	-0,23	-0,05	-1,01	0,42	0,85	0,97	0,50	accessory gene regulator protein D
Transport	TraP	SACOL2025	agrBDCA	agrC	-0,47	0,07	-0,17	-0,70	0,72	1,05	0,89	0,62	accessory gene regulator protein C
Transport	TraP	SACOL2026	agrBDCA	agrA	-0,08	0,22	-0,13	-0,42	0,94	1,16	0,91	0,75	accessory gene regulator protein A
Transport	TraP	SACOL2652	clfB	clfB	-1,71	-0,18	1,50	-0,02	0,31	0,88	2,83	0,99	clumping factor B
Transport	TraP	SACOL0162	fdh	fdh	1,41	0,63	-0,22	0,53	2,66	1,55	0,86	1,44	formate dehydrogenase
Transport	TraP	SACOL0317	geh	geh	-1,90	-0,53	-0,17	-1,48	0,27	0,69	0,89	0,36	triacylglycerol lipase
Transport	TraP	SACOL2340	gltS	gltS	-0,83	-0,30	-0,81	-1,26	0,56	0,81	0,57	0,42	ESS family glutamate:sodium (Na+) symporter
Transport	TraP	SACOL2194	hysA	hysA	-0,90	-0,81	-0,18	-0,26	0,54	0,57	0,88	0,84	hyaluronate lyase
Transport	TraP	SACOL2688	icaR	icaR	1,24	0,25	-1,86	-0,86	2,36	1,19	0,28	0,55	Ica operon transcriptional regulator R
Transport	TraP	SACOL0486	lpl2 SACOL0487 SACOL0488 SACOL0489	lpl2	-0,73	-0,62	-0,48	-0,57	0,60	0,65	0,72	0,67	staphylococcal tandem lipoprotein
Transport	TraP	SACOL0487	lpl2 SACOL0487 SACOL0488 SACOL0489	SACOL0487	-0,30	-0,53	-0,52	-0,28	0,81	0,69	0,70	0,82	hypothetical protein
Transport	TraP	SACOL0488	lpl2 SACOL0487 SACOL0488 SACOL0489	SACOL0488	-0,23	-0,31	-0,38	-0,29	0,85	0,81	0,77	0,82	hypothetical protein
Transport	TraP	SACOL0489	lpl2 SACOL0487 SACOL0488 SACOL0489	SACOL0489	-0,26	-0,18	-0,43	-0,49	0,84	0,88	0,74	0,71	hypothetical protein
Transport	TraP	SACOL2399	nirR	nirR	-4,94	-2,40	0,58	-0,67	0,03	0,19	1,49	0,63	transcriptional regulator NirR
Transport	TraP	SACOL0078	plc	plc	-0,90	0,33	0,74	-0,42	0,54	1,26	1,67	0,75	phosphatidylinositol diacylglycerol-lyase
Transport	TraP	SACOL0253	rbsKDU	rbsK	-1,04	-0,45	0,63	0,02	0,49	0,73	1,55	1,01	ribokinase
Transport	TraP	SACOL0254	rbsKDU	rbsD	-1,24	-0,47	0,55	-0,20	0,42	0,72	1,46	0,87	ribose ABC transporter, membrane protein
Transport	TraP	SACOL0255	rbsKDU	rbsU	-1,50	-0,62	0,45	-0,43	0,35	0,65	1,37	0,74	DMT superfamily drug/metabolite transporter
Transport	TraP	SACOL0462	SACOL0462	SACOL0462	2,26	1,12	-0,61	0,43	4,80	2,17	0,66	1,35	hypothetical protein
Transport	TraP	SACOL0678	SACOL0678 mnhABCDEFG	SACOL0678	2,80	2,89	-0,09	-0,12	6,99	7,41	0,94	0,92	possible bacteriophage integrase
Transport	TraP	SACOL0679	SACOL0678 mnhABCDEFG	mnhA	1,48	1,68	0,13	-0,06	2,80	3,20	1,09	0,96	CPA3 family monovalent cation (K+ or Na+):proton (H+) antiporter-3, MnhA subunit
Transport	TraP	SACOL0680	SACOL0678 mnhABCDEFG	mnhB	0,57	1,40	0,79	-0,02	1,49	2,64	1,73	0,99	CPA3 family monovalent cation (K+ or Na+):proton (H+) antiporter-3, MnhB subunit
Transport	TraP	SACOL0681	SACOL0678 mnhABCDEFG	mnhC	0,05	1,11	0,85	-0,17	1,04	2,16	1,80	0,89	CPA3 family monovalent cation (K+ or Na+):proton (H+) antiporter-3, MnhC subunit
Transport	TraP	SACOL0682	SACOL0678 mnhABCDEFG	mnhD	0,46	1,13	0,68	0,01	1,38	2,19	1,60	1,01	CPA3 family monovalent cation (K+ or Na+):proton (H+) antiporter-3, MnhD subunit
Transport	TraP	SACOL0684	SACOL0678 mnhABCDEFG	mnhE	0,61	1,00	0,48	0,08	1,52	2,00	1,39	1,06	CPA3 family monovalent cation (K+ or Na+):proton (H+) antiporter-3, MnhE subunit
Transport	TraP	SACOL0685	SACOL0678 mnhABCDEFG	mnhF	0,60	1,06	0,51	0,05	1,52	2,08	1,42	1,04	CPA3 family monovalent cation (K+ or Na+):proton (H+) antiporter-3, MnhF subunit
Transport	TraP	SACOL0686	SACOL0678 mnhABCDEFG	mnhG	0,63	1,13	0,62	0,14	1,54	2,19	1,54	1,10	CPA3 family monovalent cation (K+ or Na+):proton (H+) antiporter-3, MnhG subunit
Transport	TraP	SACOL1763	SACOL1763 thiI nifZ	SACOL1763	-0,39	-0,44	0,25	0,28	0,76	0,74	1,19	1,21	hypothetical protein
Transport	TraP	SACOL1764	SACOL1763 thiI nifZ	thiI	-0,67	-0,67	0,33	0,32	0,63	0,63	1,26	1,25	thiamine biosynthesis protein
Transport	TraP	SACOL1765	SACOL1763 thiI nifZ	nifZ	-0,78	-0,70	0,36	0,26	0,58	0,62	1,28	1,20	cysteine desulfurase
Transport	TraP	SACOL0609	sdrD	sdrD	-1,68	-0,67	-0,79	-1,71	0,31	0,63	0,58	0,31	Ser-Asp rich fibrinogen/bone sialoprotein-binding protein SdrD
Transport	TraP	SACOL0095	spa	spa	-1,87	-1,26	-0,42	-0,58	0,27	0,42	0,75	0,67	immunoglobulin G binding protein A
Transport	TraP	SACOL0478	ssl11 SACOL0479	ssl11	-0,61	0,10	0,56	-0,11	0,66	1,07	1,47	0,93	superantigen-like protein
Transport	TraP	SACOL0479	ssl11 SACOL0479	SACOL0479	0,66	0,55	-0,30	-0,19	1,58	1,46	0,81	0,88	hypothetical protein
Transport	TreR	SACOL0516	trePCR	treP	-1,34	-0,15	1,17	-0,01	0,40	0,90	2,25	0,99	PTS family trehalose (tre) porter component IIBC
Transport	TreR	SACOL0517	trePCR	treC	-0,72	-0,08	0,94	0,29	0,61	0,95	1,92	1,22	alpha, alpha-phosphotrehalase
Virulence	AgrA	SACOL1325	bsaA	bsaA	1,33	-0,42	-1,55	0,16	2,51	0,75	0,34	1,12	glutathione peroxidase
Virulence	AgrA	SACOL1295	ftsK SACOL1296 SACOL1297 SACOL1298 phbB	ftsK	0,68	0,26	-0,14	0,27	1,60	1,20	0,91	1,21	FtsK/SpoIIIE family protein
Virulence	AgrA	SACOL1296	ftsK SACOL1296 SACOL1297 SACOL1298 phbB	SACOL1296	0,94	0,48	0,06	0,49	1,92	1,39	1,04	1,40	GntR family transcriptional regulator
Virulence	AgrA	SACOL1297	ftsK SACOL1296 SACOL1297 SACOL1298 phbB	SACOL1297	-0,02	0,41	0,65	0,20	0,99	1,33	1,57	1,15	hypothetical protein
Virulence	AgrA	SACOL1298	ftsK SACOL1296 SACOL1297 SACOL1298 phbB	SACOL1298	0,22	0,41	0,53	0,32	1,17	1,33	1,44	1,25	M16 family peptidase
Virulence	AgrA	SACOL1299	ftsK SACOL1296 SACOL1297 SACOL1298 phbB	phbB	0,45	0,52	0,43	0,33	1,37	1,43	1,35	1,26	putative 3-oxoacyl-acyl carrier protein reductase
Virulence	AgrA	SACOL1207	gloA2	gloA2	1,60	0,52	-1,14	-0,08	3,03	1,43	0,45	0,95	glyoxalase/bleomycin resistance protein/dioxygenase superfamily protein
Virulence	AgrA	SACOL1665	greA udk SACOL1667 SACOL1668 SACOL1669	greA	0,65	0,54	0,07	0,17	1,57	1,45	1,05	1,13	transcription elongation factor GreA
Virulence	AgrA	SACOL1666	greA udk SACOL1667 SACOL1668 SACOL1669	udk	0,50	0,39	0,08	0,19	1,41	1,31	1,06	1,14	uridine kinase
Virulence	AgrA	SACOL1667	greA udk SACOL1667 SACOL1668 SACOL1669	SACOL1667	0,40	0,31	0,20	0,28	1,32	1,24	1,15	1,21	U32 family peptidase
Virulence	AgrA	SACOL1668	greA udk SACOL1667 SACOL1668 SACOL1669	SACOL1668	0,24	0,32	0,44	0,35	1,18	1,25	1,36	1,27	U32 family peptidase
Virulence	AgrA	SACOL1669	greA udk SACOL1667 SACOL1668 SACOL1669	SACOL1669	0,18	0,51	0,49	0,13	1,13	1,42	1,40	1,09	O-methyltransferase
Virulence	AgrA	SACOL0949	mnhG mnhF mnhE mnhD mnhC mnhB mnhA	mnhG	1,08	0,47	-0,45	0,14	2,11	1,39	0,73	1,10	monovalent cation/H+ antiporter subunit G
Virulence	AgrA	SACOL0950	mnhG mnhF mnhE mnhD mnhC mnhB mnhA	mnhF	1,14	0,37	-0,53	0,22	2,20	1,29	0,69	1,16	monovalent cation/H+ antiporter subunit F
Virulence	AgrA	SACOL0951	mnhG mnhF mnhE mnhD mnhC mnhB mnhA	mnhE	1,08	0,32	-0,57	0,17	2,11	1,25	0,67	1,13	monovalent cation/H+ antiporter subunit E
Virulence	AgrA	SACOL0952	mnhG mnhF mnhE mnhD mnhC mnhB mnhA	mnhD	1,07	0,27	-0,62	0,16	2,10	1,21	0,65	1,12	monovalent cation/H+ antiporter subunit D
Virulence	AgrA	SACOL0953	mnhG mnhF mnhE mnhD mnhC mnhB mnhA	mnhC	0,89	0,11	-0,47	0,30	1,85	1,08	0,72	1,23	monovalent cation/H+ antiporter subunit C
Virulence	AgrA	SACOL0954	mnhG mnhF mnhE mnhD mnhC mnhB mnhA	mnhB	0,87	0,15	-0,48	0,22	1,83	1,11	0,72	1,16	monovalent cation/H+ antiporter subunit B
Virulence	AgrA	SACOL0955	mnhG mnhF mnhE mnhD mnhC mnhB mnhA	mnhA	0,62	-0,03	-0,65	-0,01	1,53	0,98	0,64	0,99	monovalent cation/H+ antiporter subunit A
Virulence	AgrA	SACOL0494	mpsBA	mpsA	1,32	-0,82	-1,84	0,23	2,49	0,57	0,28	1,17	NADH dehydrogenase subunit 5
Virulence	AgrA	SACOL0495	mpsBA	mpsB	2,56	-0,72	-2,43	0,71	5,88	0,61	0,19	1,64	hypothetical protein
Virulence	AgrA	SACOL1419	pepF	pepF	1,41	1,17	-0,62	-0,37	2,65	2,25	0,65	0,77	Oligoendopeptidase F
Virulence	AgrA	SACOL2569	rocA	rocA	1,37	-0,30	-1,17	0,47	2,59	0,81	0,44	1,39	1-pyrroline-5-carboxylate dehydrogenase
Virulence	AgrA	SACOL0545	rplY pth mfd SACOL0548 mazG SACOL0550 divIC	rplY	-1,34	-0,26	0,92	-0,16	0,39	0,84	1,89	0,90	50S ribosomal protein L25/general stress protein rplY
Virulence	AgrA	SACOL0546	rplY pth mfd SACOL0548 mazG SACOL0550 divIC	pth	0,36	0,50	-0,07	-0,20	1,29	1,41	0,95	0,87	peptidyl-tRNA hydrolase
Virulence	AgrA	SACOL0547	rplY pth mfd SACOL0548 mazG SACOL0550 divIC	mfd	0,74	0,56	-0,20	-0,02	1,67	1,47	0,87	0,99	transcription-repair coupling factor
Virulence	AgrA	SACOL0548	rplY pth mfd SACOL0548 mazG SACOL0550 divIC	SACOL0548	0,82	0,63	-0,43	-0,25	1,76	1,55	0,74	0,84	polysaccharide biosynthesis protein
Virulence	AgrA	SACOL0549	rplY pth mfd SACOL0548 mazG SACOL0550 divIC	mazG	0,99	0,73	-0,48	-0,22	1,98	1,66	0,72	0,86	nucleoside triphosphate pyrophosphohydrolase
Virulence	AgrA	SACOL0550	rplY pth mfd SACOL0548 mazG SACOL0550 divIC	SACOL0550	0,81	0,52	-0,46	-0,16	1,76	1,43	0,73	0,90	S4 domain-containing protein
Virulence	AgrA	SACOL0551	rplY pth mfd SACOL0548 mazG SACOL0550 divIC	divIC	0,85	0,57	-0,42	-0,14	1,81	1,48	0,75	0,91	cell-division protein divIC
Virulence	AgrA	SACOL0196	SACOL0196 mviM iolE	SACOL0196	3,28	1,06	-1,08	1,01	9,75	2,08	0,47	2,01	Gfo/Idh/MocA family oxidoreductase
Virulence	AgrA	SACOL0197	SACOL0196 mviM iolE	mviM	2,86	1,33	-0,88	0,58	7,25	2,51	0,54	1,49	NADH-dependent dehydrogenase
Virulence	AgrA	SACOL0198	SACOL0196 mviM iolE	iolE	2,77	1,32	-0,68	0,68	6,84	2,50	0,62	1,60	sugar phosphate isomerase/epimerase
Virulence	AgrA	SACOL0595	SACOL0595	SACOL0595	1,99	0,41	-1,52	0,04	3,97	1,33	0,35	1,03	M20/M25/M40 family peptidase
Virulence	AgrA	SACOL0779	SACOL0779 recQ1	SACOL0779	1,48	0,87	-0,41	0,20	2,80	1,83	0,75	1,15	ABC transporter ATP-binding protein
Virulence	AgrA	SACOL0780	SACOL0779 recQ1	recQ1	1,41	0,87	-0,10	0,42	2,65	1,83	0,93	1,34	ATP-dependent DNA helicase RecQ1
Virulence	AgrA	SACOL1500	SACOL1500 - SACOL1503 aroA aroB aroC	SACOL1500	1,79	0,54	-1,20	0,04	3,45	1,45	0,44	1,03	hypothetical protein
Virulence	AgrA	SACOL1501	SACOL1500 - SACOL1503 aroA aroB aroC	SACOL1501	0,70	0,57	-0,19	-0,07	1,63	1,48	0,88	0,95	hypothetical protein
Virulence	AgrA	SACOL1502	SACOL1500 - SACOL1503 aroA aroB aroC	SACOL1502	0,73	0,58	-0,04	0,09	1,65	1,49	0,97	1,06	hypothetical protein
Virulence	AgrA	SACOL1503	SACOL1500 - SACOL1503 aroA aroB aroC	SACOL1503	0,51	0,27	-0,07	0,16	1,43	1,21	0,95	1,12	hypothetical protein
Virulence	AgrA	SACOL1504	SACOL1500 - SACOL1503 aroA aroB aroC	aroA	0,13	0,17	0,25	0,21	1,10	1,13	1,19	1,16	3-phosphoshikimate 1-carboxyvinyltransferase
Virulence	AgrA	SACOL1505	SACOL1500 - SACOL1503 aroA aroB aroC	aroB	-0,28	0,21	0,52	0,02	0,82	1,16	1,43	1,01	3-dehydroquinate synthase
Virulence	AgrA	SACOL1506	SACOL1500 - SACOL1503 aroA aroB aroC	aroC	-0,57	-0,07	0,44	-0,07	0,67	0,95	1,36	0,95	chorismate synthase
Virulence	AgrA	SACOL1531	SACOL1531	SACOL1531	-0,68	-0,42	0,31	0,04	0,62	0,75	1,24	1,03	hypothetical protein
Virulence	AgrA	SACOL2330	SACOL2330	SACOL2330	2,04	0,44	-1,80	-0,21	4,11	1,36	0,29	0,86	hypothetical protein
Virulence	AgrA	SACOL2368	SACOL2368 iruO	SACOL2368	2,70	0,77	-1,83	0,05	6,49	1,71	0,28	1,04	acetyltransferase
Virulence	AgrA	SACOL2369	SACOL2368 iruO	iruO	2,22	0,39	-1,89	-0,09	4,65	1,31	0,27	0,94	pyridine nucleotide-disulfide oxidoreductase
Virulence	AgrA	SACOL2471	SACOL2471 cntF cntD cnzC cntB cntA	SACOL2471	1,44	0,83	-0,57	0,04	2,71	1,78	0,67	1,03	transporter
Virulence	AgrA	SACOL2472	SACOL2471 cntF cntD cnzC cntB cntA	cntF	2,10	1,26	-0,79	0,06	4,30	2,39	0,58	1,04	peptide ABC transporter ATP-binding protein
Virulence	AgrA	SACOL2473	SACOL2471 cntF cntD cnzC cntB cntA	cntD	2,21	1,53	-0,90	-0,20	4,63	2,89	0,54	0,87	peptide ABC transporter ATP-binding protein
Virulence	AgrA	SACOL2474	SACOL2471 cntF cntD cnzC cntB cntA	cnzC	2,43	1,07	-1,12	0,20	5,40	2,10	0,46	1,15	peptide ABC transporter permease
Virulence	AgrA	SACOL2475	SACOL2471 cntF cntD cnzC cntB cntA	cntB	3,22	1,56	-1,68	-0,01	9,35	2,95	0,31	0,99	peptide ABC transporter permease
Virulence	AgrA	SACOL2476	SACOL2471 cntF cntD cnzC cntB cntA	cntA	3,02	1,52	-1,54	-0,01	8,12	2,87	0,34	0,99	peptide ABC transporter peptide-binding protein

FunCat	Regulon	Locus	Operon	Gene symbol	log2-fold change (M-value)				Fold changes				Function
					WT MHQ/ WT co	Δ*mhqR* MHQ/ Δ*mhqR* co	Δ*mhqR* MHQ/ WT MHQ	Δ*mhqR* co/ WT co	WT MHQ/ WT co	Δ*mhqR* MHQ/ Δ*mhqR* co	Δ*mhqR* MHQ/ WT MHQ	Δ*mhqR* co/ WT co	
Virulence	AgrA	SACOL2544	sdaAA sdaAB pfoR	sdaAA	0,84	-0,61	-0,47	0,94	1,79	0,66	0,72	1,92	L-serine dehydratase, iron-sulfur-dependent subunit alpha
Virulence	AgrA	SACOL2545	sdaAA sdaAB pfoR	sdaAB	0,70	-0,61	-0,31	0,95	1,62	0,66	0,81	1,93	L-serine dehydratase, iron-sulfur-dependent subunit beta
Virulence	AgrA	SACOL2546	sdaAA sdaAB pfoR	pfoR	0,66	-0,72	-0,33	0,98	1,58	0,61	0,80	1,97	hypothetical protein
Virulence	AgrA	SACOL1864	splF splE splD splC splB splA	splF	2,47	0,95	-0,07	0,85	5,55	1,93	0,95	1,80	serine protease SplF
Virulence	AgrA	SACOL1865	splF splE splD splC splB splA	splE	1,87	1,21	-0,43	0,07	3,67	2,31	0,74	1,05	serine protease SplE
Virulence	AgrA	SACOL1866	splF splE splD splC splB splA	splD	2,45	0,23	-0,45	1,04	5,48	1,17	0,73	2,06	serine protease SplD
Virulence	AgrA	SACOL1867	splF splE splD splC splB splA	splC	1,75	0,55	-0,54	0,41	3,37	1,46	0,69	1,33	serine protease SplC
Virulence	AgrA	SACOL1868	splF splE splD splC splB splA	splB	1,32	0,76	-0,04	0,31	2,49	1,69	0,97	1,24	serine protease SplB
Virulence	AgrA	SACOL1869	splF splE splD splC splB splA	splA	0,94	0,64	-0,34	-0,06	1,92	1,56	0,79	0,96	serine protease SplA
Virulence	AgrA	SACOL1448	sucB sucA	sucB	-0,60	-1,53	-0,34	0,54	0,66	0,35	0,79	1,45	dihydrolipoamide succinyltransferase
Virulence	AgrA	SACOL1449	sucB sucA	sucA	-0,81	-1,50	-0,45	0,21	0,57	0,35	0,73	1,16	2-oxoglutarate dehydrogenase E1 component
Virulence	AgrA	SACOL0626	thiD1	pdxK	2,10	0,74	-1,43	-0,09	4,30	1,67	0,37	0,94	phosphomethylpyrimidine kinase
Virulence	AgrA	SACOL1676	trmU iscS	mnmA	2,09	0,81	-1,01	0,26	4,26	1,75	0,50	1,20	tRNA-specific 2-thiouridylase MnmA
Virulence	AgrA	SACOL1677	trmU iscS	iscS	1,90	0,79	-1,01	0,08	3,72	1,73	0,50	1,06	class V aminotransferase
Virulence	AgrA	SACOL0003	yaaA recF gyrB gyrA	yaaA	1,31	0,87	-0,23	0,19	2,48	1,83	0,85	1,14	hypothetical protein
Virulence	AgrA	SACOL0004	yaaA recF gyrB gyrA	recF	0,95	0,66	-0,30	-0,02	1,93	1,58	0,81	0,99	recombination protein F
Virulence	AgrA	SACOL0005	yaaA recF gyrB gyrA	gyrB	0,69	0,33	-0,11	0,24	1,62	1,26	0,93	1,18	DNA gyrase, B subunit
Virulence	AgrA	SACOL0006	yaaA recF gyrB gyrA	gyrA	0,57	0,30	-0,18	0,08	1,48	1,23	0,88	1,06	DNA gyrase, A subunit
Virulence	MgrA	SACOL1018	agcS	agcS	-0,66	-0,01	0,22	-0,43	0,63	0,99	1,16	0,74	sodium/alanine symporter
Virulence	MgrA	SACOL0115	cap8H cps2H SACOL0117	cap8H	-1,58	0,16	0,46	-0,89	0,33	1,12	1,38	0,54	glycosyltransferase
Virulence	MgrA	SACOL0116	cap8H cps2H SACOL0117	cps2H	-0,81	0,07	0,73	-0,12	0,57	1,05	1,66	0,92	hypothetical protein
Virulence	MgrA	SACOL0117	cap8H cps2H SACOL0117	SACOL0117	-0,65	-0,47	0,13	-0,04	0,64	0,72	1,09	0,97	polysaccharide biosynthesis protein
Virulence	MgrA	SACOL0856	clfA	clfA	1,90	0,48	-0,51	0,87	3,74	1,39	0,70	1,83	fibrinogen-binding protein
Virulence	MgrA	SACOL2535	ddh	ddh	1,62	0,60	-0,80	0,19	3,08	1,52	0,57	1,14	D-lactate dehydrogenase
Virulence	MgrA	SACOL0858	emp	emp	-1,38	-0,16	0,88	-0,30	0,38	0,90	1,84	0,81	secretory extracellular matrix and plasma binding protein
Virulence	MgrA	SACOL2620	gabT	gabT	-1,15	-0,24	0,22	-0,66	0,45	0,85	1,16	0,63	4-aminobutyrate transaminase
Virulence	MgrA	SACOL0113	galE cap5M2	galE	-2,52	-1,09	0,12	-1,14	0,17	0,47	1,09	0,45	UDP-glucose 4-epimerase
Virulence	MgrA	SACOL0114	galE cap5M2	cap5M2	-1,39	-0,97	-0,79	-0,81	0,38	0,51	0,58	0,57	capsular polysaccharide biosynthesis protein
Virulence	MgrA	SACOL1349	gLY1	gLY1	1,33	0,04	-1,28	-0,01	2,51	1,03	0,41	0,99	aromatic amino acid beta-eliminating lyase/threonine aldolase
Virulence	MgrA	SACOL0526	holB SACOL0527 yabA	holB	0,83	0,68	-0,05	0,09	1,78	1,60	0,97	1,06	DNA-directed DNA polymerase III delta' subunit
Virulence	MgrA	SACOL0527	holB SACOL0527 yabA	SACOL0527	-0,08	0,39	1,09	0,58	0,95	1,31	2,13	1,49	hypothetical protein
Virulence	MgrA	SACOL0528	holB SACOL0527 yabA	yabA	-0,08	0,52	1,25	0,62	0,95	1,43	2,38	1,54	DNA replication intiation control protein YabA
Virulence	MgrA	SACOL1777	htrA1	htrA1	1,00	0,49	-1,18	-0,66	2,00	1,40	0,44	0,63	Trypsin-like serine endoprotease
Virulence	MgrA	SACOL2660	isaB	isaB	1,09	-0,49	-1,74	-0,20	2,13	0,71	0,30	0,87	immunodominant antigen B
Virulence	MgrA	SACOL0820	lysM	lysM	0,94	0,94	-0,07	-0,07	1,92	1,92	0,95	0,95	peptidoglycan hydrolysis related protein
Virulence	MgrA	SACOL1051	menF menD menH menB	menF	0,34	-0,24	-0,40	0,17	1,27	0,85	0,76	1,13	isochorismate synthase
Virulence	MgrA	SACOL1052	menF menD menH menB	menD	0,35	-0,08	-0,01	0,41	1,28	0,95	0,99	1,33	2-oxoglutarate decarboxylase
Virulence	MgrA	SACOL1053	menF menD menH menB	menH	0,75	0,21	-0,10	0,43	1,68	1,16	0,93	1,35	S33 family peptidase
Virulence	MgrA	SACOL1054	menF menD menH menB	menB	1,34	0,61	-0,47	0,24	2,52	1,53	0,72	1,18	naphthoate synthase
Virulence	MgrA	SACOL0428	metEFCI	metE	-0,67	0,15	-0,17	-0,95	0,63	1,11	0,89	0,52	5-methyltetrahydropteroyltriglutamate-- homocysteine S-methyltransferase
Virulence	MgrA	SACOL0429	metEFCI	metF	-1,06	0,05	0,09	-0,96	0,48	1,04	1,06	0,51	methylenetetrahydrofolate reductase/homocysteine S-methyltransferase
Virulence	MgrA	SACOL0430	metEFCI	metC	-1,70	-0,23	0,40	-0,91	0,31	0,85	1,32	0,53	bifunctional cystathionine gamma-lyase/gamma-synthase
Virulence	MgrA	SACOL0431	metEFCI	metI	-2,05	-1,15	0,51	-0,31	0,24	0,45	1,42	0,81	bifunctional cystathionine gamma-lyase/gamma-synthase
Virulence	MgrA	SACOL2261	moaA mobA moaD moaE mobB moeA	moaA	1,37	0,45	-1,06	-0,14	2,59	1,37	0,48	0,91	molybdenum (Mo2+) cofactor biosynthesis protein A
Virulence	MgrA	SACOL2262	moaA mobA moaD moaE mobB moeA	mobA	1,31	0,30	-1,03	-0,04	2,48	1,23	0,49	0,97	molybdenum (Mo2+) cofactor biosynthesis protein A
Virulence	MgrA	SACOL2263	moaA mobA moaD moaE mobB moeA	moaD	1,13	0,21	-1,01	-0,10	2,19	1,16	0,50	0,93	molybdopterin synthase small subunit
Virulence	MgrA	SACOL2264	moaA mobA moaD moaE mobB moeA	moaE	1,14	0,17	-1,04	-0,08	2,20	1,13	0,49	0,95	molybdopterin synthase large subunit
Virulence	MgrA	SACOL2265	moaA mobA moaD moaE mobB moeA	mobB	0,82	0,01	-0,96	-0,15	1,77	1,01	0,51	0,90	molybdopterin-guanine dinucleotide biosynthesis protein B
Virulence	MgrA	SACOL2266	moaA mobA moaD moaE mobB moeA	moeA	0,61	-0,07	-0,68	-0,02	1,52	0,95	0,62	0,99	molybdopterin biosynthesis protein MoeA
Virulence	MgrA	SACOL1195	mraY murD divIB	mraY	-1,20	-0,24	0,86	-0,11	0,43	0,85	1,82	0,93	phospho-N-acetylmuramoyl-pentapeptide- transferase
Virulence	MgrA	SACOL1196	mraY murD divIB	murD	-0,99	-0,51	0,52	0,04	0,50	0,70	1,43	1,03	UDP-N-acetylmuramoylalanine--D-glutamate ligase
Virulence	MgrA	SACOL1197	mraY murD divIB	divIB	-0,77	-0,51	0,20	-0,05	0,59	0,70	1,15	0,97	cell division protein FtsQ
Virulence	MgrA	SACOL0754	norA	norA	0,91	0,27	-0,90	-0,25	1,88	1,21	0,54	0,84	MFS family major facilitator transporter
Virulence	MgrA	SACOL2609	phnB	phnB	1,13	0,01	-1,10	0,00	2,18	1,01	0,47	1,00	3-demethylubiquinone-9 3-methyltransferase
Virulence	MgrA	SACOL2109	ptpB SACOL2108 hemK prfA tdk	hemK	-1,79	-0,80	1,16	0,16	0,29	0,57	2,23	1,12	HemK family methyltransferase
Virulence	MgrA	SACOL2110	ptpB SACOL2108 hemK prfA tdk	prfA	-1,94	-0,87	1,21	0,13	0,26	0,55	2,31	1,09	peptide chain release factor RF1
Virulence	MgrA	SACOL2111	ptpB SACOL2108 hemK prfA tdk	tdk	-1,93	-0,91	1,12	0,09	0,26	0,53	2,17	1,06	thymidine kinase
Virulence	MgrA	SACOL2119	pyrG	pyrG	-1,82	-0,94	1,17	0,29	0,28	0,52	2,25	1,22	CTP synthase
Virulence	MgrA	SACOL0217	SACOL0217	SACOL0217	1,24	0,13	-0,82	0,26	2,36	1,09	0,57	1,20	ABC transporter, binding protein
Virulence	MgrA	SACOL0529	SACOL0529 SACOL0530 rsml	SACOL0529	-0,50	0,23	0,82	0,08	0,71	1,17	1,77	1,06	hypothetical protein
Virulence	MgrA	SACOL0530	SACOL0529 SACOL0530 rsml	SACOL0530	-0,60	0,09	1,05	0,35	0,66	1,06	2,07	1,27	hypothetical protein
Virulence	MgrA	SACOL0531	SACOL0529 SACOL0530 rsml	rsml	-0,62	0,04	1,02	0,36	0,65	1,03	2,03	1,28	tetrapyrrole methylase
Virulence	MgrA	SACOL0664	SACOL0664	SACOL0664	-0,92	-0,40	0,94	0,37	0,53	0,76	1,92	1,29	deoxyribonuclease (pyrimidine dimer)
Virulence	MgrA	SACOL0809	SACOL0809	SACOL0809	-1,63	-1,35	0,29	0,01	0,32	0,39	1,22	1,01	diguanylate cyclase GGDEF domain protein
Virulence	MgrA	SACOL0871	SACOL0871	SACOL0871	-1,02	-0,08	-0,01	-0,89	0,49	0,95	0,99	0,54	hypothetical protein
Virulence	MgrA	SACOL0991	SACOL0990 oppB oppC oppD oppF oppA	oppB	-1,28	-0,35	0,00	-0,90	0,41	0,78	1,00	0,54	oligopeptide ABC transporter, membrane protein
Virulence	MgrA	SACOL0992	SACOL0990 oppB oppC oppD oppF oppA	oppC	-1,30	-0,36	0,11	-0,80	0,41	0,78	1,08	0,57	oligopeptide ABC transporter, membrane protein
Virulence	MgrA	SACOL0993	SACOL0990 oppB oppC oppD oppF oppA	oppD	-0,69	-0,21	-0,07	-0,53	0,62	0,86	0,95	0,69	oligopeptide ABC transporter, ATP-binding protein
Virulence	MgrA	SACOL0994	SACOL0990 oppB oppC oppD oppF oppA	oppF	-0,52	-0,17	-0,10	-0,44	0,70	0,89	0,93	0,74	oligopeptide ABC transporter, ATP-binding protein
Virulence	MgrA	SACOL0995	SACOL0990 oppB oppC oppD oppF oppA	oppA	-0,38	-0,04	-0,02	-0,34	0,77	0,97	0,99	0,79	oligopeptide ABC transporter, binding protein
Virulence	MgrA	SACOL1009	SACOL1009 relA1 ppnK yjbO mgtE cpaA	SACOL1009	0,34	-0,62	-0,82	0,12	1,27	0,65	0,57	1,09	hypothetical protein
Virulence	MgrA	SACOL1010	SACOL1009 relA1 ppnK yjbO mgtE cpaA	relA1	0,42	-0,81	-0,99	0,22	1,34	0,57	0,50	1,16	GTP diphosphokinase
Virulence	MgrA	SACOL1011	SACOL1009 relA1 ppnK yjbO mgtE cpaA	ppnK	0,70	-0,39	-0,78	0,29	1,63	0,76	0,58	1,22	inorganic polyphosphate/ATP-NAD kinase
Virulence	MgrA	SACOL1012	SACOL1009 relA1 ppnK yjbO mgtE cpaA	yjbO	0,90	-0,04	-0,67	0,26	1,87	0,97	0,63	1,20	RluD subfamily ribosomal large subunit pseudouridine synthase
Virulence	MgrA	SACOL1013	SACOL1009 relA1 ppnK yjbO mgtE cpaA	mgtE	0,80	0,10	-0,61	0,07	1,74	1,07	0,66	1,05	MgtE family magnesium (Mg2+)/cobalt (Co2+) transporter-E
Virulence	MgrA	SACOL1014	SACOL1009 relA1 ppnK yjbO mgtE cpaA	cpaA	0,68	0,04	-0,52	0,11	1,60	1,03	0,70	1,08	CPA2 family monovalent cation:proton (H+) antiporter-2
Virulence	MgrA	SACOL1101	SACOL1101	SACOL1101	2,02	1,04	-1,50	-0,52	4,05	2,06	0,35	0,70	hypothetical protein
Virulence	MgrA	SACOL1174	SACOL1174 SACOL1175	SACOL1174	-1,14	-0,76	-0,05	-0,40	0,45	0,59	0,97	0,76	hypothetical protein
Virulence	MgrA	SACOL1175	SACOL1174 SACOL1175	SACOL1175	-1,08	-0,74	-0,17	-0,48	0,47	0,60	0,89	0,72	hypothetical protein
Virulence	MgrA	SACOL1345	SACOL1345	SACOL1345	-1,72	-0,51	0,37	-0,80	0,30	0,70	1,29	0,57	hypothetical protein
Virulence	MgrA	SACOL1346	SACOL1346 SACOL1347 SACOL1348	SACOL1346	-1,62	-0,39	0,74	-0,47	0,33	0,76	1,67	0,72	hypothetical protein
Virulence	MgrA	SACOL1347	SACOL1346 SACOL1347 SACOL1348	SACOL1347	-1,42	-0,43	0,83	-0,15	0,37	0,74	1,78	0,90	hypothetical protein
Virulence	MgrA	SACOL1348	SACOL1346 SACOL1347 SACOL1348	SACOL1348	-1,32	-0,27	0,17	-0,79	0,40	0,83	1,13	0,58	hypothetical protein
Virulence	MgrA	SACOL1539	SACOL1539	SACOL1539	1,59	0,41	-2,10	-0,72	3,01	1,33	0,23	0,61	hypothetical protein
Virulence	MgrA	SACOL1660	SACOL1659 lamB accC accB ahs2 dur1	lamB	0,21	-0,13	0,17	0,48	1,15	0,91	1,13	1,39	possible lactam utilization protein
Virulence	MgrA	SACOL1661	SACOL1659 lamB accC accB ahs2 dur1	accC	-0,14	-0,35	0,08	0,27	0,91	0,78	1,06	1,21	biotin carboxylase
Virulence	MgrA	SACOL1662	SACOL1659 lamB accC accB ahs2 dur1	accB	-0,54	-0,40	0,20	0,04	0,69	0,76	1,15	1,03	acetyl-CoA carboxylase biotin carboxyl carrier subunit
Virulence	MgrA	SACOL1663	SACOL1659 lamB accC accB ahs2 dur1	ahs2	-0,72	-0,48	0,19	-0,06	0,61	0,72	1,14	0,96	Allophanate hydrolase subunit 2
Virulence	MgrA	SACOL1664	SACOL1659 lamB accC accB ahs2 dur1	dur1	-0,94	-0,30	0,03	-0,60	0,52	0,81	1,02	0,66	allophanate hydrolase subunit 1
Virulence	MgrA	SACOL1678	SACOL1678	SACOL1678	6,04	3,61	-2,64	-0,28	65,84	12,21	0,16	0,82	dehydrogenase
Virulence	MgrA	SACOL1847	SACOL1847	SACOL1847	-0,81	-1,33	-0,16	0,34	0,57	0,40	0,90	1,27	hypothetical protein
Virulence	MgrA	SACOL2066	SACOL2065 kdpC kdpB kdpA kdpF	kdpC	0,35	0,58	0,42	0,16	1,28	1,49	1,34	1,12	K+-transporting ATPase, C subunit
Virulence	MgrA	SACOL2067	SACOL2065 kdpC kdpB kdpA kdpF	kdpB	0,11	0,70	0,74	0,13	1,08	1,62	1,67	1,09	K+-transporting ATPase, B subunit
Virulence	MgrA	SACOL2068	SACOL2065 kdpC kdpB kdpA kdpF	kdpA	-0,98	0,57	0,83	-0,68	0,51	1,48	1,78	0,62	K+-transporting ATPase, A subunit
Virulence	MgrA	SACOL2069	SACOL2065 kdpC kdpB kdpA kdpF	kdpF	-0,72	-0,17	0,59	0,03	0,61	0,89	1,51	1,02	K+-transporting ATPase, F subunit
Virulence	MgrA	SACOL2164	SACOL2164	SACOL2164	-2,82	-0,95	1,25	-0,59	0,14	0,52	2,38	0,66	hypothetical membrane protein
Virulence	MgrA	SACOL2288	SACOL2288 sarY SACOL2290	SACOL2288	-1,05	-1,36	0,01	0,30	0,48	0,39	1,01	1,23	hypothetical protein
Virulence	MgrA	SACOL2289	SACOL2288 sarY SACOL2290	sarY	-0,77	-0,45	0,47	0,14	0,58	0,73	1,39	1,10	staphylococcal accessory regulator Y
Virulence	MgrA	SACOL2290	SACOL2288 sarY SACOL2290	SACOL2290	0,50	1,34	0,71	-0,09	1,42	2,53	1,64	0,94	AraC family transcriptional regulator
Virulence	MgrA	SACOL2439	SACOL2439	SACOL2439	3,31	0,63	-3,06	-0,40	9,92	1,55	0,12	0,76	hypothetical protein
Virulence	MgrA	SACOL2458	SACOL2458	SACOL2458	0,73	0,98	0,30	0,05	1,66	1,97	1,23	1,04	amino acid permease
Virulence	MgrA	SACOL2543	SACOL2543	SACOL2543	0,81	-0,38	0,03	0,86	1,76	0,77	1,02	1,82	hypothetical protein
Virulence	MgrA	SACOL2676	sasA	sasA	0,73	0,16	-0,75	-0,18	1,66	1,12	0,59	0,88	LPXTG cell wall surface anchor family protein
Virulence	MgrA	SACOL1806	sasC	sasC	-0,70	-0,34	0,04	-0,31	0,62	0,79	1,03	0,81	cell surface anchored protein
Virulence	MgrA	SACOL2675	secY2	secY2	1,21	1,46	-0,80	-0,92	2,31	2,75	0,57	0,53	Sec family Type I general secretory pathway protein SecY
Virulence	MgrA	SACOL2539	srtA	srtA	0,81	-0,44	-0,72	0,50	1,75	0,74	0,61	1,41	sortase
Virulence	MgrA	SACOL2279	yut	yut	2,04	0,46	-1,24	0,26	4,11	1,38	0,42	1,20	membrane-bound urea transporter
Virulence	MgrA	SACOL2090	ywpF	ywpF	-0,82	-0,96	-0,54	-0,35	0,56	0,51	0,69	0,78	hypothetical protein
Virulence	Rot	SACOL1984	aldH	aldH	2,38	1,05	-1,62	-0,30	5,20	2,07	0,33	0,81	aldehyde dehydrogenase
Virulence	Rot	SACOL1397	msrA2	msrA2	1,02	-0,04	-1,06	-0,02	2,03	0,97	0,48	0,99	methionine sulfoxide reductase A
Virulence	Rot	SACOL1308	porA porB	porA	1,00	0,92	-0,04	0,04	2,00	1,89	0,97	1,03	pyruvate flavodoxin/ferredoxin oxidoreductase domain-containing protein
Virulence	Rot	SACOL1309	porA porB	porB	1,22	1,08	-0,19	-0,05	2,33	2,11	0,88	0,97	2-oxoglutarate ferredoxin oxidoreductase subunit beta
Virulence	Rot	SACOL0970	rexB rexA	rexB	0,62	-0,33	-0,89	0,05	1,54	0,80	0,54	1,04	exonuclease RexB
Virulence	Rot	SACOL0971	rexB rexA	rexA	1,51	0,59	-0,87	0,03	2,84	1,51	0,55	1,02	exonuclease RexA
Virulence	Rot	SACOL0240	tarIJLS	tarI	-0,40	-0,67	-0,41	-0,14	0,76	0,63	0,75	0,91	2-C-methyl-D-erythritol 4-phosphate cytidylyltransferase
Virulence	Rot	SACOL0241	tarIJLS	tarJ	-0,10	-0,47	-0,44	-0,08	0,93	0,72	0,74	0,95	alcohol dehydrogenase, zinc-containing
Virulence	Rot	SACOL0242	tarIJLS	tarL	0,21	-0,34	-0,52	0,02	1,16	0,79	0,70	1,01	teichoic acid biosynthesis protein
Virulence	Rot	SACOL0243	tarIJLS	tarS	0,18	-0,21	-0,35	0,04	1,13	0,86	0,78	1,03	glycosyl transferase, group 2 family protein
Virulence	Rot	SACOL1842	yidD	yidD	1,39	0,36	-1,01	0,01	2,62	1,28	0,50	1,01	Putative membrane protein insertion efficiency factor
Virulence	Rot	SACOL1151	zapA cvpA	zapA	-0,62	-0,92	-0,40	-0,11	0,65	0,53	0,76	0,93	putative cell division protein, ZapA
Virulence	Rot	SACOL1152	zapA cvpA	cvpA	1,29	-0,54	-1,69	0,09	2,45	0,69	0,31	1,06	colicin V production protein
Virulence	SaeRS	SACOL0209	coa	coa	1,34	0,08	-1,23	0,00	2,53	1,06	0,43	1,00	staphylocoagulase
Virulence	SaeRS	SACOL1164	ecb	ecb	1,27	-0,43	-2,00	-0,39	2,40	0,74	0,25	0,76	fibrinogen binding-like protein
Virulence	SaeRS	SACOL1168	efb	efb	1,26	0,03	-1,75	-0,57	2,40	1,02	0,30	0,67	fibrinogen-binding protein
Virulence	SaeRS	SACOL1173	hla	hla	-1,74	-1,51	-0,76	-0,92	0,30	0,35	0,59	0,53	alpha-hemolysin
Virulence	SaeRS	SACOL2004	lukSF	lukF	-0,65	0,26	0,35	-0,51	0,64	1,20	1,27	0,70	possible leukocidin subunit
Virulence	SaeRS	SACOL2006	lukSF	lukS	-1,56	-0,33	0,28	-0,80	0,34	0,80	1,21	0,57	possible leukocidin subunit
Virulence	SaeRS	SACOL2002	map	map	1,00	0,72	1,55	1,83	2,00	1,65	2,93	3,56	cell surface protein MapW2
Virulence	SaeRS	SACOL0199	SACOL0199	SACOL0199	-1,17	-0,89	0,57	0,28	0,44	0,54	1,48	1,21	hypothetical protein
Virulence	SaeRS	SACOL0765	saeSRQP	saeS	1,13	-0,21	-1,51	-0,21	2,19	0,86	0,35	0,86	sensor histidine kinase SaeS
Virulence	SaeRS	SACOL0766	saeSRQP	saeR	1,02	-0,25	-1,47	-0,23	2,03	0,84	0,36	0,85	response regulator SaeR
Virulence	SaeRS	SACOL0767	saeSRQP	saeQ	0,99	-0,23	-1,43	-0,24	1,98	0,85	0,37	0,85	hypothetical membrane protein
Virulence	SaeRS	SACOL2418	sbi	sbi	1,47	-0,15	-2,28	-1,11	2,77	0,90	0,21	0,46	immunoglobulin G-binding protein SBI
Virulence	SaeRS	SACOL1169	scc	scc	1,44	0,01	-2,09	-0,70	2,72	1,01	0,23	0,62	fibrinogen-binding protein precursor-like protein
Virulence	SaeRS	SACOL0480	SCIN	SCIN	2,49	1,19	-1,68	-0,40	5,60	2,28	0,31	0,76	Staphylococcal complement inhibitor SCIN
Virulence	SarA	SACOL1712	abrB	abrB	1,70	1,35	0,03	0,36	3,24	2,55	1,02	1,28	putative ammonia monooxygenase
Virulence	SarA	SACOL1747	accA accD	accA	0,18	0,05	0,13	0,25	1,13	1,04	1,09	1,19	acetyl-CoA carboxylase carboxyltransferase subunit alpha
Virulence	SarA	SACOL1748	accA accD	accD	0,00	0,01	0,23	0,21	1,00	1,01	1,17	1,16	acetyl-CoA carboxylase subunit beta
Virulence	SarA	SACOL1437	cspA	cspA	-0,73	-0,48	0,32	0,07	0,60	0,72	1,25	1,05	CSD family cold shock protein
Virulence	SarA	SACOL1835	dkgB	dkgB	1,56	0,16	-1,67	-0,28	2,95	1,12	0,31	0,82	aldo/keto reductase
Virulence	SarA	SACOL1320	glpK glpD SACOL1322 miaA hfq	glpK	-0,46	-1,05	0,10	0,66	0,73	0,48	1,07	1,58	glycerol kinase
Virulence	SarA	SACOL1321	glpK glpD SACOL1322 miaA hfq	glpD	0,49	-0,49	-1,62	-0,65	1,41	0,71	0,33	0,64	aerobic glycerol-3-phosphate dehydrogenase
Virulence	SarA	SACOL1322	glpK glpD SACOL1322 miaA hfq	SACOL1322	0,04	-0,03	-0,87	-0,77	1,03	0,98	0,55	0,59	alpha/beta fold family hydrolase
Virulence	SarA	SACOL1323	glpK glpD SACOL1322 miaA hfq	miaA	-1,68	-1,38	-0,18	-0,47	0,31	0,38	0,88	0,72	tRNA delta(2)-isopentenylpyrophosphate transferase
Virulence	SarA	SACOL1324	glpK glpD SACOL1322 miaA hfq	hfq	-1,92	-1,44	-0,04	-0,48	0,26	0,37	0,97	0,72	hfq protein
Virulence	SarA	SACOL1427	SACOL1427	SACOL1427	-2,25	-1,19	1,19	0,13	0,21	0,44	2,28	1,09	ABC transporter ATP-binding protein
Virulence	SarA	SACOL2666	SACOL2666	SACOL2666	1,22	0,09	-1,30	-0,18	2,34	1,06	0,41	0,88	N-acetylmuramoyl-L-alanine amidase
Virulence	SarA	SACOL1003	trfA	trfA	2,56	1,45	-0,69	0,41	5,90	2,73	0,62	1,33	adaptor protein

Table S2: RNA-Seq transcriptome analysis of *S. aureus* COL wild type and the *mhqR* mutant before and after exposure to MHQ stress. *S. aureus* COL wild type and *mhqR* mutant cells were grown in 3 biological replicates and harvested before and after exposure to MHQ stress. The RNA-isolation and RNA-Seq transcriptome analysis was performed as described in the Methods section. Transcripts were analyzed for differential expression using the software DEseq2 included in the ReadXplorer v2.2 software. The signal intensity value (a-value) was calculated by log2 mean of normalized read counts and the signal intensity ratio (m-value) by log2 fold change. The evaluation of the differential RNAseq data was performed using an adjusted p-value cut-off of P ≤ 0.05 and a signal intensity ratio (m-value) cut-off of ≥ 0.6 or ≤ - 0.6. Genes were classified into regulons according to the RegPrecise database and the Aureowiki database. Only those regulons are shown with significant induced or repressed genes under MHQ-treatment in the *S. aureus* wild type which are bold-faced.
