# Supplementary material for: The MarR-Type Repressor MhqR Confers Quinone and Antimicrobial Resistance in Staphylococcus aureus
Source: Antioxid Redox Signal. 2019 Oct 17;31(16):1235–52. doi: 10.1089/ars.2019.7750 (PMC6798810; doi:10.1089/ars.2019.7750)
Supplement: Supplemental data [file Supp_Fig2.pdf]

Figure S2

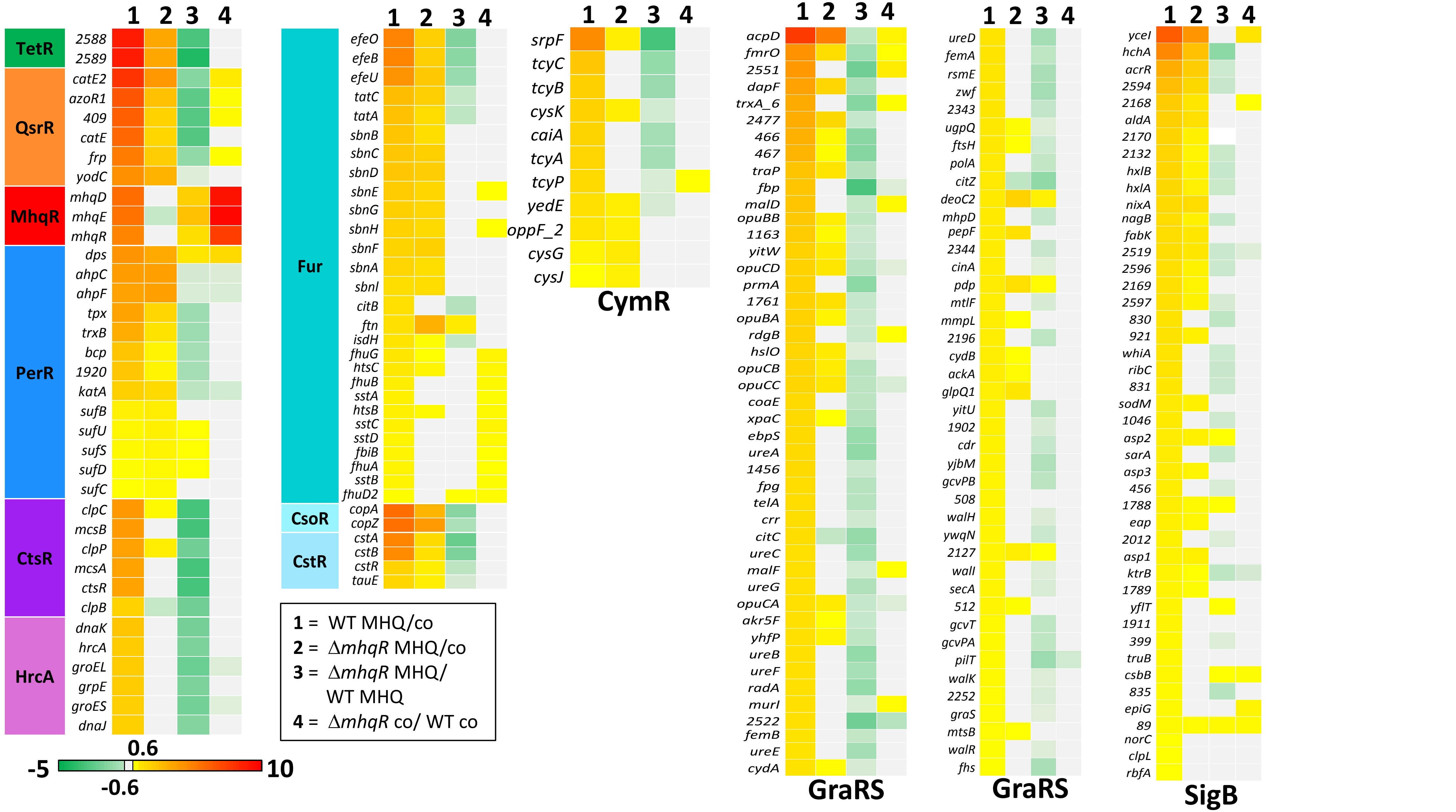

Fig. S2. RNA-seq transcriptomics of *S. aureus* COL wild type and the *mhqR* mutant under MHQ stress. For RNA-seq transcriptomics, *S. aureus* COL and the *mhqR* mutant were grown in RPMI1640 medium and treated with 45 µM MHQ stress for 30 min. The gene expression profile of highly induced or repressed genes under MHQ stress is shown in a colour-coded heatmap. Red and green indicate significantly induced and repressed transcripts, respectively (M-value ≥0.6 ≤-0.6; p-value≤0.05). The heat map displays log2 fold-changes of gene transcription between the wild type and the *mhqR* mutant under control and MHQ stress obtained from three biological replicates. The RNA-seq expression data are listed in Tables S1-S2.
