# Supplementary material for: The MarR-Type Repressor MhqR Confers Quinone and Antimicrobial Resistance in Staphylococcus aureus
Source: Antioxid Redox Signal. 2019 Oct 17;31(16):1235–52. doi: 10.1089/ars.2019.7750 (PMC6798810; doi:10.1089/ars.2019.7750)
Supplement: Supplemental data [file Supp_Fig3.pdf]

Figure S3A

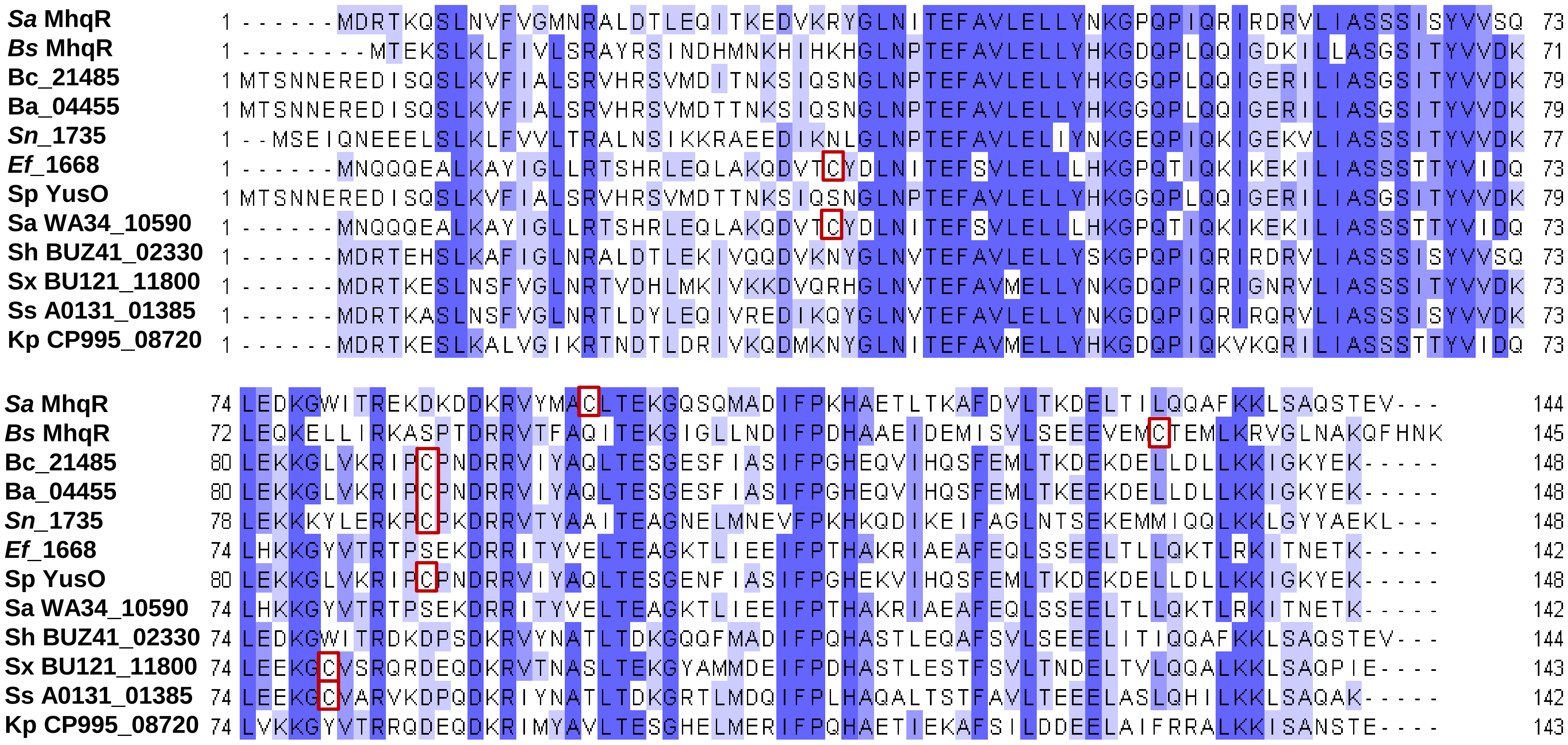

Fig. S3A. Protein sequence alignments of MhqR homologs in bacteria. Alignment was performed with ClustalΩ2 and presented in Jalview. Intensity of the blue color gradient is based on 50% sequence identity. The following protein sequences were aligned and the % identity to MhqR of *S. aureus* is given in parenthesis: MhqR (SACOL2531) of *S. aureus* COL, MhqR (BSU13670) of *B. subtilis* (38.1%), CN491_21485 of *B. cereus* (43.3%), CN907_04455 of *B. anthracis* (42.0%), HMPREF9372_1735 of *Sporosarcina newyorkensis* (43.2%), EF_1668 of *Enterococcus faecalis* (43.8%), YusO of *Streptococcus pneumoniae* (42.0%), WA34_10590 of *S. agalactiae* (43.8%), BUZ41_02330 of *S. haemolyticus* (77.8%), BU121_11800 of *S. xylosus* (66.7%), A0131_01385 of *S. saprophyticus* (67.4%) and CP995_08720 of *Klebsiella pneumoniae* (58.3%). The non-conserved cysteine residues in MhqR homologs are marked in red.

Figure S3B

```
Bs MhqD    1 MKHIYEKGTS-DNVLLLLHGTGGNEHDLLSLGRFIDPDAHLLGVRGSVLENGMPRFFKRLSEGVFDEKDL  69
Sa MhqD    1 MEHIFREGQNGAPTLILLHGTGGDEFDLLPLGEALNENYHLLSIRGQVSENGMNRYFKRLGEGVYDEEDL  70

Bs MhqD   70 VVRTRELKDFIDEAAETHQFNRGRVIAVGYSNGANIAASLLFHYKDVLKGAILHHPMVPIRGIELPDMAG 139
Sa MhqD   71 AFRGQELLTFIKEAAERYDFDIEKAVLVGFSNGSNIAINLMLRSEAPFKKALLYAPLYPVEVTSTKDLSD 140

Bs MhqD  140 LPVFIGAGKYDPLCTKEESEELYRYLRDSGASASVYWQDGGHQLTQHEAEQAREWYKEAIV 200
Sa MhqD  141 VSVLLSMGKHDPIVPLAASEQVINLFNTRGAQVEEVWVKGHEIT-ETGLTAGQQILGK--- 197
```

```
Bs MhqE    1 ----MKTEGLHHVTAFARDPQENLRFYTEVLGLRLVKKTVNFDDPGTYHFYFGNQNGDPGTIMTFFPFQGSGQGTVGKGQA  77
Sa MhqE    1 MINNHELLGIHHVTAMTDDAERNYKFFTEVLGMRLVKKTVNQDDIYTYHTFFADDVGSAGTDMTFFDFPNITKGQAGTNSI  81

Bs MhqE   78 GRVYFSVPSG-SLSFWKERLEKSGLSLEE-KTLFGEKGLIFDDTEDLPLAIMED----AKSGKSEWTPDGITTNEAITGMK 152
Sa MhqE   82 TRPSFRVPNDDALTYYEQRFDEFGVKHEGIQELFGKKVLPFEEVDGQVYQLISDELNEGVAPGVPWKNGPVPVDKAIYGLG 162

Bs MhqE  153 GVLLYSYDPQATIQLLTESFGYTKVAEEDQIVRLASS--AAVGGVIDVHLHPEKRGVGGYGTVHHVAFRTKKKEQ-AKWLP 230
Sa MhqE  163 PIEIKVSYFDDFKNILETVYGMTTIAHEDNVALLEVGEGGNGGQVILIKDDKGPAARQGYGEVHHVSFRVKDHDAIEAWAT 243

Bs MhqE  231 IIAENHLPSSEILDREYFTSVYFREKGGILFEIATDEPGFMTDETFAELGTSLKLPEWLEKHRQQITDILPEL------- 303
Sa MhqE  244 KYKEVGINNSGIVNRFYFEALYA-RVGHILIEISTDGPGFMEDEPYETLGEGLSLPPFLENKREYIESEVRPFNTKRQHG 322
```

```
Bs CatE    1 MTSIHED--THIGYAKLTIRSLERSLQFYCNVIGFQVLKKTDRQAELTAD--GKRVLLILEENPSAVVLPERSVTG  72
Sa CatE2   1 -MAFHDKTATQVTNIVLNVRDLDLMTTFYKNILGLSVKSSDDNTTVLSVGTGGHTLTLHLLEDGR---QTSPREAG  72

Bs CatE   73 LYHFAILLPDRKELGIALARLIEHGIAIGHGDHAVSEALYLSDPDGNGIEMYADRPRSTWQRDREGNYVMTTTAVD 148
Sa CatE2  73 LFHIAFLLPTTEDLANFLYFVAQKNMGIGAGDHLVSEALYFNDPEGNGIEVYRDRPSSSWEWQ-NGKVKMDTLEVD 147

Bs CatE  149 IEGLLEEAGDERKTSLPNDTIIGHIHLHVSDLKEAKAFYTDVLGFDIVGNYAGMSALFVSAGGYHHHIGLNIWAGR 224
Sa CatE2 148 SQTLLTHRTDEGWQGMPAKGMIGHLHLKTHDLDAAYQCYIEQLGFQHVSDFP--RALFMSTNHYHHHIAANTWQSN 221

Bs CatE  225 NAPPKPTNASGLDYYTVVLPHQEELDLVANRVKHAGYSIEETENSFRVKDPVSGAYITFVI 285
Sa CatE2 222 QARQNNSQSYGLTHVDIYQPDASTHTFTA----PEGFDITVHSNTDLVPEK---------- 268
```

Figure S3B (continued)

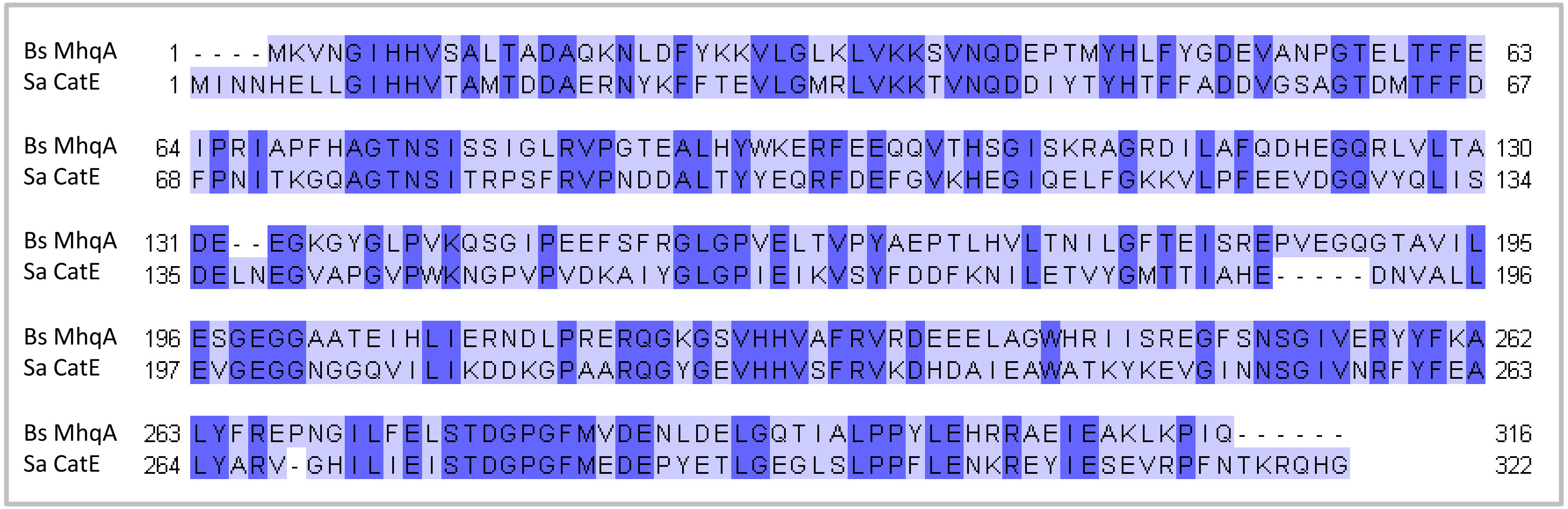

Fig. S3B. Multiple protein sequence alignments of the MhqR and QsrR-regulated phospholipase MhqD and dioxygenases MhqE, CatE and CatE2 of *S. aureus* with homologous enzymes of *B. subtilis*. The alignment was performed with ClustalΩ2 and is presented in Jalview. Intensity of the blue color gradient is based on 50% sequence identity. The following protein sequences were aligned and the % identity between *S. aureus* and *B. subtilis* enzymes is given in parenthesis: MhqE (SACOL2530) of *S. aureus* COL with MhqE (BSU19570) of *B. subtilis* (35.4%), MhqD (SACOL2529) of *S. aureus* COL with MhqD (BSU19560) of *B. subtilis* (38.8%) and CatE2 (SACOL2533) of *S. aureus* COL with CatE (BSU08240) of *B. subtilis* (34.8%) and CatE (SACOL0408) of *S. aureus* COL with MhqA (BSU12870) of *B. subtilis* (35.4%).
