# Supplementary material for: The MarR-Type Repressor MhqR Confers Quinone and Antimicrobial Resistance in Staphylococcus aureus
Source: Antioxid Redox Signal. 2019 Oct 17;31(16):1235–52. doi: 10.1089/ars.2019.7750 (PMC6798810; doi:10.1089/ars.2019.7750)
Supplement: Supplemental data [file Supp_Fig4.pdf]

Figure S4

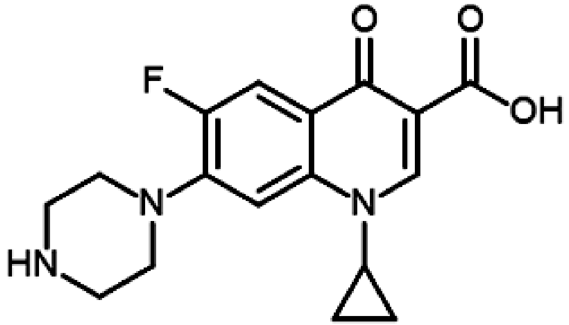

Ciprofloxacin

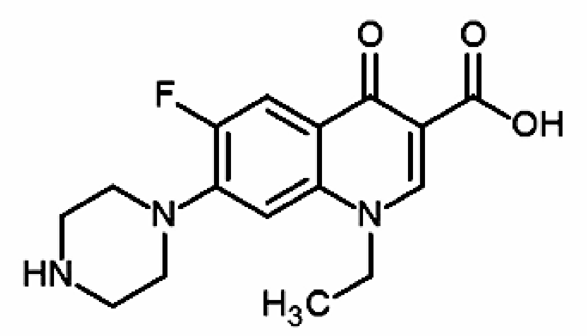

Norfloxacin

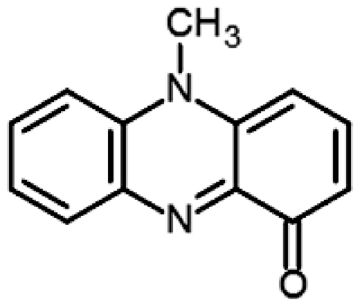

Pyocyanin

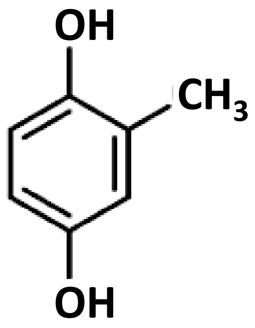

2-Methylhydroquinone

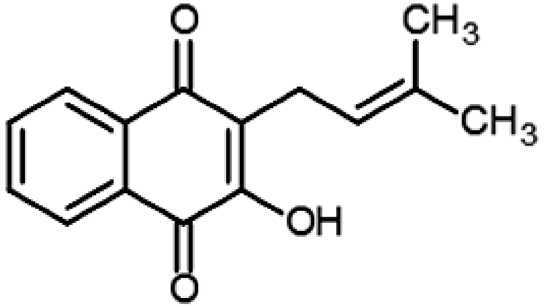

Lapachol

Fig. S4. The structures of quinone-derived antimicrobials used in this study. The structures of norfloaxin, ciprofloxacin, pyocyanin, MHQ and lapachol were drawn with 2D Sketcher (ChemDoodle®).
