# Supplementary material for: The MarR-Type Repressor MhqR Confers Quinone and Antimicrobial Resistance in Staphylococcus aureus
Source: Antioxid Redox Signal. 2019 Oct 17;31(16):1235–52. doi: 10.1089/ars.2019.7750 (PMC6798810; doi:10.1089/ars.2019.7750)
Supplement: Supplemental data [file Supp_Fig6.pdf]

Figure S6

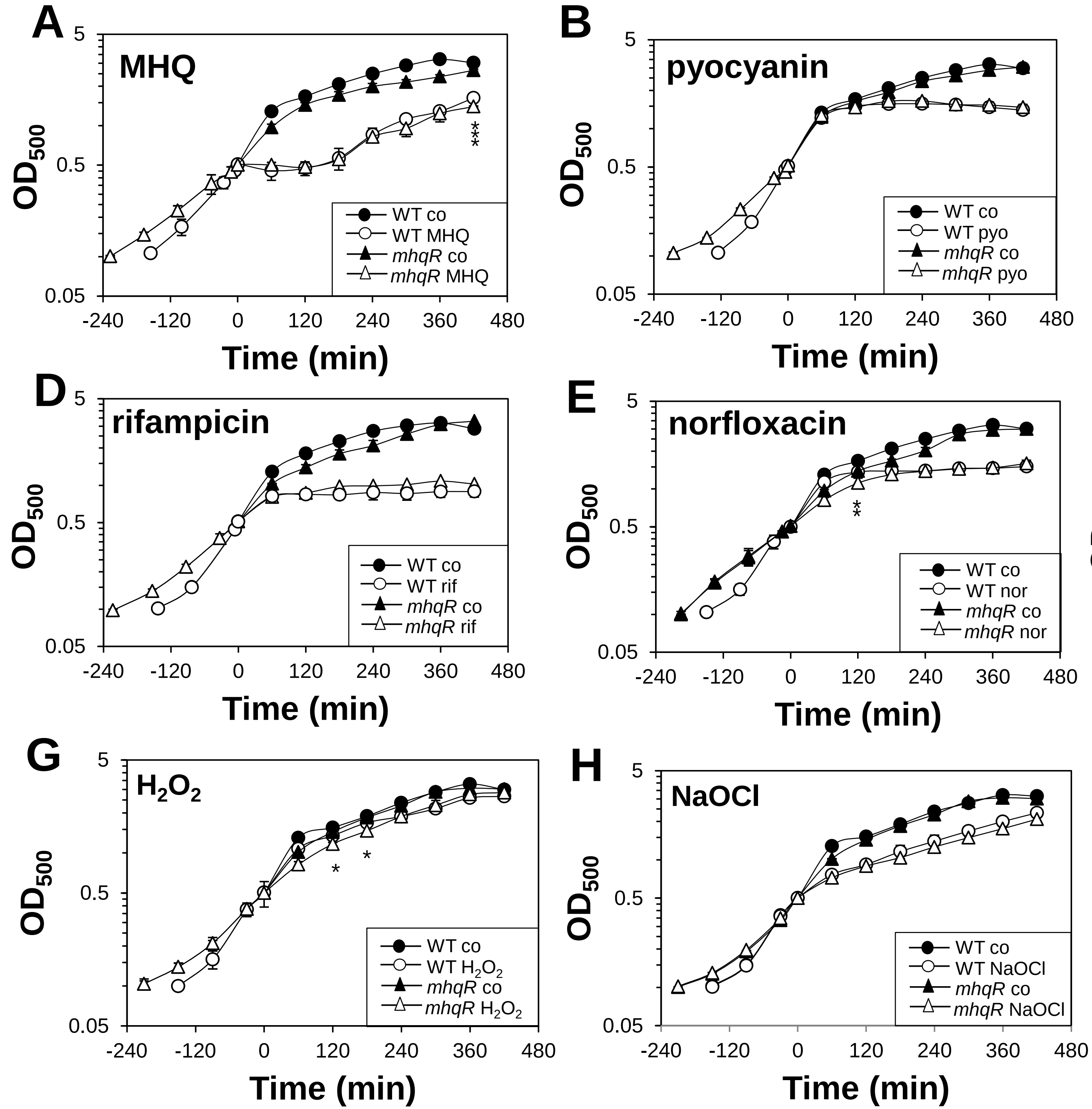

Fig. S6. The phenotype of the *mhqR* mutant can be restored to wild type level in the *mhqR* complemented strain. For the growth curves *S. aureus* COL wild type and the *mhqR* complemented strain (*mhqR*) were grown in RPMI medium. At an OD_{500} of 0.5 the strains were exposed to 50 µM MHQ, 76 µM pyocyanin, 90.5 µM ciprofloxacin, 0.05 µM rifampicin, 62.6 µM norfloxacin, 300 µM lapachol, 10 mM H_2O_2 and 1.5 mM NaOCl, respectively. The results are from 3-4 biological replicates. Error bars represent the standard deviation. *=p<0.05; **=p<0.01.
