# Supplementary material for: The MarR-Type Repressor MhqR Confers Quinone and Antimicrobial Resistance in Staphylococcus aureus
Source: Antioxid Redox Signal. 2019 Oct 17;31(16):1235–52. doi: 10.1089/ars.2019.7750 (PMC6798810; doi:10.1089/ars.2019.7750)
Supplement: Supplemental data [file Supp_Fig7.pdf]

Figure S7

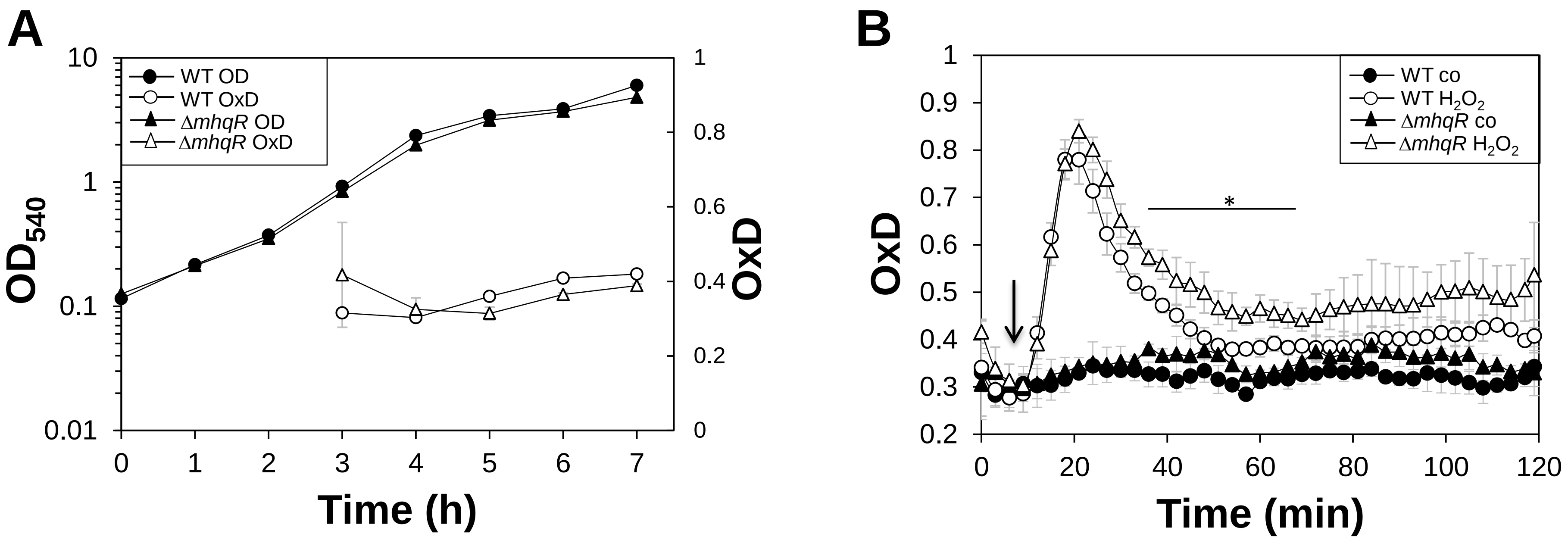

Fig. S7. The deletion of *mhqR* does not effect the basal BSH redox potential, but causes a slighly increased H_2O_2 response of the Brx-roGFP2 biosensor. (A, B) The BSH redox potential of the wild type and the *mhqR* mutant was measured with the Brx-roGFP2 biosensor along the growth curve and after exposure to 100 mM H_2O_2. The OxD was calculated based on 405/488 nm excitation ratios with emission at 510 nm and related to the fully oxidized and reduced controls. The *mhqR* mutant had a slightly increased biosensor response after exposure to 100 mM H_2O_2 and a significant delayed recovery of the reduced state. The mean values of four technical and three biological replicates are shown. Error bars represent the standard deviation. *$p<0.05$.
