# Supplementary material for: The MarR-Type Repressor MhqR Confers Quinone and Antimicrobial Resistance in Staphylococcus aureus
Source: Antioxid Redox Signal. 2019 Oct 17;31(16):1235–52. doi: 10.1089/ars.2019.7750 (PMC6798810; doi:10.1089/ars.2019.7750)
Supplement: Supplemental data [file Supp_Fig8.pdf]

Figure S8

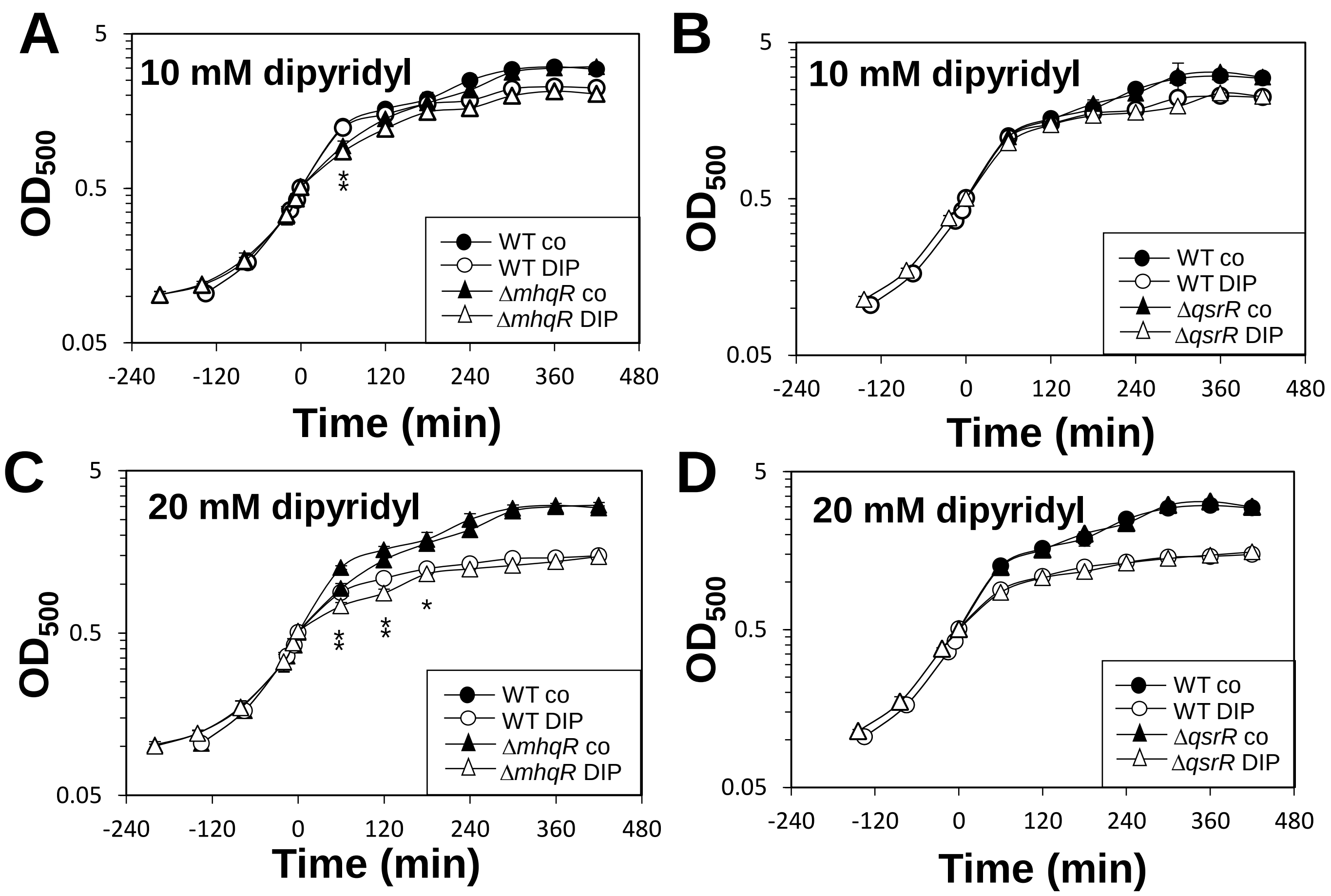

Fig. S8. The MhqR and QsrR regulons have no functions under iron limitation. For the growth curves, *S. aureus* COL wild type, the *mhqR* **(A, C)** and *qsrR* mutants **(B, D)** were grown in RPMI medium until an OD_{500} of 0.5 and treated with 10 mM **(A, B)** and 20 mM **(C, D)** of the iron-scavenger 2,2'-dipyridyl (DIP). The growth of the *mhqR* and *qsrR* mutants is not impaired under iron starvation. The results are from three biological replicates. Error bars represent the standard deviation. *p<0.05.
