# Supplementary material for: The MarR-Type Repressor MhqR Confers Quinone and Antimicrobial Resistance in Staphylococcus aureus
Source: Antioxid Redox Signal. 2019 Oct 17;31(16):1235–52. doi: 10.1089/ars.2019.7750 (PMC6798810; doi:10.1089/ars.2019.7750)
Supplement: Supplemental data [file Supp_Table3.pdf]

Table S3: Bacterial strains, phages and plasmids.

Strain	Description	Reference
Escherichia coli		
BL21(DE3)*plysS*	F-*ompT hsdS gal* (rb-mb+)DE3(*Sam7* Δ*nin5 lacUV5-T7 Gen1*)	(1)
BL21(DE3)*plysS* pET11b-*mhqR*	overexpression of MhqR	This study
BL21(DE3)*plysS* pET11b-*mhqRC95A*	overexpression of MhqRC95A	This study
Staphylococcus aureus		
COL	archaic HA-MRSA strain	(2, 3)
RN4220	restriction negative strain/MSSA cloning intermediate derived from 8325-4	(4)
COL pRB473	COL with empty plasmid	(5)
COL-Δ*mhqR*	COL *mhqR* deletion mutant	This study
COL-Δ*qsrR*	COL *qsrR* deletion mutant	This study
COL-Δ*mhqR*::pRB473-*mhqR*	COL *mhqR* complemented Δ*mhqR* mutant	This study
COL-Δ*mhqR*::pRB473-*mhqRC95A*	COL *mhqRC95A* complemented Δ*mhqR* mutant	This study
COL-Brx-roGFP2	COL pRB473-*brx-roGFP2*	(5)
COL- Δ*mhqR* Brx-roGFP2	COL *mhqR* mutant pRB473-*brx-roGFP2*	This study
**Bacteriophages**		
Staphylococcus phage 81		(6)
**plasmids**		
pMAD	*S. aureus* shuttle vector, Ery^r	(7)
pET11b	*E. coli* expression plasmid	Novagen
pRB473	pRB373-derivative, *E. coli* /*S. aureus* shuttle vector, containing xylose -inducible P_{Xyl} promoter Amp^r, Cm^r	(8, 9)
pMAD-*qsrR*	pMAD-derivative, *S. aureus* shuttle vector, *qsrR* deletion, Ery^r	This study
pMAD-mhq*R*	pMAD-derivative, *S. aureus* shuttle vector, *mhqR* deletion, Ery^r	This study
pET11b-*mhqR*	pET11b-derivative for overexpression of His-tagged MhqR	This study
pET11b-*mhqRC95A*	pET11b-derivative for overexpression of His-tagged MhqRC95A	This study
pRB473-*mhqR*	pRB473-derivative expressing *mhqR* under P_{Xyl}	This study
pRB473-*mhqRC95A*	pRB473-derivative expressing *mhqRC95A* under P_{Xyl}	This study
pRB473-*brx-roGFP2*	pRB473-derivative expressing *brx-roGFP2* under P_{Xyl}	(5)

^{R}: resistant, Amp: ampicillin, Ery: erythromycin, Cm: chloramphenicol

Supplementary References

1. Studier FW, Moffatt BA. 1986. Use of bacteriophage T7 RNA polymerase to direct selective high-level expression of cloned genes. J Mol Biol 189:113-30.

2. Shafer WM, Iandolo JJ. 1979. Genetics of staphylococcal enterotoxin B in methicillin-resistant isolates of *Staphylococcus aureus*. Infect Immun 25:902-11.

3. Gill SR, Fouts DE, Archer GL, Mongodin EF, Deboy RT, Ravel J, Paulsen IT, Kolonay JF, Brinkac L, Beanan M, Dodson RJ, Daugherty SC, Madupu R, Angiuoli SV, Durkin AS, Haft DH, Vamathevan J, Khouri H, Utterback T, Lee C, Dimitrov G, Jiang L, Qin H, Weidman J, Tran K, Kang K, Hance IR, Nelson KE, Fraser CM. 2005. Insights on evolution of virulence and resistance from the complete genome analysis of an early methicillin-resistant *Staphylococcus aureus* strain and a biofilm-producing methicillin-resistant *Staphylococcus epidermidis* strain. J Bacteriol 187:2426-38.

4. Kreiswirth BN, Lofdahl S, Betley MJ, O'Reilly M, Schlievert PM, Bergdoll MS, Novick RP. 1983. The toxic shock syndrome exotoxin structural gene is not detectably transmitted by a prophage. Nature 305:709-12.

5. Loi VV, Harms M, Müller M, Huyen NTT, Hamilton CJ, Hochgräfe F, Pane-Farre J, Antelmann H. 2017. Real-time imaging of the bacillithiol redox potential in the human pathogen *Staphylococcus aureus* using a genetically encoded bacilliredoxin-fused redox biosensor. Antioxid Redox Signal 26:835-848.

6. Rosenblum ED, Tyrone S. 1964. Serology, density, and morphology of staphylococcal phages. J Bacteriol 88:1737-42.

7. Arnaud M, Chastanet A, Debarbouille M. 2004. New vector for efficient allelic replacement in naturally nontransformable, low-GC-content, gram-positive bacteria. Appl Environ Microbiol 70:6887-91.

8. Brückner R, Wagner E, Götz F. 1993. Characterization of a sucrase gene from *Staphylococcus xylosus*. J Bacteriol 175:851-7.

9. Pöther DC, Gierok P, Harms M, Mostertz J, Hochgräfe F, Antelmann H, Hamilton CJ, Borovok I, Lalk M, Aharonowitz Y, Hecker M. 2013. Distribution and infection-related functions of bacillithiol in *Staphylococcus aureus*. Int J Med Microbiol 303:114-23.
