# Supplementary material for: The MarR-Type Repressor MhqR Confers Quinone and Antimicrobial Resistance in Staphylococcus aureus
Source: Antioxid Redox Signal. 2019 Oct 17;31(16):1235–52. doi: 10.1089/ars.2019.7750 (PMC6798810; doi:10.1089/ars.2019.7750)
Supplement: Supplemental data [file Supp_Table4.pdf]

Table S4: Oligonucleotide sequences

Primer name	Sequence (5' to 3')
SACOL2531-pET- For-NheI	CTAGCTAGCATGGATCGAACGAAACAATCTC
SACOL2531-pET-Rev-BamHI	CGCGGATCCTTAGTGATGGTGATGGTGATGCACTTCTGTAGATT GTGCACTT
SACOL2531-pET-C95A-Rev	ACCTTTTTCAGTTAACGCAGCCATATATACACG
SACOL2531-pET-C95A-For	CGTGTATATATGGCTGCGTTAACTGAAAAAGGT
SACOL2531-pMAD-For-BglII	CGCAGATCTTTTAAGTAGTTTTTTGCCAACAC
SACOL2531-pMAD-Rev-SalI	CCAGTCGACTGTTTAACACCAAACTCATCAAA
SACOL2531-pMAD-F1 -Rev	GTCTCAGCATGCTTAGGGAAACTTTTGTAATTTGCTCTAATGTG
SACOL2531-pMAD-F2-For	CACATTAGAGCAAATTACAAAAGTTTCCCTAAGCATGCTGAGAC
SACOL2531-pRB-For-BamHI	TAGGGATCCTAAAATAAAAAAGTTGGTGATCATATGGATCGAAC GAAACAAT
SACOL2531-pRB-Rev-KpnI	CTCGGTACCTTACACTTCTGTAGATTGTGCACTT
emsa2531-for	ACATATCAACTCCTATCATGATT
emsa2531-rev	CGTTCGATCCATATGATCACC
EMSA_m1 _for	TATCCCTAAATCGAAATA
EMSA_m1 _rev	TATTTCGATTTAGGGATA
EMSA_m2 _for	TATCTCGAAATAGGAATA
EMSA_m2 _rev	TATTCCTATTTCGAGATA
SACOL2529-for	GCATGGTACAGGTGGTGATG
SACOL2529-rev	CTAATACGACTCACTATAGGGAGATGTGCCCCACGTGTATTAAA
SACOL2115-pMAD-for-BglII	CGCAGATCTTCGCATCAATAATAATGCGCC
SACOL2115-pMAD-F1-Rev	CTTCAATTGGATGTAAAGCCTCGGACATACTTCCATCATCTTC
SACOL2115-pMAD-F2-For	GAAGATGATGGAAGTATGTCCGAGGCTTTACATCCAATTGAAG
SACOL2115-pMAD-rev-SalI	CCAGTCGACCTTTCACTCCTCAAGAATGATT
trxA-for	GCAACATGGTGTGGTCCAT
trxA-rev	CTAATACGACTCACTATAGGGAGATGGTTGGAAACCAACAACTT
