# Supplementary material for: The MarR-Type Repressor MhqR Confers Quinone and Antimicrobial Resistance in Staphylococcus aureus
Source: Antioxid Redox Signal. 2019 Oct 17;31(16):1235–52. doi: 10.1089/ars.2019.7750 (PMC6798810; doi:10.1089/ars.2019.7750)
Supplement: Supplemental data [file Supp_Fig5.pdf]

Figure S5

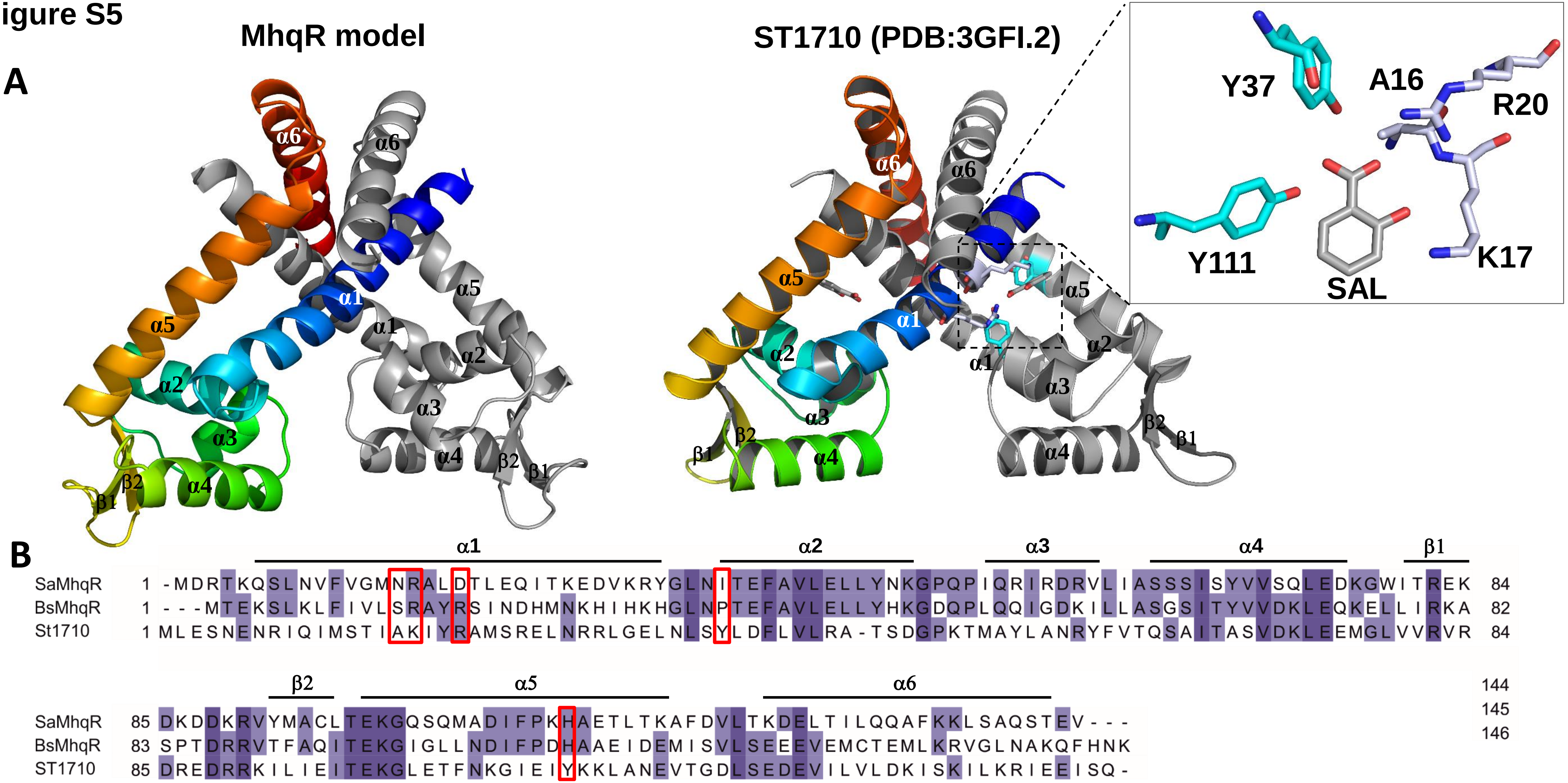

Figure S5: **Structural model of MhqR in comparison to the ST1710–salicylate complex (A) and alignment of MhqR homologs with ST1710 (B). (A)**The structural model of MhqR was generated based on the template of ST1710 from *Sulfolobus tokodaii* (3GFI) using SWISS-MODEL and visualized with PyMOL. The inset shows the salicylate binding site of ST1710 as adapted from Kumarevel *et al.*, NAR 37: 4723–4735, 2009. **(B)** *S. aureus* MhqR (SACOL2531) was aligned with *B. subtilis* MhqR (MHQR_BACSU) (39.44% identity) and ST1710 (Q96ZY1_SULTO) of *Sulfolobus tokodaii* (18.18% identity) using Clustal Omega and Jalview. Intensity of the blue colour gradient is based on 50% sequence identity. Red boxes denote salicylate contact residues A16, K17, R20, Y37, Y111 in ST1710 in the ligand binding pocket of ST1710.
